# Synthesis and Characterization of Three-Dimensional Uranyl Coordination Polymers Containing Selenophene and Tellurophene Linkers

**DOI:** 10.1021/acs.inorgchem.6c02207

**Published:** 2026-08-03

**Authors:** Ecem Çelik, Vamsi K. Karapala, Brett Lottes, Michael A. Sinnwell, Korey P. Carter

**Affiliations:** † Department of Chemistry, 4083 University of Iowa, Iowa City, Iowa 52242, United States; ‡ Office of The Vice President for Research, University of Iowa, Iowa City, Iowa 52242, United States

## Abstract

Herein, we describe the synthesis and characterization of three-dimensional uranyl coordination polymers (CPs) featuring 2,5-selenophenedicarboxylic acid (2,5-SeDC) and 2,5-tellurophenedicarboxylic acid (2,5-TeDC) linkers. All compounds were analyzed structurally using single-crystal and powder X-ray diffraction and were spectroscopically characterized via Raman and infrared spectroscopies, thermogravimetric analysis, and scanning electron microscopy. Thermal analysis revealed that uranyl–2,5-SeDC and 2,5-TeDC-based CPs were stable between 250–350 °C. These measurements also led to the discovery of a ligand-induced redox transformation to a reduced uraninite-type (UO_2_/UO_2+x_) phase under inert atmosphere (N_2_), wherein heating of U­(VI) hybrid materials featuring 2,5-SeDC and 2,5-TeDC did not lead to U­(VI) oxides after framework degradation, rather U­(IV) was formed at ambient pressure in the absence of chemical reductants. This redox transformation does not occur when hybrid materials are prepared with 2,5-thiophenedicarboxylic acid (2,5-TDC) or 2,5-furandicarboxylic acid (2,5-FDC); however, physical mixing of 2,5-TeDC with uranyl-2,5-TDC or uranyl-2,5-FDC compounds in a 1:1 stoichiometric ratio does lead to the formation of a UO_2_/UO_2+x_ phase. Pre- and post-TGA SEM/EDS analyses revealed that Se and Te content decrease to near-background levels after heating under N_2_, and these findings are consistent with decomposition pathways involving volatile chalcogen-containing intermediates that facilitate spontaneous *in situ* U­(VI) to U­(IV) reduction under mild conditions.

## Graphical abstract

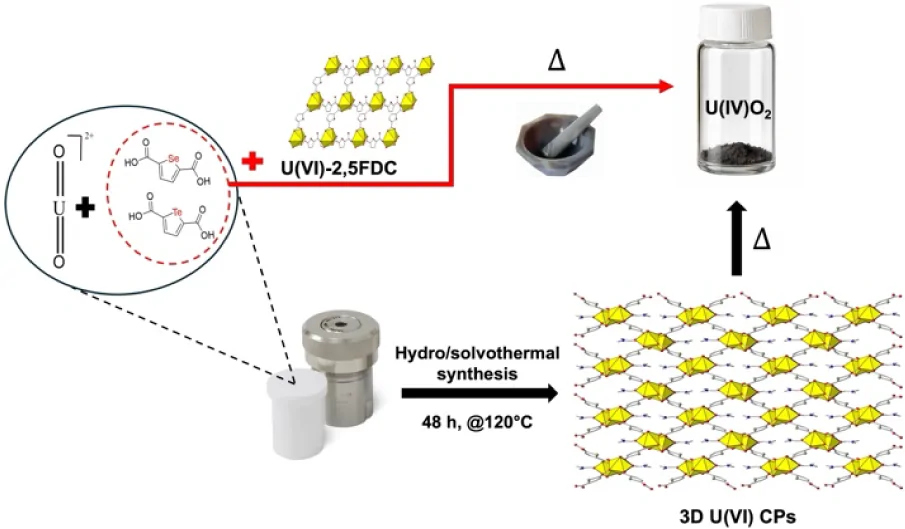

## Introduction

Uranyl crystal chemistry is a topic of sustained research interest due to the central role of the hexavalent uranyl cation (UO_2_^2+^) in the nuclear fuel cycle and numerous environmental processes.^1–7^ Coordination chemistry with the uranyl cation is largely governed by its strong interactions with oxo atoms in axial positions that generally limit further binding to the equatorial plane.^8–13^ Despite the linear nature of the UO_2_^2+^ species, it can form complexes with multidentate ligands leading to a range of different structural motifs and reactivities that are particularly useful in hybrid materials such as coordination polymers (CPs) and metal–organic frameworks (MOFs).^1,2,14–21^ MOFs possess tunable porosity, high surface area, and diverse chemical functionalities, which make them an attractive platform for investigating fundamental aspects of actinide chemistry as they can be tailored to suit specific applications by meticulously selecting metal ions, organic linkers, and synthetic conditions.^22–29^ Uranyl MOFs have garnered interest for their ability to create porous architectures, exceptional thermal stability, and diverse chemical properties, yet these species are rare owing to the propensity of the terminal oxygen atoms of UO_2_^2+^ to restrict material dimensionality.^1,2,30–33^ As a result, within uranyl hybrid materials one-dimensional (1D) chain and two-dimensional (2D) sheet-like coordination polymers are known to predominate,^1,2,9,10,34,35^ with the use of flexible organic ligands the primary workaround that can provide a pathway to generating uranyl three-dimensional (3D) frameworks.^36–41^

Advances in hybrid material and MOF synthesis that have occurred over the past two decades have been extended to the actinide series, which has led to a range of novel compounds with distinct properties made possible by the use of organic linkers with various functional groups.^42–46^ Moieties included in actinide hybrid materials have included carboxylates,^47–49^ phosphonates,^50–52^ sulfonates,^53^ and neutral nitrogen donors,^13,54–57^ yet use of linkers featuring these functional groups with U­(VI) cations only rarely yields 3D structures.^1,2^ To extend uranyl chemistry to three dimensions necessitates the use of flexible linkers as demonstrated by the uranyl MOFs ZnTCPP-U2, NU-1307, NU-1301, and RPL-1, which feature tetrakis­(4-carboxyphenyl)­porphyrin (TCPP), tetrakis­(4-carboxyphenyl)­dibromobenzene (TCPB), and 4,4′,4″-(2,4,6-trimethylbenzene-1,3,5-triyl)­tribenzoate (TMTB), respectively.^58,59^ Another route for tuning the structure and properties of hybrid materials is via the use of bent chalcogenophene-based linkers, which are heterocyclic compounds incorporating group 16 elements such as oxygen (O), sulfur (S), selenium (Se), and tellurium (Te). Pairing this class of linkers with transition metals leads to MOFs with distinct structural topologies and properties,^60–64^ and moving to heterocycles with larger chalcogen atoms (Se and Te) offers attractive advantages in CP and MOF design due to their unique electronic properties and tunable bandgaps. Whereas furan and thiophene-based dicarboxylic acid derivatives have been explored in uranyl coordination compounds,^47,65–68^ and shown to support π-stacking and flexible coordination modes, extensions to heavier chalcogen-containing analogues have not been explored. In fact, the synthesis of hybrid materials incorporating Se- and Te-based linkers remains understudied across the periodic table, with only a few examples reported in the Cambridge Structural Database.^61,69,70^ This limited development is likely related, in part, to the less established synthetic chemistry and additional stability and handling considerations associated with heavier Se- and Te-containing heterocycles compared with their lighter furan and thiophene analogues.^71–73^ Nevertheless, incorporation of Se and Te linkers within uranyl CPs and MOFs introduces the possibility of different types of bonding and noncovalent interactions, due to the larger size, polarizability, and redox tunability of these elements. Collectively, these characteristics present promising avenues for designing actinide materials with enhanced functionality, where control of redox chemistry and electronic flexibility are crucial, and thus we endeavored to expand the chemical space of uranyl hybrid materials to include Se- and Te-containing chalcogenophene linkers.

Herein we describe the synthesis and characterization of a series of uranyl hybrid materials employing either 2,5-selenophenedicarboxylic acid (2,5-SeDC) or 2,5-tellurophenedicarboxylic acid (2,5-TeDC) as bridging linkers. Remarkably, all eight compounds are 3D coordination polymers or frameworks and this shift in structural dimensionality, from one- and two-dimensional materials that are generally observed with U­(VI) cations, underscores the ability of Se- and Te-based linkers to overcome geometric constraints and promote the formation of novel uranyl architectures. This behavior contrasts with prior studies of thiophenedicarboxylate-based uranyl systems,^47,67,68^ which exhibit a strong tendency toward diperiodic architectures, often forming 2D coordination polymers with varying degrees of interpenetration or polycatenation. Thermogravimetric analysis (TGA) revealed that the uranyl-chalcogenophene-based CPs and MOFs described herein remain stable up to 250–350 °C, and most notably, undergo a ligand-induced redox transformation to a uraninite-type (UO_2_/UO_2+x_) phase under inert atmosphere (N_2_), wherein the heating of U­(VI) hybrid materials featuring 2,5-SeDC and 2,5-TeDC does not lead to U­(VI) oxides after framework degradation, rather U­(IV) is formed without the need for high pressures or addition of chemical reductants. This transformation was not observed in analogous coordination polymers featuring 2,5-thiophenedicarboxylic acid (2,5-TDC) or 2,5-furandicarboxylic acid (2,5-FDC), indicating that incorporation of Se and Te within framework linkers is integral for enabling uranium reduction. Pre/post-TGA field emission scanning electron microscopy (FE-SEM) measurements revealed that Te content drops to near zero after heating under N_2_, while Se content decreases but remains detectable, and powder X-ray diffraction (PXRD) patterns of post-TGA products revealed no crystalline Se or Te phases, which suggests volatile species (e.g., hydrides) may drive *in situ* U­(IV) formation. Overall, this work demonstrates that ligand design can be strategically leveraged to access new structural motifs and redox behavior in uranyl hybrid materials and establishes a platform for the development of actinide frameworks with built-in electronic tunability; a result of potential relevance across fields ranging from nuclear energy to the environmental remediation of radionuclides.

## Experimental Section

### Materials and Methods

#### Ligand Synthesis and Characterization

Chemicals used in the synthesis of 2,5-SeDC and 2,5-TeDC include selenophene (Acros Organics, 99%), *n*-butyllithium, *n*-BuLi, 2.5 M in hexanes (Sigma-Aldrich), *sec*-butyllithium, *s*-BuLi, 1.4 M in cyclohexane (Sigma-Aldrich), *N*,*N*,*N*′,*N*′-tetramethylethylenediamine, TMEDA, (TCI America, 98%), tellurium (Te) (Strem Chemicals Inc., 99.8%), sodium borohydride, NaBH_4_, (Beantown Chemical Inc., 98%), potassium hydroxide (KOH) (ACS reagent, ≥85%, pellets, Sigma-Aldrich), and 1,4-bis­(trimethylsilyl)-1,3-butadiyne (synthesized in-house). Commercially available reagents were used as received without further purification. Tetrahydrofuran (THF) (Sigma-Aldrich, 99.9%) and *n*-pentane (Sigma-Aldrich, ≥99%) were dried by passage through columns of activated alumina, while water was obtained from a Milli-Q IQ 7000 purification system.

^1^H and ^13^C NMR spectra were recorded on either Bruker Fourier 300 MHz, Avance NEO 400 MHz or Bruker Avance III 500 MHz spectrometers, while ^125^Te spectra were recorded on a Bruker Avance III 500 MHz spectrometer. All spectra were internally referenced to deuterated solvents or known standards (Ph_2_Te_2_ in CDCl_3_ for Te), and chemical shifts are reported in parts per million (δ ppm). Multiplicities are indicated by **s** (singlet), **br** (broad), **dd** (doublet of doublets), and **m** (multiplet).

***Note***: Appropriate attention should be given to the organoselenium and organotellurium compounds while handling them, and even though their toxicity and biological solubility are low, they should be handled with proper personal protective equipment.

The 2,5-SeDC and 2,5-TeDC linkers used in this study were synthesized in-house, and full synthetic details are provided in Schemes S1 and S2 and the Supporting Information (SI).

#### Hybrid Material Synthesis

**CAUTION!** *Uranyl nitrate hexahydrate ((UO*_2_ *)­(NO*_3_)_2_ *·6H*_2_ *O) and uranyl acetate dihydrate ((UO*_2_ *)­(CH*_3_ *COO)*_2_ *·2H*_2_ *O) used in this study contain* ^238^ *U (t*_*1/2*_ *= 4.47 × 10* ^*9*^ *years), which is an α-emitting radionuclide that should be manipulated only in a specifically designated facility in accordance with appropriate safety controls. All reactions and measurements were conducted in University of Iowa radiological laboratories and/or using multiple containment procedures.*

All materials used in the synthesis of uranyl hybrid materials were purchased from commercial vendors and used as received, other than the organic linkers 2,5-SeDC and 2,5-TeDC. This includes uranyl nitrate hexahydrate, UO_2_(NO_3_)_2_·6H_2_O, (International Bio-Analytical Industries Inc., 98%), uranyl acetate dihydrate, UO_2_(CH_3_COO)_2_·2H_2_O, (International Bio-Analytical Industries Inc., 98%), N,N-dimethylformamide, DMF, (Fisher Scientific, ≥99.9%), and dimethyl sulfoxide, DMSO, (Fisher Scientific, ≥ 99.7%). Water used in all reactions was obtained from a Milli-Q IQ 7000 purification system.

Each compound was hydro- or solvothermally synthesized inside a 23 mL Parr autoclave by placing it into an oven at 120 °C for 48 h. Upon cooling the reaction vessels to room temperature, pale-yellow rectangular plate-like crystals were separated from the bulk product for all eight compounds. Since the synthetic procedures for the entire series are identical except for the linker used, only the syntheses of three representative examples with 2,5-SeDC, [(UO_2_)­(C_6_H_2_O_4_Se)­(C_3_H_7_NO)]_n_ (**1**), [(UO_2_)­(C_6_H_2_O_4_Se)­(C_2_H_6_OS)]_n_ (**3**), and [(UO_2_)­(C_6_H_2_O_4_Se)]_n_·2H_2_O (**5**) will be described herein. Analogous procedures for 2,5-TeDC compounds and minor phases (compounds **7** and **8**) are included in the SI.

Compounds **1**, **3**, and **5** were prepared using 23 mL Parr autoclaves where each reaction vessel was heated according to the procedure described above. After the reaction vessels were removed from the oven and allowed to cool to room temperature overnight, crystals suitable for single-crystal X-ray diffraction analysis were isolated from the mother liquor and placed directly into high-viscosity NVH oil.

##### Synthesis of [(UO_2_)­(C_6_H_2_O_4_Se)­(C_3_H_7_NO)]_n_ (**1**)

Synthesis of compound **1** involved 0.0251 g (50 μmol) UO_2_(NO_3_)_2_·6H_2_O, 0.0110 g (50 μmol) 2,5-SeDC, 0.8147 g H_2_O, and 41 μL DMF.

##### Synthesis of [(UO_2_)­(C_6_H_2_O_4_Se)­(C_2_H_6_OS)]_n_ (**3**)

Compound **3** was synthesized by combining 0.0217 g (50 μmol) UO_2_(CH_3_COO)_2_·2H_2_O, 0.0110 g (50 μmol) 2,5-SeDC, 0.7830 g H_2_O, and 40 μL DMSO.

##### Synthesis of [(UO_2_)_2_(C_6_H_2_O_4_Se)_2_]_n_·2H_2_O (**5**)

Synthesis of compound **5** involved combining 0.0217 g (50 μmol) UO_2_(CH_3_COO)_2_·2H_2_O, 0.0110 g (50 μmol) 2,5-SeDC, and 0.7653 g H_2_O.

### Characterization

#### X-ray Structure Determination

Structural analysis on compound **2** was conducted on a Bruker D8 Quest single crystal X-ray diffractometer equipped with a microfocus X-ray beam (Mo Kα; λ = 0.71073 Å) and a CMOS detector, while compounds **1** and **3**–**8** were analyzed on a Bruker D8 Venture Duo single crystal X-ray diffractometer equipped with an IμS 3.0 microfocus source operated at 75 W (50 kV, 1.5 mA) to generate Mo Kα radiation (λ = 0.71073 Å) and a PHOTON III detector. All data were collected at low temperature using Oxford Systems low temperature cryostreams where crystals from each sample were placed under a cold nitrogen stream maintained at 100(2) K. Suitable single crystals for **1**–**8** were selected using a Zeiss Stemi 305 microscope and mounted with a small amount of NVH immersion oil on either Hampton Research nylon loops or MiTeGen micromounts before being transferred to SCXRD instruments. Samples were optically centered with the aid of a video camera to ensure that no translations were observed as the crystals were rotated through all positions. Peak intensity data were corrected for Lorentz, polarization, background, and absorption effects using either the TWINABS or SADABS multiscan methods within the Bruker APEX 3, 4, and 5 software packages.^74,75^ Initial structure solutions were generated using the intrinsic phasing method and further refined with the SHELXL least-squares method within the Olex2 1.5 software package.^76,77^ Compounds **1**–**7** were refined as two-component inversion twins, with appropriate twin laws applied and BASF parameters refined individually for each structure. Absorption corrections were applied using TWINABS for compound **1** and SADABS for compounds **2**–**8**. All non-hydrogen atoms in compounds **1**–**8** were refined anisotropically, with restraints (SADI, SIMU, and/or RIGU) applied where necessary. Compound **2** contains a disordered DMF molecule that was modeled over two positions using SADI, SIMU, and RIGU restraints. Compound **6** exhibits positional disorder of the tellurophene ring, modeled over two sites with refined occupancies of approximately 0.75 and 0.25 that required SADI, SIMU, and RIGU restraints. Compound **8**, obtained as a minor product, contains a disordered tellurophene moiety modeled with fixed occupancies and stabilized using planarity (FLAT) and RIGU restraints. Compounds **1** and **5** required RIGU restraints to stabilize anisotropic displacement parameters of the chalcogenophene ligands, while compounds **3**, **4**, and **7** did not require positional disorder modeling. Hydrogen atoms bound to carbon atoms in compounds **1**–**8** were placed in calculated positions and refined using riding models, with methyl groups treated as rotating groups where applicable. Hydrogen atoms associated with lattice water molecules were treated separately. For compounds **6** and **7**, these hydrogen atoms were located from difference Fourier maps and then geometrically constrained, while the lattice water hydrogen atoms in compounds **5** and **8** could not be reliably located and were therefore not included in the refinement models. More detailed refinement information for each structure is provided in the SI. Worthy of additional comment, the final CIFs for compounds **2** and **4** do contain one and two CheckCIF Alert A alerts, respectively, due to residual electron density near atoms within the structures. This was a result of absorption effects from more extended data collections on crystals featuring multiple heavy atoms (U and Te), and these residual electron density peaks could not be addressed using SADABS or via face-indexing. This residual electron density did not affect the overall structures or refinement metrics for **2** and **4**, and we have provided details within the SI and CIFs addressing how data quality limitations were addressed. Crystallographic parameters for **1**–**8** are included in Table S2, and figures for all compounds were generated using the CrystalMaker graphical software package.^78^ CIF files for **1**–**8** have been deposited at the Cambridge Crystallographic Database Centre (CCDC) and can be obtained from https://www.ccdc.cam.ac.uk by citing reference numbers 2528352–2528359. Topological analysis of compounds **1**–**8** was carried out using ToposPro (v5.5.2.2)^79^ employing the standard simplification procedure, in which metal centers were retained as nodes at their crystallographic positions, while organic ligands and coordinated water molecules were reduced to their centers of mass (Figures S71–S78).

#### Powder X-ray Diffraction

Bulk purity of compounds **1**–**6** was assessed using powder X-ray diffraction, wherein 5–10 mg of each sample was ground to a fine polycrystalline powder using an agate mortar and pestle before being placed on KS Analytics zero background holders and analyzed using a Bruker D8 Advanced powder X-ray diffractometer (Cu Kα = 1.54 Å) equipped with a LynxEye solid-state detector. Data were collected over the range 3–60° 2θ with a step size of 0.02° and an exposure time of one second/step with synchronous rotation of the sample holder. PXRD patterns for compounds **1**–**6** were processed via background subtraction, smoothing, Kα2 stripping, and peak selection using PreDICT indexing software^80^ from the International Centre for Diffraction Data (ICDD). Experimental patterns were compared to simulated patterns, based upon single-crystal X-ray diffraction data for **1**–**6**, using the JADE software suite^81^ where simulated patterns were generated using CrystalMaker (Figures S15–S20).^78^ Materials formed after thermogravimetric analysis (TGA) experiments were also analyzed via PXRD and these diffractograms can be found in Figures S21–S30.

#### Vibrational Spectroscopy

Spectroscopic studies involved Raman and attenuated total reflectance infrared (ATR-IR) measurements. Raman spectra were collected on single crystals of compounds **1**–**6** using a Renishaw inVia Raman microscope. Data for each compound were collected using a 514 nm laser at varying power, 20× magnification, and the extended scan setting with a spectral window of 3000–100 cm^–1^. Acquisition methods included ten second exposure times with ten scans per spectrum. Raman spectra were baseline corrected with the PreDICT indexing software from the ICDD. PreDICT software is intended for the analysis of powder X-ray diffraction patterns, yet there is literature supporting its use for processing vibrational spectra as well.^82^ Raman peaks were then fitted with Gaussian or Lorentzian line shapes depending on the characteristics of individual vibrational bands using Origin 2024 software^83^ in the 1600–400 cm^–1^ spectral window. Infrared spectra for compounds **1**–**6** were collected in the mid-IR (4000–400 cm^–1^) and far-IR (400–100 cm^–1^) regions on a Bruker VERTEX 70v instrument using a platinum ATR microscope objective and the OPUS 8.5 software package. Far-IR peaks were fitted with either Voigt or Gaussian line shapes depending on the characteristics of individual vibrational bands. In cases where peak broadening appeared, a Voigt profile was used to account for the convolution of Lorentzian and Gaussian contributions, whereas for more symmetrical and well-isolated peaks, Gaussian functions proved satisfactory. All far-IR baseline corrections and fittings were performed using the Origin 2024 software suite in the 400–100 cm^–1^ region. It was not possible to obtain fits that converged for mid-IR peaks in the 4000–400 cm^–1^ region regardless of function type for any compound. All vibrational spectroscopic data and spectral fittings can be found in Figures S31–S52.

#### Thermogravimetric Analysis

The thermal stability of compounds **1**–**6** was probed using a TA Instruments SDT-Q600 thermogravimetric analyzer. Approximately 5–10 mg of powdered sample for each compound was added to a ceramic pan, capped with a ceramic lid, and heated, from 20 to 850 °C for compounds **1** and **2** and 50–850 °C for compounds **3**–**6**, at a rate of 10 °C/min. Data for each compound were collected under an inert atmosphere (N_2_) at a flow rate of 100 mL/min. For physically mixed samples, a similar sample preparation procedure for TGA analysis was followed, and samples were heated from 50 to 850 °C at a rate of 10 °C/min. Data for the physically mixed samples were then collected separately under N_2_ at a flow rate of 50 mL/min, CO_2_ free air at a flow rate of 100 mL/min, and pure CO_2_ at a flow rate of 20 mL/min. The identity of products after each heat cycle were determined using powder X-ray diffraction, and all TGA data were processed with Origin 2024 (Figures S21–S30 and Figures S53–S60).

#### Scanning Electron Microscopy and Energy Dispersive Spectroscopy

Morphological and semiquantitative compositional analysis of representative samples (compounds **3** and **4**) was performed using a Hitachi 3400N Scanning Electron Microscope (SEM) (Figures S61 and S62). Crystals for each compound were transferred from the mother liquor and adhered to a SEM stub using gold coating. The operating voltage and the emission current were 10.0 kV and 80–120 μA, respectively, and SEM images were collected at magnifications of 1.8k, 3.2k, 8.5k, and 100. The elemental composition of representative samples (compounds **2**–**4**) and selected pre- and post-TGA physical mixing samples were verified using energy dispersive spectroscopy (EDS) with a JEOL JSM-1T700HR field emission scanning electron microscope (FE-SEM). These selected bulk powders were placed on aluminum stubs with double-sided carbon tape and were not coated prior to analysis. The operating voltage was 20 kV and a backscattered electron detector (BED-C) in low-vacuum (40 Pa) mode was used for data collection at a constant working distance of 10 mm (Figures S63–S70).

## Results and Discussion

### Single Crystal X-ray Structural Descriptions

Single crystal X-ray diffraction analysis revealed that compounds **1**–**4** are isostructural and crystallize in the orthorhombic *Pna*2_1_ space group, while **5**–**8** exhibit distinct structures and crystallize in a range of space groups. More specifically, compounds **5** and **8** crystallize in the monoclinic space groups *Cc* and *C*2/*c*, respectively, and compounds **6** and **7** crystallize in the orthorhombic space groups *Pna*2_1_ and *Pca*2_1_. Notably, all eight compounds reported herein are three-dimensional, which is highly unusual for U­(VI) species, with the heavier chalcogenophene-based organic linkers **2,5-SeDC** and **2,5-TeDC** playing a key role in promoting the formation of 3D frameworks.

Compound **1**, [(UO_2_)­(C_6_H_2_O_4_Se)­(C_3_H_7_NO)]_n_, is a representative example of the isostructural compounds **1**–**4**, and thus it will be described in detail. It is a uranyl 3D coordination polymer featuring 2,5-SeDC linkers and coordinated DMF molecules (Figure 1) that adopts a 4,4T150 topology (Figure S71). The single crystallographically unique [UO_2_]^2+^ cation adopts pentagonal bipyramidal geometry with two axial oxygens (O1, O2) bound at distances of 1.785(5) Å and 1.763(5) Å. In the equatorial plane, the uranium metal center coordinates with five oxygen atoms: two oxygens (O3, O4) from a fully deprotonated bidentate carboxylate group of the 2,5-SeDC linker (C_6_H_2_O_4_Se), two additional oxygens (O5, O6) from the second deprotonated carboxylate group of the ligand, and one oxygen from the DMF molecule (O7) that is bound through its carbonyl oxygen atom (Figure 1a). The U–O bond distances in the equatorial plane range from 2.348(4) to 2.447(4) Å, with an average bond distance of 2.386 Å. The local structure of compound **1** features dinuclear uranyl-based secondary building units (SBUs) where [UO_2_]^2+^ moieties are bridged by 2,5-SeDC linkers through their bidentate carboxylate groups, and these SBUs are further linked into a polymeric 3D network through additional ligand coordination (Figure 1b). Selenium (Se1) is embedded in the aromatic selenophene ring and Se bond distances with nearest neighbor carbon atoms are Se1–C2 = 1.869(7) Å and Se1–C5 = 1.887(6) Å. C–C bond distances range from 1.374(8) Å-1.418(8) Å within the 2,5-SeDC ring, and this is consistent with known values observed in analogous thiophene and selenophene structures.^68,84^ Se1–C2–C1 = 123.8(5)° and Se1–C5–C6 = 122.2(4)° bond angles suggest retention of aromatic character, and related torsion angles, O4–C1–C2–Se1 = 7.3(8)° and O6–C6–C5–Se1 = 18.2(8)°, indicate a slight twist of the carboxylate groups of the 2,5-SeDC linker, potentially optimizing alignment so that a three-dimensional coordination polymer can ultimately form. Comparing thiophene- and selenophene-2,5-dicarboxylate frameworks provides useful structural context for ascertaining how carboxylate-group orientations and linker conformations change in chalcogenophene-based U­(VI)-CPs. Prior studies have shown that small changes in heteroatom identity, ligand bend angle, and linker orientation can be propagated into meaningful changes in framework topology and interpenetration behavior.^61,68^ In this context, the modest carboxylate torsion angles observed for compound **1** indicate that the 2,5-SeDC linker remains only slightly twisted from the heteroaromatic ring, allowing it to bridge uranyl SBUs and support formation of the observed three-dimensional coordination polymer. Selected bond distances and angles for compound **1** are provided in Table S3.

**Figure 1:**
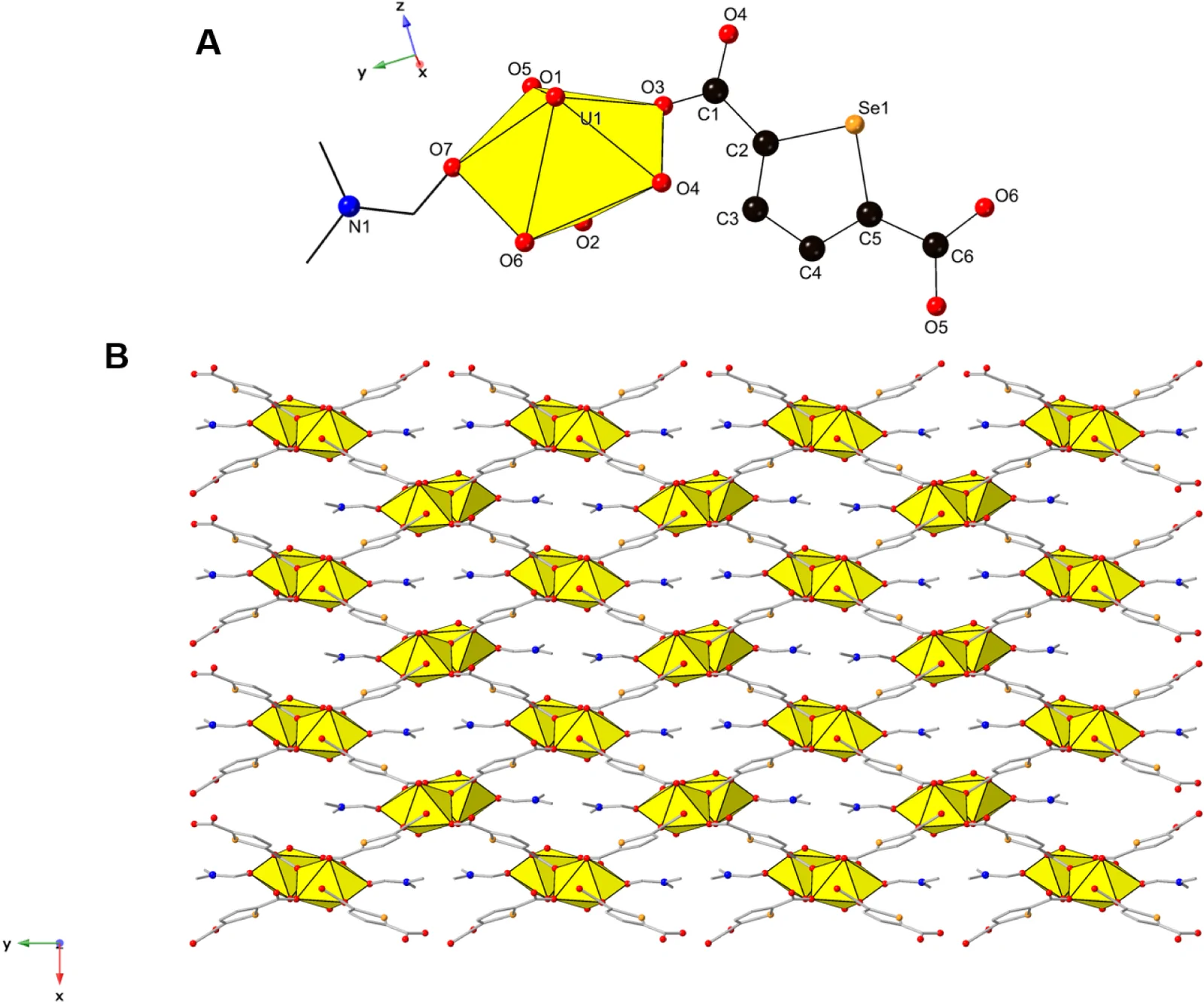
(**a**) Asymmetric unit of [(UO_2_)­(C_6_H_2_O_4_Se)­(C_3_H_7_NO)]_n_ and (**b**) 3D framework structure of compound **1** viewed in the *xy*-plane. U atoms are depicted as yellow polyhedra, C atoms as black wireframes, and N, O, and Se atoms as blue, red, and orange spheres, respectively. All H atoms have been omitted for clarity.

Replacing 2,5-SeDC with 2,5-TeDC in the DMF-containing system yields compound **2**, [(UO_2_)­(C_6_H_2_O_4_Te)­(C_3_H_7_NO)]_n_, which is isomorphous with compound **1** (Figure S7) and adopts the same topology (Figure S72). The overall SBU connectivity and uranyl coordination environment are also comparable to those described for compound **1**. However, notable differences arise within the tellurophene ring structure of 2,5-TeDC (Figure S8). More specifically, the Te–C bond lengths are 2.04(3) Å (Te1–C2) and 2.08(3) Å (Te1–C5), and the Te–C–C bond angles are 121(2)° and 118.7(16)°, respectively. The corresponding torsion angles O3–C1–C2–Te1 = 2(4)° and O5–C6–C5–Te1 = −21(4)° are comparable to those observed for compound **1** within experimental uncertainty. Within the tellurophene ring, carbon atoms C3 and C4 are linked at a bond distance of 1.27(4) Å, which is notably shorter than the analogous bond distance in the 2,5-SeDC ring of **1** (Figure S8). The remaining internal C–C bond distances (C4–C5: 1.36(4) Å and C2–C3: 1.34(4) Å), fall within the range observed for tellurophene rings.^72^ Selected bond distances and angles for compound **2** are provided in Table S4.

Substitution of DMF with DMSO in the 2,5-SeDC system affords compound **3**, [(UO_2_)­(C_6_H_2_O_4_Se)­(C_2_H_6_OS)]_n_, which exhibits identical packing (Figure S9) and the same topology as compound **1** (Figure S73). Structural metrics associated with the 2,5-SeDC ring remain largely unchanged, with Se–C bond lengths of 1.868(6) Å (Se1–C2) and 1.881(6) Å (Se1–C5) and bond angles (Se1–C2–C1 = 123.1(6)°, Se1–C5–C6 = 122.7(4)°), indicating conserved aromatic character. Torsion angles are O3–C1–C2–Se1 = 179.9(4)° and O6–C6–C5–Se1 = 10.4(7)°, and compound **3** also exhibits a slightly shorter average equatorial U–O_eq_ bond distance (2.378(4) Å vs 2.386(4) Å) compared to **1**, accompanied by a marginal reduction in unit cell volume (1286.8(10) Å^3^ vs 1279.81(9) Å^3^). Selected bond distances and angles for compound **3** are provided in Table S5. Switching from 2,5-SeDC to 2,5-TeDC using the DMSO solvent system results in compound **4** (Figure S10), [(UO_2_)­(C_6_H_2_O_4_Te)­(C_2_H_6_OS)]_n_, which also adopts the same topology as compounds **1**–**3** (Figure S74). The uranyl center retains pentagonal bipyramidal geometry, and detailed structural metrics are compiled in Table S6. In compound **4**, the 2,5-TeDC linker exhibits Te–C bond distances of 2.04(2) Å (Te1–C2) and 2.07(2) Å (Te1–C5), which are comparable to those observed in compound **2**. The remaining internal C–C bond distances (C4–C5: 1.37(3) Å and C2–C3: 1.37(3) Å) also fall within the expected range for tellurophene rings.^72^ Moreover, the bond distance for C3–C4 (1.39(3) Å) is shorter than the corresponding value observed in compound **3** (Figure S11). The associated torsion angles (O4–C1–C2–Te1 = −165.7(13)° and O5–C6–C5–Te1 = 23(2)°) indicate moderate twisting of the tellurophene moiety within the CP. Relative to compound **2**, replacement of DMF with DMSO in **4** results in a slightly longer average equatorial U–O bond distance (2.375 Å vs 2.365 Å) (Tables S4 and **S6**), while the underlying SBU connectivity and three-dimensional framework architecture are preserved.

To further evaluate framework-level characteristics across compounds **1**–**8**, pore metrics were assessed using Mercury Pore Analysis (Table S1). Compounds **1**–**4** exhibit small, geometrically constrained void spaces, with limited pore volumes and almost no percolated dimensions, consistent with dense three-dimensional packing of these uranyl–chalcogenophenedicarboxylate networks. Among the series, compound **3** exhibits the largest maximum pore diameter of 2.44 Å, whereas compound **1** displays the smallest calculated void volume; however, these differences remain modest and do not alter overall pore accessibility. The calculated pore limiting diameters for compounds **1–4** range from 0.71 to 1.22 Å, and maximum pore diameters range from 1.99 to 2.44 Å for each compound, indicating that substitution of 2,5-SeDC with 2,5-TeDC or DMF with DMSO does not introduce extended, accessible channels within compounds **1**–**4**. In all cases, void spaces are largely internal and not expected to support guest diffusion, in agreement with the absence of network-accessible surface area. These results confirm that while **1**–**4** adopt three-dimensional framework topologies, their porosity is structurally constrained rather than functionally open, and variations in linker identity and coordinated solvent primarily influence local coordination metrics rather than overall pore architectures.

Compound **5**, [(UO_2_)_2_(C_6_H_2_O_4_Se)_2_]_n_·2H_2_O, crystallizes in the *Cc* space group, and displays a 3D CP structure featuring U­(VI)–U­(VI) cation–cation interactions (CCIs) (Figure 2). Unlike compounds **1** and **3**, which feature the same linker and include coordinating solvents such as DMF and DMSO, compound **5** was synthesized hydrothermally, and the resulting structure lacks solvent coordination in the uranyl equatorial planes. Both uranyl centers in compound **5** adopt pentagonal bipyramidal geometries, with two axial oxo groups (U1–O1 = 1.809(14) Å, U1–O2 = 1.749(14) Å; U2–O3 = 1.769(13) Å, U2–O4 = 1.748(14) Å) and five equatorial oxygen atoms (Figure 2a). For U2, equatorial binding occurs through a combination of carboxylate group coordination, from 2,5-SeDC ligands, and a CCI, where the oxo group of one uranyl ion (U1) coordinates to the equatorial plane of an adjacent uranyl center (e.g., U2–O1 = 2.478(13) Å). CCIs, also referred to as actinyl–actinyl interactions (AAIs), are rare within uranyl­(VI) chemistry,^1,85–88^ and here these interactions enable additional connectivity within the CP (Figure 2b). The bond distances and angles in the uranyl equatorial plane reveal a slight distortion, characteristic of structures with U­(VI) cation–cation interactions, specifically, the U–O distances to the carboxylate oxygen atoms range from 2.322(13) Å to 2.426(14) Å, while the U–O bond involving the cation–cation interaction is slightly longer at 2.478(13) Å. The selenium atoms embedded in the selenophene rings of compound **5** exhibit Se–C bond distances of 1.878(19) Å (Se1–C2), 1.837(18) Å (Se1–C5), 1.841(18) Å (Se2–C8), and 1.846(18) Å (Se2–C11), with C–Se–C bond angles of 86.8(8)° and 85.8(8)°, respectively. These values are slightly lower than those observed in compounds **1** and **3**, likely reflecting packing effects and the absence of coordinated solvent. Torsion angles are C5–Se1–C2–C3 = −0.4(15)°, C5–Se1–C2–C1 = −178.4(15)°, and C2–Se1–C5–C6 = 176.2(15)°, which indicate nearly planar selenophene rings, in contrast to the more twisted geometries seen in **1** and **3**, suggesting reduced heterocycle distortion. Compared to compounds **1** and **3**, where DMF and DMSO solvent molecules are incorporated into the first coordination sphere, the SBUs in compound **5** are distinct, as they are constructed solely through bridging 2,5-SeDC ligands and cation–cation interactions (Figure 2b), and they highlight an alternative SBU motif within the same ligand system. In this CP, the presence of U­(VI) CCIs led to a previously unobserved 3,4²,5-c net topology (Figure S75), which differs from the topologies observed for compounds **1** and **3**. Selected bond distances and angles for **5** are provided in Table S7.

**Figure 2:**
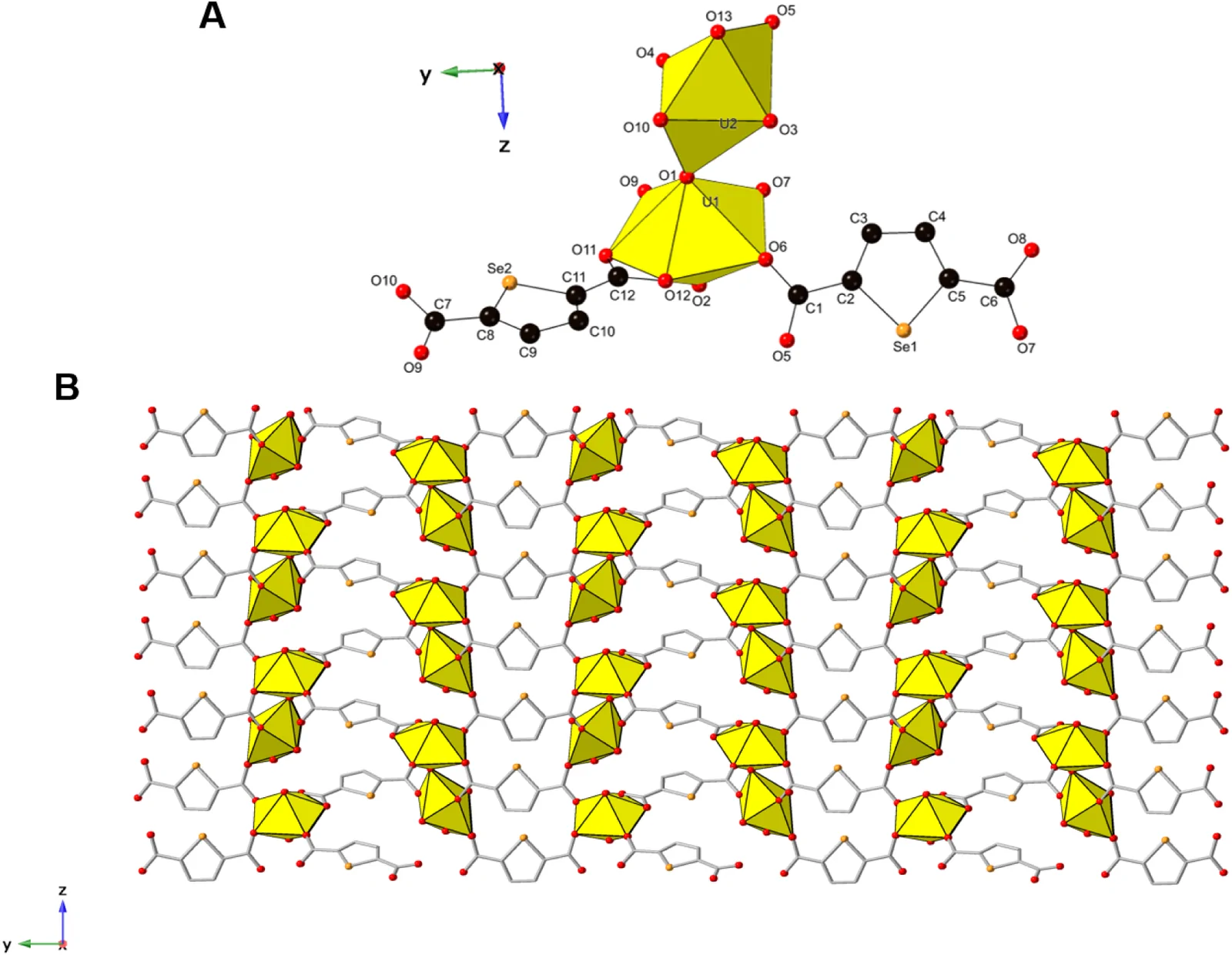
(**a**) Asymmetric unit of [(UO_2_)_2_(C_6_H_2_O_4_Se)_2_]_n_·2H_2_O and (**b**) three-dimensional CP structure of compound **5** viewed in the *yz*-plane. All H atoms have been omitted for clarity.

Hydrothermal synthesis with 2,5-TeDC yielded compound **6**, [(UO_2_)­(C_6_H_2_O_4_Te)]_n_·H_2_O, which crystallizes in the space group *Pna*2_1_ and features bidentate bridging 2,5-TeDC ligands linking chains of uranyl centers into a three-dimensional framework (Figure 3). The crystallographically unique uranyl cation in **6** adopts distorted pentagonal bipyramidal geometry, with axial U=O bond lengths of 1.793(15) Å and 1.766(16) Å and equatorial U–O distances ranging from 2.332(19) to 2.404(10) Å with an average distance of 2.368 Å (Figure 3a). All equatorial positions around the [UO_2_]^2+^ cation are occupied by carboxylate oxygen atoms from 2,5-TeDC ligands, and no solvent molecules coordinate to the uranium centers. This solvent free coordination sphere contrasts with the DMF- and DMSO-bound structures observed in compounds **2** and **4,** and compound **6** adopts a different 4,4T3 topology (Figure S76). The tellurophene ring exhibits Te–C bond lengths of 2.08(3) Å (Te1–C2) and 2.05(3) Å (Te1–C5), consistent with the elongation observed in compounds **2** and **4**. Moreover, the internal C–C distances in compound **6** range from 1.36 to 1.46 Å, which are comparable to the values observed for compounds **2** and **4**, and consistent with the broader tellurophene-based structures.^89,90^ The Te–C–C bond angle of 80.6(8)° and torsion angle of −179(3)° (C1–C2–C3–C4) suggest moderate ring strain and slight twisting of the ligand backbone, yet the aromatic character of the 2,5-TeDC ring is retained. Unlike compound **5**, which features CCIs and a more open framework with a larger maximum pore diameter (2.63 Å vs 1.78 Å), compound **6** does not exhibit CCIs or π–π interactions between the chalcogenophene rings. Instead, its 3D connectivity arises solely from ligand bridging, which highlights the influence of the chalcogen atom within heteroaromatic rings on resulting structures and network topologies in uranyl-based MOFs. Selected bond distances and angles for **6** are provided in Table S8.

**Figure 3:**
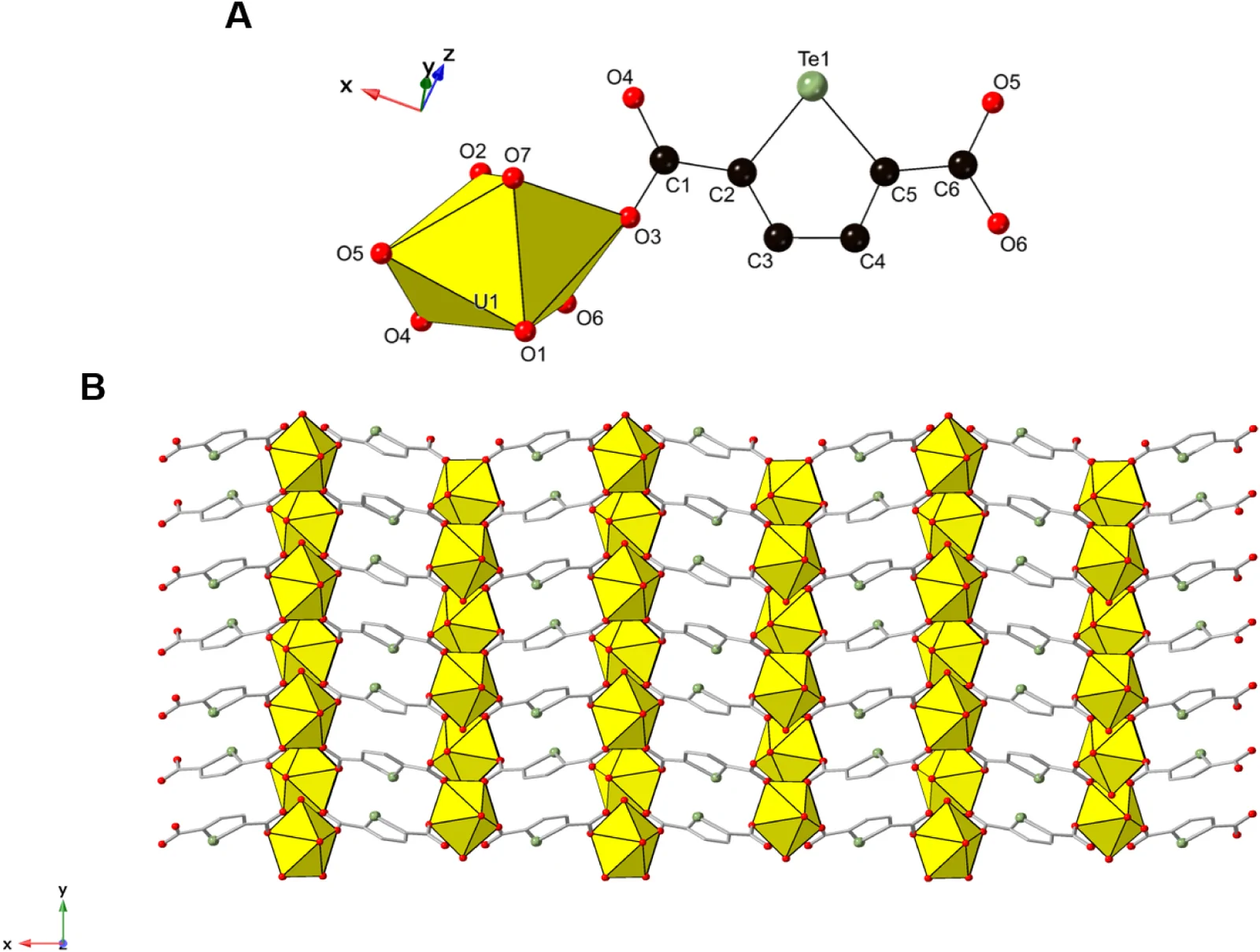
(**a**) Asymmetric unit of [(UO_2_)­(C_6_H_2_O_4_Te)]_n_·(H_2_O) and (**b**) three-dimensional CP structure of compound **6** viewed in the *xy*-plane. All H atoms have been omitted for clarity.

Consistent with the framework-level analysis discussed for compounds **1**–**4**, Mercury Pore Analysis was also carried out for compounds **5** and **6** to assess the effect of topology and reaction conditions on void space and pore accessibility (Table S1). In contrast to the solvent-coordinated CPs **1**–**4**, compound **5** exhibits pore space that is largely confined to small, poorly connected cavities rather than extended channels. The packing associated with the 3,4^2^,5-c net topology results in a substantially increased pore volume relative to compounds **1**–**4**. Interestingly, compound **5** exhibits the most open structure among all compounds in this study, featuring two-dimensional ultramicroporous channels with a pore limiting diameter of 1.31 Å and a maximum pore diameter of 2.63 Å, yet it appears to possess limited porosity that may restrict guest diffusion. Mercury Pore Analysis on compound **6** revealed the three-dimensional network in this structure is defined by pore spaces with limited accessibility (Table S1). The calculated void space is small for **6** and dominated by isolated, narrow cavities, with a pore limiting diameter of 0.89 Å and a maximum pore diameter of 1.78 Å, with no continuous pore channels identified throughout the structure. These features are consistent with the dense packing associated with the CP topology of compound **6**. Compared to the solvent-containing coordination polymers **1**–**4**, compound **6** further exhibits a reduction in void volume and pore accessibility, reinforcing the impact of structural topologies on resulting CP and MOF architectures.

#### Structural Discussion

Compounds **1**–**8** were all synthesized with chalcogenophene-based dicarboxylic acid ligands featuring either Se or Te; thus, the resulting structures provide an opportunity to explore how differences in chalcogen characteristics and reaction conditions impact material dimensionality, porosity, and structural topologies within uranyl hybrid materials. Comparing the dimensionality and means of assembly observed in compounds **1**–**8** (Table 1), and the electronic and structural manifestations thereof, we can make several comments about the influence of solvent systems and metal-to-ligand ratios. The choice of solvent (DMF–H_2_O, DMSO–H_2_O, or only H_2_O) and variations in stoichiometric ratios impact the local coordination environment around the uranyl cations, yet do not impact the dimensionality of the resulting frameworks when 2,5-SeDC or 2,5-TeDC are used as linkers. Compounds **1**–**4**, assembled in DMF–H_2_O or DMSO–H_2_O solvent systems, form 3D CPs^91^ with direct coordination of cosolvent molecules (DMF or DMSO) to the uranyl centers. The change from DMF to DMSO in compounds **3** and **4** does not alter the framework dimensionality or SBU connectivity, as both solvent systems yield topologically identical three-dimensional structures. Compound **5**, prepared solely in water with 2,5-SeDC, forms a distinct three-dimensional framework enabled by uranyl CCIs, whereas compound **6**, the 2,5-TeDC analog of **5**, adopts a different three-dimensional structure that lacks CCIs. Three-dimensional structures are consistently observed across **1**–**6** and this unusual uranyl assembly behavior is driven by a synergistic combination of geometric and steric characteristics of the 2,5-SeDC and 2,5-TeDC linkers. Compounds **7** and **8**, minor products that were initially cosynthesized with **1** and **2**, could be isolated in pure form using higher metal-to-ligand ratios (1:2), and these structures feature no coordinated solvent in uranyl first coordination spheres. Instead, these frameworks incorporate *N*,*N*-dimethylacetamide (DMA^+^) as interstitial, charge-balancing species arising from DMF hydrolysis to charge balance the anionic uranyl frameworks. Compound **7** adopts a hcb topology (Figure S77), while compound **8** exhibits a 2C1 topology (Figure S78). In both structures, the frameworks are constructed from mononuclear uranyl SBUs bridged exclusively by carboxylate ligands. Despite this shared SBU nuclearity, the two compounds differ in ligand conformation, framework packing, and pore architecture, leading to distinct topologies and void characteristics, and both minor products possess greater internal void space relative to compounds **1**–**6** (Table S1).

**Table 1:** Structural Summary for Compounds **1**–**8** under Varying Reaction Conditions^a^

Compound	Chalcogenophene-based ligand	Metal-to-ligand ratio	Dimensionality	Solvent system	UO_2_-UO_2_ cation–cation interaction	Uranyl SBU
**1**	2,5-SeDC	1:1	3D CP	DMF-H_2_O	No	Dimer
**2**	2,5-TeDC	1:1	3D CP	DMF-H_2_O	No	Dimer
**3**	2,5-SeDC	1:1	3D CP	DMSO-H_2_O	No	Dimer
**4**	2,5-TeDC	1:1	3D CP	DMSO-H_2_O	No	Dimer
**5**	2,5-SeDC	1:1	3D CP	H_2_O	Yes	Monomer
**6**	2,5-TeDC	1:1	3D CP	H_2_O	No	Monomer
**7**	2,5-SeDC	1:2	3D MOF	DMF-H_2_O	No	Monomer
**8**	2,5-TeDC	1:2	3D MOF	DMF-H_2_O	No	Monomer

a Full compound formulae for 1-8 are provided in Table S2.

Control experiments conducted under reaction conditions matching those included in the experimental section and Table 1 with the structurally analogous linkers 2,5-FDC and 2,5-TDC further clarify the critical role of linker identity in dictating framework dimensionality. The solvothermal reaction of U­(VI) and 2,5-FDC in DMF yielded a new two-dimensional anionic framework with a honeycomb-structure that lacks coordinated solvent (Figure S14 and Tables S11–S12), while a similar reaction with 2,5-TDC afforded a known two-dimensional uranyl coordination polymer.^65^ Neither of these control reactions resulted in uranyl 3D frameworks, indicating that incorporation of heavier chalcogen atoms in organic linkers plays a critical role in promoting higher-dimensional connectivity. 2,5-SeDC and 2,5-TeDC combine increased electronic polarizability and smaller HOMO–LUMO gaps, in contrast to 2,5-FDC and 2,5-TDC, reflecting greater electronic softness and conformational flexibility,^84^ and these electronic characteristics, as manifested in the observed ring distortions and packing differences, are a likely driver of the three-dimensional uranyl architectures observed herein. Known examples of three-dimensional uranyl frameworks have often been accessed using rigid, multitopic O-donor linkers, which are most commonly aromatic dicarboxylates that can bridge uranyl centers through multiple coordination modes and support higher network connectivity.^9,10,92^ Moreover, coordination through the carboxylate groups extends connectivity beyond the equatorial plane of the uranyl cation center, enabling formation of three-dimensional frameworks.^41^ In this regard, 2,5-SeDC and 2,5-TeDC share key design features with established uranyl-MOF linkers, namely a rigid aromatic backbone and ditopic carboxylate functionality capable of connecting multiple uranyl nodes to generate extended architectures. A distinguishing aspect of our system is replacement of lighter heteroatoms with heavier chalcogens within the same heteroaromatic scaffold, which introduces increased polarizability and electronic softness that manifest as differences in framework assembly and packing across the series.

#### Thermogravimetric Analysis

One of the key findings of this study is revealed through thermogravimetric analysis, which was conducted on the 2,5-SeDC-containing compounds **1**, **3**, and **5** as well as the 2,5-TeDC-containing compounds **2**, **4**, and **6** to evaluate their thermal stability and decomposition behavior. The decomposition profiles for each compound were further analyzed to determine distinct degradation steps and results are presented in Figures S55–S60. For the 2,5-SeDC-containing compounds, the linker itself exhibits stability up to 270 °C, providing a baseline for evaluating the decomposition pathways of the corresponding uranyl compounds (Figure S53). Compound **1** undergoes an initial weight loss of approximately 14% below 220 °C, corresponding to solvent removal in two steps, followed by significant decomposition events between 220–800 °C, with a total weight loss of roughly 40% that is consistent with the theoretical expectation (39%) for 2,5-SeDC degradation. The total remaining weight is ca. 46% of the original sample mass, which is slightly lower than the expected weight (48%), and based on PXRD analysis is primarily composed of a reduced uraninite-type (UO_2_/UO_2+x_) phase (Figure S22). Compound **3** follows a similar trend with a small initial weight loss occurring at 130 °C that could be associated with loss of adventitious water followed by solvent loss occurring in two steps between 230 and 340 °C. This weight loss of approximately 13% is in good agreement with the theoretical expectation (14%) and is followed by a major weight loss event spanning 340–800 °C wherein 2,5-SeDC breaks down and a reduced uraninite-type (UO_2_/UO_2+x_) phase forms, as confirmed by PXRD (Figure 4a). Compound **5** decomposes in a slightly different manner, with solvent evaporation occurring below 180 °C corresponding to an approximate 3.5% weight loss that is in good agreement with the theoretical calculation (3.5%). Two major decomposition events follow between 380–800 °C, amounting to a weight loss of 26%, which is lower than the expected value (43%) suggesting incomplete framework degradation. The total remaining weight is approximately 69% of the original mass, which is higher than the expected value (53%), and the final residue is also a reduced uraninite-type (UO_2_/UO_2+x_) phase according to PXRD analysis (Figure 4b).

**Figure 4:**
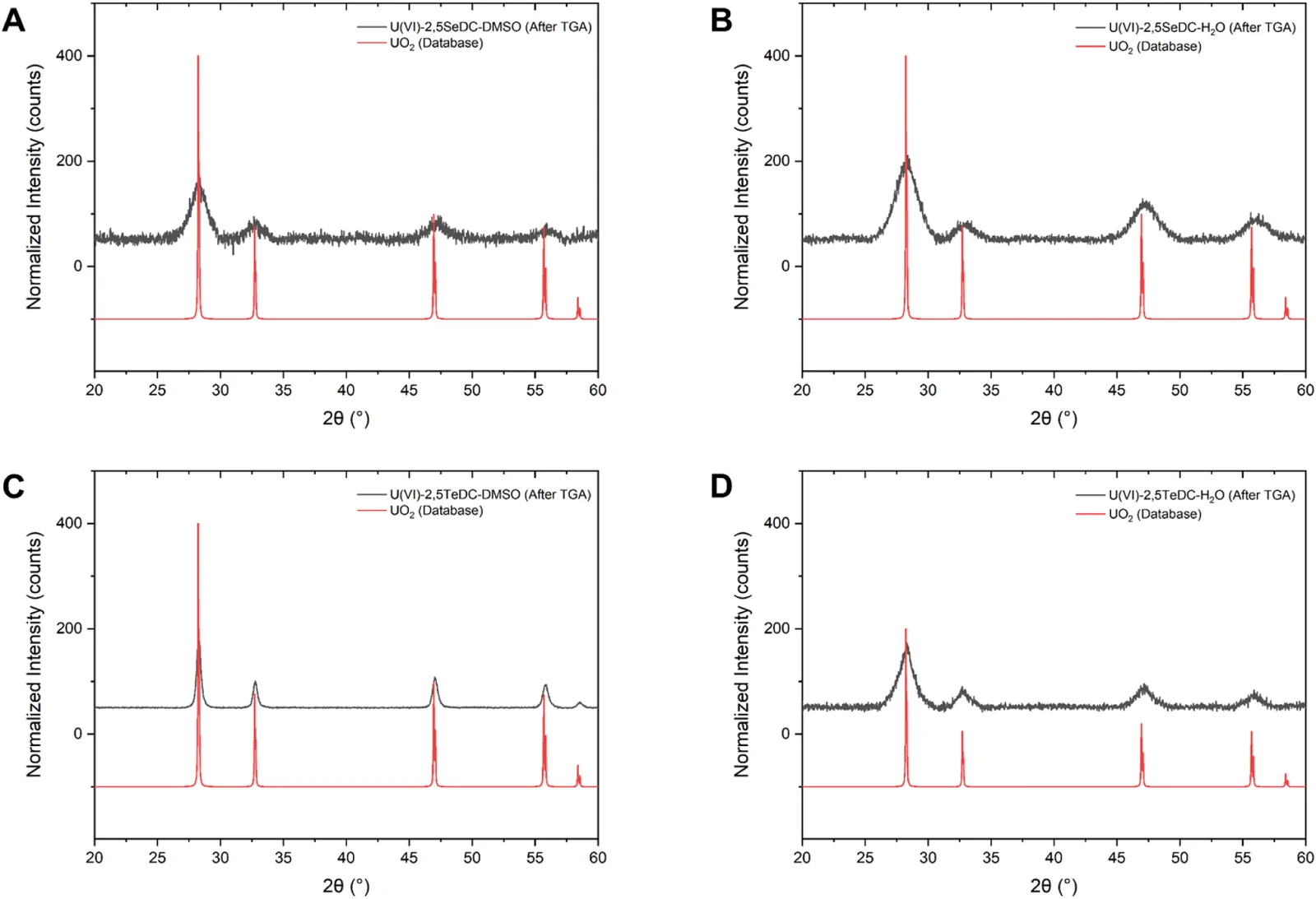
Post-TGA PXRD patterns of representative uranyl frameworks compared with the reference pattern of UO_2_ (PDF card no: 01–071–6416). (**a**) Compound **3** after TGA, (**b**) compound **5** after TGA, (**c**) compound **4** after TGA, and (**d**) compound **6** after TGA. Experimental PXRD data are shown in black and reference patterns are shown in red. All samples exhibit diffraction features consistent with formation of a UO_2_/UO_2+x_ phase following thermal treatment.

Switching to 2,5-TeDC, a similar ligand decomposition pattern is observed when only the linker is evaluated via TGA, which shows that 2,5-TeDC stays intact up to 260 °C (Figure S54). Compound **2** undergoes a total initial weight loss of approximately 13% below 200 °C associated with the solvent removal that is consistent with the expected theoretical weight loss (12%). This is followed by decomposition events between 200–800 °C that result in a total weight loss of 51%, which is greater than the calculated theoretical mass loss for the 2,5-TeDC ligand removal alone (43%), suggesting the possibility of continued material volatilization at elevated temperatures. The residual mass is approximately 36% of the initial sample mass and PXRD analysis of the post-TGA residue confirms the formation of a reduced uraninite-type (UO_2_/UO_2+x_) phase (Figure S23). Compound **4** shows solvent loss below 220 °C that corresponds to a 12.5% weight loss, in good agreement with the theoretical value (13%). Subsequently, major decomposition events occur in several steps between 220–670 °C, exhibiting a total mass loss of 42% that aligns with theoretical calculations for the ligand breakdown (43%), with the remaining residue identified as a reduced uraninite-type (UO_2_/UO_2+x_) phase by PXRD (Figure 4c). Compound **6** features an initial dehydration step below 250 °C that results in a 3% weight loss that aligns well with theoretical expectations (3%), followed by three major decomposition events in the range of 250–700 °C. The total weight % loss during the three decomposition events is approximately 50%, which is in reasonable agreement with the expectations for 2,5-TeDC breakdown (48%). The remaining weight is ca. 46% of the initial residue and was identified as a reduced uraninite-type (UO_2_/UO_2+x_) phase by PXRD (Figure 4d). The overall thermal stability of these uranyl compounds, with decomposition temperatures exceeding 325 °C for **3** and **5** is indicative of notable framework structural integrity relative to conventional organic-based materials. Additionally, the thermal stability of these compounds is comparable to that of most CPs and MOFs which also typically decompose below 400 °C.^93–96^ Despite subtle differences in TGA profiles across 2,5-SeDC and 2,5-TeDC systems, the final residues indicate the formation of a reduced (UO_2_/UO_2+x_) phase that can be identified as the stable end product for compounds **1**–**6** after heating (Figures 4 and S22–S23). Since post-TGA PXRD patterns were collected without an internal standard,^97,98^ the exact oxygen stoichiometry of the reduced uranium oxide residues cannot be definitively assigned from PXRD alone; therefore, these products are best described as UO_2_/UO_2+x_ phases.

The redox transformation observed in TGA final products is worthy of further discussion as it reflects a reduction of U­(VI) to U­(IV). Typically, the heating of U­(VI) hybrid materials results in decomposition to U­(VI) oxides.^59,99,100^ While reduction of U­(VI) to U­(IV) under thermal or hydrothermal conditions has been reported in a few cases,^101,102^ these transformations are generally governed by either solution-phase chemistry or thermolysis of molecular precursors in reactive organic media. In contrast, in our system the organic components are incorporated as framework linkers, and UO_2_ formation is dependent on the incorporation of heavier chalcogen atoms within organic ligands. More explicitly, materials containing 2,5-SeDC and 2,5-TeDC undergo reduction to a UO_2_/UO_2+x_ phase, whereas analogous frameworks containing 2,5-TDC and 2,5-FDC do not under comparable conditions. This distinction indicates that, although thermal decomposition occurs in the systems reported by Hudry and colleagues^86^ and Benarib et al.,^87^ reduction in our study is not simply a general consequence of framework decomposition, rather it is linked to the thermolysis of the heavier chalcogenophene-based ligands. Instead, results herein indicate reduction is a result of a unique ligand-dependent solid-state process based on results in mixed-ligand systems and physical mixing experiments (*vide infra*). As evidenced by the PXRD patterns of **1**–**6** collected after heating (Figures 4 and S22–S23), we do not observe U­(VI) oxide formation after framework degradation, rather U­(IV) is formed without the need for high pressures or addition of chemical reductants.^103,104^ The spontaneous reduction likely arises from thermally induced decomposition of the heavier chalcogenophene-based ligands used herein,^84^ which may generate reductive intermediates through C–H, C–C, or C–Te bond cleavage and bond-forming pathways during thermolysis. Additionally, the conjugated, electron-rich nature of the selenophene and tellurophene rings can create localized environments that promote electron delocalization, facilitating thermal decomposition of the ligands.^72,105^ The consistent formation of UO_2_/UO_2+*x*_ across these varied systems points to a common mechanistic feature tied to the decomposition of the chalcogenophene-based ligands themselves, and our hypothesis is that the reduction of U­(VI) to U­(IV) observed during the thermal decomposition of selenophene- and tellurophene-based uranyl compounds likely arises from the formation of thermally generated organochalcogen species rather than from the *in situ* oxidation of Se or Te to their oxide forms (Scheme S3). Organochalcogen compounds are known to be thermally labile, and prior studies have shown that C–Se and C–Te bonds can undergo homolytic or heterolytic cleavage upon heating to generate reactive, low-valent chalcogen-containing fragments.^106,107^ Upon heating under inert atmosphere, the cleavage of C–Se and C–Te bonds likely produces low-valent intermediates such as R–SeH or R–TeH, which are capable of donating electrons to nearby uranyl centers. Consistent with this hypothesis, the uranyl ion is known to undergo reduction to U­(IV) in the presence of sufficiently reducing species.^108^ This redox process could then facilitate the reduction of U­(VI) to U­(IV), yielding nanocrystalline uraninite as observed in this study. Post-TGA PXRD patterns (Figure 4) show pronounced peak broadening relative to bulk UO_2_ references, consistent with formation of nanocrystalline uraninite,^109^ and although internal oxygen sources such as carboxylate decomposition products or coordinated solvent molecules could in principle enable further oxidation of tellurium, PXRD analysis after TGA data collection does not reveal peaks characteristic of crystalline SeO_2_ or TeO_2_ in final residues. Similar behavior has been reported in systems where chalcogen-containing species undergo thermal decomposition to noncrystalline or elemental products rather than well-defined oxides.^110,111^ This suggests that the chalcogen species responsible for uranium reduction either remains in a low-valent or elemental form, evaporates during heating, or are converted into amorphous products that are not detectable by PXRD. In addition, the lack of evidence for crystalline TeO_2_ formation further supports the likelihood that transient, volatile low-valent organochalcogen intermediates are the primary electron donors in the U­(VI) to U­(IV) redox transformation observed for compounds **2**, **4**, and **6**.

U­(VI) to U­(IV) redox transformations are also specific to 2,5-SeDC and 2,5-TeDC systems and do not occur upon heating when analogous uranyl hybrid materials are prepared with 2,5-FDC or 2,5-TDC since furan and thiophene moieties lack redox-active chalcogen centers and produce decomposition byproducts that are not sufficiently reducing. Interestingly, physical mixtures of solid 2,5-TeDC and uranyl-2,5-TDC (U­(VI)–2,5-TDC) or uranyl-2,5-FDC (U­(VI)–2,5-FDC) in a 1:1 stoichiometric ratio can also lead to the formation of UO_2_/UO_2+*x*_ under inert atmosphere, as confirmed by PXRD (Figures 5 and S26, S29, and S30). To further probe the role of the chalcogenophene linker in the observed redox transformation, a series of physical mixing control experiments were conducted using U­(VI)–2,5-FDC and U­(VI)–2,5-TDC compounds with varying amounts of 2,5-TeDC under different atmospheres. When U­(VI)–2,5-FDC was physically mixed with a stoichiometric 1:1 amount of 2,5-TeDC and heated under N_2_, PXRD revealed the formation of a reduced uraninite-type (UO_2_/UO_2+x_) phase (Figure 5a), confirming that 2,5-TeDC can directly promote U­(VI) to U­(IV) reduction. In contrast, a substoichiometric amount of 2,5-TeDC (0.1 equiv) under the same conditions resulted in a mixture of U_3_O_8_ and UTeO_4_ phases (Figure 5b), indicating partial uranium reduction but not complete conversion to U­(IV). This observation suggests that addition of substoichiometric quantities of 2,5-TeDC may allow for accessing incomplete reduction products or intermediates along the reduction pathway, whereas stoichiometric quantities of the linker are necessary to facilitate quantitative U­(VI) to U­(IV) reduction. A similar 1:1 physical mixture of U­(VI)–2,5-FDC with 2,5-SeDC yielded only U_3_O_8_, highlighting that the lighter Se analog is less effective at promoting U­(VI) to U­(IV) reduction under the same conditions during physical mixing (Figure S24). When the 2,5-TeDC–U­(VI)–2,5-FDC mixture was heated under CO_2_ free-air or CO_2_, PXRD patterns revealed that only U_3_O_8_ and tellurium-containing oxide phases (UTeO_4_ or UO_2_TeO_3_) formed (Figures 5c and 5d), which indicated the heating environment is integral to the U­(VI) to U­(IV) redox process as well. The analogous heating of U­(VI)–2,5-TDC physically mixed with 2,5-TeDC under CO_2_ free-air or CO_2_ atmospheres also led to U_3_O_8_ and Te-based oxide phase mixtures (Figures S27 and S28). Moreover, mixing elemental Te^0^ with U­(VI)–2,5-FDC (1:1) yielded U_3_O_8_ and UO_2_TeO_3_ phases (Table S13 and Figure S25), which indicated that Te^0^ alone cannot induce the same U­(VI) to U­(IV) transformation as the intact 2,5-TeDC ligand. Similarly, U­(VI)–2,5-TDC physically mixed with 2,5-TeDC and heated under N_2_ produced a reduced uraninite-type (UO_2_/UO_2+x_) phase at both 600 °C and 700 °C (Table S13 and Figures S29 and S30), confirming that the redox process does not require temperatures of 800 °C and above. Taken together, these findings underscore that the U­(VI) to U­(IV) reduction is both ligand and atmosphere-dependent, in addition to being a stoichiometric process, and that 2,5-TeDC uniquely enables U­(VI) to U­(IV) reduction even in mixed-ligand systems (Table S13). The formation of a UO_2_/UO_2+x_ phase following heating of physical mixtures containing 2,5-TeDC and U­(VI)–FDC or U­(VI)–2,5-TDC compounds also suggests that decomposition of 2,5-TeDC produces reducing species capable of facilitating U­(VI) to U­(IV) reduction, even in the absence of direct coordination to uranyl centers. This observation points to a thermally induced, chalcogen-mediated redox process that involves direct solid-state contact and/or volatile Te-containing decomposition products generated during heating, likely driven by the thermal lability and inherent redox activity of the tellurophene moiety. The ability of 2,5-TeDC to induce U­(VI) reduction in physically mixed systems underscores the distinct chemical reactivity of heavier chalcogenophene-based linkers, which can likely generate a locally reducing environment upon thermal decomposition.^112,113^ The lack of U­(VI) reduction upon physical mixing with elemental Te^0^ further supports the conclusion that the active reducing species are transient intermediates accessible only during the thermal breakdown of the Te-containing 2,5-TeDC linker. This behavior again stands in contrast to the lighter chalcogenophene analogues, such as furan and thiophene and to some extent selenophene, which do not exhibit similar redox activity under comparable conditions.

**Figure 5:**
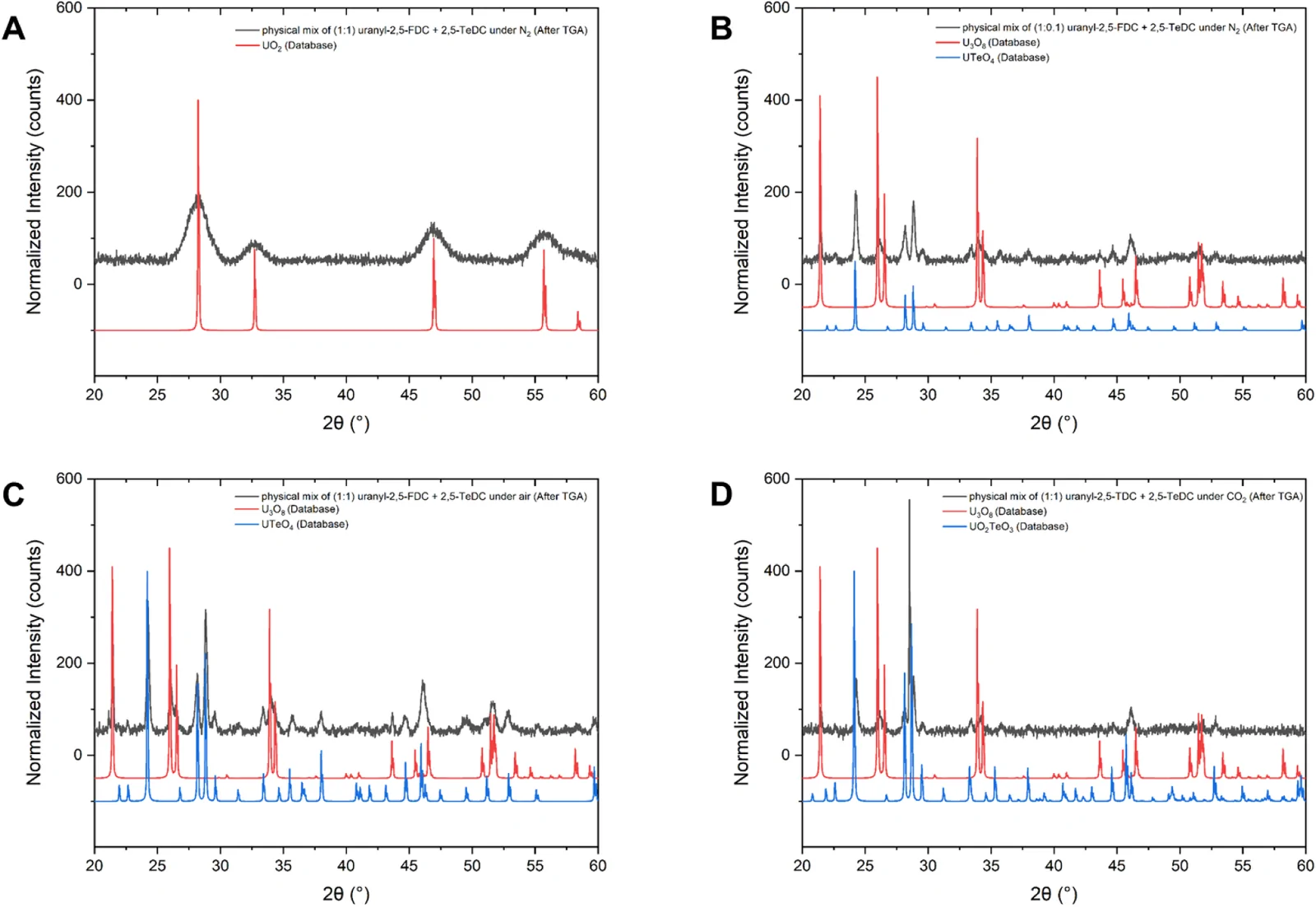
Post-TGA PXRD patterns of representative physical mixtures collected under different conditions and compared with reference oxide phases. Experimental PXRD data are shown in black with reference patterns shown in red and blue and overlaid for comparison. (**a**) Physical mixture of (1:1) U­(VI)–2,5-FDC and 2,5-TeDC heated under N_2_ compared with UO_2_ (PDF card no. 01–071–6416). (**b**) Physical mixture of (1:0.1) U­(VI)–2,5-FDC and 2,5-TeDC heated under N_2_ compared with U_3_O_8_ (PDF card no. 04–007–1246) and UTeO_4_ (PDF card no. 00–055–0562). (**c**) Physical mixture of (1:1) U­(VI)–2,5-FDC and 2,5-TeDC heated under air compared with U_3_O_8_ (PDF card no. 04–007–1246) and UTeO_4_ (PDF card no. 00–055–0562). (**d**) Physical mixture of (1:1) U­(VI)–2,5-TDC and 2,5-TeDC heated under CO_2_, compared with U_3_O_8_ (PDF card no. 04–007–1246) and UO_2_TeO_3_ (PDF card no. 04–015–5352).

#### Scanning Electron Microscopy-Energy Dispersive Spectroscopy

SEM-EDS analysis was conducted on compounds **3** and **4**, which were selected as representative samples, to examine differences in composition, morphology, particle size, and surface texture between selenophene- and tellurophene-containing uranyl frameworks within this series. SEM images were collected at multiple magnifications (1.8k, 3.2k, 8.5k, and 100) to provide a detailed comparison of structural and morphological variations (Figures S61 and S62). At the lowest magnification (100), both compounds exhibit agglomerated heterogeneous morphologies, yet noticeable differences are observed in their overall aggregation behavior. Compound **3** forms more dense and compact clusters, whereas compound **4** displays a more porous and loosely packed morphology. At the highest 8.5k magnification, surface textures become increasingly distinct. The 2,5-SeDC containing **3** maintains its sharp-edged, compact crystalline morphology, while **4** displays a highly porous, fragmented surface with plate-like structures. At lower magnifications, 1.8k and 3.2k, additional distinct morphological features emerge; however, for both, grains^114^ are roughly about five to ten microns. Compound **3** is composed of well-defined, angular crystallites with layered surface features, indicative of an ordered growth pattern. In contrast, compound **4** exhibits elongated, rod-like structures with highly fractured and disordered textures. These differences may arise from variations in metal-linker interactions, where the increased polarizability of 2,5-TeDC compared to 2,5-SeDC could influence the nucleation sites and growth dynamics of the resulting crystals.

Subsequently, EDS analysis was also performed on compounds **2**, **3**, and **4** both before and after TGA analysis to evaluate elemental composition changes associated with thermal decomposition (Figures S63–S68). Prior to heating, EDS spectra and elemental maps confirm the presence of uranium, oxygen, carbon, and the expected chalcogen element (Se for **3** and Te for **2** and **4**). Minor signals from Al and Si are attributed to the sample substrate and mounting materials. Importantly, chalcogen signals persist after TGA data collection but at reduced and spatially heterogeneous levels, as evidenced by decreased Se or Te amounts in post-TGA EDS maps (Figures S64, S66, and S68), consistent with partial volatilization or redistribution of chalcogen-containing material during heating. These observations are consistent with PXRD analysis of the post-TGA residues, which revealed UO_2_/UO_2+*x*_ formation and a lack of peaks corresponding with crystalline TeO_2_. Notably, backscattered electron (BSE)-SEM images of UO_2_/UO_2+x_ residues obtained after TGA show differences in aggregate morphology and packing that depend on the identity of the chalcogenophene linker (Figure 6). UO_2_/UO_2+x_ obtained from the thermal degradation of selenophene-containing compound **3** forms denser, angular aggregates with well-defined edges and layered surface features, appearing as compact, high-contrast domains consistent with closely packed particles of uniform composition (Figure 6b). In contrast, UO_2_/UO_2+x_ obtained from the tellurophene-containing compound **4** exhibits a more open aggregate morphology, appearing as loosely packed, granular regions with greater contrast variation in BSE images (Figure 6d).

**Figure 6:**
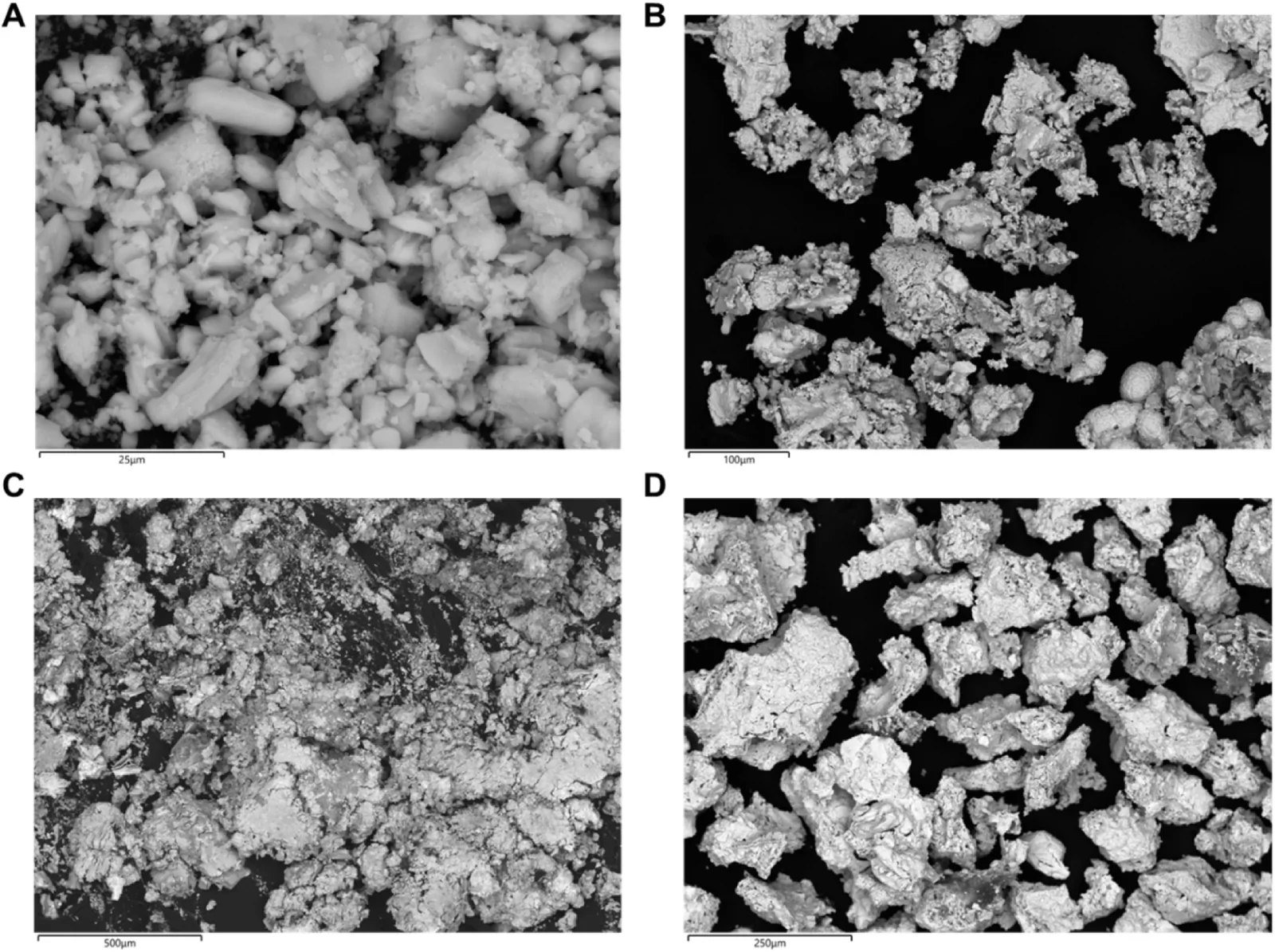
BSE-SEM images of (**a**) pre-TGA compound **3**, (**b**) post-TGA compound **3**, (**c**) pre-TGA compound **4**, and (**d**) post-TGA compound **4**.

Building on the SEM-EDS results obtained for intact uranyl CPs, we next examined a representative pre- and post-TGA physical mixing experiment to evaluate whether analogous elemental and morphological trends emerge in the absence of extended framework connectivity. A 1:1 stoichiometric physical mixture of U­(VI)–2,5-TDC-DMSO with 2,5-TeDC was selected as a representative system and examined before and after TGA data collection under inert atmosphere (Figures S69 and S70). Prior to heating, BSE-SEM images reveal a heterogeneous assembly of distinct particulate domains corresponding to the uranyl–TDC phase and the 2,5-TeDC ligand (Figure 7a). EDS spectra and elemental maps collected before TGA data collection confirm the presence of uranium, oxygen, carbon, sulfur, and tellurium, with Te-rich regions spatially segregated from uranium-rich domains, as expected for a simple physical mixture (Figure S69). Following TGA measurement, pronounced changes in both composition and morphology are observed. BSE-SEM images show the collapse of the initial heterogeneous morphology into aggregated uranium-rich domains with increased contrast uniformity, consistent with expectations for uranium oxide residues (Figure 7b). Correspondingly, post-TGA EDS analysis revealed a substantial decrease in carbon intensity accompanied by an increase in the relative oxygen contributions across sampled regions (Figure S70). Tellurium signals remain detectable after thermal treatment but at markedly reduced and spatially heterogeneous intensities, indicating partial loss or redistribution of Te during decomposition, rather than retention as a crystalline tellurium oxide phase. These observations are consistent with PXRD analysis of the post-TGA residues, which confirms formation of UO_2_/UO_2+x_ while showing no evidence for crystalline tellurium oxide formation. The persistence of reduced but detectable Te signals even in the absence of framework connectivity also demonstrates that direct contact between uranyl species and the tellurophene ligand in the solid state is sufficient to promote U­(VI) to U­(IV) reduction during thermal decomposition through volatile, transient, low-valent organotellurium intermediates. The similarity between the trends observed for physically mixed samples and intact uranyl frameworks suggests that the chalcogen-driven redox behavior is not contingent upon extended network formation, rather it reflects an intrinsic property of the 2,5-TeDC linker under these conditions. Taken together, these results further reinforce our hypothesis that thermally generated organochalcogen, and specifically organotellurium, species act as transient reducing agents, while also highlighting that linker identity alone can dictate both the redox outcome and microstructural evolution of uranium oxide phases.

**Figure 7:**
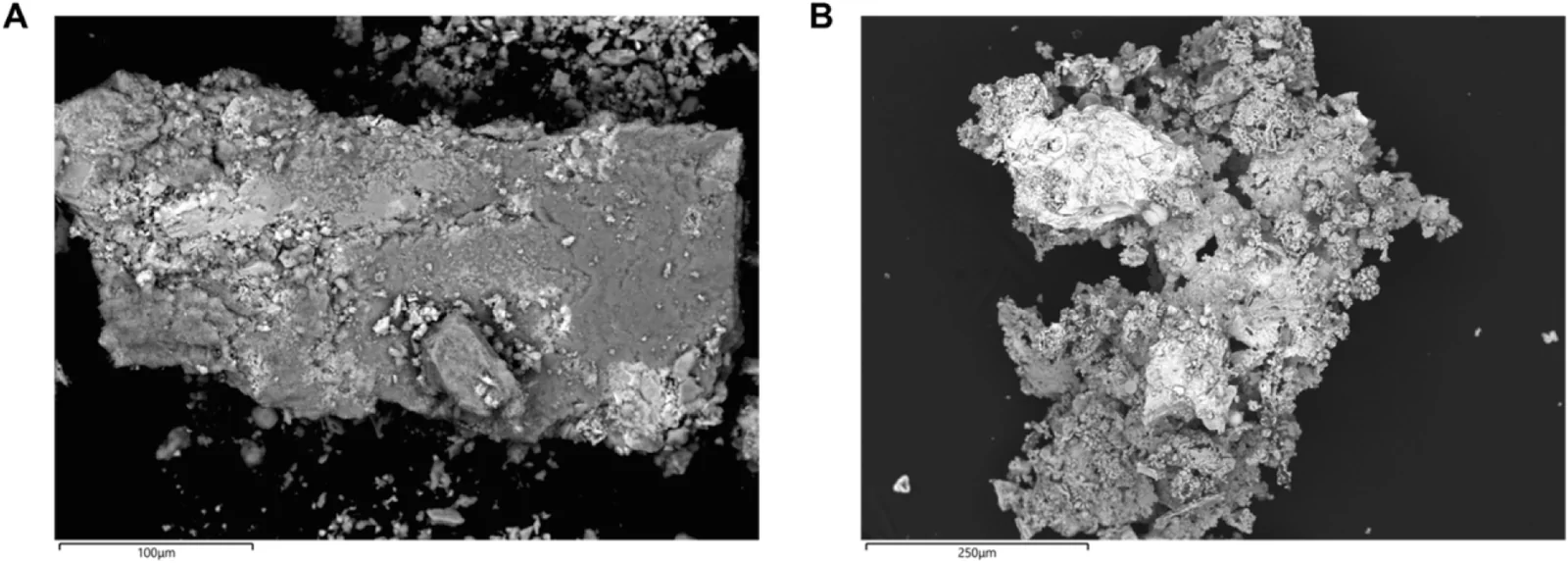
BSE-SEM images of physical mixture of (1:1) U­(VI)–2,5-TDC-DMSO with 2,5-TeDC (**a**) pre-TGA and (**b**) post-TGA.

#### Vibrational Spectroscopy

Raman and infrared (IR) spectroscopy are complementary techniques that offer valuable information about the bonding characteristics and local structural environments of uranyl compounds. The uranyl cation exhibits three distinct vibrational modes with its symmetric (ν_1_, 860–880 cm^–1^, Raman active), asymmetric (ν_3_, 930–960 cm^–1^, infrared active), and bending modes (ν_2_, observed between 150–300 cm^–1^ in the far-IR region) particularly responsive to ligand coordination. This sensitivity arises from the weakening of uranyl bonds due to equatorial ligand interactions. In this study, we built upon previous efforts in the literature to identify the effects of equatorial coordination and oxo interactions on uranyl vibrational spectra by extending our analysis to uranyl CPs (compounds **1**–**6**), and we focused on distinguishing the spectroscopic variations arising from linker identity (2,5-SeDC vs 2,5-TeDC) and differences in solvent coordination. Hence, Raman, mid-IR (MIR), and far-IR (FIR) spectra for compounds **1**–**6** were collected and peak assignments were made based on spectral fitting and previous literature results. All fitted spectra in the spectral windows of interest (1600–400 cm^–1^ for Raman and 400–100 cm^–1^ for far-IR) can be found in Figures S33–S38 and S47–S52, respectively.

Looking at the Raman spectra for the 2,5-SeDC containing compounds **1**, **3**, and **5** reveals distinct spectral shifts in the characteristic ν_1_ symmetric stretching mode of the uranyl cation as well as ligand associated vibrations that reflect variations in bonding and local environments. The Raman spectrum of **1** was fitted using Gaussian peak profiles, whereas **3** and **5** were fitted using Lorentzian peak profiles, and all three spectra are presented in Figure 8. In each of the compounds, the dominant ν_1_ (U=O) symmetric stretching mode is observed, and this band is at 844 cm^–1^ in **1**, 836 cm^–1^ in **3**, and appears as a dominant band at 852 cm^–1^ with an additional weaker and broadened feature at 805 cm^–1^ in **5**. These frequencies are red-shifted compared to the free uranyl cation (860–880 cm^–1^ in aqueous solution), indicating that equatorial ligand coordination weakens U=O bonds. The most pronounced redshifts are observed in **3** and **5**, and notably, the spectrum for compound **5** also exhibits a unique low frequency ν_1_ feature at 805 cm^–1^. This band is attributed to the presence of U­(VI) CCIs in **5**, which are known to result in lower ν_1_ frequencies.^115,116^ Consistent with this interpretation, compound **5** displays an elongated axial U1–O1 bond (1.812(14) Å) corresponding to the oxo atom participating in the CCI, which is significantly longer than those in the other compounds and correlates with the observed ν_1_ red shift.

**Figure 8:**
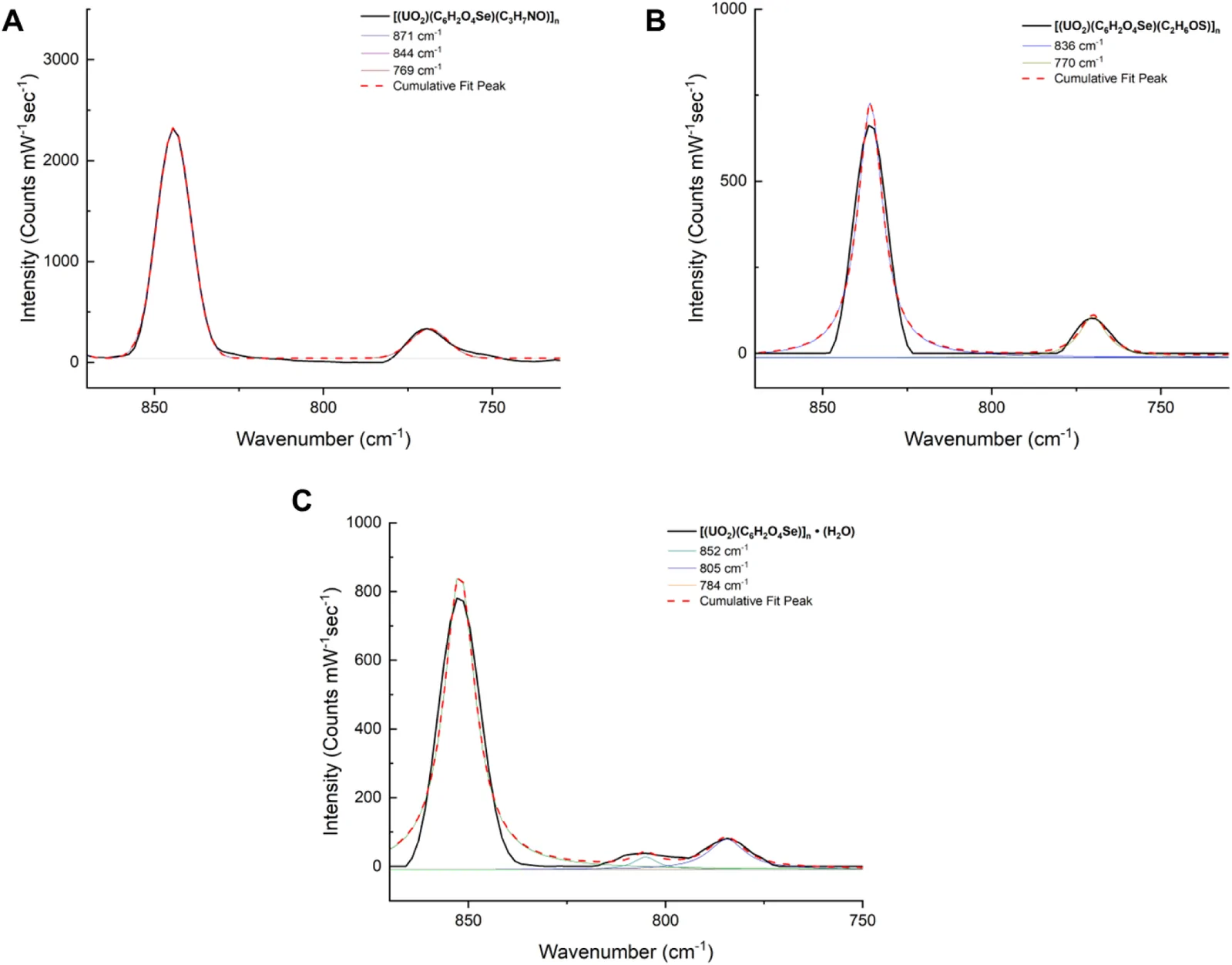
(**a**) Raman spectra of compound **1**, (**b**) compound **3**, and (**c**) compound **5** in the 900–750 cm^–1^ spectral window fit to Gaussian (**1**) or Lorentzian (**3** and **5**) functions.

Switching to the Raman spectra of the 2,5-TeDC containing compounds **2**, **4**, and **6**, we observed the U=O ν_1_ symmetric stretch at 839 cm^–1^ for compound **2**, 829 cm^–1^ for compound **4**, and 824 cm^–1^ for compound **6** (Figure 9). These assignments are based on Lorentzian fittings of Raman spectra for each compound in the spectral window of interest (1600–400 cm^–1^), and ν_1_ values here are consistently red-shifted compared to the free uranyl cation. The lowest energy ν_1_ stretching frequency was observed in **6**, which may be a result of the slight elongation of one uranyl axial bond at 1.793(15) Å, compared to U=O bond distances of 1.770(18) and 1.756(18) Å in compound **2** and 1.776(9) and 1.777(9) Å in compound **4**. Across the tellurophene-based series, axial and equatorial U–O bond distances remain comparable regardless of coordinated solvent, with compound **6** displaying equatorial bond distances within the same range as **2** and **4** (2.343(8)–2.430(8) Å), providing no evidence for enhanced equatorial electron donation. The redshift of ν_1_ in compound **6**, despite these structural similarities, indicates that the observed vibrational differences cannot be attributed solely to solvent coordination. Instead, these results point to the influence of subtle structural features, such as differences in crystal packing, that may modulate vibrational behavior without producing major geometric distortions in the uranyl coordination environment.

**Figure 9:**
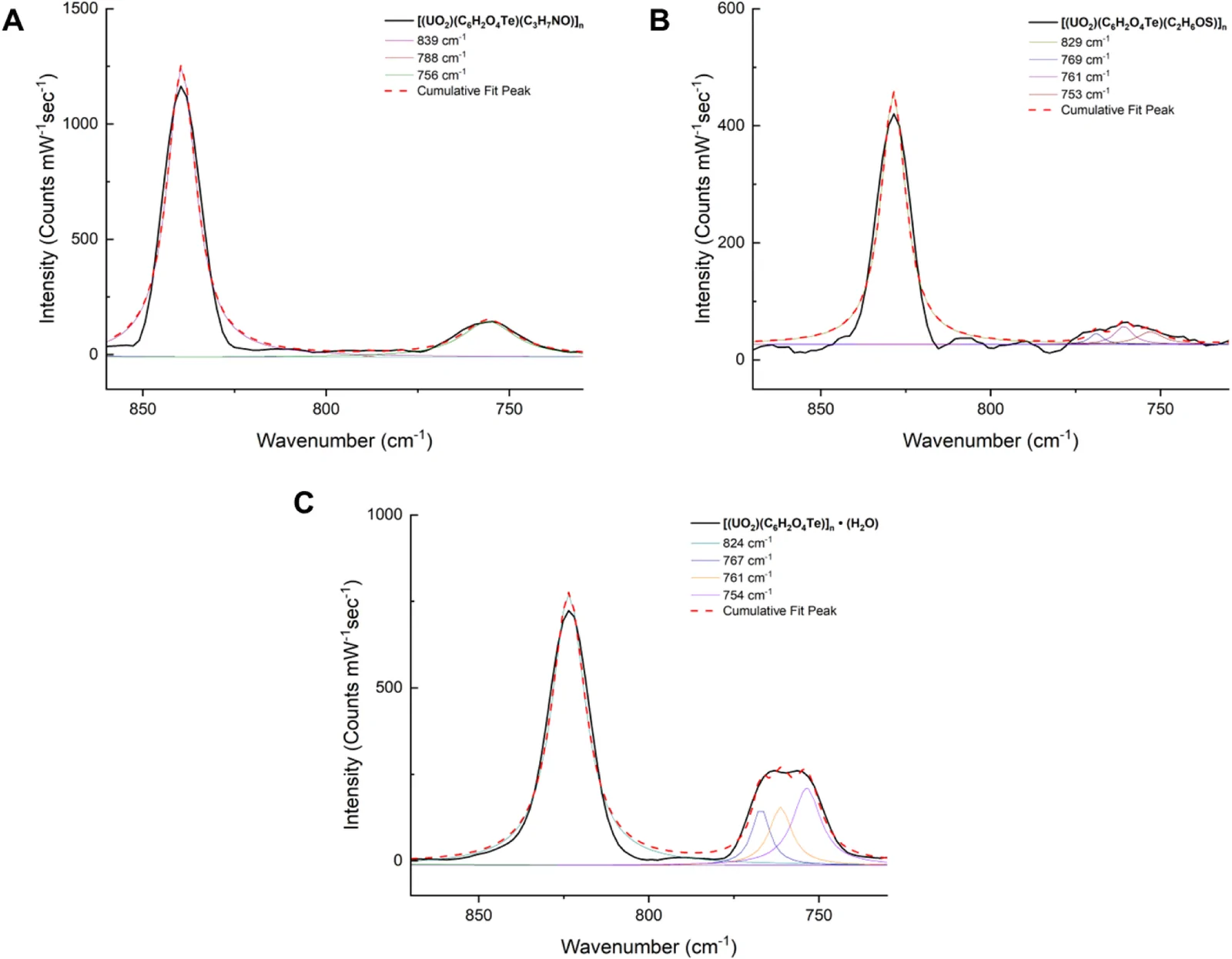
(**a**) Raman spectra of compound **2**, (**b**) compound **4**, and (**c**) compound **6** in the 900–750 cm^–1^ spectral window fit to Lorentzian functions.

The mid- and far-IR spectra of compounds **1**, **3**, and **5**, all of which contain 2,5-SeDC, display distinct variations in uranyl stretching frequencies and subtle differences in ligand-based vibrations.^116–118^ The fitted far-IR spectra of compounds **1**, **3**, and **5** are presented in Figures S47, S49, and S51, while the mid-IR spectra are shown in Figures S41, S43, and S45. In the mid-IR region, the ν_3_(U=O) asymmetric stretch is observed as a sharp band around approximately 900 cm^–1^ for **1**, **3**, and **5**. The assignment of these modes as ν_3_(U=O) asymmetric stretches is also supported by their characteristic patterns and spectral intensities, differentiating them from symmetric ν_1_(U=O) modes observed at lower frequencies in the Raman spectra. Ligand-based modes including C–Se (∼610 cm^–1^), aromatic C–C (∼1450 cm^–1^), and C=C (∼1600 cm^–1^) stretches remain consistent within ±10 cm^–1^ across all three structures, confirming minimal perturbation to the 2,5-SeDC backbone. In the far-IR region, multiple absorptions are observed between 300 and 200 cm^–1^ for compounds **1**, **3**, and **5**, which is the spectral window where uranyl ν_2_(O=U=O) bending modes are commonly reported in solid-state uranyl compounds. For compound **1**, spectral bands are observed at 257 and 229 cm^–1^, the spectrum for compound **3** includes features at 278, 259, 242, 218, and 206 cm^–1^, and the spectrum for compound **5** displays bands at 273, 255, 238, and 222 cm^–1^. In extended uranyl networks, ν_2_ bending vibrations are frequently split and strongly coupled with lattice and ligand motions, and these features can therefore be treated as containing contributions from uranyl bending mixed with framework vibrations rather than being assigned as isolated ν_2_ modes. Additional lower frequency bands below 200 cm^–1^ are also present, including peaks at 185, 176, 157, and 143 cm^–1^ in compound **1**, 170 and 155 cm^–1^ in compound **3**, and 187, 171, and 143 cm^–1^ in compound **5**, which can be attributed primarily to lattice modes and coupled motions involving equatorial U–O coordination and ligand deformation. Features in the 400–300 cm^–1^ range remain largely unchanged, further reflecting framework vibrations that are not strongly modulated by changes in local coordination.

The mid- and far-IR spectra of 2,5-TeDC containing compounds **2**, **4**, and **6** reveal variations that highlight the influence of chalcogen substitution on the overall vibrational landscape of uranyl frameworks.^116,118,119^ The mid-IR and fitted far-IR spectra of **2**, **4**, and **6** covering the spectral window of interest (4000–100 cm^–1^) are shown in Figures S42, S44, S46, S48, S50, and S52. The mid-IR spectra of compounds **2**, **4**, and **6** feature the uranyl ν_3_(U=O) asymmetric stretch as a dominant, intense band near 900 cm^–1^. In each case, this stretching mode is substantially more intense than neighboring bands and falls within the characteristic frequency range for the uranyl asymmetric stretch. Ligand-based modes including C–Te (∼530–700 cm^–1^), aromatic C–C (∼1450 cm^–1^), and C=C (∼1600 cm^–1^) stretches remain consistent within ±10 cm^–1^ across compounds **2**, **4**, and **6**, indicative of minimal perturbation of the 2,5-TeDC backbone, analogous to the 2,5-SeDC series. In the far-IR region, compounds **2**, **4**, and **6** display multiple stretching modes between 300 and 200 cm^–1^. For compound **2**, spectral bands are observed at 295, 258, 241, and 213 cm^–1^, the spectrum for compound **4** exhibits features at 301, 265, 246, and 207 cm^–1^, and the spectrum for compound **6** displays bands at 275, 251, 238, 216, and 200 cm^–1^. Additional lower frequency bands below 200 cm^–1^ (e.g., 188, 168, and 152 cm^–1^ in **2**, 180 and 157 cm^–1^ in **4**, and 182, 155, and 142 cm^–1^ in **6**) are attributed primarily to lattice vibrations and coupled motions involving equatorial U–O coordination and ligand deformation. Similar to 2,5-SeDC containing compounds **1, 3**, and **5**, far-IR spectra for **2**, **4**, and **6** include features in the 400–300 cm^–1^ region that remain largely unchanged across the series, consistent with framework-dominated modes that are not strongly influenced by changes in local coordination.

## Conclusions

In this study, we demonstrated that incorporation of heavier chalcogenophene-based dicarboxylic acid linkers provides access to eight novel uranyl CPs that combine three-dimensional extended architectures with unique ligand-driven redox behavior. These findings establish heavier chalcogenophene-based linkers as a powerful and previously underexplored design element in uranyl coordination chemistry. Structural analysis confirms that these ligands behave as multitopic dicarboxylates capable of supporting extended connectivity, placing them within the broader class of linkers known to generate higher dimensional uranyl hybrid materials, while also imparting distinct chalcogen-dependent structural and packing features. Thermogravimetric analysis revealed that the chalcogenophene-based U­(VI) frameworks were thermally stable up to 250–350 °C, prior to complete ligand decomposition. Most remarkably, heating of uranyl–2,5-SeDC and 2,5-TeDC CPs under inert atmosphere reproducibly leads to formation of a reduced UO_2_/UO_2+x_ phase rather than higher-valent uranium oxides, representing a ligand-dependent example of spontaneous U­(VI) to U­(IV) reduction in the solid state that does not require addition of harsh chemical reductants or use of elevated pressures. This behavior contrasts sharply with that of uranyl hybrid materials constructed from furan- or thiophene-based linkers, which do not undergo analogous metal-ion reduction under identical conditions. Physical mixing experiments further demonstrated that the presence of 2,5-TeDC alone is also sufficient to promote this U­(VI) to U­(IV) reduction, even in the absence of extended framework connectivity. Pre- and post-TGA SEM–EDS analyses revealed substantial loss of chalcogen content upon heating, particularly for tellurium-containing systems, while PXRD patterns displayed no evidence for crystalline selenium or tellurium oxide byproducts. Together, these observations support a mechanistic pathway in which thermally generated, volatile low-valent chalcogen-containing species (e.g., hydrides) formed during linker decomposition act as transient reducing agents, enabling spontaneous *in situ* reduction of U­(VI) to U­(IV) through solid-state contact. Beyond establishing a novel class of three-dimensional uranyl CPs, the findings herein demonstrate the potential for linker identity to be leveraged not only to control extended structure formation in uranyl systems, but also to mediate redox transformations of actinide centers under mild thermal conditions. The ability of heavier chalcogen-containing linkers to couple structural roles with redox activity provides a new perspective on how framework decomposition pathways, electronic structure, and the nature of resulting oxide products may be intrinsically connected. Overall, this study expands the scope of uranyl and actinide CP chemistry and provides rare examples of spontaneous linker-induced U­(VI) reduction in the solid state, offering a foundation for future studies probing actinide redox chemistry and hybrid material design.

## Supplementary Material



## Data Availability

The data that support the findings of this study are available in the Supporting Information section of this article.

## Notes

^1^H and ^13^C NMR data for CP linkers, crystallographic data and refinement details, structural details for minor products, PXRD patterns for compounds **1**–**8**, post-TGA PXRD patterns, physical mixing experimental details, Raman and IR spectra for **1**–**6**, fitted Raman and IR spectra with vibrational mode assignments, TGA curves for compounds **1**–**6**, FE-SEM images and EDS analysis for selected compounds, Mercury Pore Analysis, and topological analysis (ToposPro) figures (PDF)
