# Supplementary material for: Synthesis and Characterization of Three-Dimensional Uranyl Coordination Polymers Containing Selenophene and Tellurophene Linkers
Source: Inorg Chem. 2026 Aug 3;65(32):18711–29. doi: 10.1021/acs.inorgchem.6c02207 (PMC13488406; doi:10.1021/acs.inorgchem.6c02207)
Supplement: Supplementary file 1 [file ic6c02207_si_001.pdf]

Synthesis and Characterization of Three-Dimensional Uranyl Coordination Polymers Containing Selenophene and Tellurophene Linkers

Ecem Çelik,[1] Vamsi K. Karapala,[1] Brett Lottes,[1] Michael A. Sinnwell,[1,2] and Korey P. Carter[1]*

[1] Department of Chemistry, University of Iowa, Iowa City, IA 52242, United States

[2] Office of the Vice President for Research, University of Iowa, Iowa City, IA 52242, United States

* Corresponding author's e-mail: korey-carter@uiowa.edu

Table of Contents

Ligand Synthesis Details .... S**Error! Bookmark not defined.**

Nuclear Magnetic Resonance (NMR) S4

Hybrid Material Synthesis Details S7

Synthetic Procedures for 2,5-TeDC Containing Compounds S7

Mercury Pore Analysis S8

Single Crystal X-ray Diffraction (SC-XRD) S9

Refinement Details S16

Crystallographic Details S18

Synthetic, Refinement, and Crystallographic Details of an Additional Compound (U(VI)–2,5-FDC) S25

Powder X-Ray Diffraction (PXRD) S27

Summary of Physical Mixing Experiments S35

Proposed Mechanism Scheme for U(VI) to U(IV) Redox Transformation S36

Vibrational Spectroscopy S37

Thermogravimetric Analysis (TGA) S54

Scanning Electron Microscopy (SEM) and Energy Dispersive Spectroscopy (EDS) S58

Topological Analysis (ToposPro) S67

References S671

Ligand Synthesis Details

Synthesis of 2,5-selenophene dicarboxylic acid (2,5-SeDC)

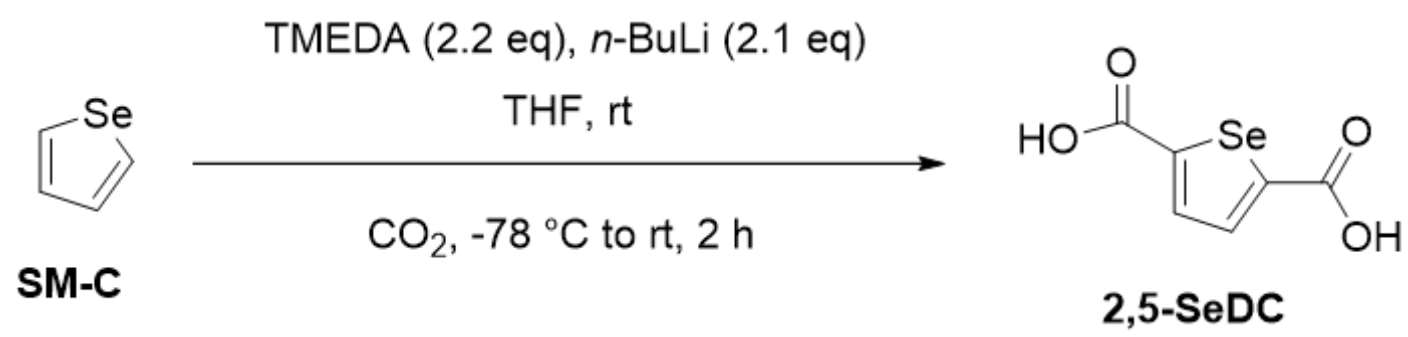

Scheme S1. One-step synthetic pathway used herein to synthesize **2,5-SeDC**.

The reaction was carried out using oven-dried glassware charged with a magnetic stir bar and conducted under an inert nitrogen atmosphere using typical Schlenk techniques. To a stirred solution of selenophene (**SM-C**) (10.0 g, 7.63 mmol, 1 eq) in dry THF (10x volume to **SM-C**), TMEDA (1.95 g, 16.7 mmol, 2.2 eq) was added and then *n*-BuLi (6.4 mL, 2.1 eq) was added slowly at room temperature. The reaction was then allowed to stir at 50 °C for two hours. After cooling to room temperature, the reaction was brought to -78 °C by placing the round bottom in a liquid N_2/ethyl acetate bath. Solid carbon dioxide (4.02 g, 91.56 mmol, 12 eq) was directly added to the reaction, which was stirred at room temperature for two hours. The reaction was slowly quenched with 1 N HCl at 0 °C, and subsequently the precipitate was filtered, washed with water (3 × 10 mL), and dried under vacuum to yield pure **2,5-SeDC** (590 mg, yield, 35.3%). **^{1}H NMR** (300 MHz, d_6-DMSO): δ 13.48 (s, 2H), 7.95 (s, 2H) (**Figure S1**, **Supporting Information**).

Synthesis of tellurophene and 2,5-tellurophene dicarboxylic acid (2,5-TeDC)

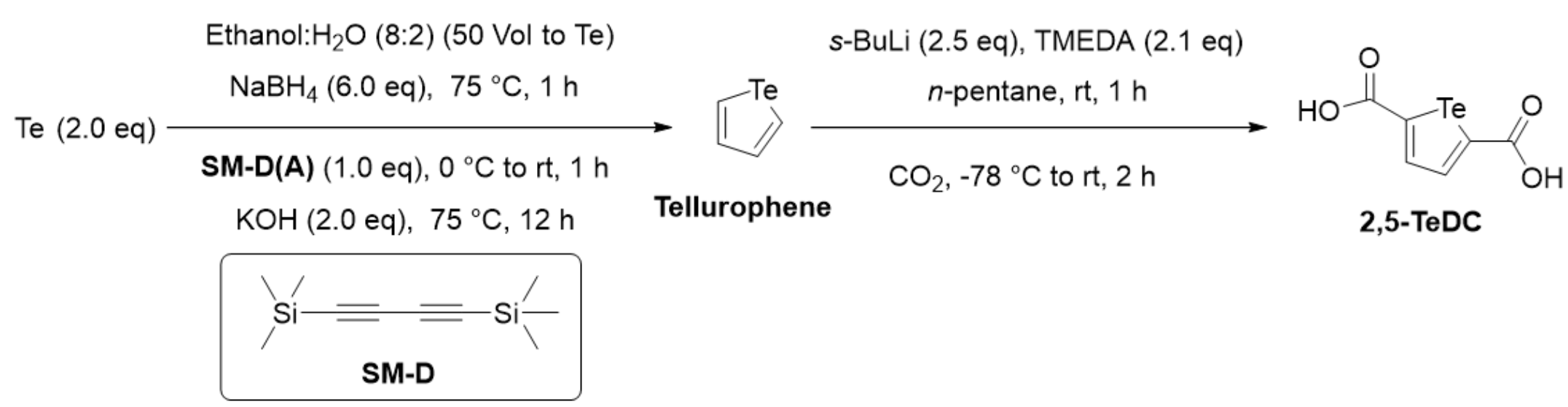

Scheme S2. Two-step synthetic pathway used herein to synthesize tellurophene and **2,5-TeDC**.

Tellurophene is not commercially available, so this heterocycle had to be synthesized ahead of preparing **2,5-TeDC**. Tellurophenes and related 2,5-disubstituted tellurophene derivatives have been previously reported, providing synthetic precedent for the preparation of functionalized tellurophene linkers,[1-3] and here we present modified procedures for both tellurophene and **2,5-TeDC** that results in improved synthesis yields.

The reaction to make tellurophene was carried out in a 250 mL two-neck round-bottom flask backfilled with nitrogen gas and equipped with a magnetic stir bar and reflux condenser using typical Schlenk techniques. Te (0.656 g, 5.14 mmol, 2.0 eq) and $NaBH_4$ (0.583 g, 15.4 mmol, 6.0 eq) were added to a degassed ethanol:water (8:2) solvent mixture and refluxed for one hour. 1,4-

bis(trimethylsilyl)-1,3-butadiyne, **SM-D,** (0.5 g, 2.57 mmol, 1.0 eq) was slowly added to the reaction at 0 °C and was allowed to stir to room temperature for one hour. Subsequently, KOH powder (0.288 g, 5.14 mmol, 2.0 eq) was added to the reaction, which was then stirred to reflux for twelve hours. The reaction was quenched by exposing it to the atmosphere for 30 minutes at vigorous stirring as this allows for complete oxidation of any remaining Na_2Te, which prevents precipitation during workup. The mixture was filtered through Celite and washed with diethyl ether, and the filtrate was then diluted with water and diethyl ether before extraction with pentane (3 × 50 mL). The combined organic layers were dried over sodium sulfate and concentrated under reduced pressure (>400 torr, @ rt, to avoid co-evaporation of the tellurophene product). The obtained crude product was then purified using column chromatography with pentane as an eluent (220 mg, 72%). **^{1}H NMR** (500 MHz, $CDCl_3$): δ 8.977-8.961 (m, $H_{2,5}$ with Te satellites), 7.849-7.260 (m, $H_{3,4}$ with Te satellites). (Note: Peaks below δH 4 ppm correspond to pentane, which remained in the final product and was integrated for calculation of the product mass), **^{13}C NMR** (500 MHz, $CDCl_3$): δ 137.97, 126.31, 65.99, 15.40 (Note: Peaks at δH 65.99 and 15.40 ppm correspond to diethyl ether, which remained in the final product and was integrated for calculation of the product mass), **^{125}Te NMR** (500 MHz, $CDCl_3$): δ 778.0 (**Figures S2-S4**, **Supporting Information**).

The reaction to make **2,5-TeDC** was also carried out in a 250 mL two-neck round-bottom flask backfilled with nitrogen gas and equipped with a magnetic stir bar and reflux condenser using typical Schlenk techniques. To a stirred solution of tellurophene (0.3 g, 1.66 mmol, 1 eq) in dry pentane (10x volume to tellurophene), TMEDA (0.407 g, 3.50 mmol, 2.1 eq) was added and then s-BuLi (5.0 mL, 4.17 mmol, 2.5 eq) was added slowly. The reaction was allowed to stir at room temperature for two hours and then was cooled to -78 °C by placing the round bottom in a liquid N_2/ethyl acetate bath. Solid carbon dioxide (0.730 g, 16.69 mmol, 10 eq) was added and the reaction was allowed to stir to room temperature for two hours. The reaction was slowly quenched with 1 N HCl at 0 °C, then the precipitated solid was filtered, and subsequently, washed with water (3 × 10 mL), and dried under vacuum to yield pure **2,5-TeDC** (446 mg, yield 47.3%). **^{1}H NMR** (500 MHz, d_6-DMSO): δ 13.183 (br. s, 2H), 8.458 (s, 2H), **^{13}C NMR** (500 MHz, d_6-DMSO): δ 166.73, 146.27, 143.14 (**Figures S5** and **S6**, **Supporting Information**).

Nuclear Magnetic Resonance (NMR)

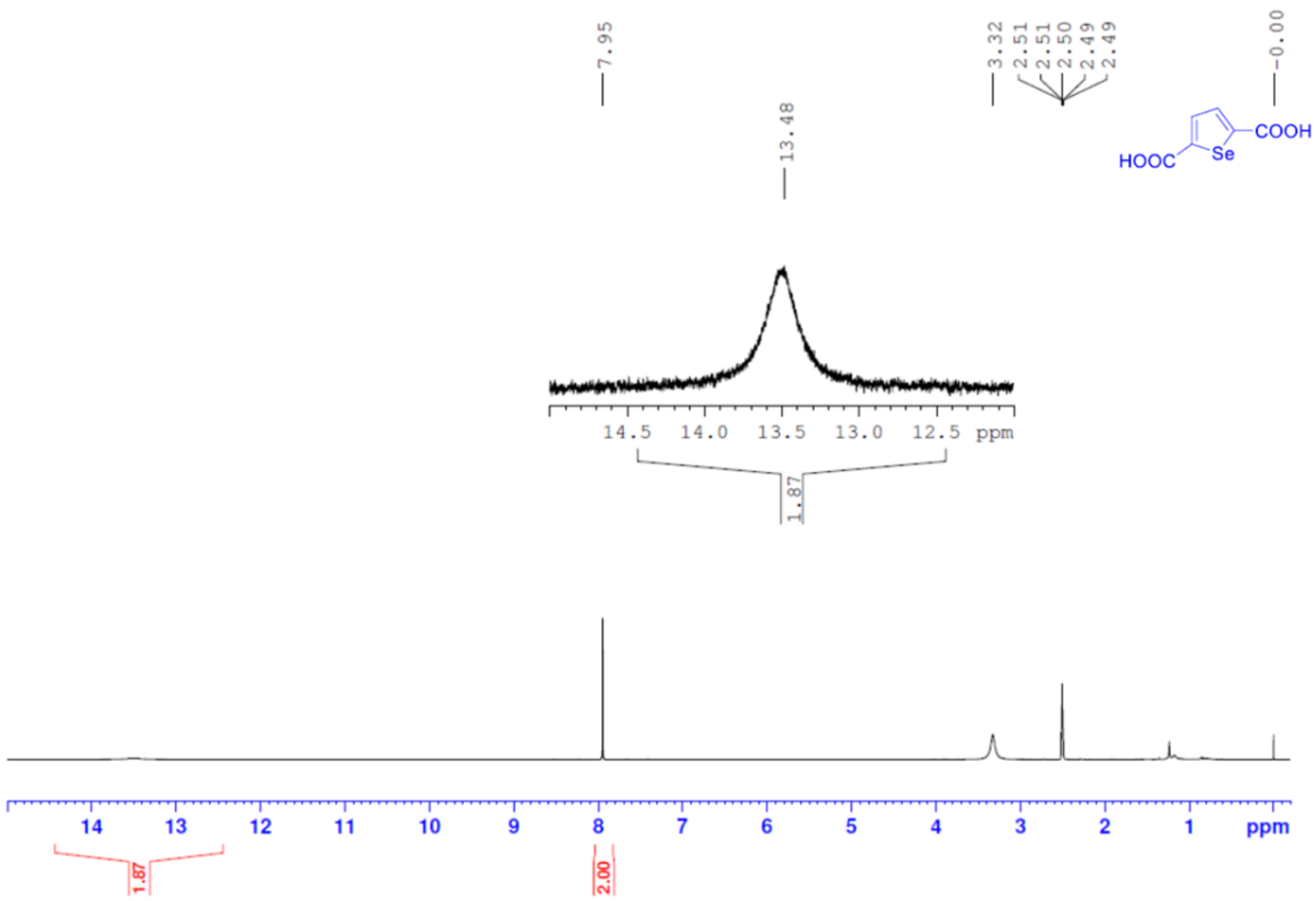

Figure S1. ^{1}H NMR spectrum (400 MHz, d_6-DMSO) for 2,5-selenophenedicarboxylic acid (2,5-SeDC).

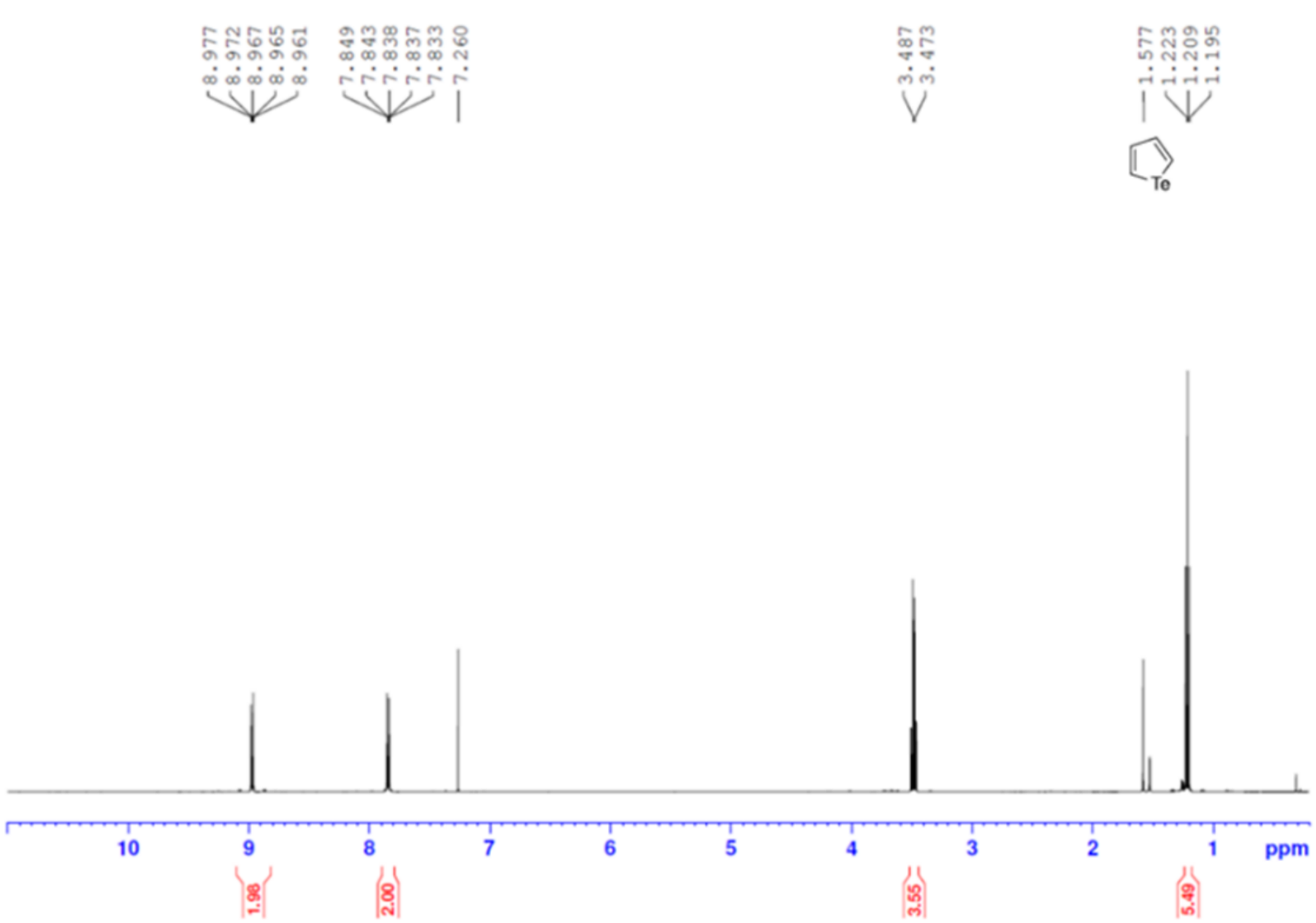

Figure S2. ^{1}H NMR spectrum (500 MHz, $CDCl_3$) for tellurophene.

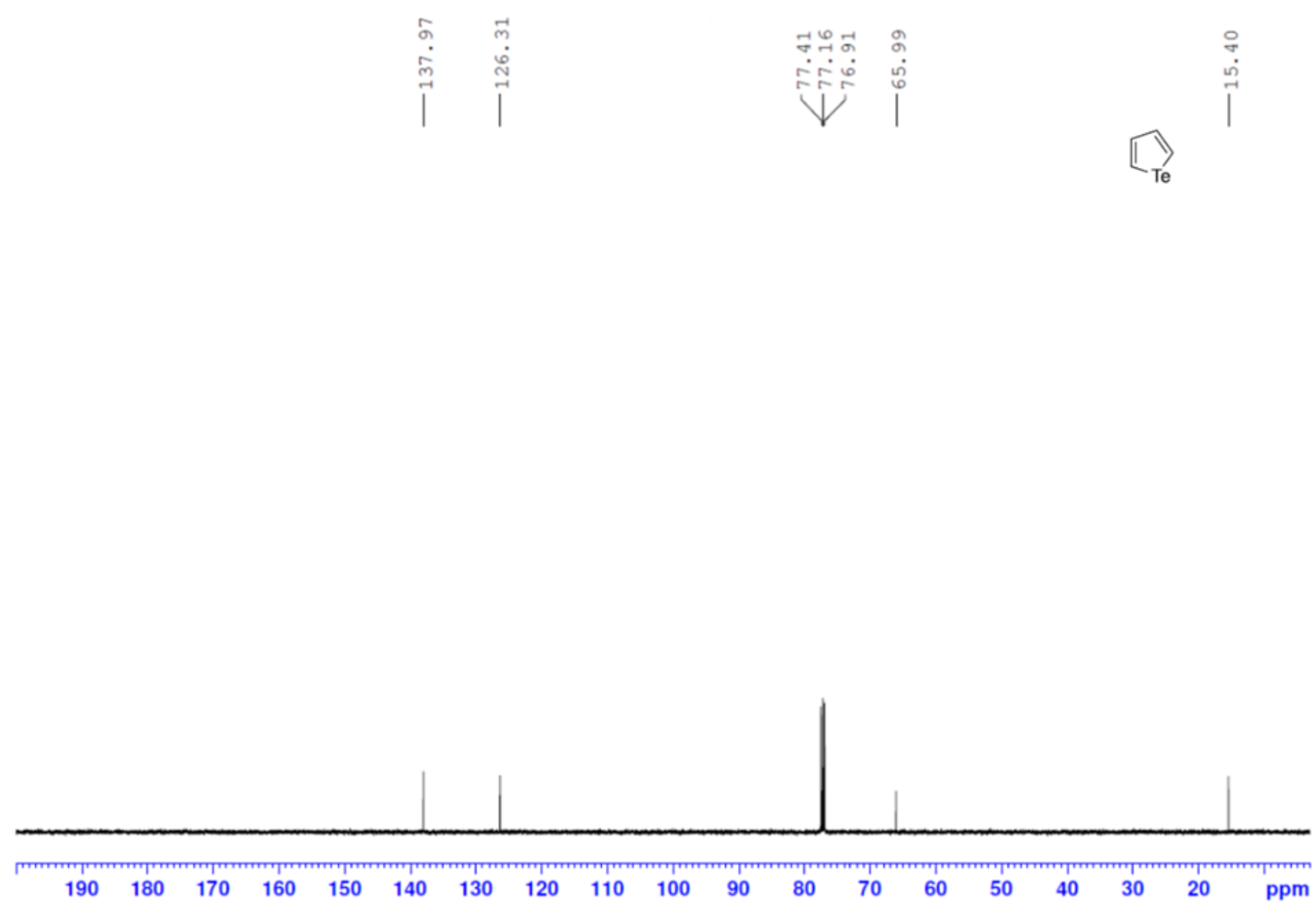

Figure S3. ^{13}C NMR spectrum (500 MHz, $CDCl_3$) for tellurophene.

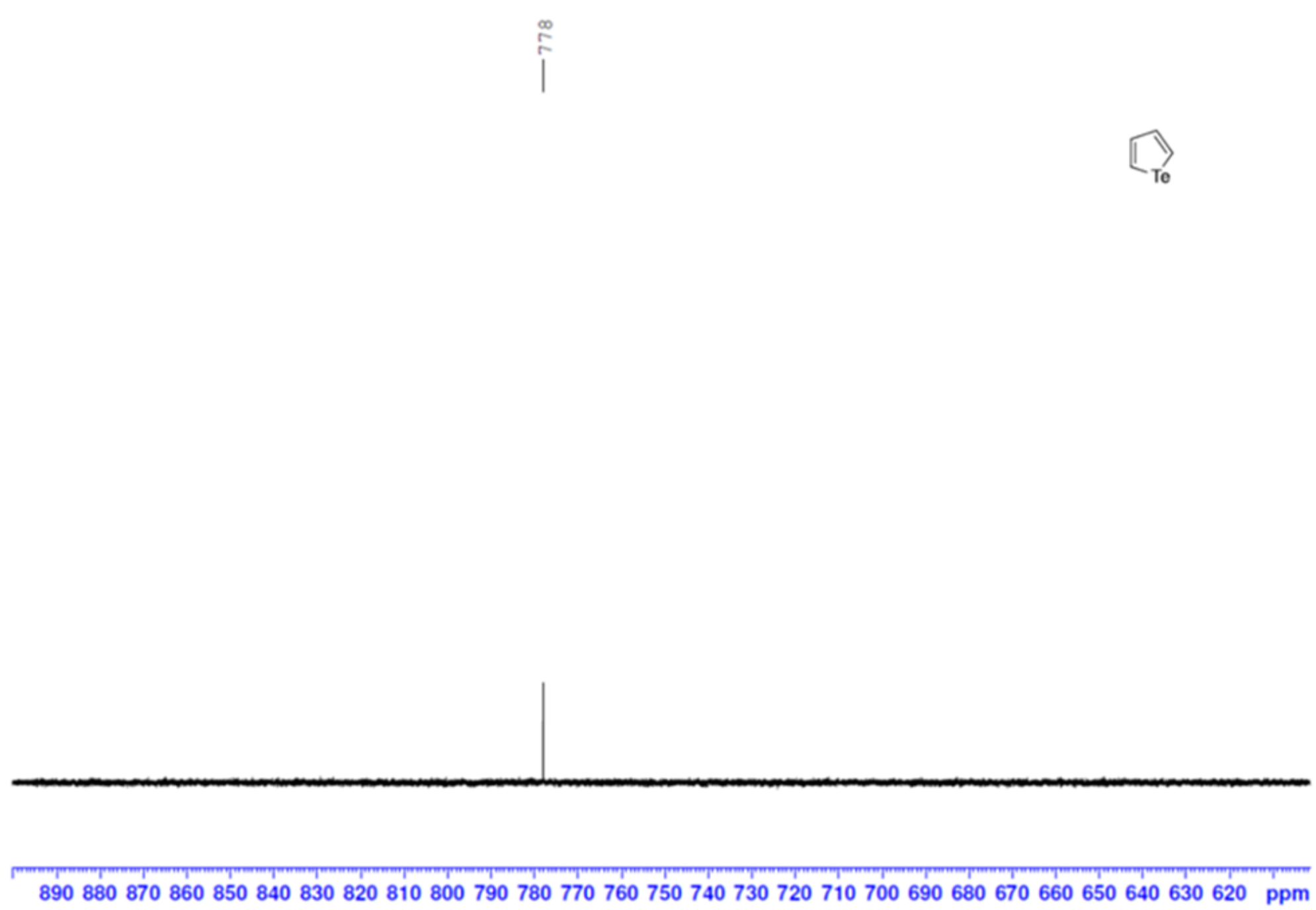

Figure S4. ^{125}Te NMR spectrum (500 MHz, $CDCl_3$) for tellurophene.

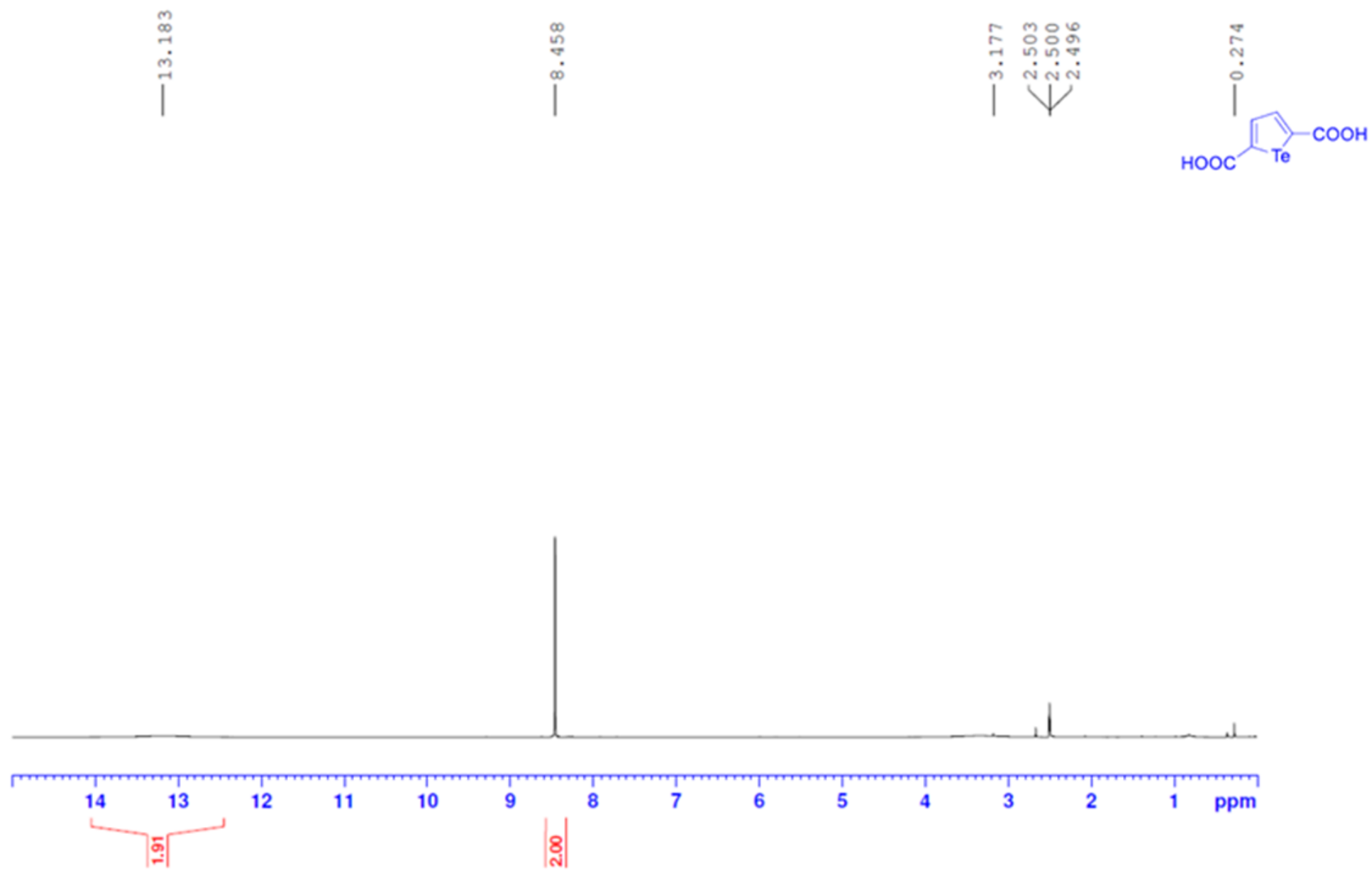

Figure S5. ^{1}H NMR spectrum (500 MHz, d_6-DMSO) for 2,5-tellurophenedicarboxylic acid (2,5-TeDC).

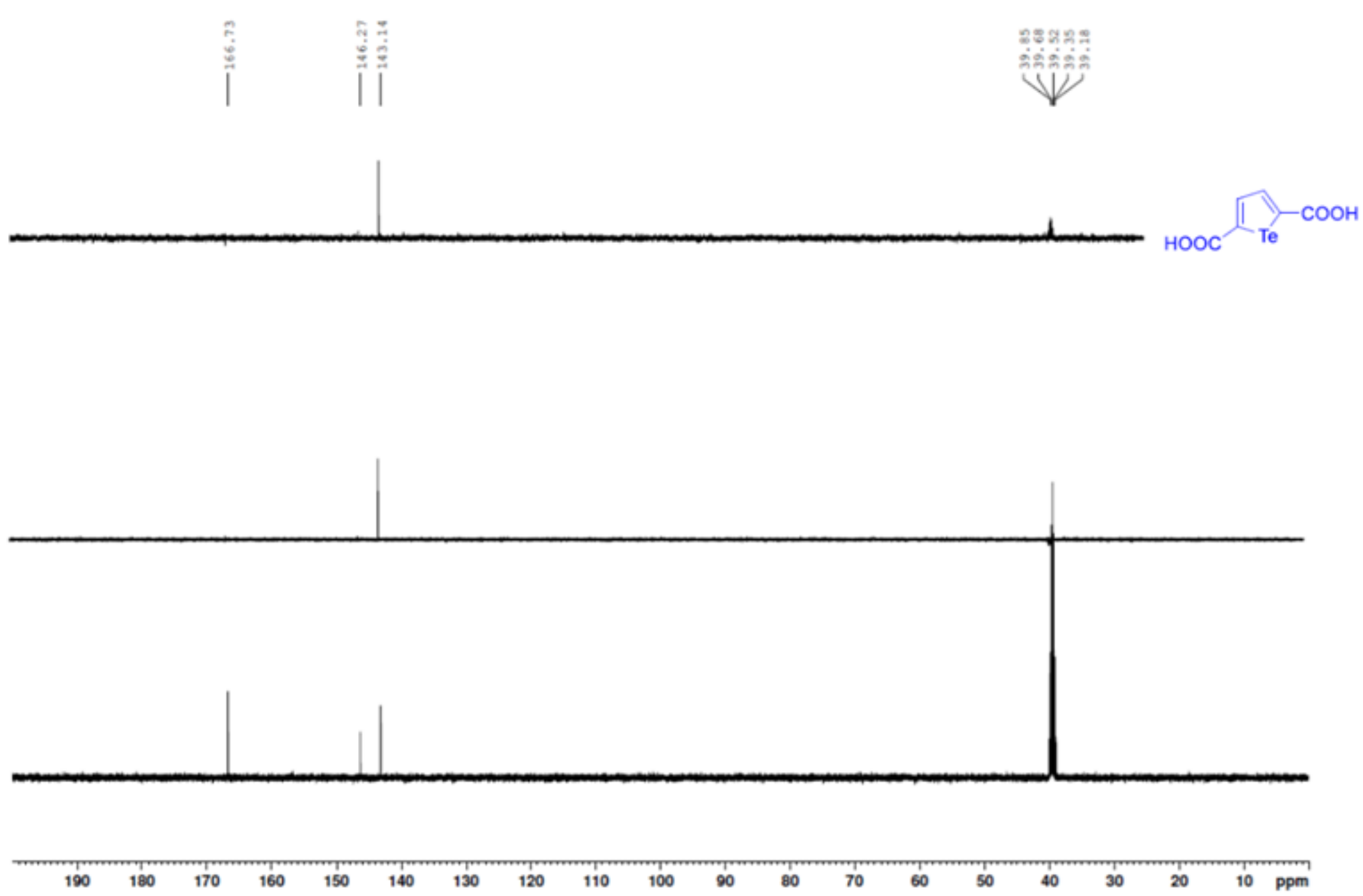

Figure S6. ^{13}C (500 MHz, d_6-DMSO) **(bottom)**, DEPT-90 (d_6-DMSO) **(middle)** and DEPT-135 (d_6-DMSO) **(top)** NMR spectra of 2,5-TeDC ligand. The DEPT-90 and DEPT-135 spectra show no CH or CH_2 signals for the aromatic region, confirming that all tellurophene carbons are quaternary.

Hybrid Material Synthesis Details

Synthetic Procedures for 2,5-TeDC Containing Compounds

Synthesis of $[(UO_2)(C_6H_2O_4Te)(C_3H_7NO)]_n$ (2):

Synthesis of compound **2** involved adding 0.0251 g (50 μmol) $UO_2(NO_3)_2{\bullet}6H_2O$, 0.0134 g (50 μmol) 2,5-TeDC, 0.7549 g H_2O, and 38 μL DMF into a 23 mL Parr autoclave and placing it into an oven at 120 °C for 48 h. Once removed, the Parr autoclave was opened after cooling overnight and pale-yellow rectangular plate-like crystals suitable for single crystal X-ray diffraction were removed from the mother liquor and placed directly into high viscosity NVH oil.

Synthesis of $[(UO_2)(C_6H_2O_4Te)(C_2H_6OS)]_n$ (4):

Compound **4** was synthesized by combining 0.0217 g (50 μmol) $UO_2(CH_3COO)_2{\bullet}2H_2O$, 0.0134 g (50 μmol) 2,5-TeDC, 0.7799 g H_2O, and 40 μL DMSO in a 23 mL Parr autoclave and placing it into an oven at 120 °C for 48 h. Once removed, the Parr autoclave was opened after cooling overnight and pale-yellow rectangular plate-like crystals suitable for single crystal X-ray diffraction were removed from the mother liquor and placed directly into high viscosity NVH oil.

Synthesis of $[(UO_2)(C_6H_2O_4Te)]_n{\bullet}H_2O$ (6):

Synthesis of compound **6** involved combining 0.0217 g (50 μmol) $UO_2(CH_3COO)_2{\bullet}2H_2O$, 0.0134 g (50 μmol) 2,5-TeDC, and 0.7852 g H_2O in a 23 mL Parr autoclave and placing it into an oven at 120 °C for 48 h. Once removed, the Parr autoclave was opened after cooling overnight and pale-yellow rectangular plate-like crystals suitable for single crystal X-ray diffraction were removed from the mother liquor and placed directly into high viscosity NVH oil.

Synthesis of $[\{UO_2(C_6H_2O_4Se)_{1.5}\}^-_4{\bullet}2(H_2O){\bullet}4(C_2H_6N)^+]_n$ (7):

Compound **7** was prepared by mixing 0.0217 g (50 μmol) $UO_2(CH_3COO)_2{\bullet}2H_2O$, 0.0220 g (100 μmol) 2,5-SeDC, 0.7589 g H_2O, and 38 μL DMF in a 23 mL Parr autoclave and placing it into an oven at 120 °C for 48 h. Once removed from the oven and allowed to cool overnight, the reaction vessel was opened and pale-yellow rectangular plate-like crystals suitable for single crystal X-ray diffraction analysis were removed from the mother liquor and placed directly into high viscosity NVH oil.

Synthesis of $[\{UO_2(C_6H_2O_4Te)_{1.5}\}^-_2{\bullet}2.5(H_2O){\bullet}2(C_2H_6N)^+]_n$ (8):

Compound **8** was prepared by adding 0.0251 g (50 μmol) $UO_2(NO_3)_2{\bullet}6H_2O$, 0.0268 g (100 μmol) 2,5-TeDC, 0.7512 g H_2O, and 38 μL DMF into a 23 mL Parr autoclave and placing it in an oven at 120 °C for 48 h. Once removed, the Parr autoclave was opened after cooling overnight and pale-yellow rectangular plate-like crystals suitable for single crystal X-ray diffraction were removed from the mother liquor and placed directly into high viscosity NVH oil.

Mercury Pore Analysis

Pore analysis was performed using the Pore Analyzer tool implemented in Mercury, which evaluates geometric void space directly from crystallographic models. For compounds **1**-**6**, no solvent molecules were removed prior to analysis, as all solvent molecules present in these structures are coordinated and no solvent-accessible voids are present in the pores or interstitial regions. In contrast, for minor compounds **7** and **8**, solvent molecules residing within the pore space were removed from the structural models prior to analysis to evaluate the intrinsic framework porosity. Mercury Pore Analysis results for compounds **1-8** are reported in **Table S1**.

Table S1. Mercury Pore Analysis results for compounds **1-8**.

Parameter	1	2	3	4	5	6	7	8
**System volume (A^3)**	1286.8	1302.8	1279.8	1302.7	1960.5	974.9	3238.2	3798.21
**System mass (g/mol)**	2240.6	2435.1	2260.7	2455.3	3960.5	2214.8	4764.6	6058.98
**System density (g/cm^3)**	2.891	3.104	2.933	3.13	3.355	3.772	2.443	2.649
**Total surface area (A^2)**	0	0	0	0	0	0	0	67.81
**Total surface area per volume (A^2/cm^3)**	0	0	0	0	0	0	0	178.53
**Total surface area per mass (A^2/g)**	0	0	0	0	0	0	0	67.4
**Network-accessible surface area (A^2)**	0	0	0	0	0	0	0	43.79
**Network-accessible surface area per volume (A^2/cm^3)**	0	0	0	0	0	0	0	115.28
**Network-accessible surface area per mass (A^2/g)**	0	0	0	0	0	0	0	43.52
**Total helium volume (A^3)**	5.41	9.063	13.896	15.671	71.904	19.648	4470.56	532699.2
**Total geometric volume (A^3)**	319.2	320.5	339.7	336.1	586.5	231.7	1383.1	1848.97
**Network-accessible helium volume (A^3)**	5.41	9.063	13.894	15.671	71.883	19.648	4470.04	532699.2
**Network-accessible geometric volume (A^3)**	319.1	320.2	339.4	335.7	586.1	230.9	1382.16	1848.21
**Pore limiting diameter (Å)**	0.71	1	1.22	1.14	1.31	0.89	2.36	3.38
**Maximum pore diameter (Å)**	2.01	1.99	2.44	2.27	2.63	1.78	3.85	4.39
**Number of percolated dimensions**	0	0	1	1	2	0	1	1

Single Crystal X-ray Diffraction (SC-XRD)

Figures of Isomorphous Structures and SC-XRD Structural Descriptions of Minor Compounds

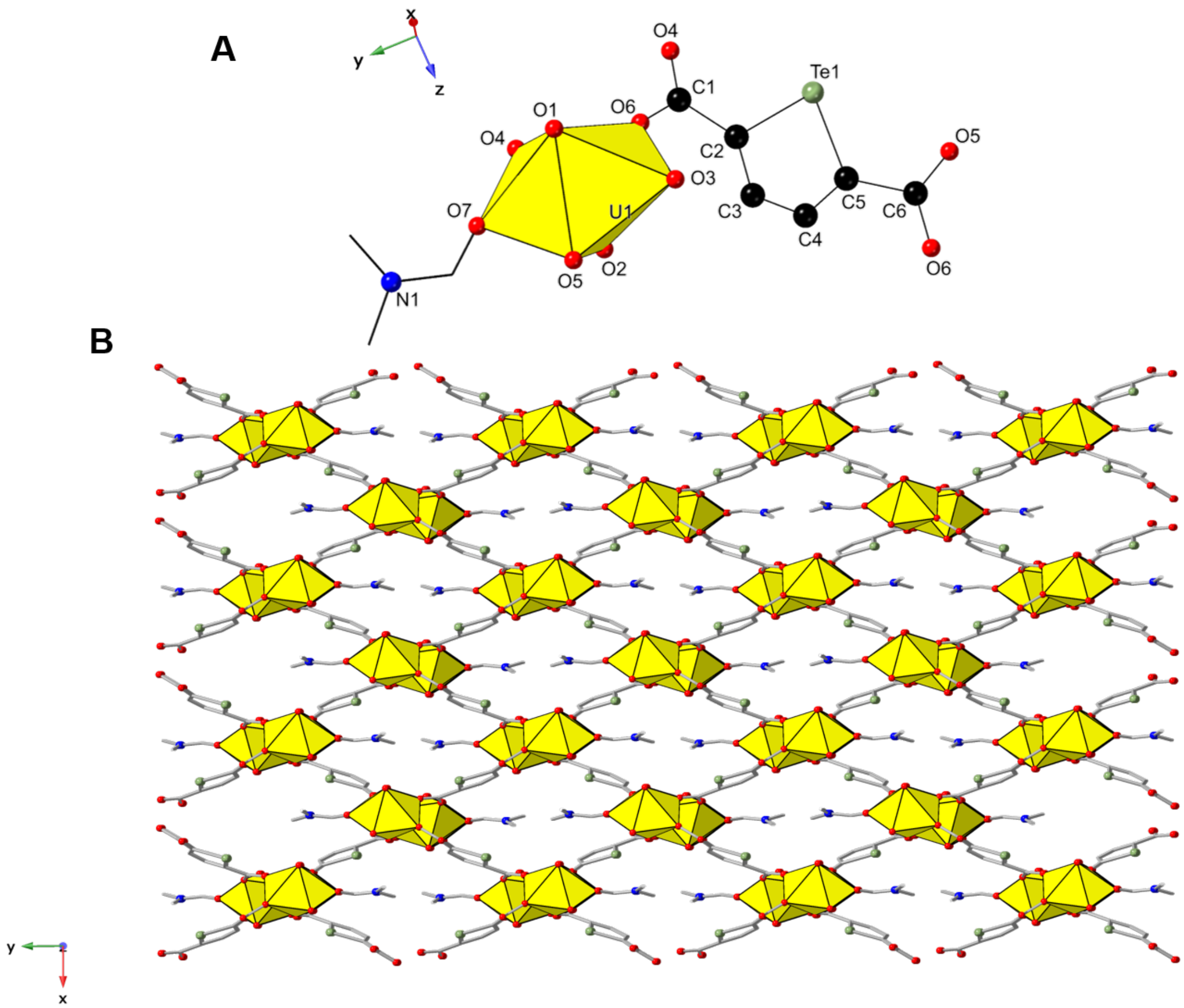

Figure S7. (a) Asymmetric unit of $[UO_2(C_6H_2O_4Te)(C_3H_7NO)]_n$ and **(b)** three-dimensional MOF structure of compound **2** viewed in the *xy*-plane. U atoms are depicted as yellow polyhedra, C atoms as black wireframes, and N, O, and Te atoms are depicted as blue, red, and green spheres, respectively. All H atoms have been omitted for clarity.

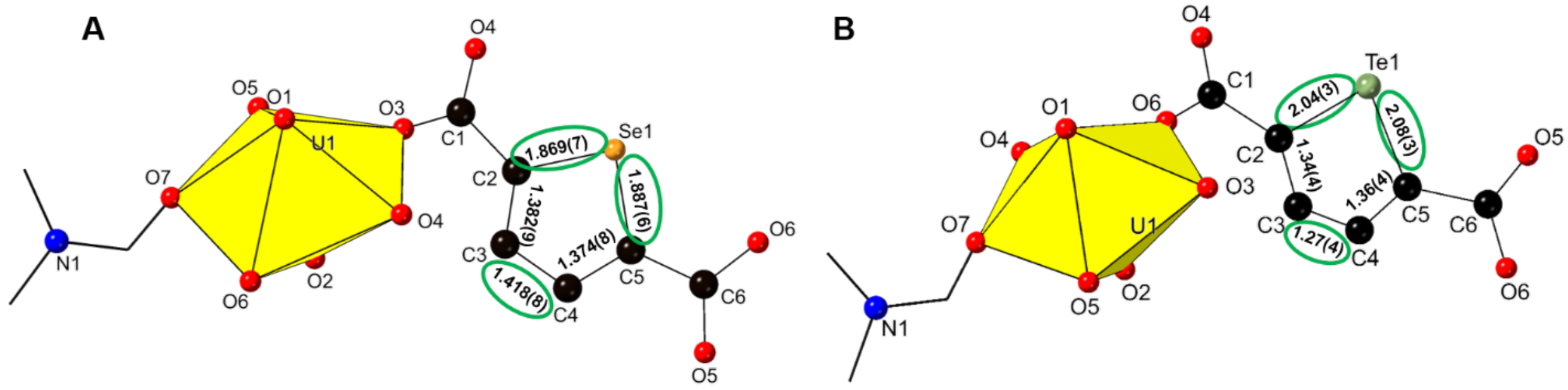

Figure S8. Bond length comparison between **(a)** the selenophene ring in compound **1** and **(b)** the tellurophene ring in compound **2**.

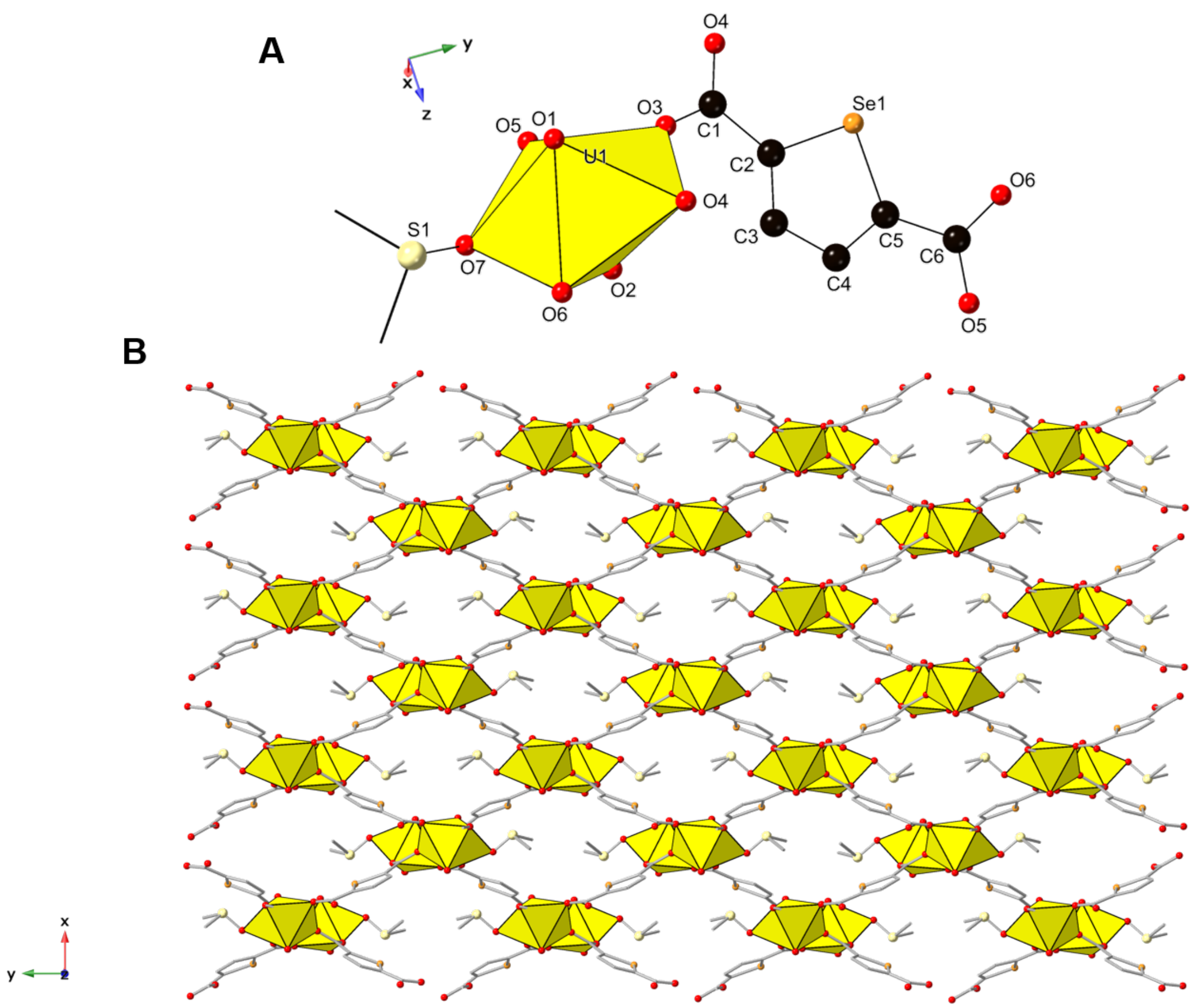

Figure S9. **(a)** Asymmetric unit of $[UO_2(C_6H_2O_4Se)(C_2H_6OS)]_n$ and **(b)** three-dimensional MOF structure of compound **3** viewed in the *xy*-plane. S atoms are depicted as light-yellow spheres. All H atoms have been omitted for clarity.

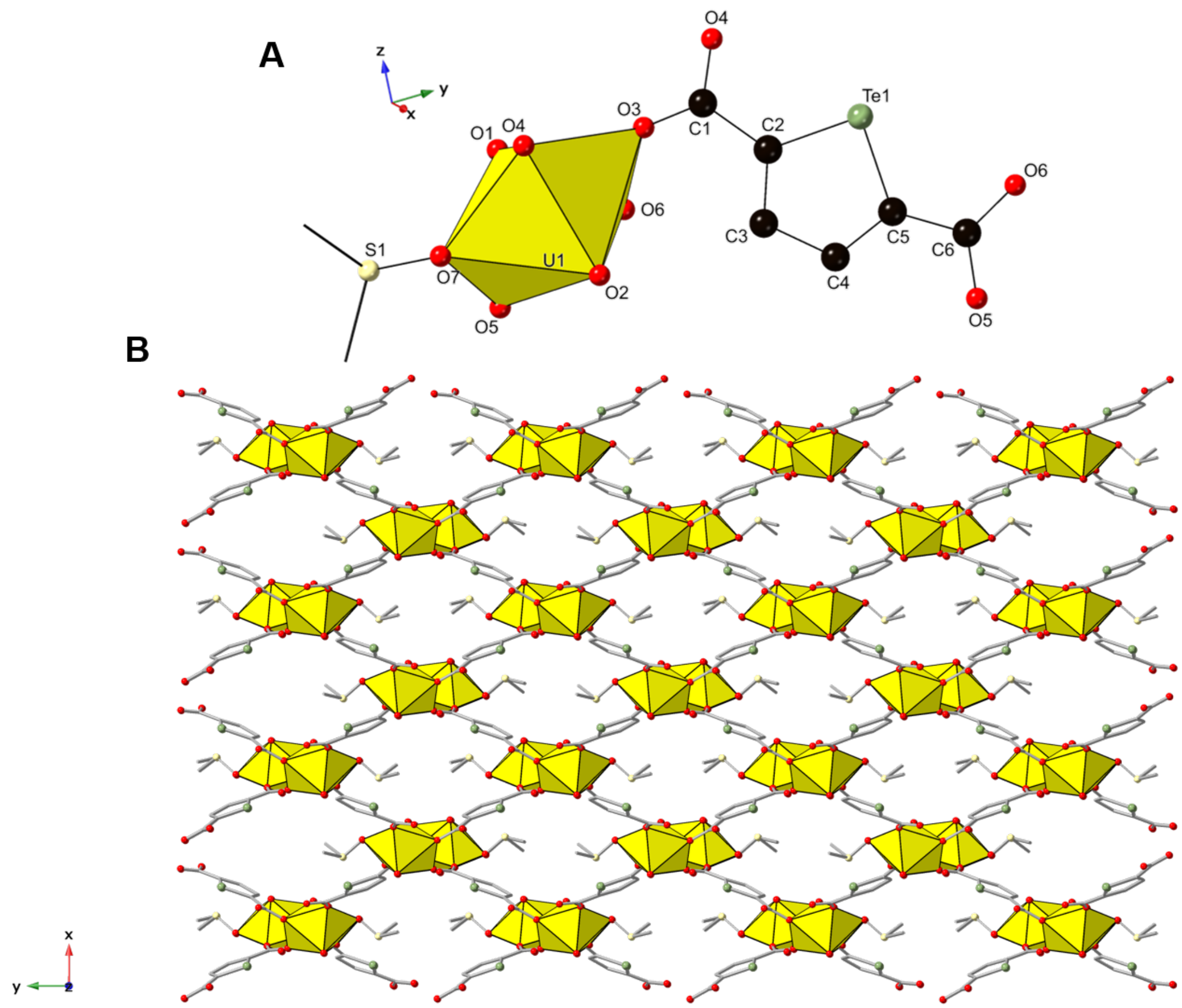

Figure S10. (a) Asymmetric unit of $[UO_2(C_6H_2O_4Te)(C_2H_6OS)]_n$ and **(b)** three-dimensional MOF structure of compound **4** viewed in the *xy*-plane. All H atoms have been omitted for clarity.

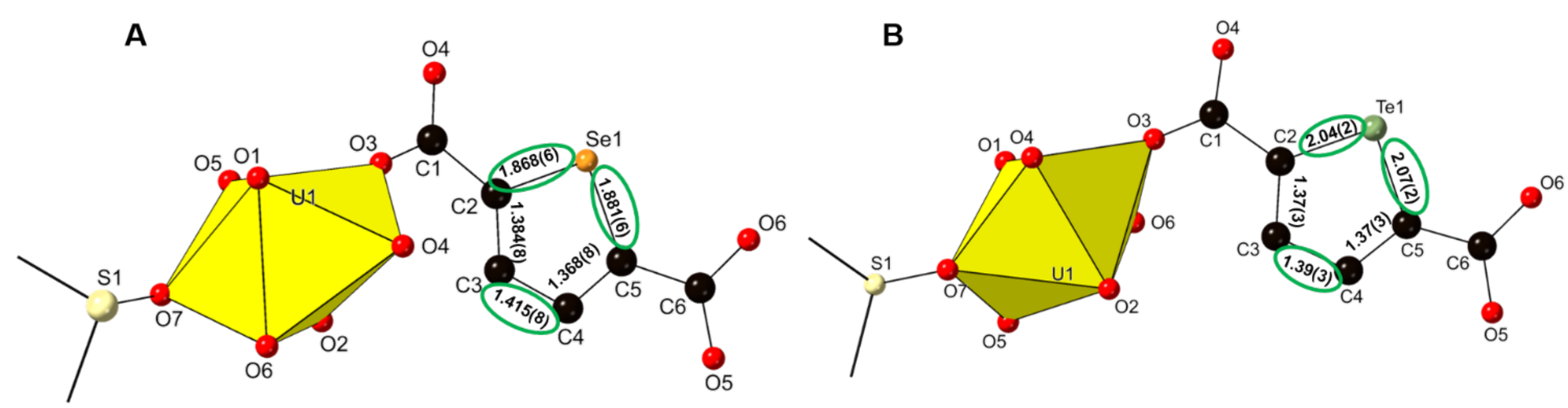

Figure S11. Bond length comparison between **(a)** the selenophene ring in compound **3** and **(b)** the tellurophene ring in compound **4**.

Compound **7**, $[\{UO_2(C_6H_2O_4Se)_{1.5}\}^{-}{}_4 \cdot 2(H_2O) \cdot 4(C_2H_6N)^{+}]_n$, is a minor product in the synthesis of **1** and is a 3D anionic framework that is charge balanced by dimethylacetamide (DMA^+) cations. We were able to select single crystals of **7** from the bulk product of **1** and compound **7** can be obtained in pure form by increasing the metal-to-ligand ratio to 1:2 in the reaction mixture. This compound crystallizes in the space group $Pca2_1$ with a hcb topology (**Figure S76**) and in contrast to **1**, the DMF molecules in this structure do not coordinate with the uranyl centers (**Figure S12a**). Instead, the altered reaction conditions facilitate hydrolysis of DMF to yield DMA^+, which resides in the lattice of structure to charge-balance the anionic uranyl network. In **7**, $[UO_2]^{2+}$ cations are exclusively coordinated by the bidentate carboxylate groups of the 2,5-SeDC ligand and adopt distorted hexagonal bipyramidal geometries (**Figure S12a**). For U1, the two axial U=O bonds are U1–O2: 1.758(5) Å and U1–O1: 1.765(5) Å with an O1–U1–O2 angle of 177.3(2)° indicating nearly linear coordination, while the six equatorial U–O bonds are at an average distance of 2.481 Å. Similarly, U2 exhibits axial U=O bonds at U2–O3: 1.775(5) Å and U2–O4: 1.772(4) Å with an O3–U2–O4 angle of 177.7(2)° and equatorial U–O bonds with an average distance of 2.470 Å. The absence of DMF coordination represents a key structural distinction compared to compound **1**, resulting in a three-dimensional anionic network, and an evaluation of the packing of **7** reveals a lack of available porosity as the DMA counterions reside in interstitial voids of the structure. Additionally, both compounds **1** and **7** share similar Se-C bond distances within selenophene rings, ranging from 1.854(6) Å to 1.881(7) Å, and in **7**, Se1 is bound to C2 and C5 at distances of 1.854(6) Å and 1.858(6) Å, Se2 coordinates to C8 and C11 at distances of 1.860(6) Å and 1.881(7) Å, and Se3 bonds to C14 and C17 at distances of 1.856(7) Å and 1.869(7) Å. These bond distances are consistent with those observed in compound **1**, suggesting no perturbation in the chalcogenophene ring geometry upon the change in compound formation.

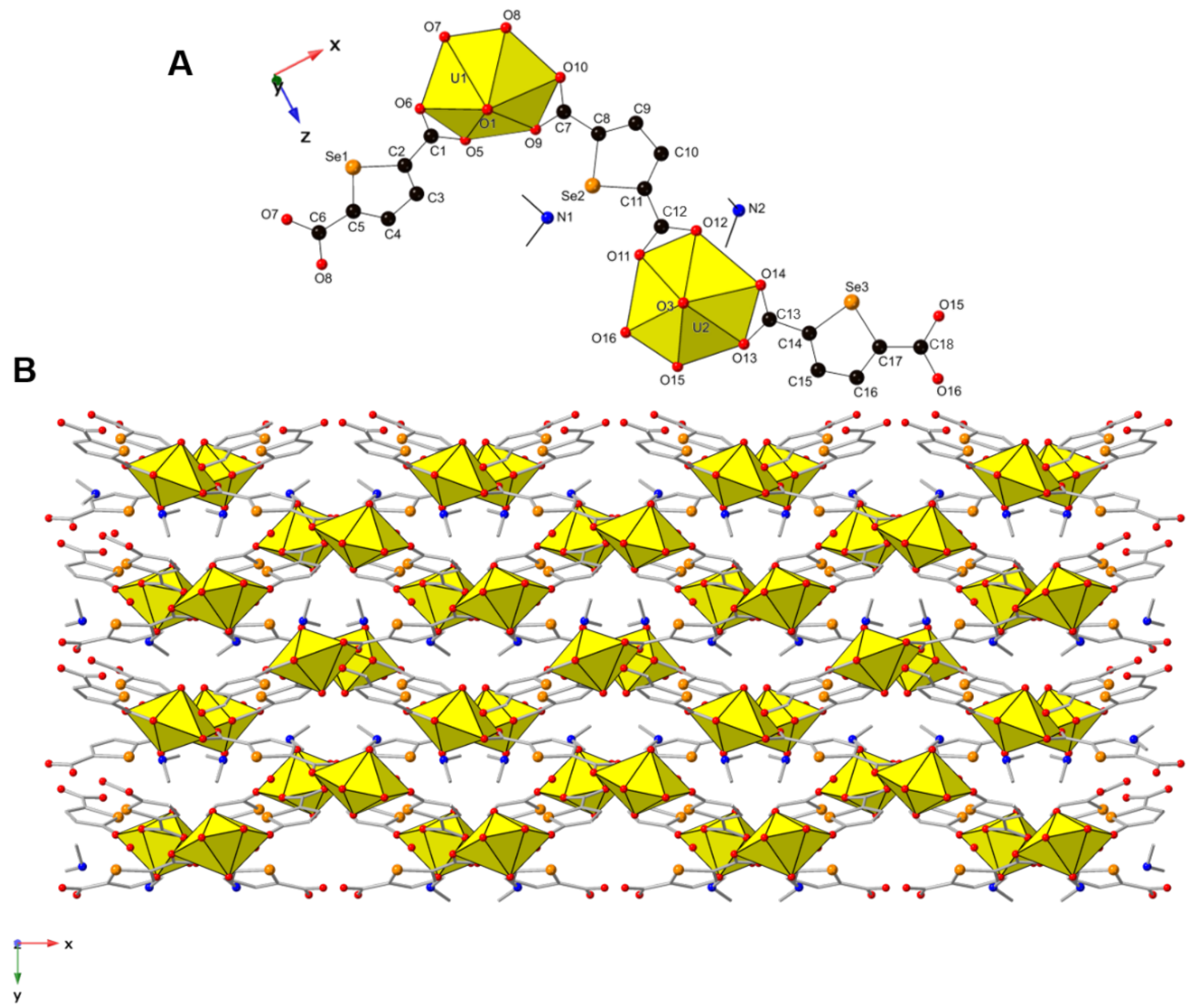

Figure S12. (a) Asymmetric unit of $[\{UO_2(C_6H_2O_4Se)_{1.5}\}^-_4 \bullet 2(H_2O) \bullet 4(C_2H_6N)^+]_n$ and **(b)** three-dimensional anionic framework structure of compound **7** viewed in the *xy*-plane. All H atoms have been omitted for clarity.

Mercury Pore Analysis was also performed for compound **7** to evaluate the influence of topology and packing on void space and pore accessibility (**Table S1**). Compound **7** exhibits a substantially larger unit-cell volume and lower framework density relative to compounds **1**-**6**, reflecting increased internal void space. The calculated geometric pore volume is significantly higher than that observed for the earlier compounds, with a corresponding increase in pore limiting and maximum pore diameters (PLD = 2.36 Å; MPD = 3.85 Å). Despite this expansion, the pore network in **7** remains restricted, exhibiting one-dimensional percolation and limited accessible surface area, indicating the presence of narrow, poorly interconnected channels rather than extended open pores. These results demonstrate that while the topology adopted by compound **7** promotes increased framework openness relative to compounds **1**-**6**, the resulting structure remains ultramicroporous with constrained pore accessibility.

Compound **8**, $[\{UO_2(C_6H_2O_4Te)_{1.5}\}^-_2 \bullet 2.5(H_2O) \bullet 2(C_2H_6N)^+]_n$, is a 3D anionic framework that is charge balanced by DMA^+ cations, which is minor product in the synthesis of **2** that can be obtained in pure form by increasing the metal-to-ligand ratio in the reaction mixture (**Figure S13**). This compound crystallizes in the space group *C*2/*c* and features uranyl centers coordinated by two axial oxo groups (O1, O2) and six equatorial carboxylate oxygen atoms (O3, O4, O5, O6, O7, O8) from 2,5-TeDC ligands resulting in a distorted hexagonal bipyramidal geometry (**Figure**

S13a). In contrast to compound **2**, DMF molecules are not present, but rather interstitial voids contain DMA molecules from DMF hydrolysis, consistent with the observations for **7**. The structural divergence from compound **2** therefore reflects not only the absence of neutral solvent coordination, but also differences in equatorial donor saturation, ligand binding geometry, and framework packing. As a result, compound **8** adopts a distinct topology relative to compound **7**, exhibiting a 2C1 net rather than an hcb topology (**Figure S77**). The U–O bond distances in **8** range from 1.743(15) Å to 2.517(12) Å, with axial U=O bonds U1–O1: 1.743(15) Å and U1–O2: 1.744(13) Å with an O2–U1–O1 angle of 178.6(6)°, and equatorial U–O bonds in the range of 2.433(14) Å to 2.517(12) Å, with an overall average of 2.475 Å that is elongated relative to those in **2**. This elongation is consistent with differences in equatorial donor saturation and ligand binding geometry imposed by the distinct framework topology of **8**. The tellurophene rings in compound **8** display Te–C bond distances of 2.054(17) Å (Te1–C2) and 2.049(19) Å (Te1–C5), consistent with the elongation observed in compound **2** and longer than in compound **4**. Internal C–C distances within the tellurophene ring range from 1.35 to 1.44 Å, which contrasts with the contraction observed in compound **2**. Te2 also exhibits similar elongation, with Te–C bond lengths of 2.03(5) Å (Te2–C8) and 2.02(5) Å (Te2–C9). Structural differences in compound **8**, compared to compound **2**, are a result of changes in the coordination environment that arise from the stoichiometric change and the presence of DMA^+ molecules in interstitial voids. Moreover, in compound **8**, the replacement of Se with Te also results in notable changes to the overall packing and connectivity, generating distinct solvent-accessible voids as can be seen in **Figure S13b** that are absent in compound **7**. Although both frameworks lack coordinated solvent molecules, the chalcogen substitution alone is sufficient to induce a topological shift, change in porosity, and to modify the spatial arrangement of the network. These observations underscore the structural tunability of uranyl hybrid materials where subtle variations in linker identity can lead to substantial changes in the framework architecture and porosity.

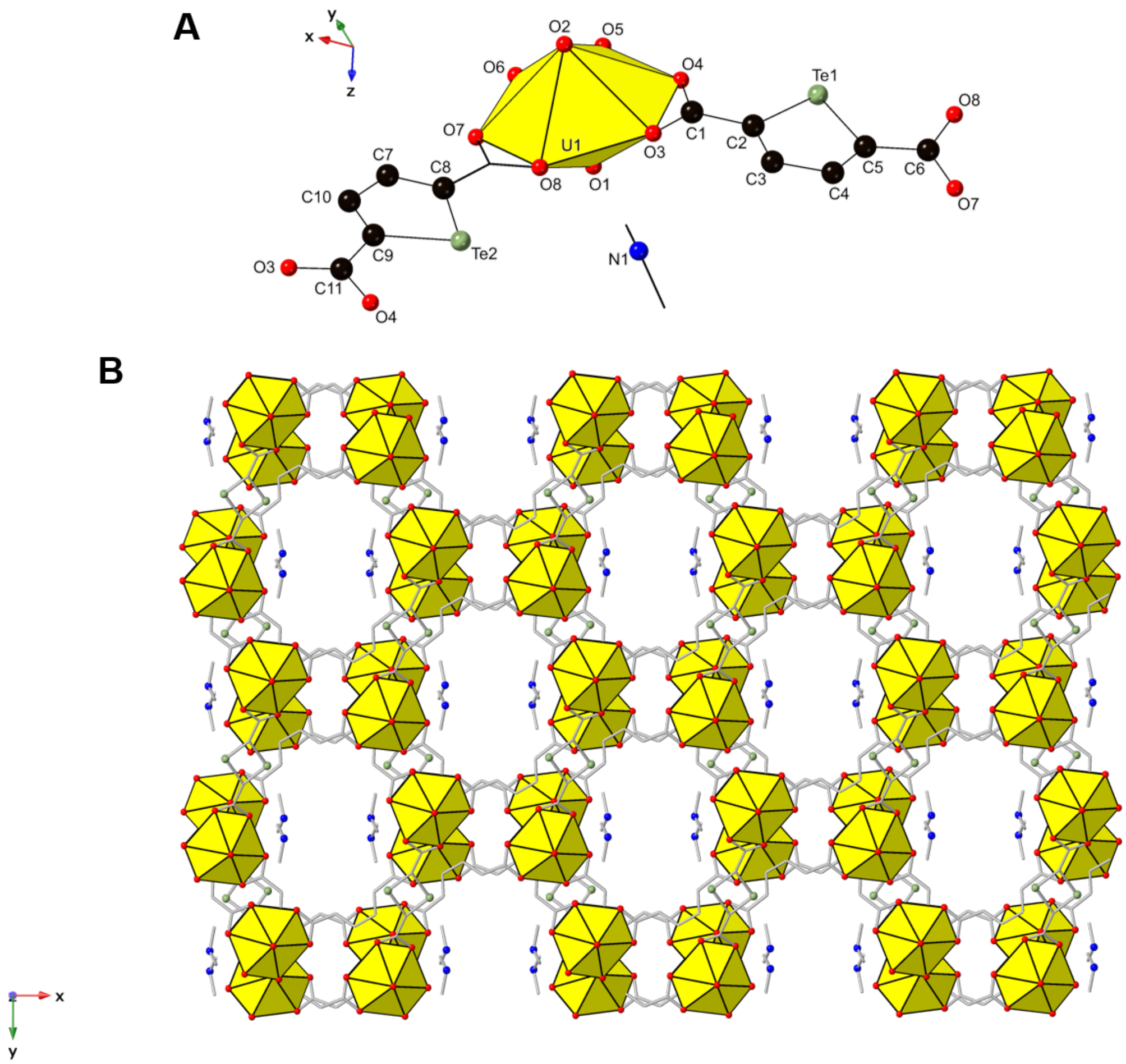

Figure S13. (a) Asymmetric unit of $[\{UO_2(C_6H_2O_4Te)_{1.5}\}^-{}_2{\bullet}2.5(H_2O){\bullet}2(C_2H_6N)^+]_n$ and **(b)** three-dimensional anionic framework structure of compound **8** viewed in the *xy*-plane. All H atoms have been omitted for clarity.

According to Mercury Pore Analysis (**Table S1**), compound **8** exhibits the largest unit-cell volume and one of the lowest framework densities within the series, indicative of increased internal void space relative to the other compounds. This is reflected in expanded pore dimensions, with a pore limiting diameter of 3.38 Å and a maximum pore diameter of 4.39 Å, representing the largest pore characteristics observed across compounds **1**-**8**. Unlike **1**-**7**, compound **8** displays a small network-accessible surface area, consistent with the presence of one-dimensional percolated channels. Nevertheless, the accessible surface area remains limited, and the pore network is constrained by narrow apertures and restricted connectivity. These results indicate that although the framework topology adopted by compound **8** promotes increased openness relative to the rest of the series, the material still remains ultramicroporous with modest pore accessibility.

Refinement Details

Compound 1:

Compound **1** was refined against twinned data. Initial analysis using CELL_NOW identified a multi-domain twin; however, the final refinement was performed using a two-component inversion twin model. Absorption corrections for the twinned data were applied using TWINABS-2012/1. The structure was refined as a two-component inversion twin with refined BASF scale factors of 0.53855 and 0.46145. All non-hydrogen atoms were refined anisotropically. To improve anisotropic displacement parameter behavior, rigid-bond restraints (RIGU; σ = 0.001) were applied to select selenophene ring atoms (C1, C2, C3, and Se1). Hydrogen atoms bound to aromatic and amide carbon atoms (C3, C4, and C7) were placed in calculated positions and refined using riding models, while methyl groups (C8 and C9) were treated as idealized rotating groups using AFIX commands.

Compound 2:

A spherical absorption correction was applied, and owing to the limited crystal quality, the spherical absorption correction parameter (μ) was fixed at 1.0 to stabilize the correction during integration. The structure was refined as a two-component inversion twin using TWIN/BASF with refined twin scale factors of 0.57947 and 0.42053. All non-hydrogen atoms were refined anisotropically. The coordinated DMF molecule exhibits positional disorder and was modeled over two sites with linked occupancies. To maintain chemically reasonable geometries and displacement parameters for the disordered DMF and the 2,5-tellurophene dicarboxylate moieties, a combination of distance restraints (SADI), displacement parameter similarity restraints (SIMU), and RIGU restraints were applied. Corresponding bond distances between disorder components were restrained to be equal, and Uiso/Uaniso similarity restraints were applied to atoms within 2 Å. Hydrogen atoms bound to aromatic and amide carbon atoms (C3, C4, and C7) were placed in calculated positions and refined using riding models, while methyl groups (C8 and C9) were treated as idealized rotating groups using AFIX commands.

Compound 3:

Compound **3** was refined and treated as a two-component inversion twin with refined twin scale factors of 0.84048 and 0.15952. Twinning was modeled using an inversion twin model. All non-hydrogen atoms were refined anisotropically, and no positional disorder or additional geometric or displacement restraints were required. Hydrogen atoms bound to aromatic and amide carbon atoms (C3, C4) were placed in calculated positions and refined using riding models. Methyl groups associated with the coordinated DMSO (C7 and C8) molecule were refined as rotating groups using AFIX commands.

Compound 4:

Compound **4** was refined as a two-component inversion twin with refined BASF scale factors of 0.80554 and 0.19446. Twinning was accounted for during refinement using an inversion twin model. All non-hydrogen atoms were refined anisotropically. No ligand or solvent disorder was modeled. Hydrogen atoms bound to aromatic carbon atoms (C3 and C4) were placed in calculated

positions and refined using riding models, while methyl groups of the coordinated DMSO molecule (C7 and C8) were treated as rotating groups using AFIX commands.

Compound 5:

Compound **5** was refined as a two-component inversion twin, with refined twin scale factors of 0.8363 and 0.1637. All non-hydrogen atoms were refined anisotropically. To maintain reasonable anisotropic displacement parameters for both selenophene moieties, RIGU ($\sigma = 0.001$) restraints were applied independently to each aromatic ring. Hydrogen atoms bound to aromatic carbon atoms were placed in calculated positions and refined using riding models. Hydrogen atoms associated with water molecules could not be reliably located and were therefore not included in the refinement. No solvent disorder was modeled.

Compound 6:

Compound **6** was refined as a two-component inversion twin, with refined twin scale factors of 0.62879 and 0.37121. All non-hydrogen atoms were refined anisotropically. The 2,5-tellurophene dicarboxylate ligand exhibits positional disorder over two sites and was modeled using PART commands with refined occupancies of approximately 0.75 and 0.25 for the major and minor components, respectively. To maintain chemically reasonable geometry and anisotropic displacement parameters for the disordered 2,5-TeDC ligand, a combination of SADI, SIMU, and RIGU restraints were applied. SADI restraints were used to equate corresponding Te–C and C–C bond distances between disordered components, with tighter σ values (0.02 Å) applied to primary bonds and looser restraints (0.04 Å) applied to secondary distances. SIMU restraints were applied to atoms within 2 Å to maintain comparable displacement parameters across the disordered fragments, and RIGU ($\sigma = 0.001$) restraints were applied to the tellurophene atoms and adjacent framework atoms to stabilize their anisotropic refinement. Hydrogen atoms bound to aromatic carbon atoms (C3, C4 and their disordered counterparts C3A, C4A) were placed in calculated positions and refined using a riding model, while hydrogen atoms associated with the lattice water molecule (O7) were located from difference Fourier maps and then geometrically constrained.

Compound 7:

A spherical absorption correction was applied, with the absorption coefficient (μ) set to 3.0 to provide a stable correction appropriate for the limited crystal quality of the sample. Twinning was accounted for using a two-component inversion twin model. All non-hydrogen atoms were refined anisotropically, and no additional geometric or displacement restraints were required. Hydrogen atoms bound to carbon were placed in calculated positions and refined using riding models, with methyl groups treated as idealized rotating groups. Hydrogen atoms associated with lattice water molecules were located from difference Fourier maps and then geometrically constrained.

Compound 8:

Compound **8** was refined with positional disorder. One tellurophene moiety exhibits positional disorder and was modeled over two positions with fixed occupancies of 0.50/0.50. To maintain chemically reasonable geometry for the disordered tellurophene fragment, planarity restraints (FLAT, $\sigma = 0.1$) were applied to atoms Te2, C1, C10, C11, and C12. RIGU restraints were applied to the disordered tellurophene atoms (Te2, C1, C9-C12) and adjacent coordinating atoms (O3, O4), as well as to the tertiary amine fragment (N1, C13, C14), to stabilize anisotropic displacement parameters. All non-hydrogen atoms were refined anisotropically. Hydrogen atoms bound to carbon atoms were placed in calculated positions and refined using riding models, with aromatic C–H and methyl groups treated as rotating groups where applicable; no hydrogen atoms were refined on disordered sites beyond those included via riding constraints. Residual solvent electron density was treated using a solvent mask to account for diffuse solvent contributions that were not explicitly modeled. Despite the limited crystal quality, the final refinement converged with acceptable agreement indices ($R_1 = 0.0762$, $wR_2 = 0.1702$ for all data), supporting the adopted disorder and restraint model.

Crystallographic Details

Table S2. Crystallographic parameters for compounds **1-8**

**Compound**	**1**	**2**	**3**	**4**
Formula	$[(UO_2)(C_6H_2O_4Se)(C_3H_7NO)]$	$[(UO_2)(C_6H_2O_4Te)(C_3H_7NO)]$	$[(UO_2)(C_6H_2O_4Se)(C_2H_6OS)]$	$[(UO_2)(C_6H_2O_4Te)(C_2H_6OS)]$
Formula Weight	560.16	608.80	565.19	613.83
Temp (K)	100(2)	100(2)	100(2)	100(2)
Space group	$Pna2_1$	$Pna2_1$	$Pna2_1$	$Pna2_1$
a (Å)	8.935(5)	8.9857(7)	9.1956(6)	9.311(3)
b (Å)	15.224(5)	15.1810(12)	14.6492(6)	14.4555(18)
c (Å)	9.460(5)	9.5505(7)	9.5006(4)	9.679(4)
α (°)	90	90	90	90
β (°)	90	90	90	90
γ (°)	90	90	90	90
V ($Å^3$)	1286.8(10)	1302.80(17)	1279.81(9)	1302.7(7)
Z	4	4	4	4
Density (g/cm^3)	2.891	3.104	2.933	3.130
μ (mm^{-1})	15.469	14.681	15.711	14.836
λ (Mo Kα; Å)	0.71073	0.71073	0.71073	0.71073

R_{int}	0.0795	0.1269	0.0522	0.0530
R_1	0.0184	0.0485	0.0231	0.0476
wR_2	0.0445	0.0942	0.0463	0.1204
GooF	1.086	1.095	1.038	1.133
**Compound**	**5**	**6**	**7**	**8**
Formula	**$[(UO_2)_2(C_6H_2O_4Se)_2]_2$ •$2H_2O$**	**$[(UO_2)(C_6H_2O_4Te)]$ •H_2O**	**$[\{(UO_2)(C_6H_2O_4Se)_{1.5}]^-\}_4$ •$2H_2O$ •$4\{(C_2H_6N)\}^+$**	**$[\{(UO_2)(C_6H_2O_4Te)_{1.5}]^-\}_2$ •$2H_2O$ •$2\{(C_2H_6N)\}^+$**
Formula Weight	990.13	553.72	2594.68	1527.58
Temp (K)	100(2)	100(2)	100(2)	100(2)
Space group	*Cc*	*Pna*2$_1$	*Pca*2$_1$	*C*2/*c*
a/Å	6.2528(5)	17.9878(13)	14.3166(8)	22.5318(15)
b/Å	37.546(3)	7.2189(6)	12.5112(7)	15.8860(10)
c/Å	8.3837(7)	7.5082(4)	18.0788(11)	12.3130(9)
α (°)	90	90	90	90
β (°)	95.069(3)	90	90	120.481(2)
γ (°)	90	90	90	90
V (Å^3)	1960.5(3)	974.96(12)	3238.2(3)	3798.2(5)
Z	4	4	2	4
Density (g/cm^3)	3.355	3.772	2.661	2.671
μ (mm^{-1})	20.277	19.594	13.438	10.850
λ (Mo Kα; Å)	0.71073	0.71073	0.71073	0.71073
R_{int}	0.0949	0.0894	0.0996	0.0944
R_1	0.0406	0.0390	0.0242	0.0762
wR_2	0.0785	0.0965	0.0536	0.1702
GooF	1.072	1.036	1.030	1.093

Table S3. Select bond lengths (Å) and angles (°) for compound **1**.

U1-O1	1.785(5)
U1-O2	1.763(5)
U1-O7	2.399(4)
U1-O3	2.363(4)
U1-O6[3]	2.447(4)
U1-O5[2]	2.375(4)
U1-O4[3]	2.348(4)
Se1-C2	1.869(7)
Se1-C5	1.887(6)
C2-C3	1.382(9)
C2-C1	1.492(8)
C4-C5	1.374(8)
C4-C3	1.418(8)
C5-C6	1.493(8)
Se1–C2–C1	123.8(5)
Se1–C5–C6	122.2(4)
O4–C1–C2–Se1	7.3(8)
O6–C6–C5–Se1	18.2(8)
O2–U1–O1	178.3(2)

[1]-1/2+X,1/2-Y,-1+Z; [2]3/2-X,-1/2+Y,-1/2+Z; [3]1-X,-Y,-1/2+Z

Table S4. Select bond lengths (Å) and angles (°) for compound **2**.

U1-O6[1]	2.333(17)
U1-O1	1.766(18)
U1-O2	1.755(18)
U1-O4[2]	2.353(17)
U1-O7	2.40(2)
U1-O3	2.341(18)
U1-O5[3]	2.401(16)
Te1-C2	2.04(3)
Te1-C5	2.08(3)
C2-C3	1.34(4)
C2-C1	1.53(4)
C4-C3	1.27(4)
C4-C5	1.36(4)
C5-C6	1.51(4)
Te1–C2–C1	121(2)
Te1–C5–C6	118.7(16)
O3–C1–C2–Te1	2(4)
O5–C6–C5–Te1	–21(4)
O2–U1–O1	177.9(9)

[1]1/2+X,3/2-Y,+Z; [2]1-X,1-Y,-1/2+Z; [3]1/2-X,-1/2+Y,1/2+Z

Table S5. Select bond lengths (Å) and angles (°) for compound **3**.

U1-O1	1.761(4)
U1-O5[1]	2.387(4)
U1-O4[3]	2.344(4)
U1-O2	1.776(4)
U1-O3	2.328(4)
U1-O6[2]	2.433(4)
U1-O7	2.396(4)
Se1-C5	1.881(6)
Se1-C2	1.868(6)
C5-C4	1.368(8)
C5-C6	1.476(8)
C2-C3	1.384(8)
C2-C1	1.470(8)
C3-C4	1.415(8)
Se1–C2–C1	123.1(6)
Se1–C5–C6	122.7(4)
O3–C1–C2–Se1	179.9(4)
O6–C6–C5–Se1	10.4(7)
O2–U1–O1	178.7(2)

[1]1/2+X,1/2-Y,+Z; [2]1-X,1-Y,-1/2+Z; [3]1/2-X,1/2+Y,1/2+Z

Table S6. Select bond lengths (Å) and angles (°) for compound **4**.

U1-O1	1.790(12)
U1-O2	1.786(12)
U1-O3	2.335(14)
U1-O7	2.408(12)
U1-O5[2]	2.406(12)
U1-O6[3]	2.331(14)
U1-O4[1]	2.394(14)
Te1-C2	2.04(2)
Te1-C5	2.07(2)
C4-C3	1.39(3)
C4-C5	1.37(3)
C2-C1	1.52(3)
C2-C3	1.37(3)
C5-C6	1.51(2)
Te1–C2–C1	121.8(14)
Te1–C5–C6	121.0(13)
O4–C1–C2–Te1	–165.7(13)
O5–C6–C5–Te1	23(2)
O2–U1–O1	179.0(6)

[1]1/2-X,-1/2+Y,-1/2+Z; [2]1/2+X,3/2-Y,-1+Z; [3]1-X,1-Y,-1/2+Z

Table S7. Select bond lengths (Å) and angles (°) for compound **5**.

U2-O10	2.322(13)
U2-O1[1]	2.478(13)
U2-O3	1.769(13)
U2-O5[2]	2.358(12)
U2-O8[3]	2.340(11)
U2-O4	1.748(14)
U2-O13	2.426(14)
U1-O9	2.254(13)
U1-O2	1.749(14)
U1-O6	2.309(12)
U1-O1	1.809(14)
U1-O11[4]	2.408(14)
U1-O12[4]	2.478(13)
U1-O7[5]	2.386(13)
Se2-C11	1.846(18)
Se2-C8	1.841(18)
Se1-C2	1.878(19)
Se1-C5	1.837(18)
C9-C10	1.42(3)
C9-C8	1.37(2)
C11-C10	1.37(3)
C11-C12	1.48(2)
C1-C2	1.47(2)
C3-C2	1.37(2)
C3-C4	1.40(3)
C8-C7	1.48(2)
C4-C5	1.38(2)
C6-C5	1.48(3)
C8-Se2-C11	85.8(8)
C5-Se1-C2	86.8(8)
C2–Se1–C5–C6	176.2(15)
C5–Se1–C2–C1	–178.4(15)
C5–Se1–C2–C3	–0.4(15)
O2–U1–O1	177.5(7)
O4–U2–O3	178.4(6)

[1]-1+X,+Y,+Z; [2]-1+X,+Y,-1+Z; [3]-3/2+X,-3/2-Y,-1/2+Z; [4]1+X,-1-Y,1/2+Z; [5]-1/2+X,-3/2-Y,-1/2+Z

Table S8. Select bond lengths (Å) and angles (°) for compound **6**.

U1-O1	1.793(15)
U1-O6[1]	2.340(16)
U1-O5[2]	2.389(19)
U1-O4[3]	2.381(16)
U1-O7	2.404(10)
U1-O2	1.766(16)
U1-O3	2.332(19)
Te1-C2	2.08(3)
Te1-C5	2.05(3)
C2-C3	1.36(4)
C6-C5	1.46(3)
C5-C4	1.36(4)
C3-C4	1.44(3)
C2-C7	1.46(3)
Te1–C2–C5	80.6(8)
Te1–C5–C6	125(2)
O5–C6–C5–Te1	0(5)
O6–C6–C5–Te1	179(3)
O2–U1–O1	176.7(5)

[1]1/2+X,3/2-Y,+Z; [2]1-X,1-Y,-1/2+Z; [3]3/2-X,-1/2+Y,1/2+Z

Table S9. Select bond lengths (Å) and angles (°) for compound **7**.

U1-O1	1.765(5)
U1-O6	2.502(5)
U1-O5	2.448(5)
U2-O4	1.772(4)
U1-O7[1]	2.401(5)
U1-O8[1]	2.592(5)
U1-O9	2.534(4)
U1-O10	2.438(5)
U1-O2	1.758(5)
U2-O3	1.775(5)
U2-O11	2.460(5)
U2-O12	2.463(5)
U2-O13	2.491(5)
U2-O14	2.458(5)
U2-O15[2]	2.466(4)
U2-O16[2]	2.451(5)
Se1-C2	1.854(6)
Se1-C5	1.858(6)
Se2-C8	1.860(6)
Se2-C11	1.881(7)
Se3-C14	1.856(7)

Se3-C17	1.869(7)
Se1–C2–C5	86.9(3)
Se1–C5–C6	115.7(5)
Se2–C8–C11	85.5(3)
Se2–C11–C12	121.6(5)
Se3–C16–C17	112.0(5)
Se3–C16–C17	118.4(5)
O2–U1–O1	177.3(2)
O4–U1–O3	177.7(2)

[1]2-X,1-Y,1/2+Z; [2]-X,-Y,-1/2+Z; [3]-X,-Y,1/2+Z; [4]2-X,1-Y,-1/2+Z

Table S10. Select bond lengths (Å) and angles (°) for compound **8**.

U1-O1	1.743(15)
U1-O6	2.433(14)
U1-O5	2.493(13)
U1-O4	2.472(13)
U1-O7[1]	2.463(13)
U1-O8[1]	2.517(12)
U1-O9	2.534(4)
U1-O3	2.517(12)
U1-O2	1.744(13)
Te1-C2	2.054(17)
Te1-C5	2.049(19)
Te2-C8	2.03(5)
Te2-C9	2.02(5)
Te1–C2–C4	113.1(14)
Te1–C7–C8	122.3(13)
Te2–C1–C11	117(4)
Te2–C9–C11	118(3)
O2–U1–O1	178.6(6)

[1]-1/2+X,1/2-Y,-1/2+Z

Synthetic, Refinement, and Crystallographic Details of an Additional Compound (U(VI)–2,5-FDC)

Synthetic Details

Synthesis of **U(VI)–2,5-FDC** involved adding 0.0257 g (50 µmol) $UO_2(NO_3)_2{\bullet}6H_2O$, 0.0080 g (50 µmol) 2,5-FDC, 0.8066 g H_2O, and 41 µL DMF into a 23 mL Parr autoclave and placing it into an oven at 120 °C for 48 h. Once removed, the Parr autoclave was opened after cooling overnight.

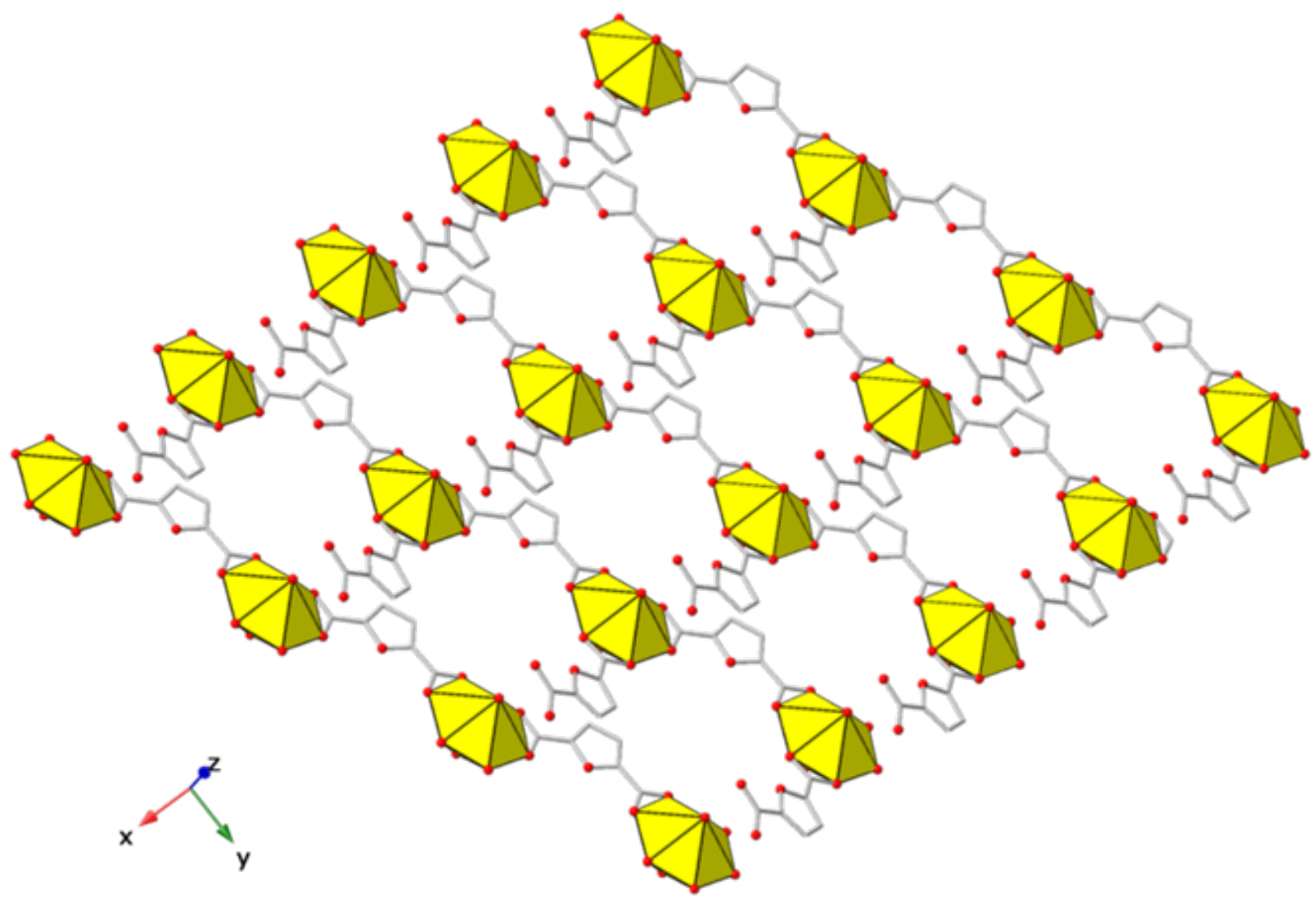

Figure S14. Two-dimensional anionic framework honeycomb-structure of **U(VI)–2,5-FDC** viewed in the *xy*-plane. All H atoms have been omitted for clarity.

Refinement Details

The U(VI)–2,5-FDC structure was refined as a two-component twin, with refined twin mass fractions of 0.4(6) and 0.6(6). All non-hydrogen atoms were refined anisotropically, and no geometric restraints were applied. The solvent cation was modeled with the associated N–H hydrogen atoms included at fixed 0.50 occupancy. Hydrogen atoms bound to carbon atoms were placed in calculated positions and refined using riding models. Additional diffuse electron density in solvent-accessible voids could not be reliably modeled as discrete atomic positions and was therefore treated using a solvent mask. The solvent mask identified four voids of approximately 147.6 Å^3 each, containing ca. 46.8-48.8 electrons per void, which were assigned as highly disordered solvent.

Crystallographic Details

**Table S11.** Crystallographic parameters for U(VI)–2,5-FDC			
Formula	**$[\{(UO_2)(C_6H_2O_5)_{1.5}\}^{-}\bullet(C_2H_8N)^{+}]_n$**		
Formula Weight	565.67	V (Å^3)	3029.9(10)
Temp (K)	100.00	Z	8
Space group	*C*222$_1$	Density (g/cm^3)	2.480
a (Å)	7.8821(16)	μ (mm^{-1})	10.768
b (Å)	17.813(4)	λ (Mo Kα; Å)	0.71073
c (Å)	21.580(2)	R_{int}	0.0419
α (°)	90	R_1	0.0300
β (°)	90	wR_2	0.0751
γ (°)	90	GooF	1.151

Table S12. Select bond lengths (Å) and angles (°) for **U(VI)–2,5-FDC**.

U1-O1	1.773(8)
U1-O6	2.451(8)
U1-O5	2.523(8)
U1-O4	2.435(8)
U1-O8^1	2.525(8)
U1-O9	2.450(7)
U1-O3	2.832(10)
U1-O2	1.778(7)
O7-C5	1.368(12)
O7-C8	1.376(11)
C7-C6	1.413(14)
C6-C5	1.331(15)
C7-C8	1.336(15)
O7–C5–C8	105.0(8)
O7–C6–C5	110.9(9)
O7–C7–C8	110.7(9)
O2–U1–O1	179.6(4)

^{1}1/2+X,1/2+Y,+Z; ^{2}-1/2+X,-1/2+Y,+Z; ^{3}+X,-Y,1-Z; ^{4}2-X,+Y,1/2-Z

Powder X-Ray Diffraction (PXRD)

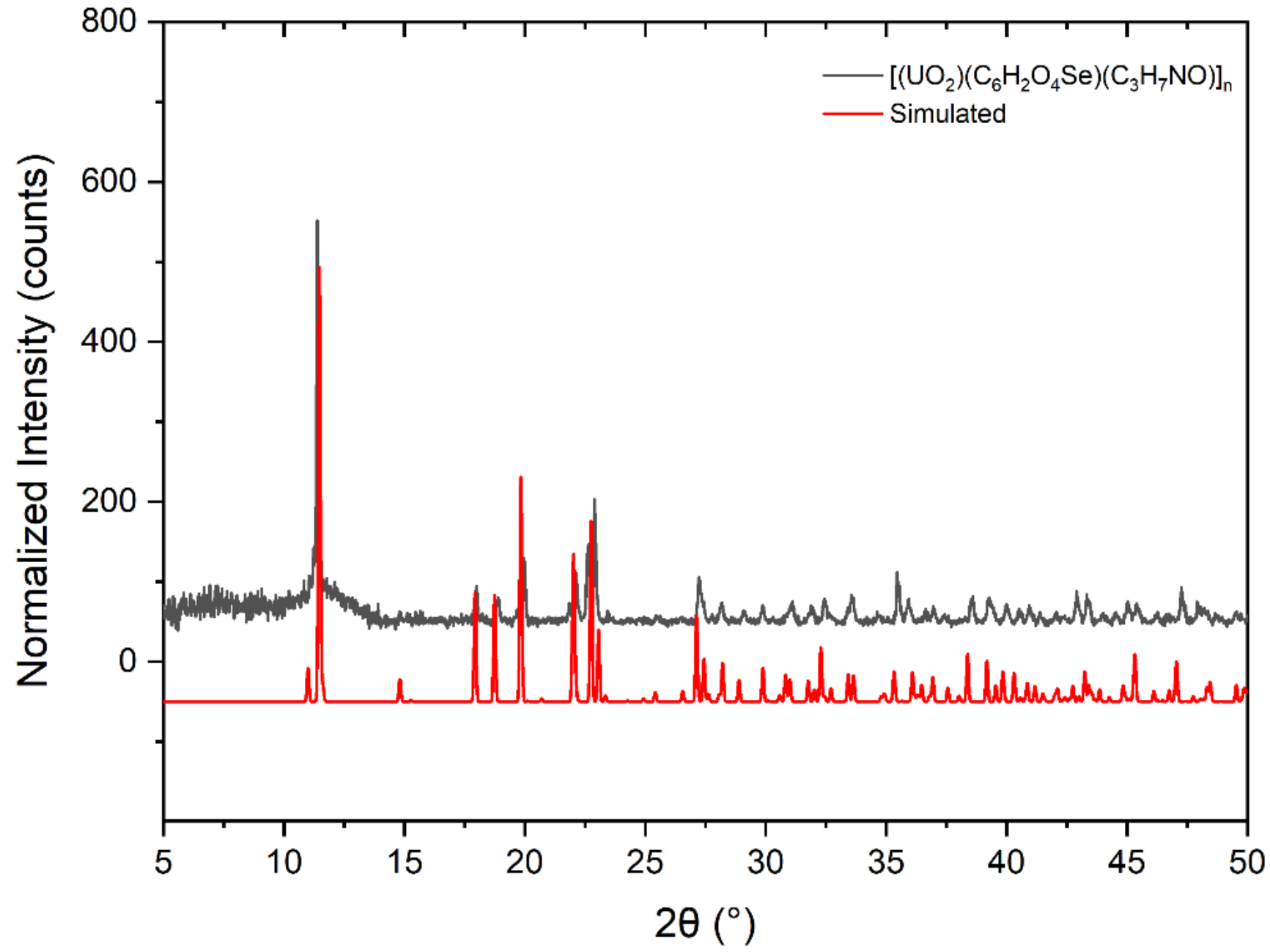

Figure S15. PXRD pattern for compound **1** (black) with calculated pattern from the SCXRD structure of **1** overlaid in red.

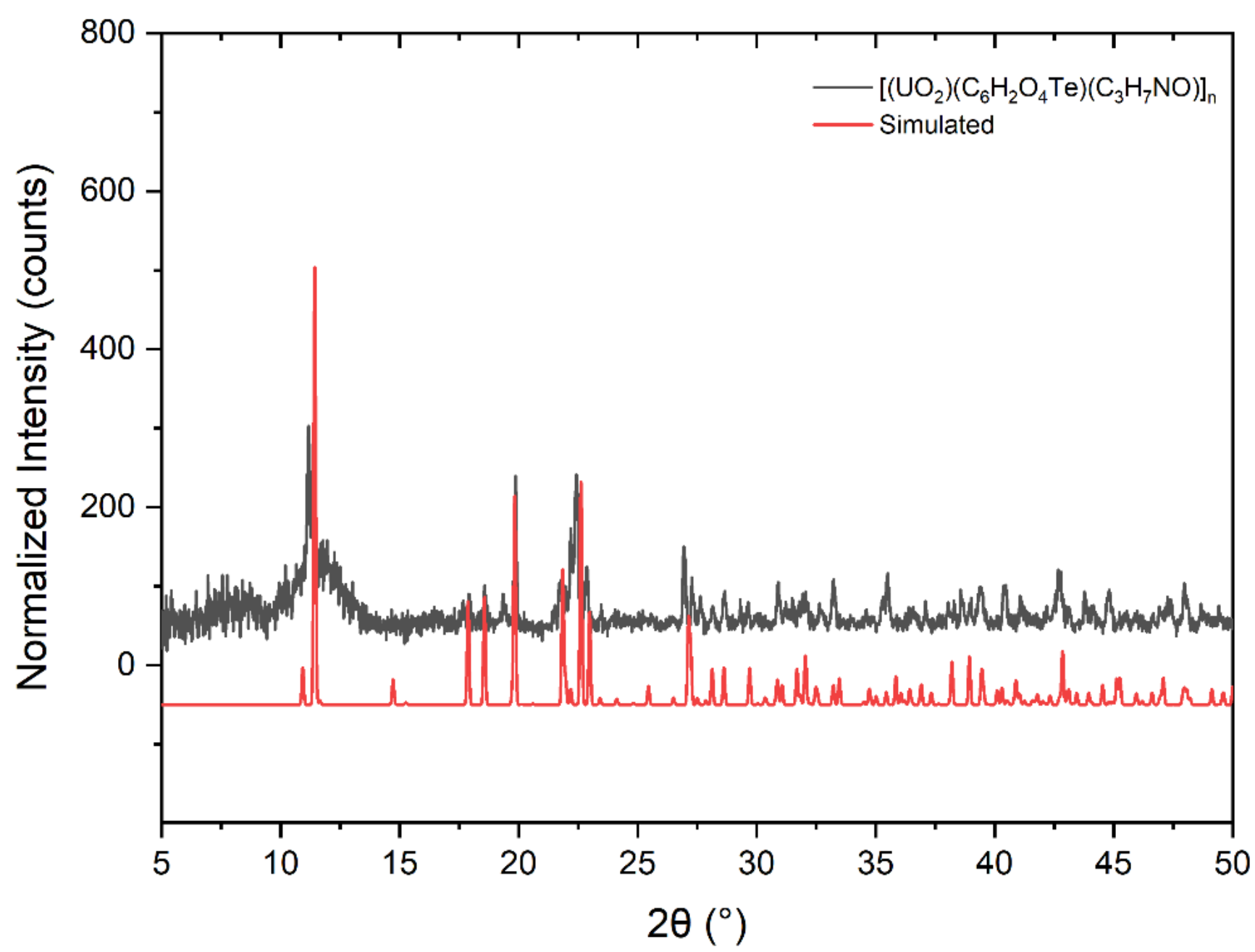

Figure S16. PXRD pattern for compound **2** (black) with calculated pattern from the SCXRD structure of **2** overlaid in red.

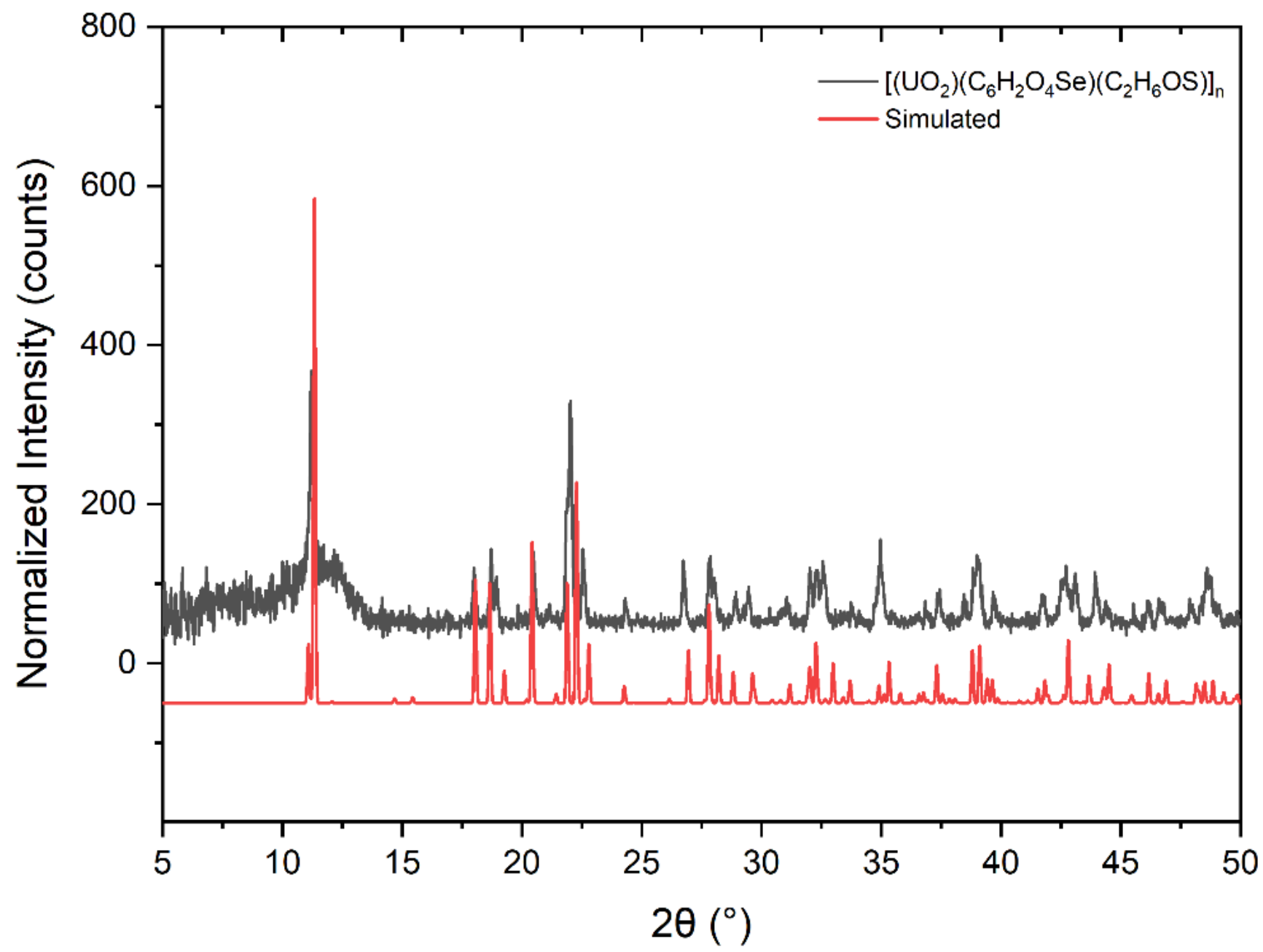

Figure S17. PXRD pattern for compound **3** (black) with calculated pattern from the SCXRD structure of **3** overlaid in red.

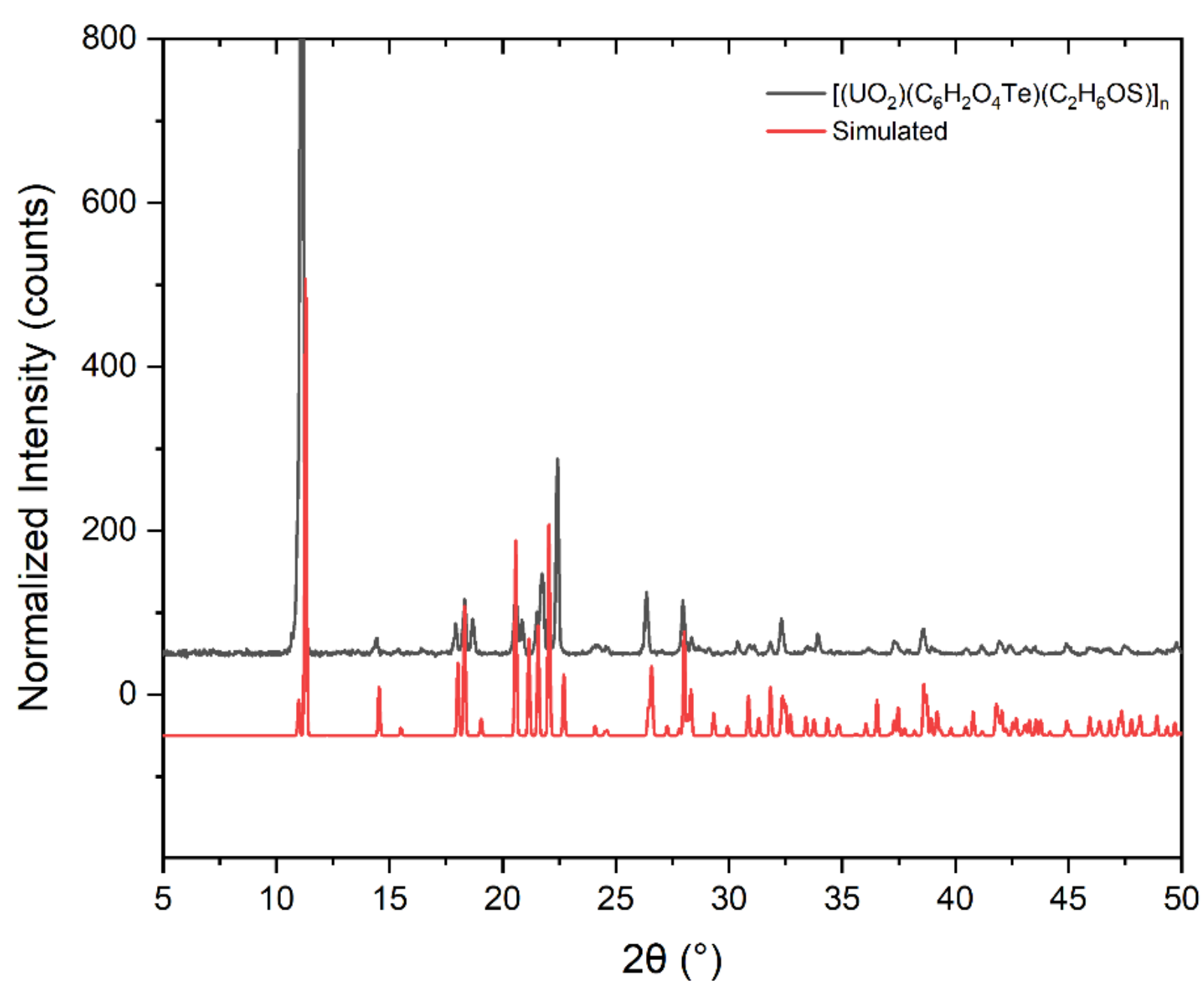

Figure S18. PXRD pattern for compound **4** (black) with calculated pattern from the SCXRD structure of **4** overlaid in red.

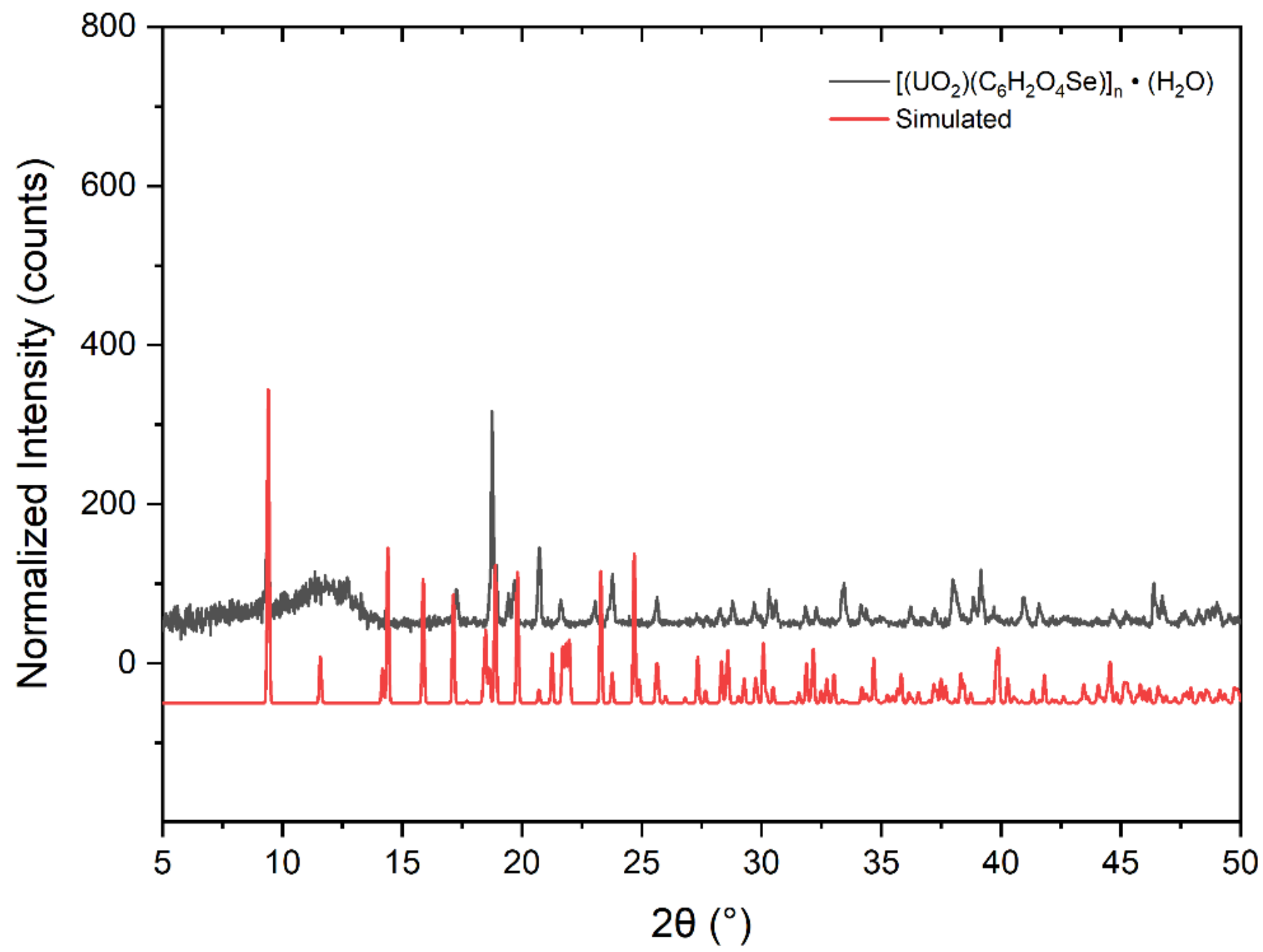

Figure S19. PXRD pattern for compound **5** (black) with calculated pattern from the SCXRD structure of **5** overlaid in red.

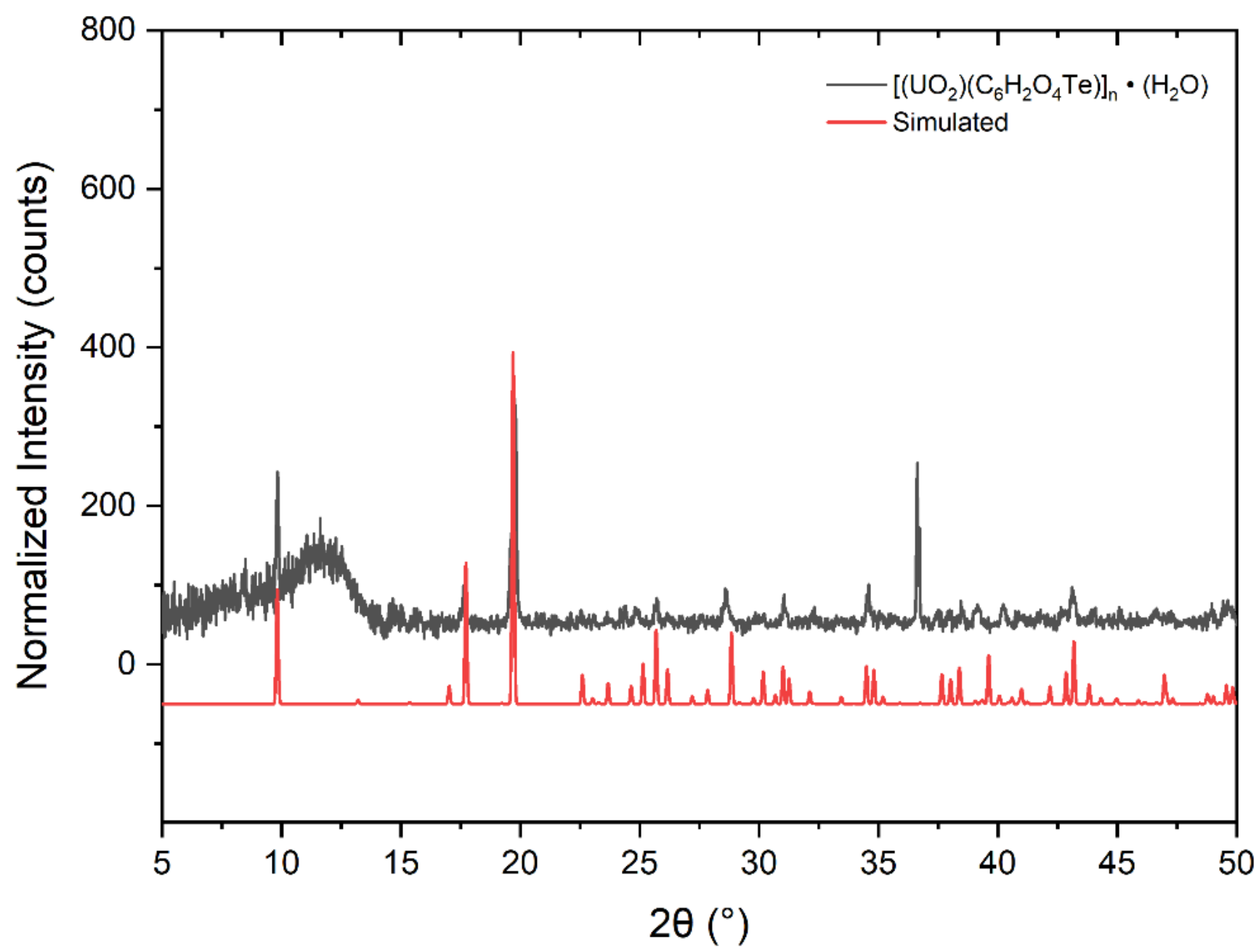

Figure S20. PXRD pattern for compound **6** (black) with calculated pattern from the SCXRD structure of **6** overlaid in red.

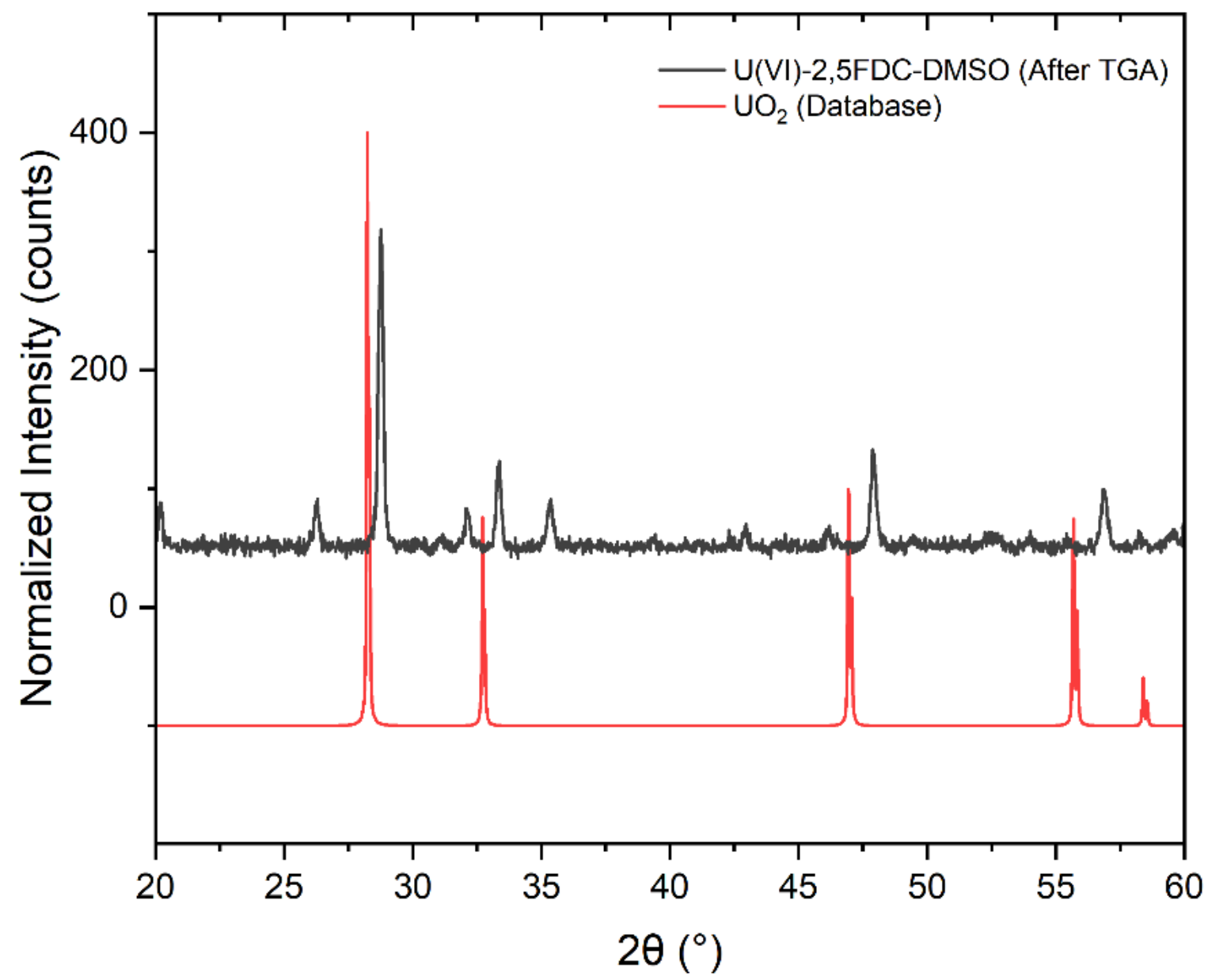

Figure S21. Post-TGA PXRD pattern of U(VI)-2,5FDC-DMSO (black) compared with known spectrum of UO_2 (PDF card no: 01-071-6416) overlaid in red.

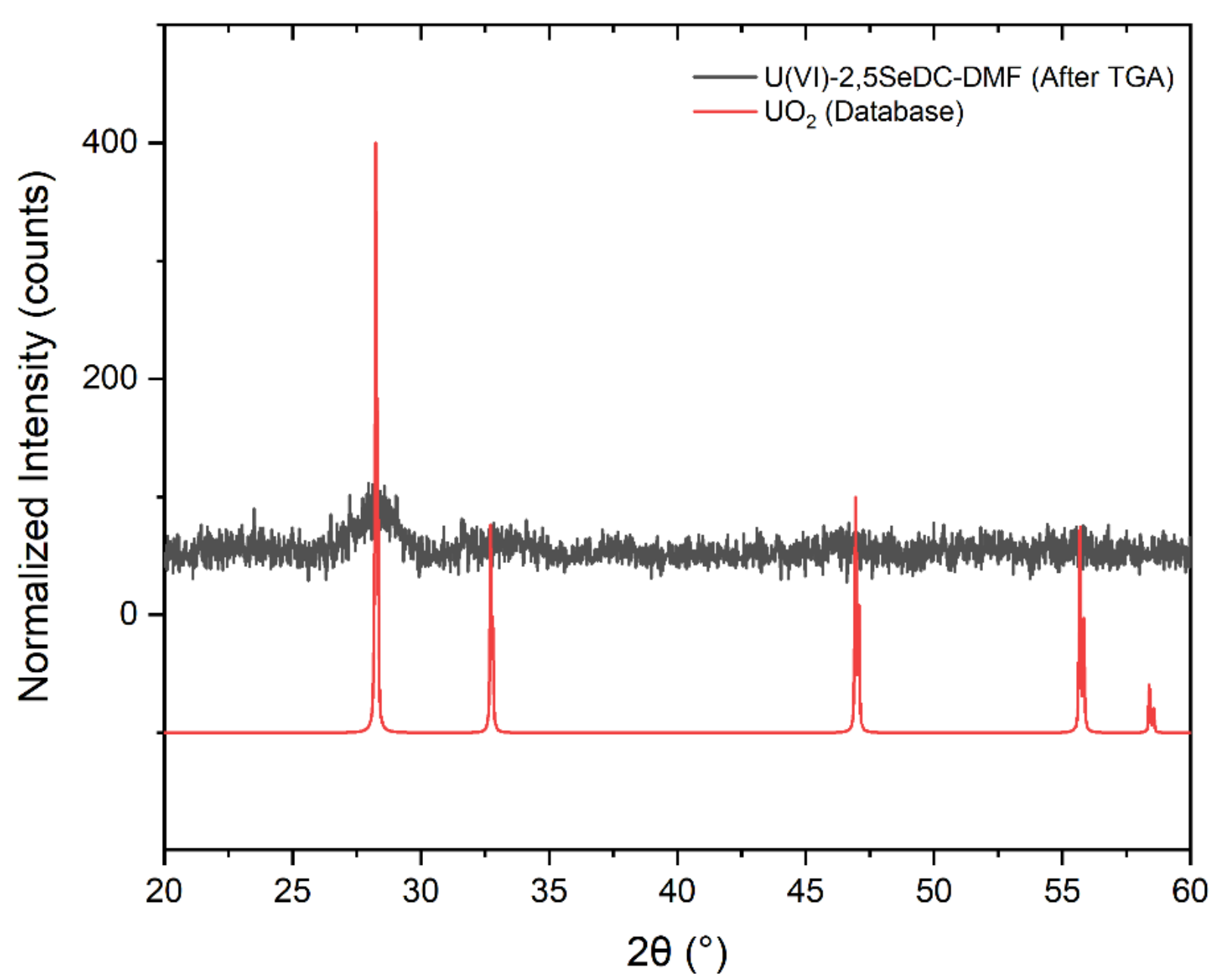

Figure S22. Post-TGA PXRD pattern of compound **1** (black) compared with known spectrum of UO_2 (PDF card no: 01-071-6416) overlaid in red.

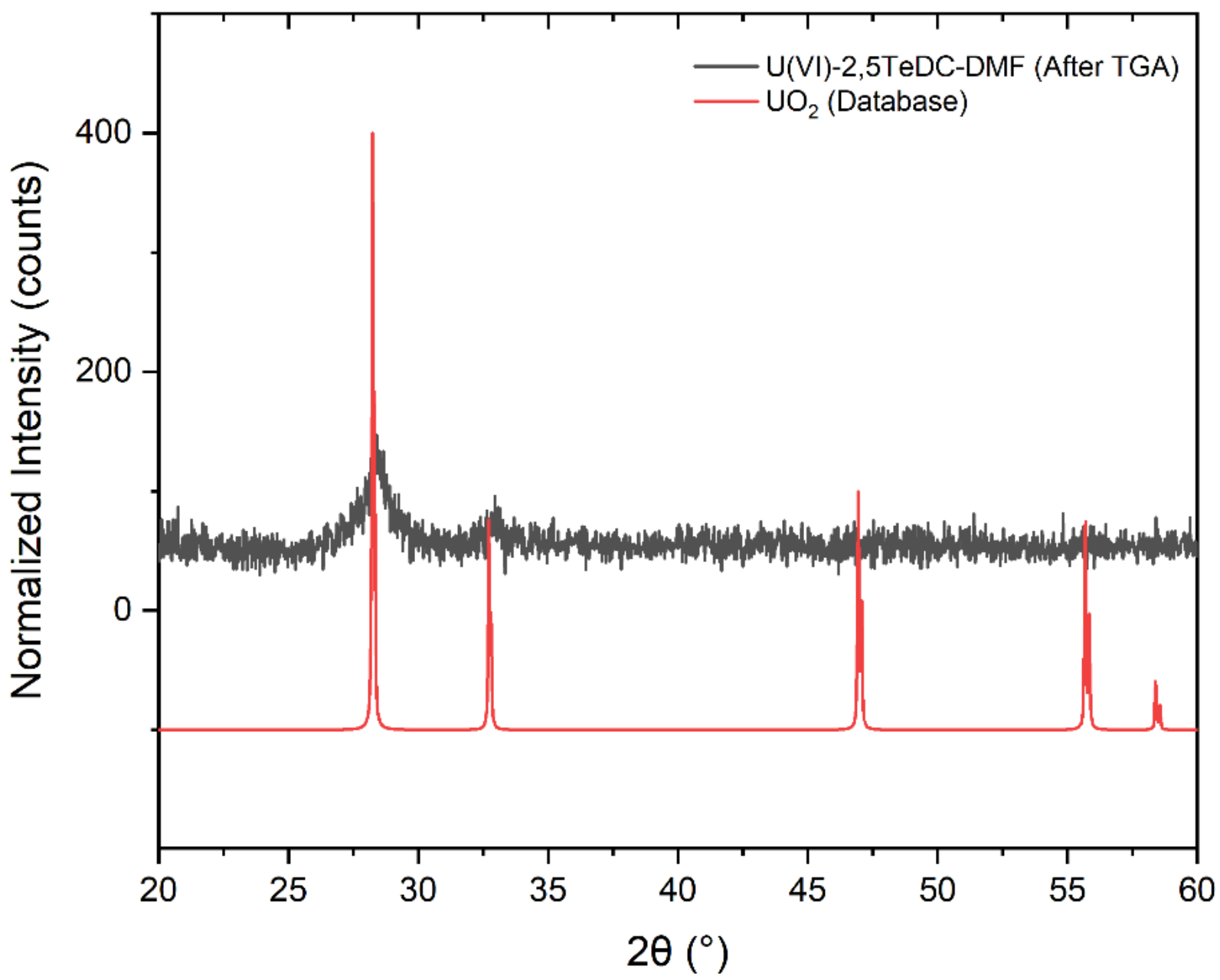

Figure S23. Post-TGA PXRD pattern of compound **2** (black) compared with known spectrum of UO_2 (PDF card no: 01-071-6416) overlaid in red.

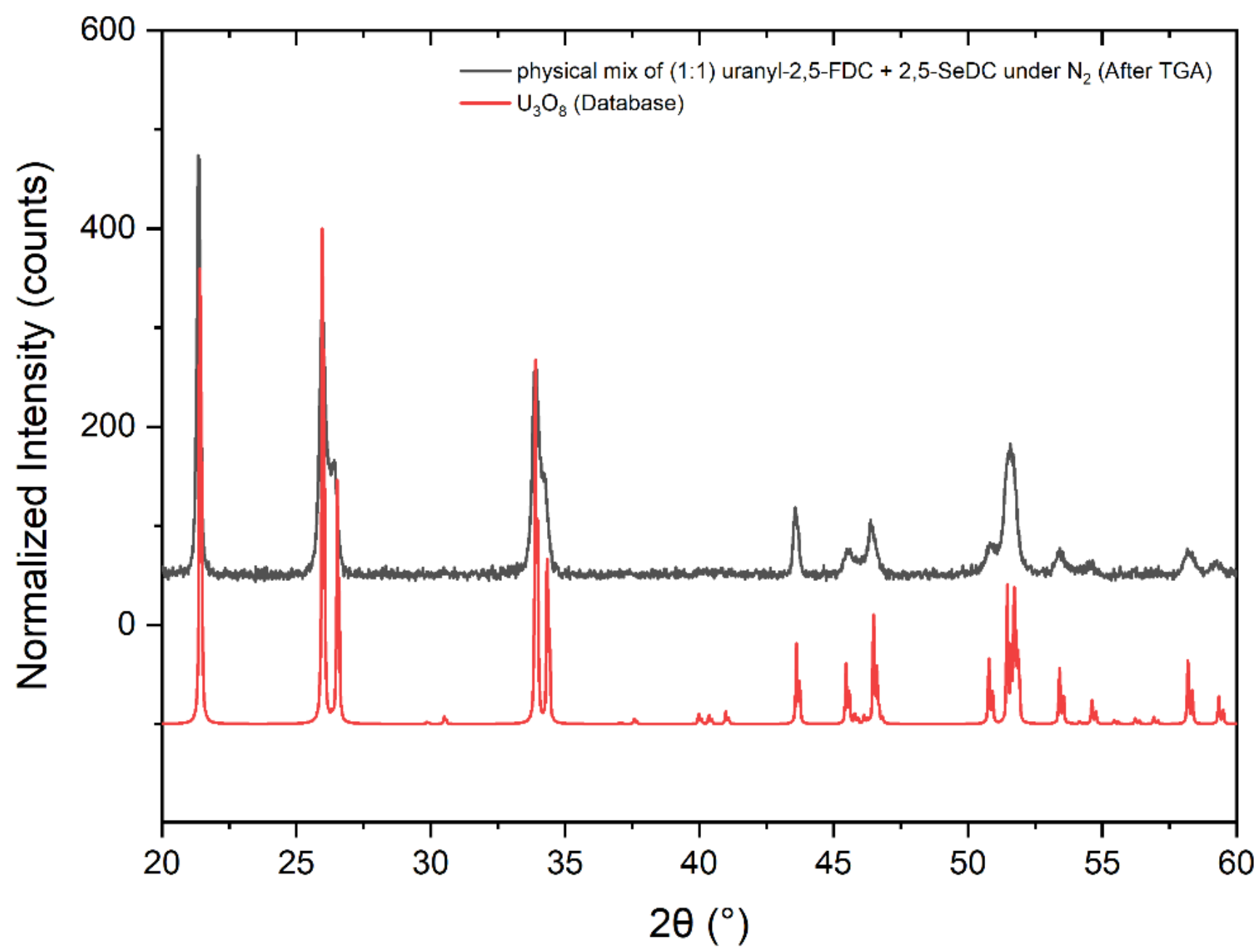

Figure S24. Post-TGA PXRD pattern of physical mixture of (1:1) U(VI)-2,5FDC with 2,5-SeDC under N_2 (black) compared with known spectrum of U_3O_8 (PDF card no: 04-007-1246) overlaid in red.

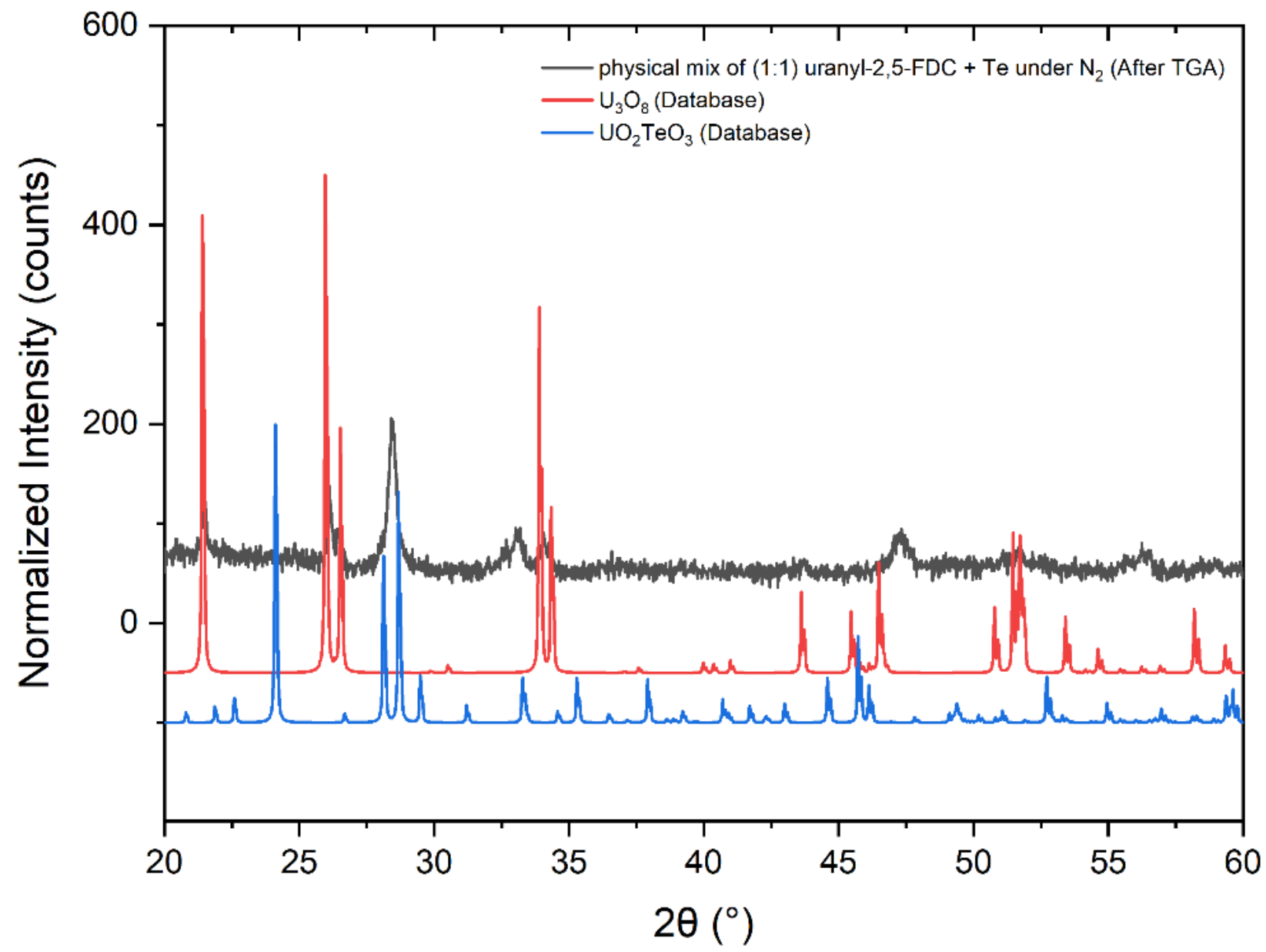

Figure S25. Post-TGA PXRD pattern of physical mixture of (1:1) U(VI)-2,5FDC with Te under N_2 (black) compared with known spectra of U_3O_8 (PDF card no: 04-007-1246) and UO_2TeO_3 (PDF card no: 04-015-5352) overlaid in red and blue, respectively.

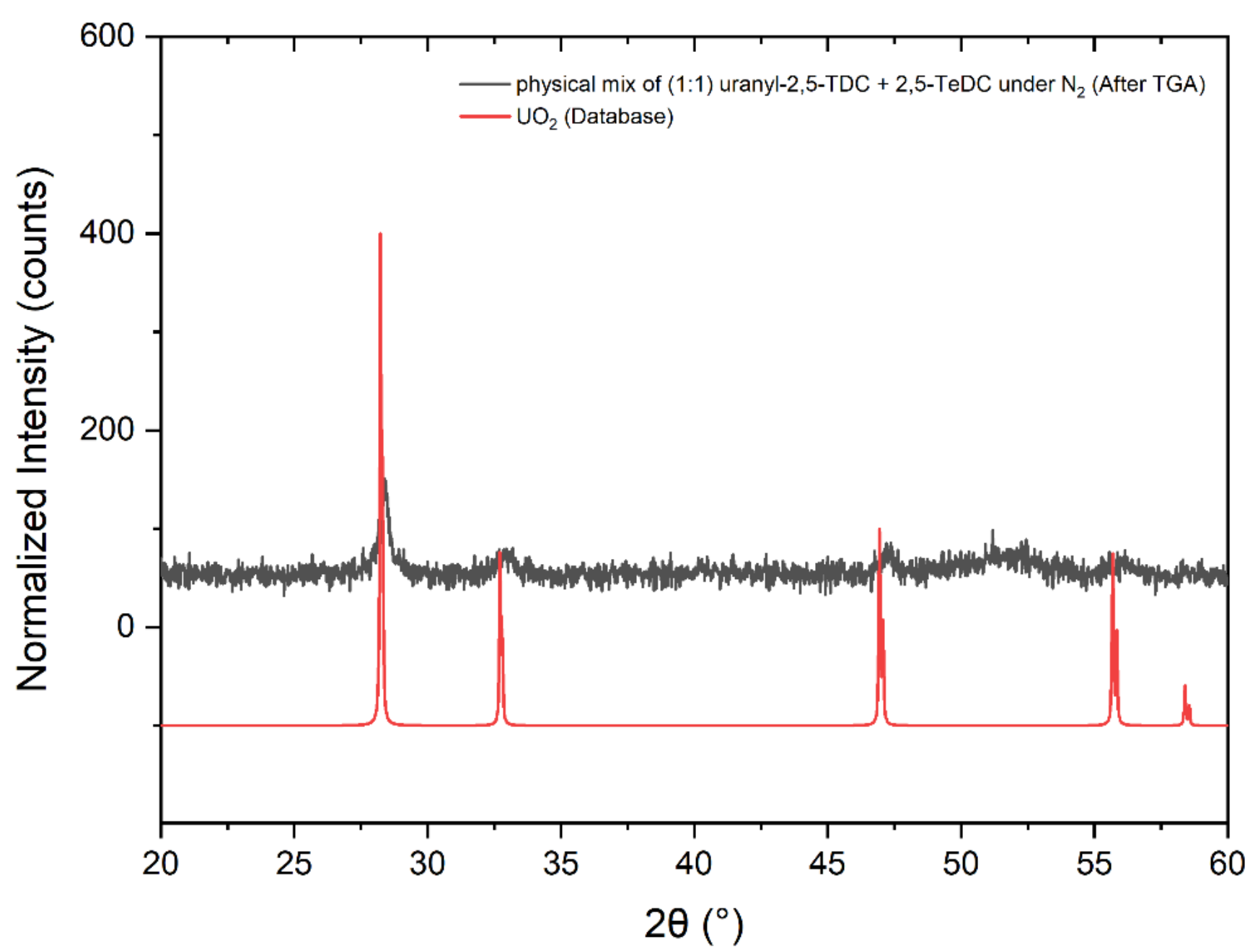

Figure S26. Post-TGA PXRD pattern of physical mixture of (1:1) U(VI)-2,5TDC with 2,5-TeDC under N_2 (black) compared with known spectrum of UO_2 (PDF card no: 01-071-6416) overlaid in red.

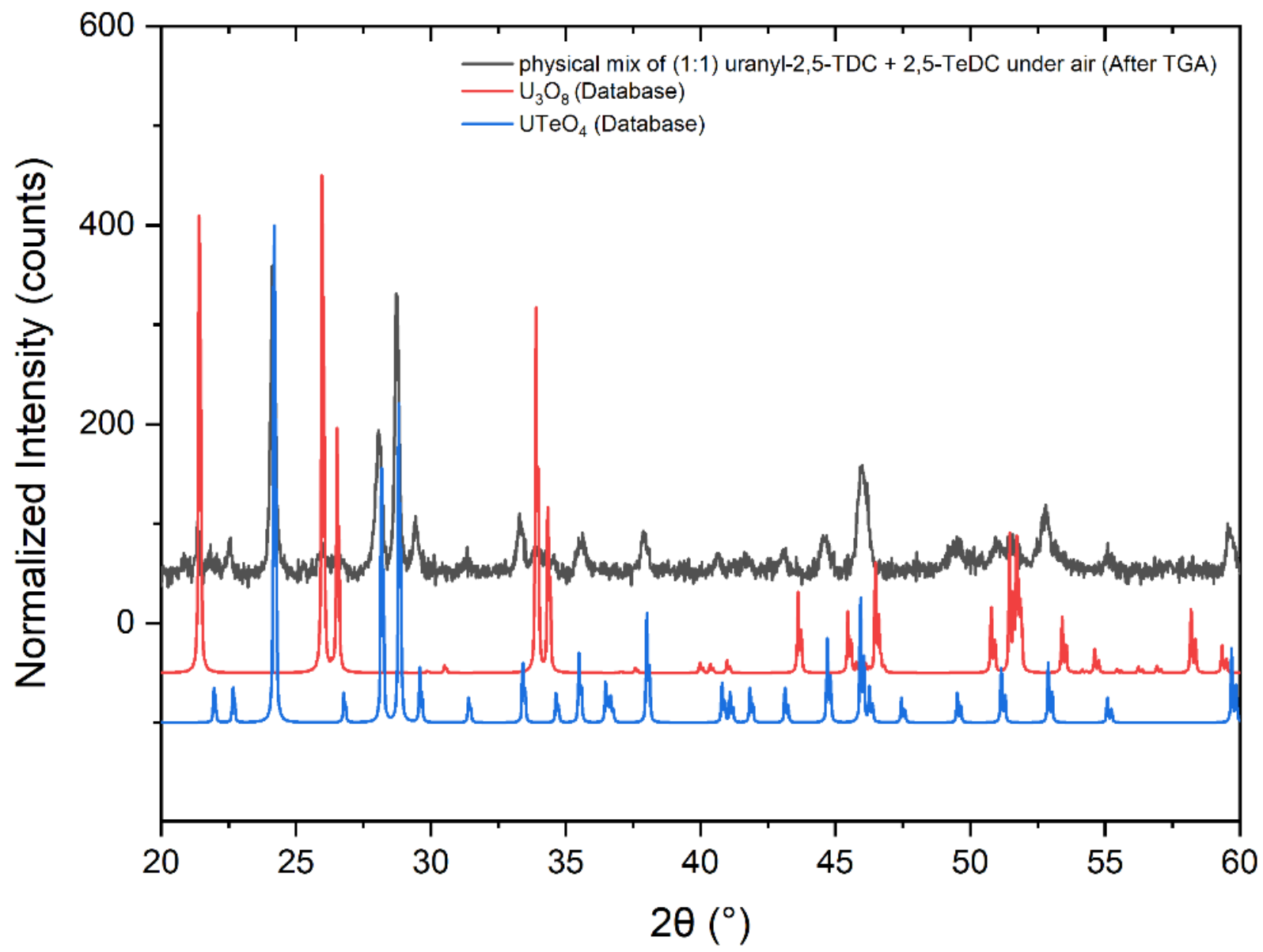

Figure S27. Post-TGA PXRD pattern of physical mixture of (1:1) U(VI)-2,5TDC with 2,5-TeDC under air (black) compared with known spectra of U_3O_8 (PDF card no: 04-007-1246) and $UTeO_4$ (PDF card no: 00-055-0562) overlaid in red and blue, respectively.

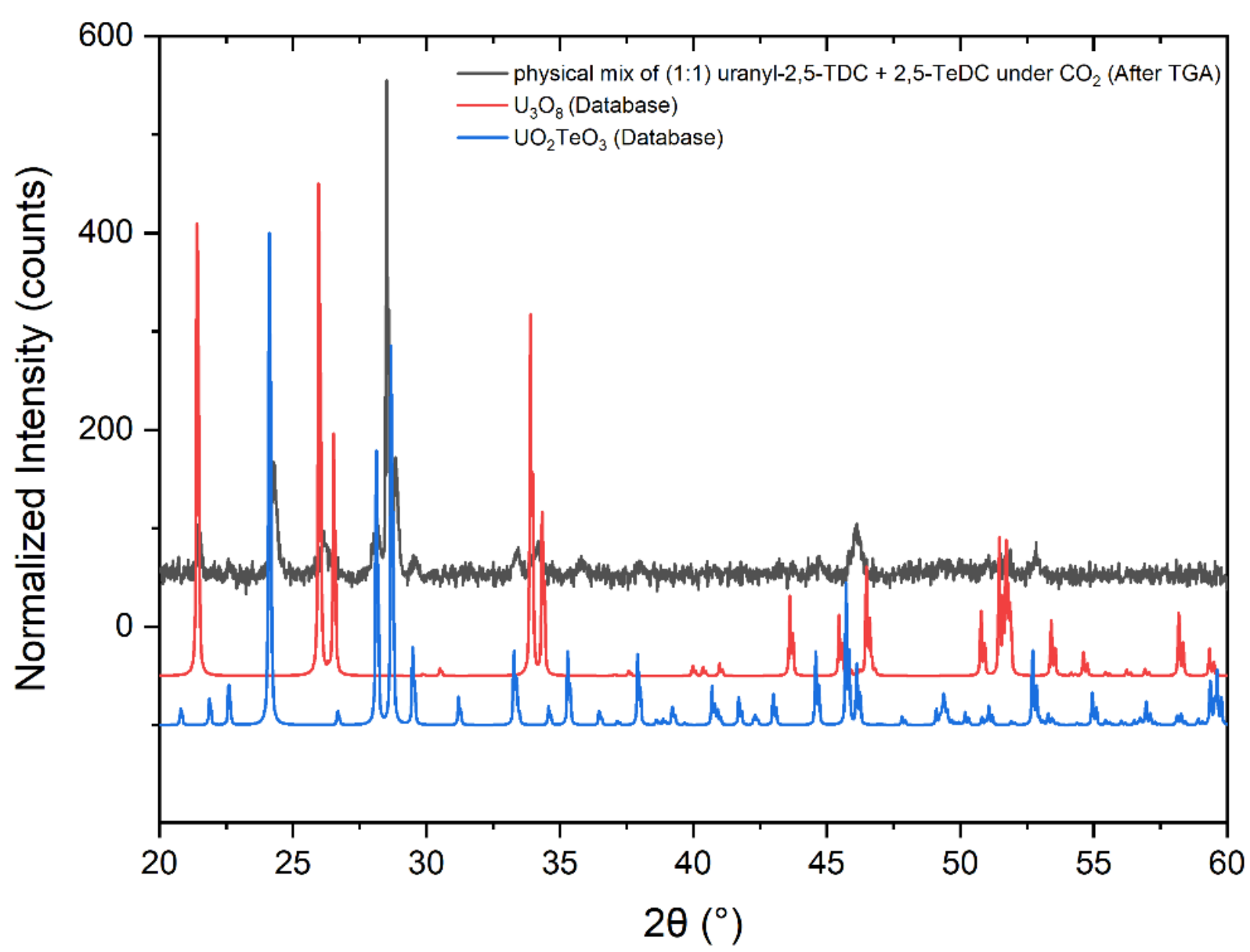

Figure S28. Post-TGA PXRD pattern of physical mixture of (1:1) U(VI)-2,5TDC with 2,5-TeDC under CO_2 (black) compared with known spectra of U_3O_8 (PDF card no: 04-007-1246) and UO_2TeO_3 (PDF card no: 04-015-5352) overlaid in red and blue, respectively.

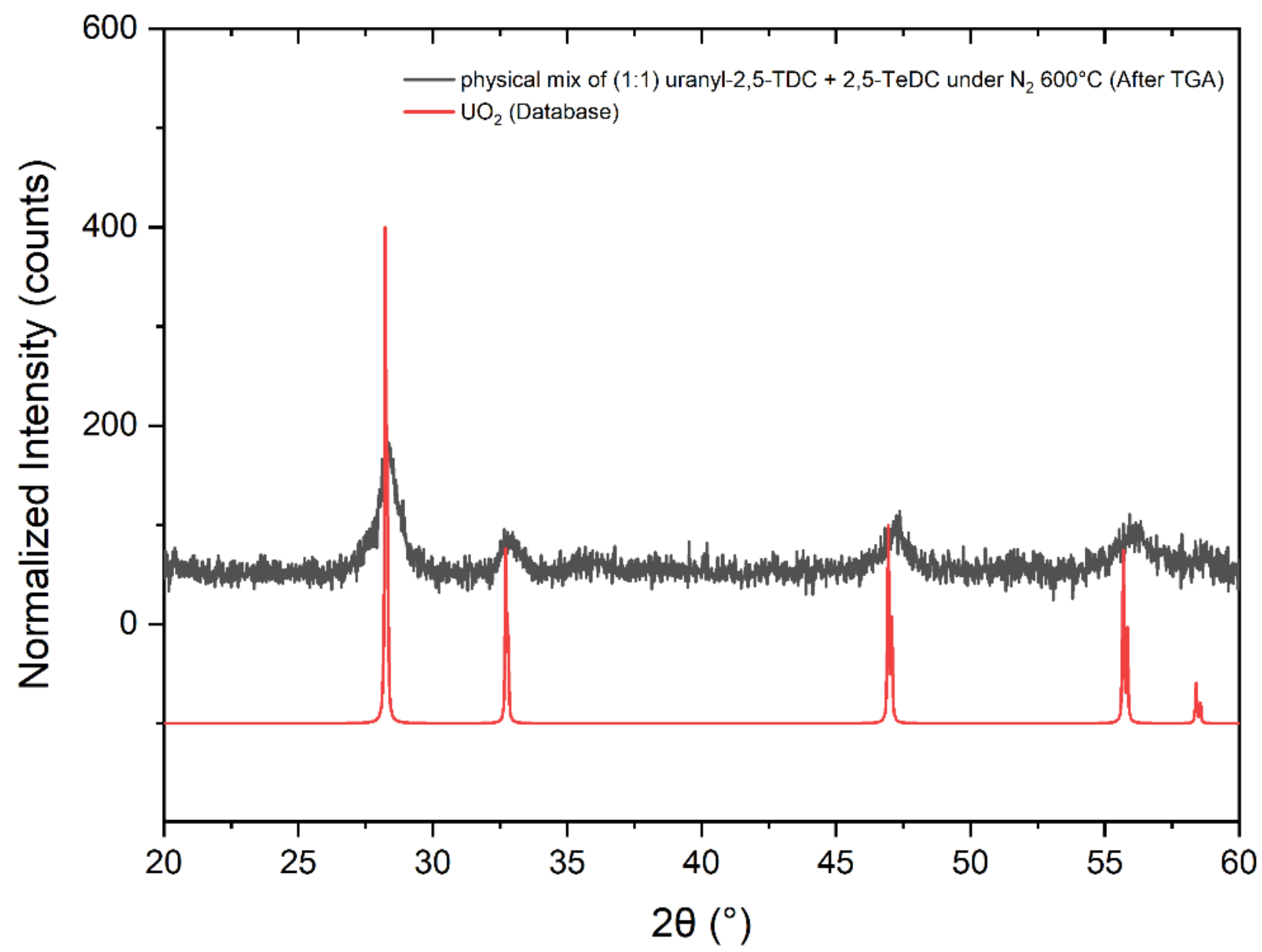

Figure S29. Post-TGA at 600 °C PXRD pattern of physical mixture of (1:1) U(VI)-2,5TDC with 2,5-TeDC under N_2 (black) compared with known spectrum of UO_2 (PDF card no: 01-071-6416) overlaid in red.

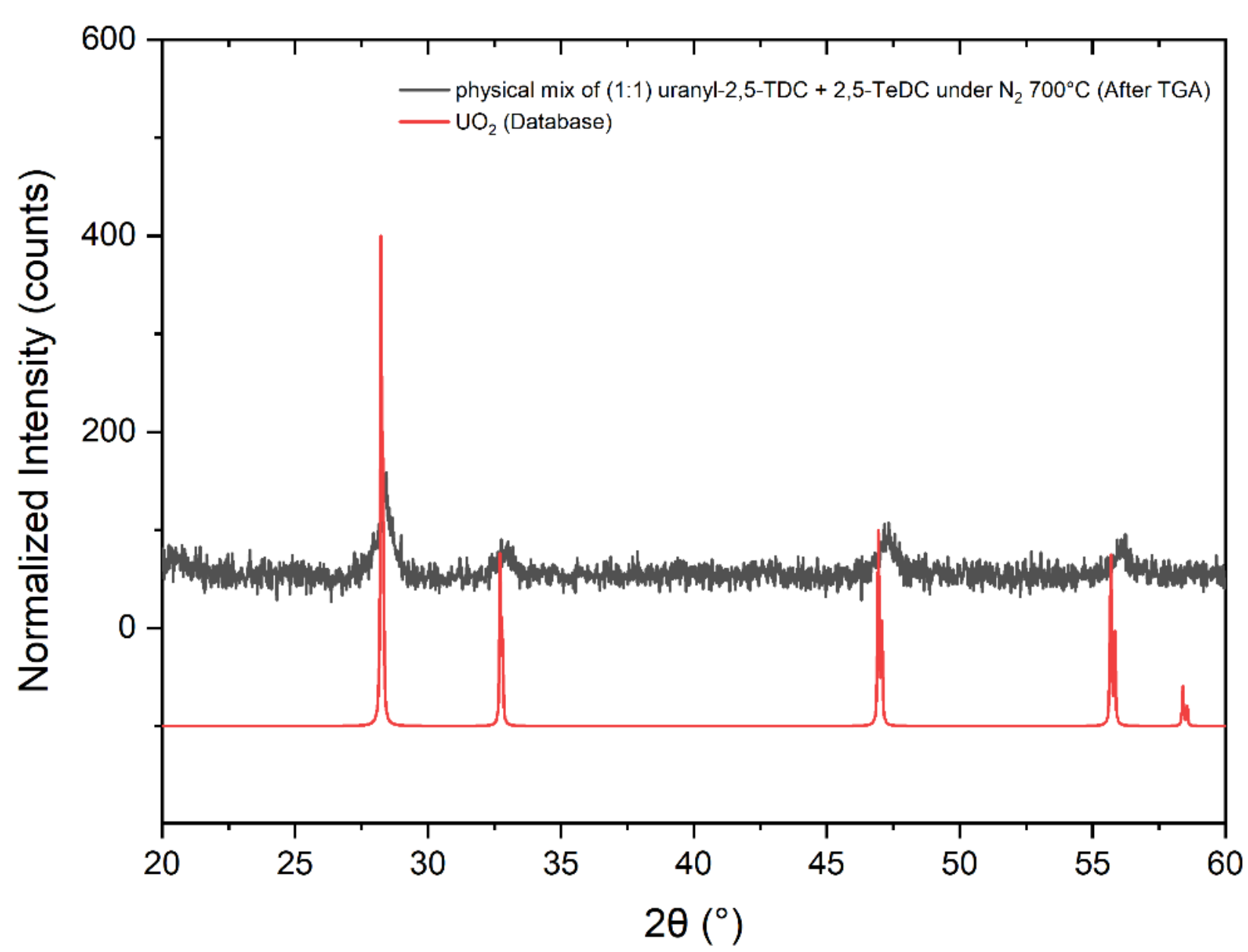

Figure S30. Post-TGA at 700 °C PXRD pattern of physical mixture of (1:1) U(VI)-2,5TDC with 2,5-TeDC under N_2 (black) compared with known spectrum of UO_2 (PDF card no: 01-071-6416) overlaid in red.

Summary of Physical Mixing Experiments

In physical mixing experiments, the U(VI) starting material was combined with a heavier chalcogenophene-based dicarboxylic acid linker (e.g., 2,5-SeDC or 2,5-TeDC) in either stoichiometric (1:1) or substoichiometric (e.g., 1:0.1) amounts. Solids were manually ground in a mortar and pestle for approximately 1-2 minutes until visually homogeneous. The resulting mixture was then subjected to thermal treatment at 600–800 °C under the specified atmosphere (flowing N_2, CO_2, or CO_2-free air). Finally, the composition of the final residues was determined by PXRD. Experimental variations in starting materials, temperature, atmosphere, and stoichiometry are summarized below in **Table S11**.

Table S13. Summary of physically mixed sample experiments evaluating UO_2 formation under different conditions.

Starting Material	Starting Material	Stoichiometry	Atmosphere	Temperature	UO_2 formation
U(VI)–2,5-FDC	2,5-SeDC	1:1	N_2	800 °C	No
U(VI)–2,5-FDC	Elemental Te	1:1	N_2	800 °C	No
U(VI)–2,5-TDC	2,5-TeDC	1:1	N_2	800 °C	Yes
U(VI)–2,5-TDC	2,5-TeDC	1:1	Air (CO_2 free)	800 °C	No
U(VI)–2,5-TDC	2,5-TeDC	1:1	CO_2	800 °C	No
U(VI)–2,5-TDC	2,5-TeDC	1:1	N_2	600 °C	Yes
U(VI)–2,5-TDC	2,5-TeDC	1:1	N_2	700 °C	Yes
U(VI)–2,5-FDC	2,5-TeDC	1:1	N_2	800 °C	Yes
U(VI)–2,5-FDC	2,5-TeDC	1:0.1	N_2	800 °C	No
U(VI)–2,5-FDC	2,5-TeDC	1:1	Air (CO_2 free)	800 °C	No
U(VI)–2,5-TDC	2,5-TeDC	1:1	CO_2	800 °C	No

Proposed Mechanism Scheme for U(VI) to U(IV) Redox Transformation

Scheme S3. Proposed mechanism for linker-mediated U(VI) to U(IV) redox transformation during thermal decomposition.

1. **Thermal decomposition of the chalcogenophene dicarboxylate linker**
   Upon heating under N_2, C–X bonds, where X = Se or Te, in the 2,5-chalcogenophene dicarboxylate linker undergo homolytic or heterolytic cleavage, generating reactive reduced organochalcogen fragments and volatile decomposition products:

$$C_4H_2\,(COO)_2X \xrightarrow{\Delta} R - XH \; + R - X \bullet \; + CO_2/CO + organic\ volatiles$$

2. **Formation of electron-donating organochalcogen species**
   The thermally generated organochalcogen intermediates, represented as R–XH, donate electrons to nearby uranyl centers:

$$2R - XH \xrightarrow{\Delta} 2R - X \bullet + 2H^+ + 2e^-$$

3. **Reduction of U(VI) to U(IV)**
   These transient species serve as solid-state reducing agents, donating electrons to nearby uranyl centers:

$$UO_2^{2+} + \ 2e^- \rightarrow UO_{2(s)}$$

4. **Coupled proposed redox step**

$$UO_2^{2+} + 2R - XH \rightarrow UO_{2(s)} + \ 2R - X \bullet + 2H^+$$

   where X = Se or Te and R–XH/R–X• represent proposed thermally generated organochalcogen intermediates.

Vibrational Spectroscopy

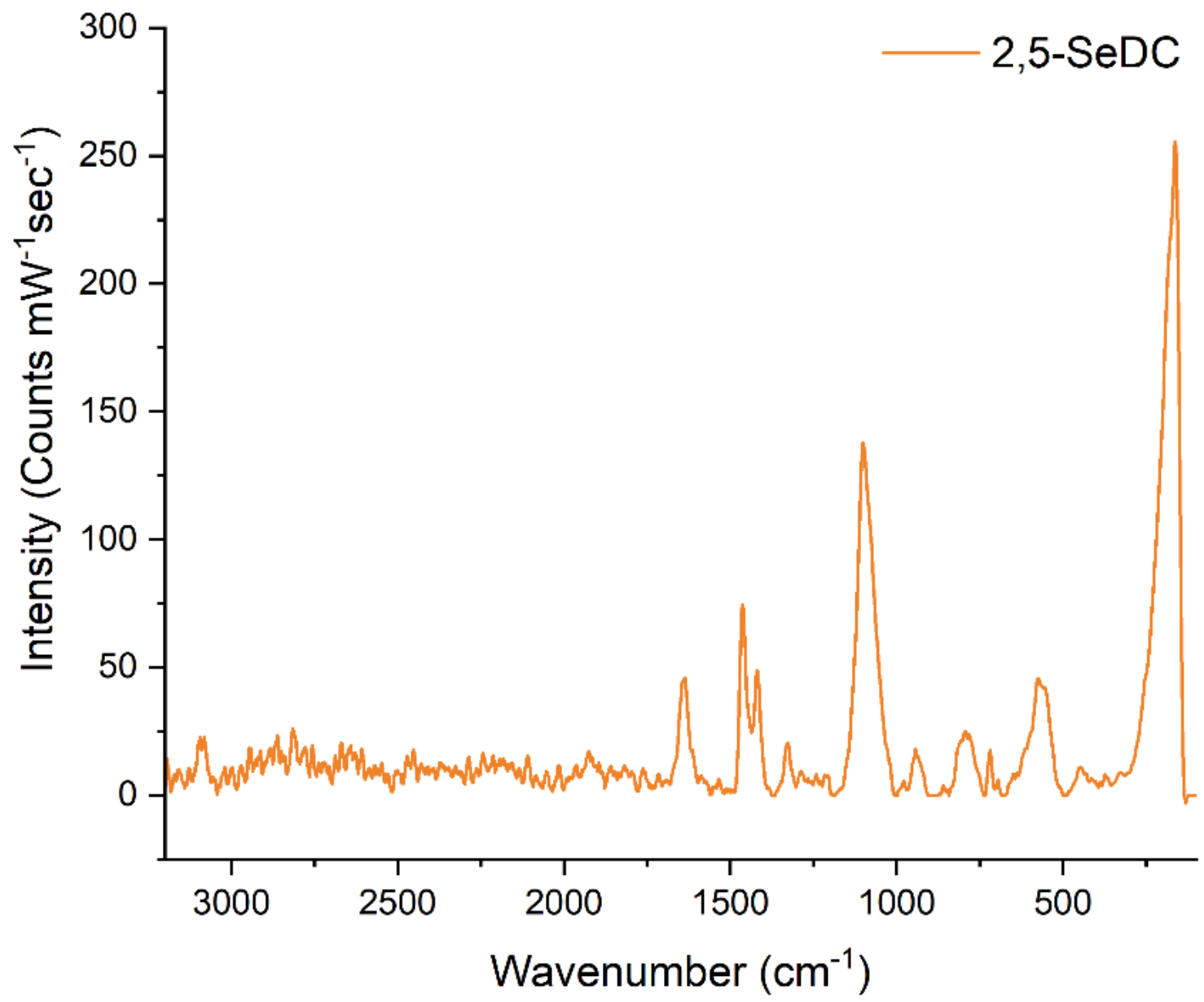

Figure S31. Solid-state Raman spectrum of 2,5-SeDC.

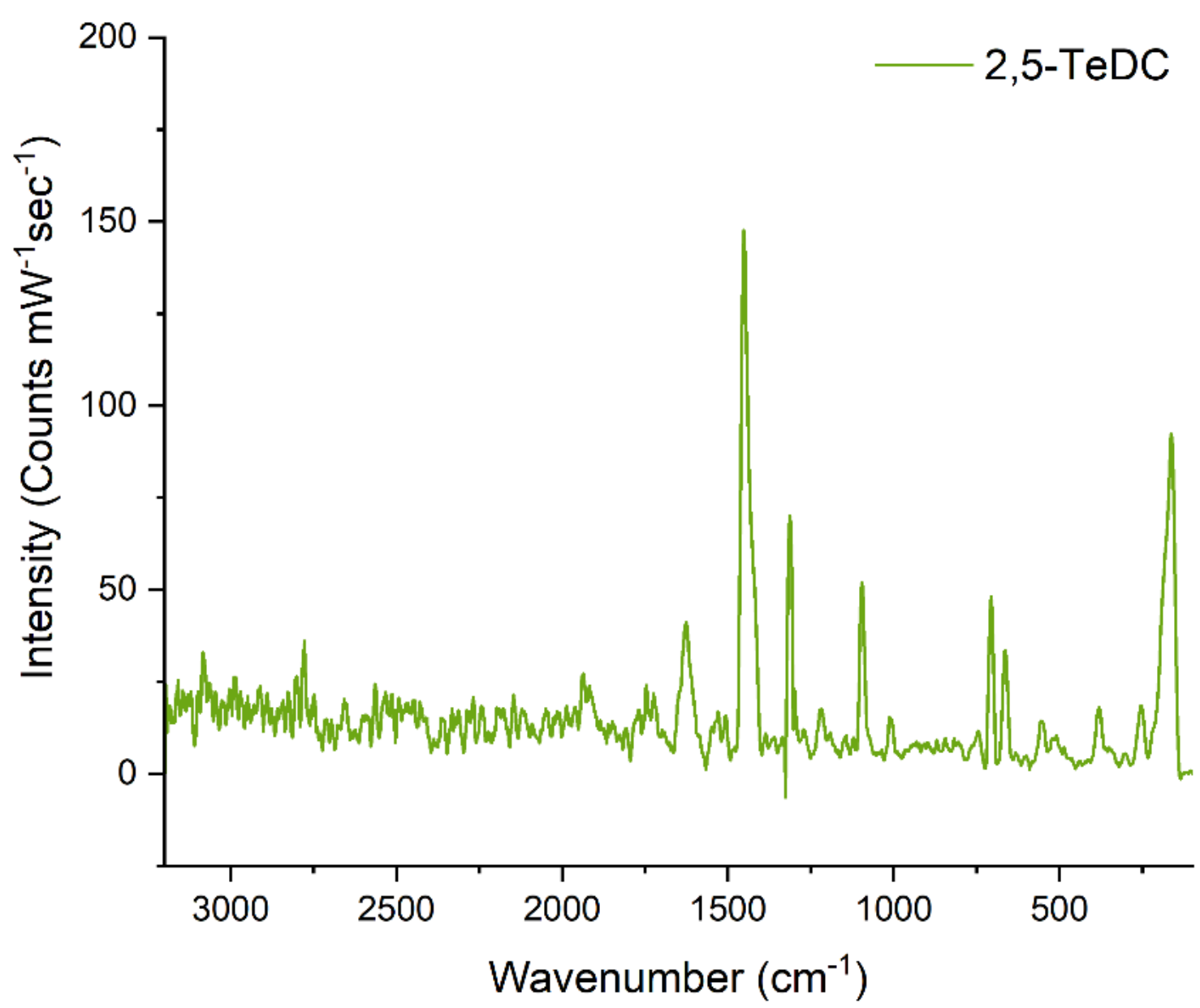

Figure S32. Solid-state Raman spectrum of 2,5-TeDC.

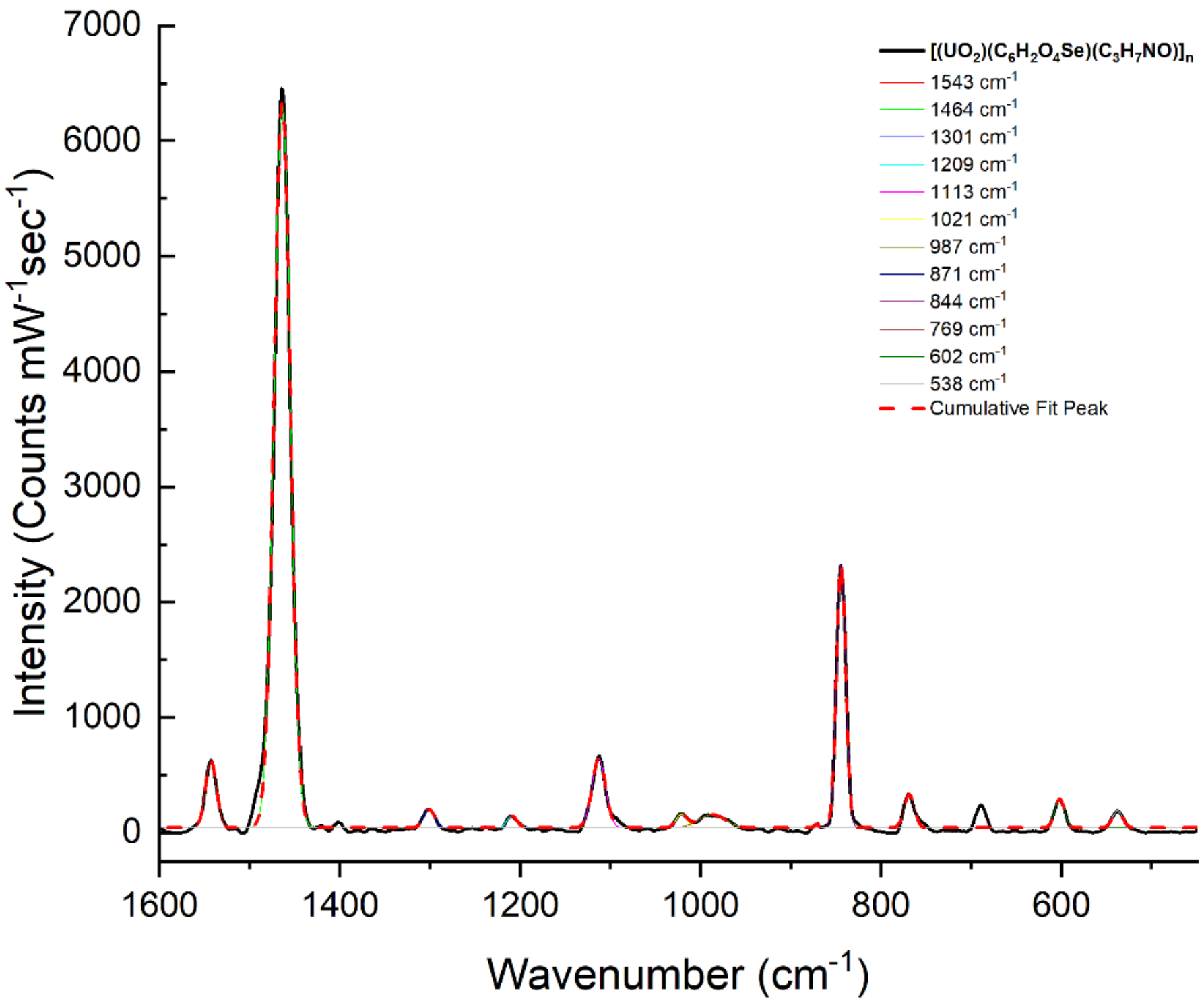

Model	Gauss											
Equation	y=y0 + (A/(w*sqrt(pi/2)))*exp(-2*((x-xc)/w)^2)											
Plot	Peak1	Peak2	Peak3	Peak4	Peak5	Peak6	Peak7	Peak8	Peak9	Peak10	Peak11	Peak12
xc	1543 ± 0	1464 ± 0	1301 ± 1	1209 ± 1	1113 ± 0	1021 ± 1	987 ± 2	871 ± 3	844 ± 0	769 ± 0	602 ± 0	538 ± 1
A	8787.90024 ± 310.44101	141575.49122 ± 380.52961	2399.04415 ± 311.91029	1406.40021 ± 303.72608	11243.21741 ± 354.92661	1703.72802 ± 406.51832	4071.56879 ± 581.11214	192.23185 ± 197.44973	28700.75306 ± 294.91059	4014.3322 ± 310.16729	3242.48096 ± 303.57278	2055.04986 ± 316.29513
Reduced Chi-Sqr	2.01E+03											
R-Square (COD)	0.99653											
Adj. R-Square	0.99637											

Figure S33. (**Top**) The fitted Raman spectrum of compound **1** in the 1600–400 cm^{-1} spectral window. (**Bottom**) Fitted spectral features result in a R^2 of 0.996 and a χ^2 of 2.01 x 10^3.

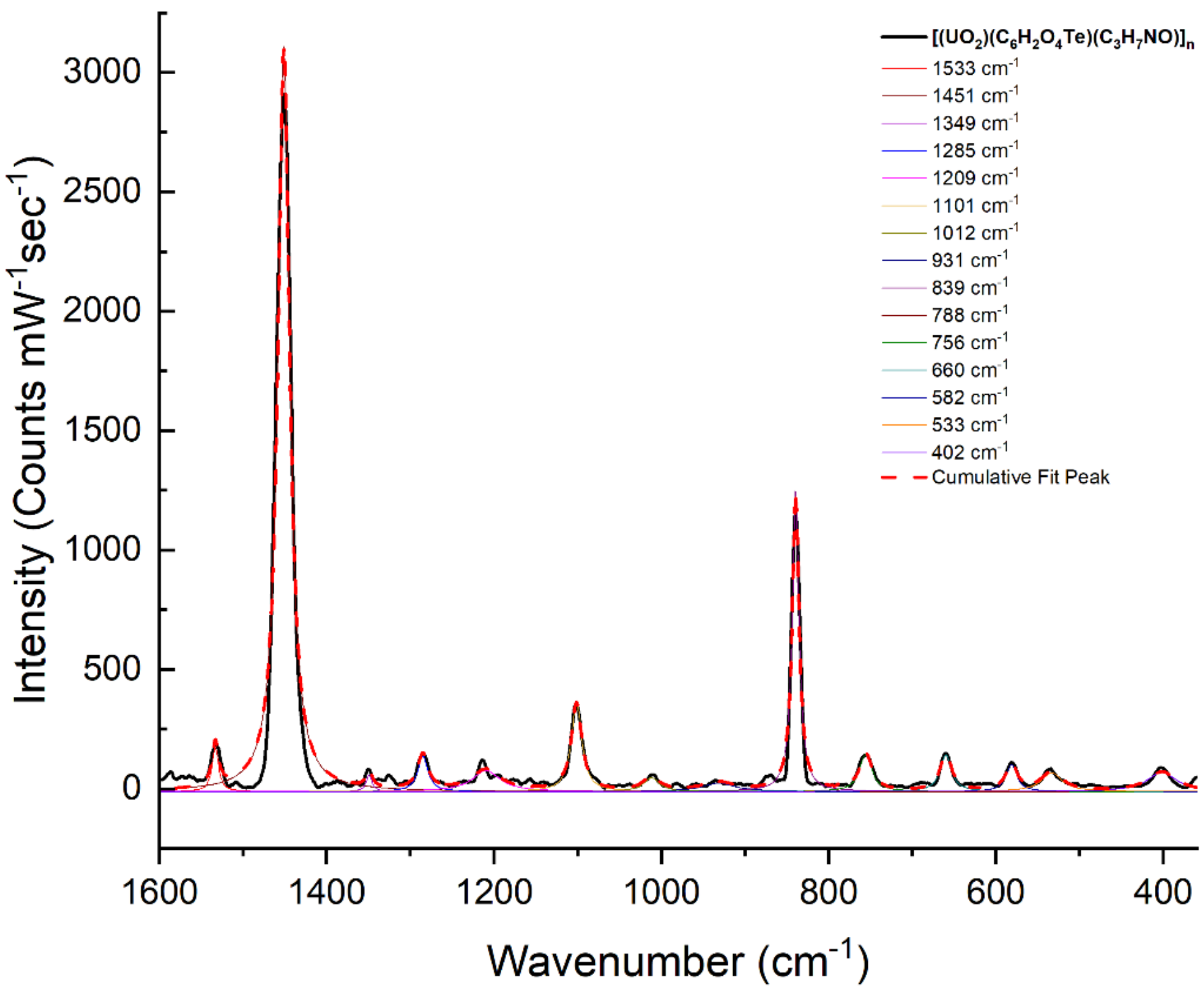

Model	Lorentz														
Equation	y = y0 + (2*A/pi)*(w/(4*(x-xc)^2 + w^2))														
Plot	Peak1	Peak2	Peak3	Peak4	Peak5	Peak6	Peak7	Peak8	Peak9	Peak10	Peak11	Peak12	Peak13	Peak14	Peak15
xc	1533 ± 1	1451 ± 0	1349 ± 2	1285 ± 1	1209 ± 4	1101 ± 1	1012 ± 4	931 ± 10	839 ± 0	788 ± 9	756 ± 1	660 ± 1	582 ± 2	533 ± 4	402 ± 4
A	2710.20524 ± 466.57365	82385.68863 ± 638.86646	916.38381 ± 451.75136	3416.53607 ± 650.23627	5681.10562 ± 1106.54895	8779.01586 ± 640.0087	2105.60477 ± 900.10844	2652.17212 ± 1321.45404	19214.89977 ± 521.58391	22.28291 ± 199.86467	4368.83983 ± 712.05904	4018.85564 ± 674.45973	2789.11591 ± 779.82417	3758.91995 ± 1099.14714	5387.59803 ± 1150.34235
Reduced Chi-Sqr	2.52E+03														
R-Square (COD)	0.9787														
Adj. R-Square	0.97743														

Figure S34. (**Top**) The fitted Raman spectrum of compound **2** in the 1600–400 cm^{-1} spectral window. (**Bottom**) Fitted spectral features result in a R^2 of 0.978 and a χ^2 of 2.52 x 10^3.

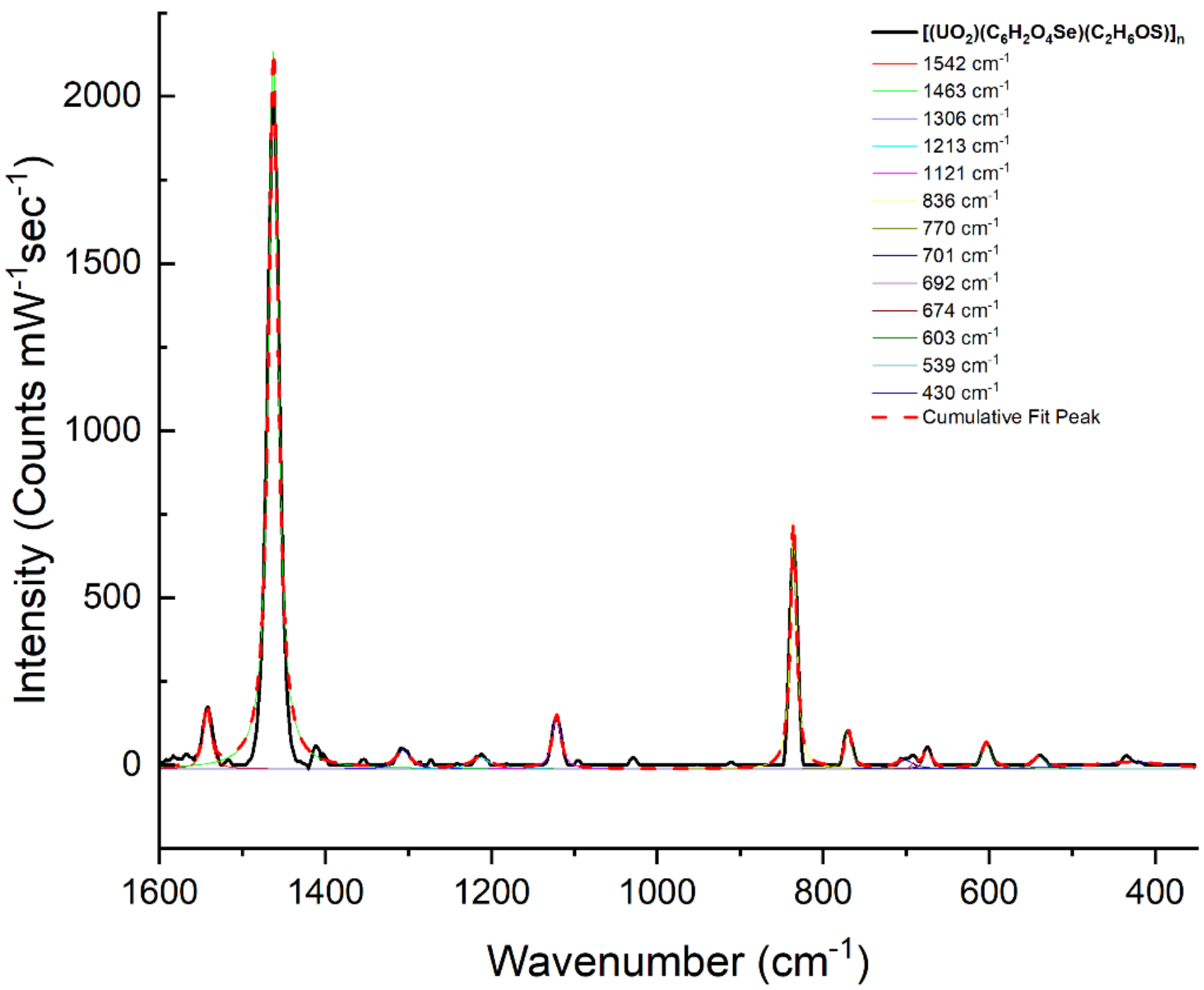

Model	Lorentz												
Equation	y = y0 + (2*A/pi)*(w/(4*(x-xc)^2 + w^2))												
Plot	Peak1	Peak2	Peak3	Peak4	Peak5	Peak6	Peak7	Peak8	Peak9	Peak10	Peak11	Peak12	Peak13
xc	1542 ± 0	1463 ± 0	1306 ± 2	1213 ± 4	1121 ± 1	836 ± 0	770 ± 1	701 ± 17	692 ± 4	674 ± 2	603 ± 1	539 ± 3	430 ± 15
A	3028.6053 ± 281.45059	47265.58606 ± 325.57107	1649.65244 ± 379.48617	1443.08855 ± 465.70982	3142.68906 ± 316.68502	10395.78748 ± 271.1394	1838.50491 ± 299.05007	1018.60673 ± 1632.33752	188.84756 ± 873.72955	954.31495 ± 540.27216	1595.9855 ± 349.88361	953.99602 ± 469.05229	3744.39141 ± 1405.31859
Reduced Chi-Sqr	7.92E+02												
R-Square (COD)	0.98302												
Adj. R-Square	0.98215												

Figure S35. (**Top**) The fitted Raman spectrum of compound **3** in the 1600–400 cm^{-1} spectral window. (**Bottom**) Fitted spectral features result in a R^2 of 0.983 and a χ^2 of 7.92 x 10^2.

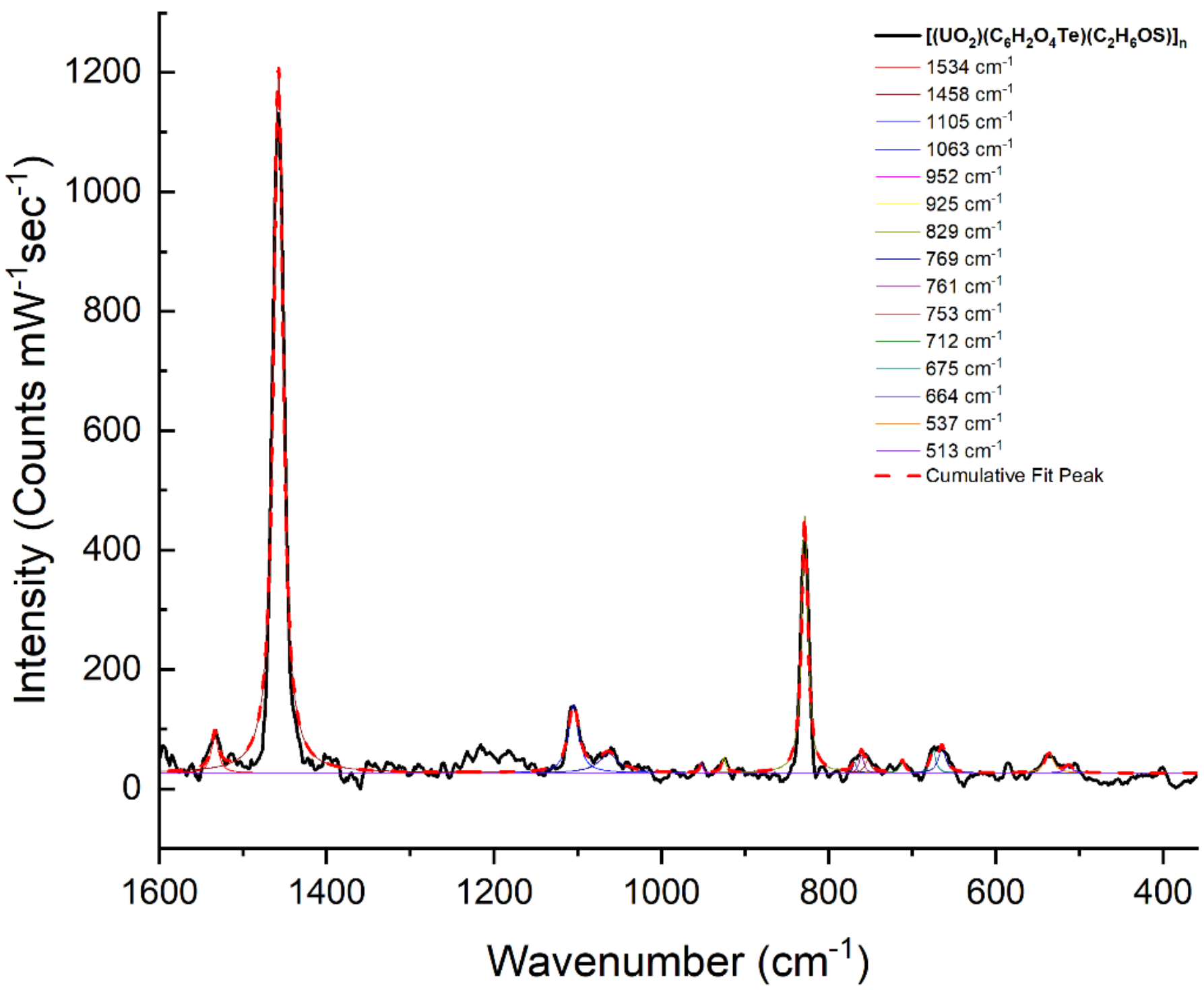

Model	Lorentz														
Equation	y = y0 + (2*A/pi)*(w/(4*(x-xc)^2 + w^2))														
Plot	Peak1	Peak2	Peak3	Peak4	Peak5	Peak6	Peak7	Peak8	Peak9	Peak10	Peak11	Peak12	Peak13	Peak14	Peak15
xc	1534 ± 1	1458 ± 0	1105 ± 1	1063 ± 3	952 ± 2	925 ± 2	829 ± 0	769 ± 3	761 ± 2	753 ± 5	712 ± 3	675 ± 2	664 ± 2	537 ± 2	513 ± 6
A	1013.86693 ± 193.79911	25622.47719 ± 224.32572	2716.749 ± 303.47875	1497.86103 ± 414.70992	107.22886 ± 129.42871	223.46153 ± 149.72002	5984.82857 ± 186.36813	145.40518 ± 258.76454	317.71352 ± 581.62065	310.10854 ± 514.35522	229.94317 ± 176.0015	443.1901 ± 367.70964	711.58178 ± 418.83451	681.0721 ± 289.47548	202.50301 ± 261.68079
Reduced Chi-Sqr	3.82E+02														
R-Square (COD)	0.97356														
Adj. R-Square	0.97199														

Figure S36. (**Top**) The fitted Raman spectrum of compound **4** in the 1600–400 cm^{-1} spectral window. (**Bottom**) Fitted spectral features result in a R^2 of 0.973 and a χ^2 of 3.82 x 10^2.

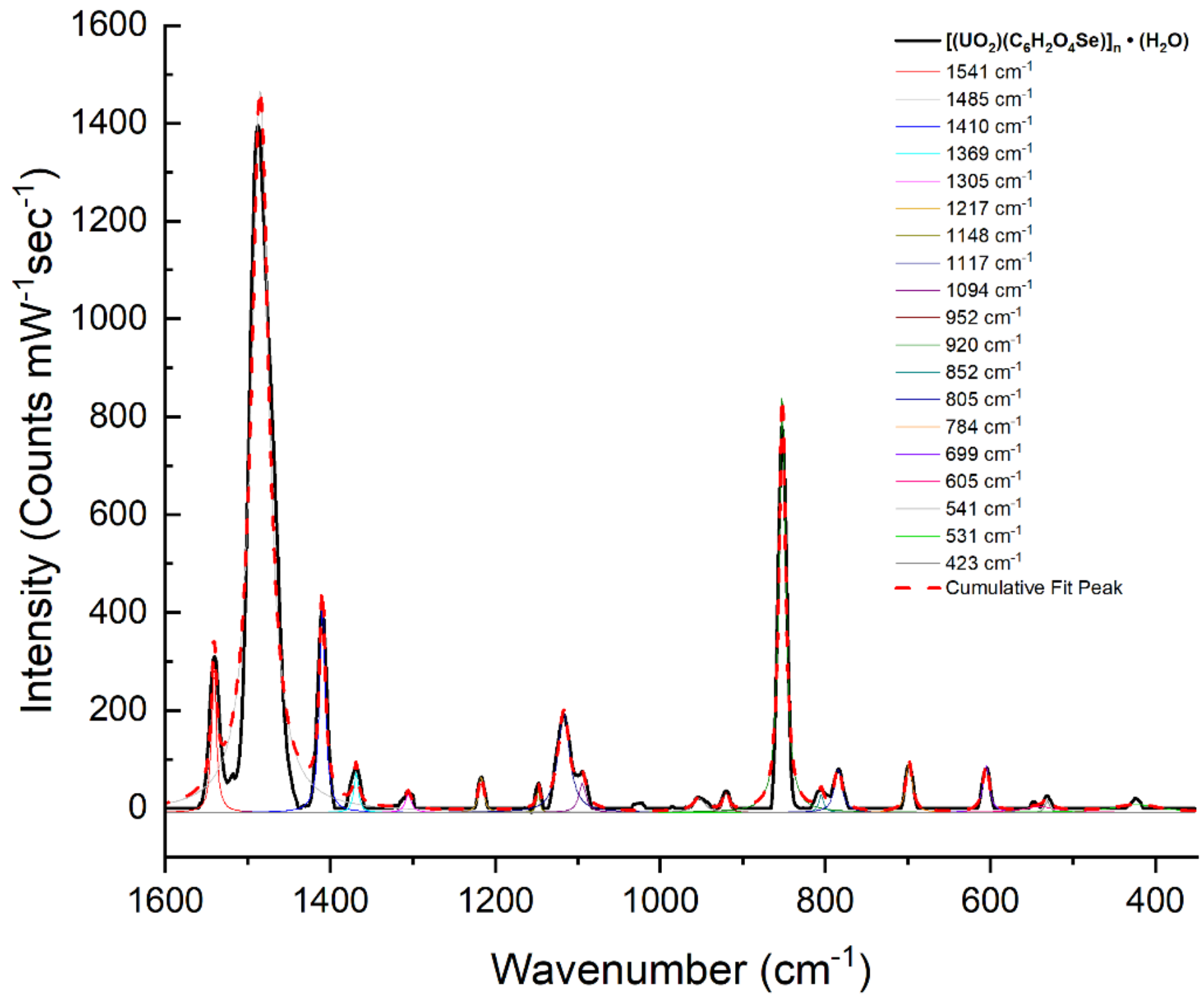

Model	Lorentz																		
Equation	y = y0 + (2*A/pi)*(w/(4*(x-xc)^2 + w^2))																		
Plot	Peak1	Peak2	Peak3	Peak4	Peak5	Peak6	Peak7	Peak8	Peak9	Peak10	Peak11	Peak12	Peak13	Peak14	Peak15	Peak16	Peak17	Peak18	Peak19
xc	1541 ± 0	1485 ± 0	1410 ± 0	1369 ± 1	1305 ± 3	1217 ± 1	1148 ± 1	1117 ± 1	1094 ± 2	952 ± 6	920 ± 3	852 ± 0	805 ± 3	784 ± 2	699 ± 1	605 ± 1	541 ± 39	531 ± 5	423 ± 22
A	3212.36789 ± 331.40823	59285.40416 ± 637.01782	5454.16821 ± 356.15837	859.31662 ± 317.34732	533.18869 ± 373.29876	855.13882 ± 332.06693	307.35045 ± 259.34922	5452.25876 ± 745.27802	998.76835 ± 581.32931	751.89664 ± 557.40492	490.94121 ± 373.11123	13018.98615 ± 392.30081	418.53312 ± 399.35381	1557.03285 ± 490.16913	1494.74941 ± 386.28281	1442.88198 ± 438.85048	891.27948 ± 1596.51203	272.53326 ± 924.78168	1780.975 ± 1469.52039
Reduced Chi-Sqr	1.54E+03																		
R-Square (COD)	0.96507																		
Adj. R-Square	0.96236																		

Figure S37. (**Top**) The fitted Raman spectrum of compound **5** in the 1600–400 cm^{-1} spectral window. (**Bottom**) Fitted spectral features result in a R^2 of 0.965 and a χ^2 of 1.54 x 10^3.

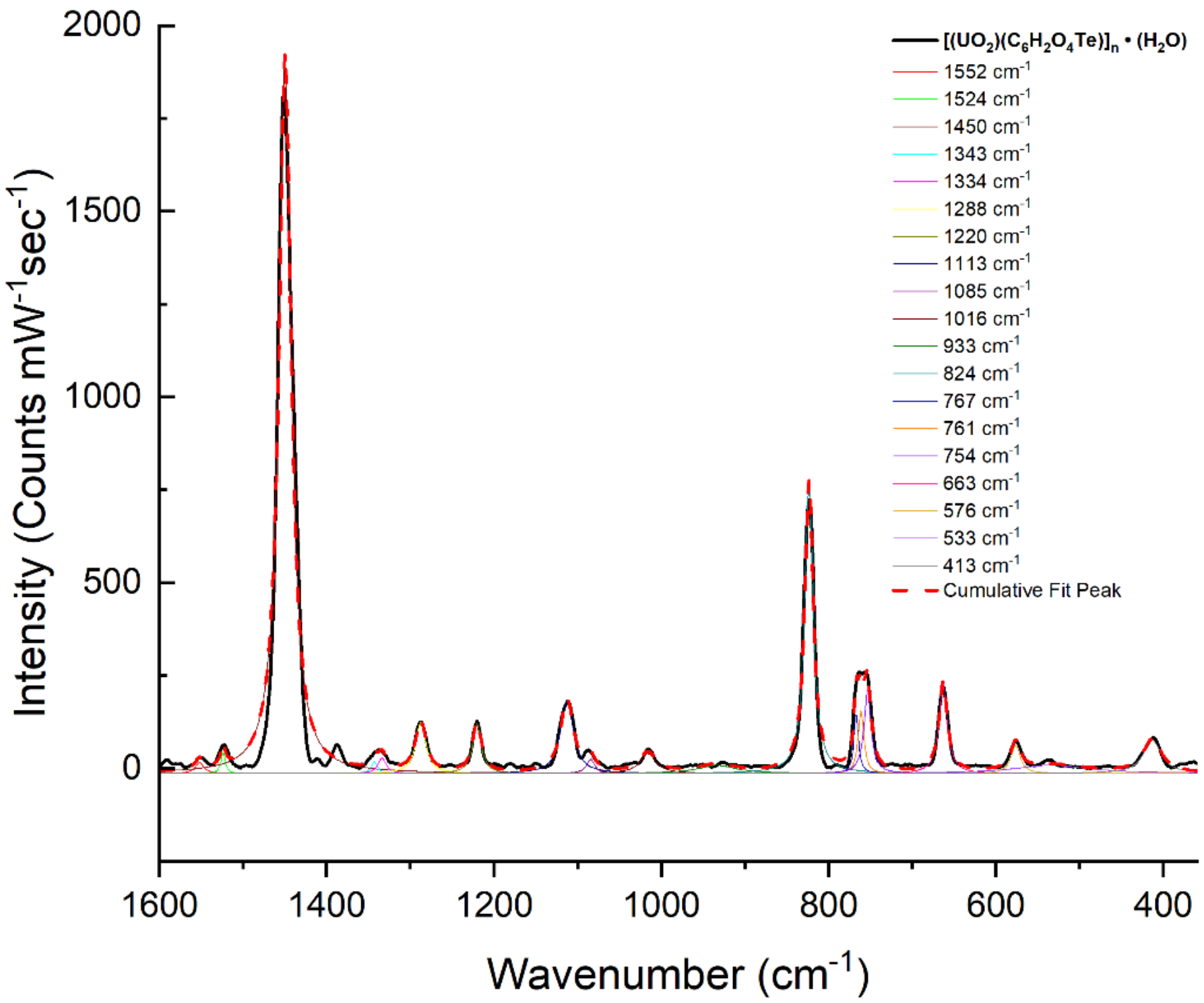

Model	Lorentz																		
Equation	y = y0 + (2*A/pi)*(w/(4*(x-xc)^2 + w^2))																		
Plot	Peak1	Peak2	Peak3	Peak4	Peak5	Peak6	Peak7	Peak8	Peak9	Peak10	Peak11	Peak12	Peak13	Peak14	Peak15	Peak16	Peak17	Peak18	Peak19
xc	1552 ± 4	1524 ± 1	1450 ± 0	1343 ± 5	1334 ± 5	1288 ± 1	1220 ± 1	1113 ± 1	1085 ± 3	1016 ± 3	933 ± 11	824 ± 0	767 ± 1	761 ± 1	754 ± 1	663 ± 0	576 ± 2	533 ± 23	413 ± 2
A	572.58847 ± 357.42458	538.38845 ± 248.63527	53440.46006 ± 410.59509	461.12554 ± 1044.47773	775.95382 ± 1144.07625	3229.05447 ± 405.60212	2639.7201 ± 351.68567	5677.38997 ± 542.34168	722.26078 ± 450.72792	1999.57525 ± 591.68715	1955.78497 ± 924.04255	14811.47334 ± 367.52095	1655.23096 ± 1652.74279	2115.56677 ± 3075.42952	3525.8922 ± 1684.99524	4541.16639 ± 412.35621	1557.52502 ± 719.6908	3791.8371 ± 2007.52061	3709.4909 ± 676.94773
Reduced Chi-Sqr	9.90E+02																		
R-Square (COD)	0.9798																		
Adj. R-Square	0.97824																		

Figure S38. (**Top**) The fitted Raman spectrum of compound **6** in the 1600–400 cm^{-1} spectral window. (**Bottom**) Fitted spectral features result in a R^2 of 0.979 and a χ^2 of 9.90 x 10^2.

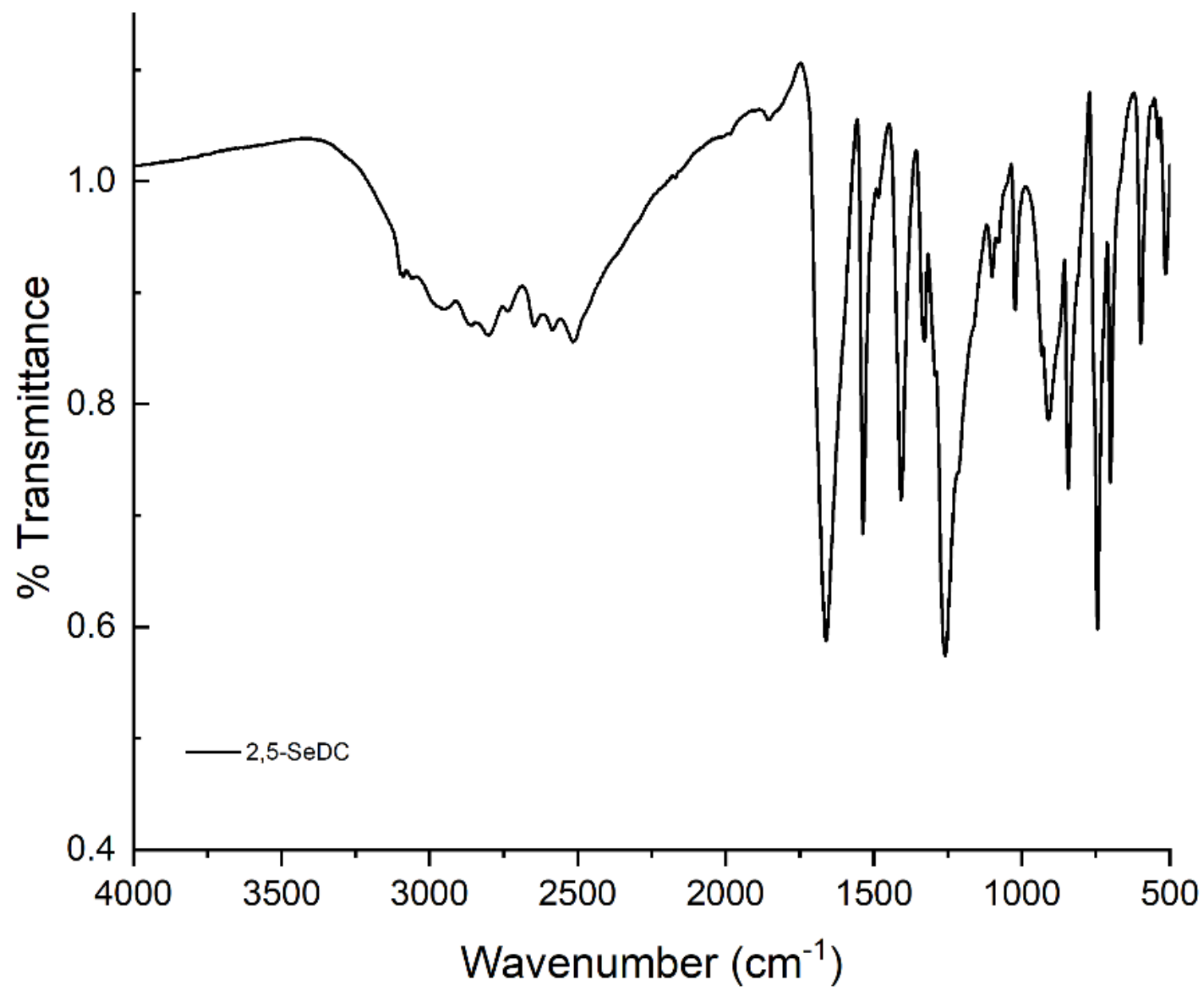

Figure S39. Mid-IR spectrum of 2,5-SeDC.

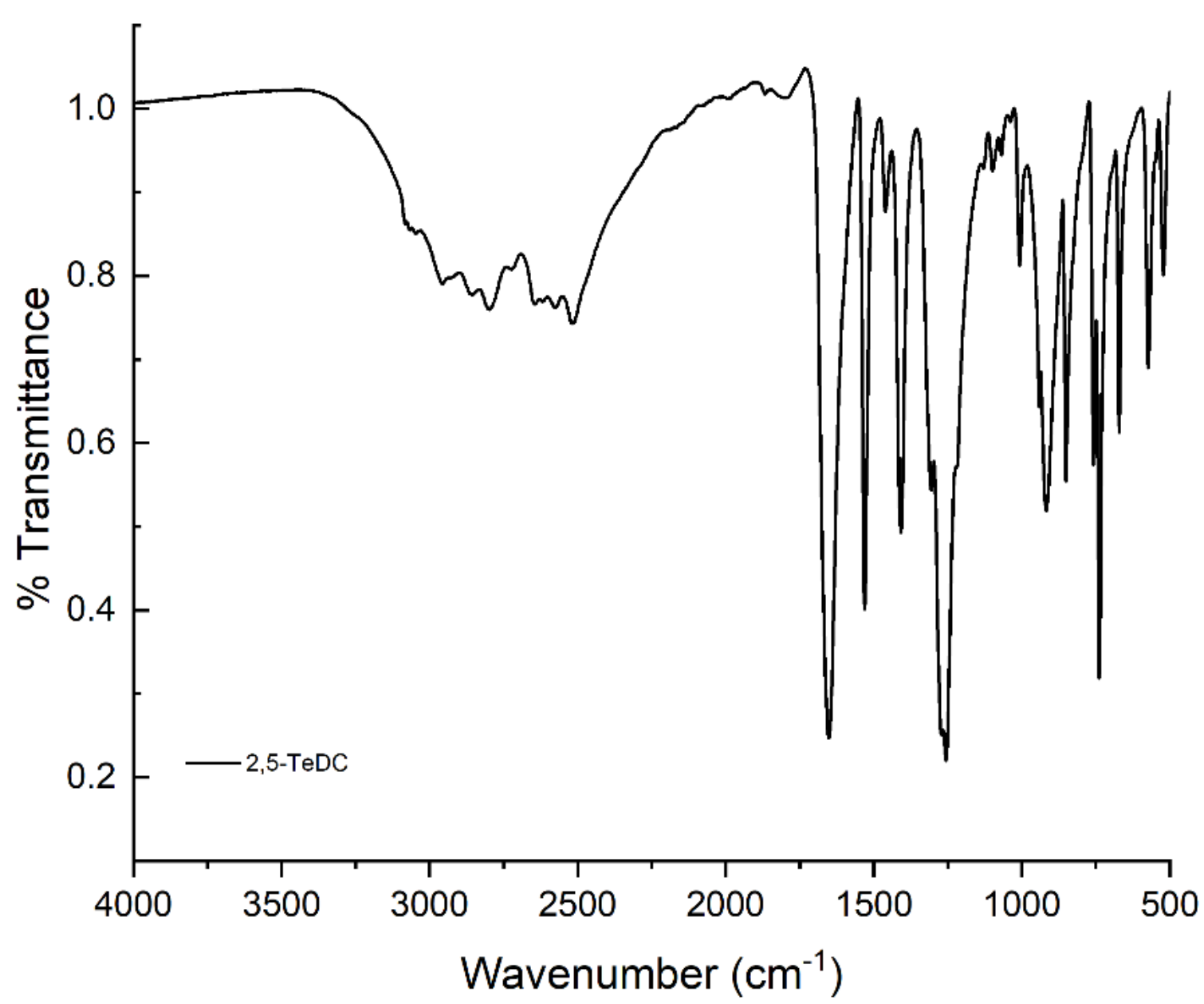

Figure S40. Mid-IR spectrum of 2,5-TeDC.

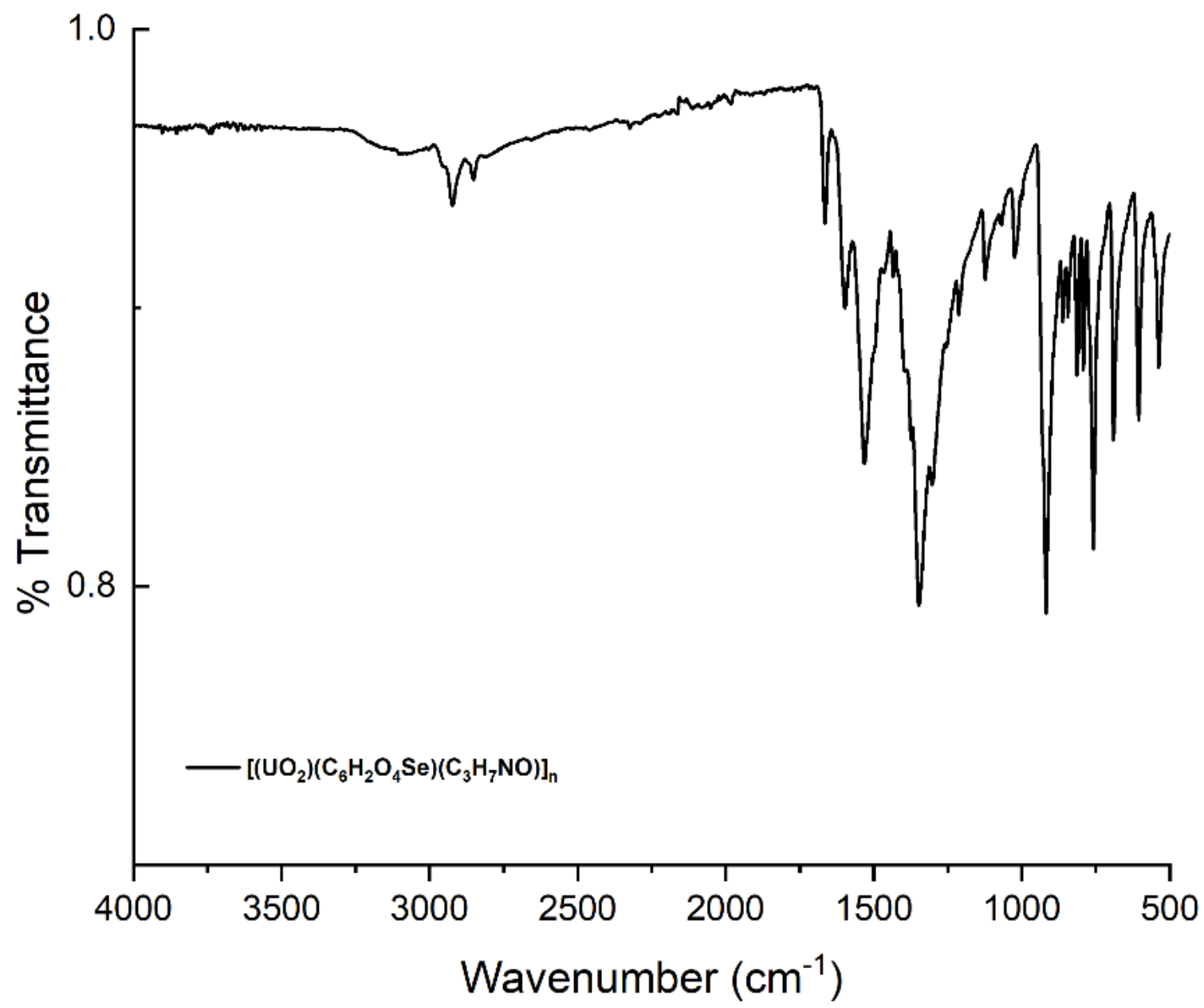

Figure S41. Mid-IR spectrum of compound **1**.

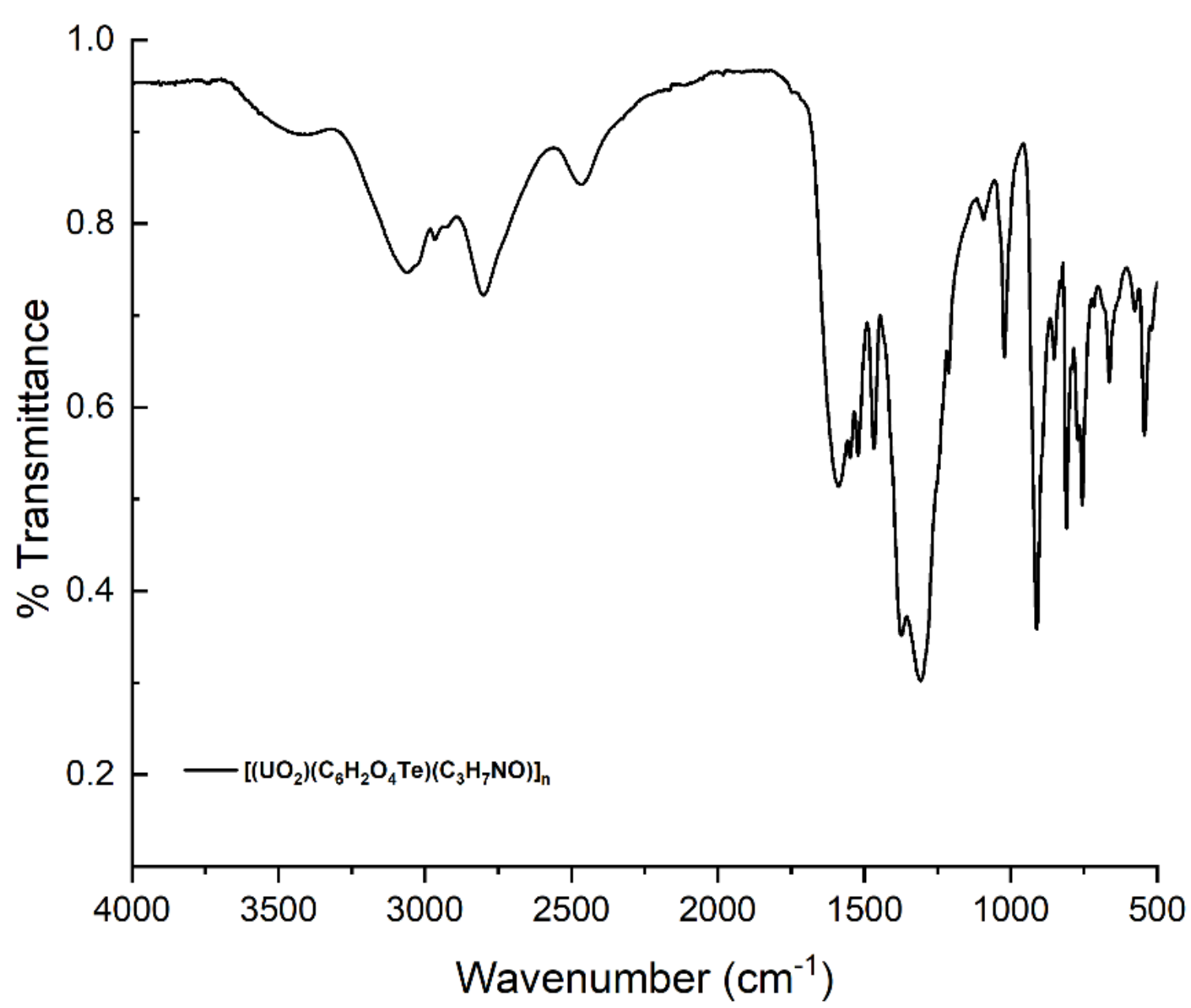

Figure S42. Mid-IR spectrum of compound **2**.

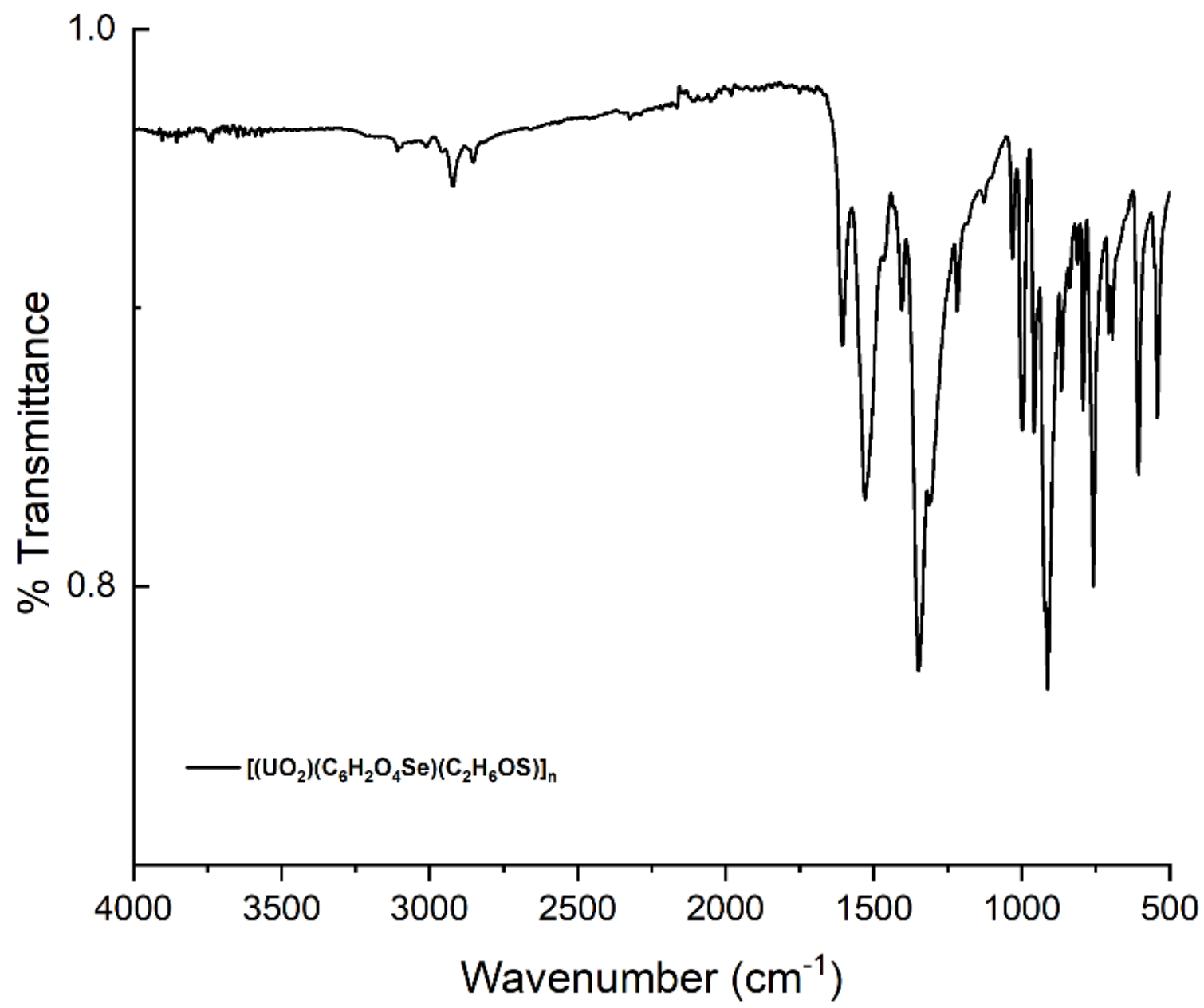

Figure S43. Mid-IR spectrum of compound **3**.

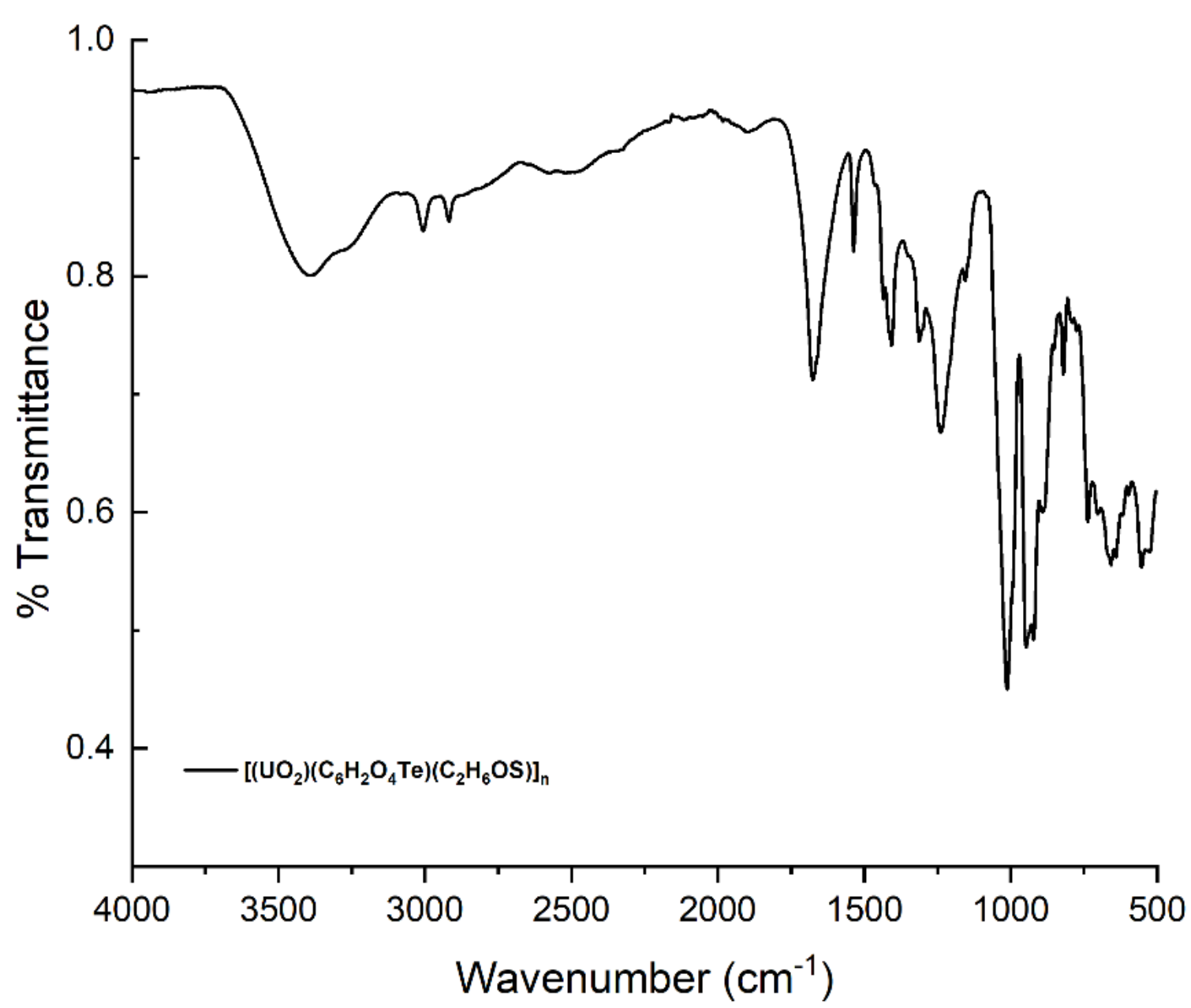

Figure S44. Mid-IR spectrum of compound **4**.

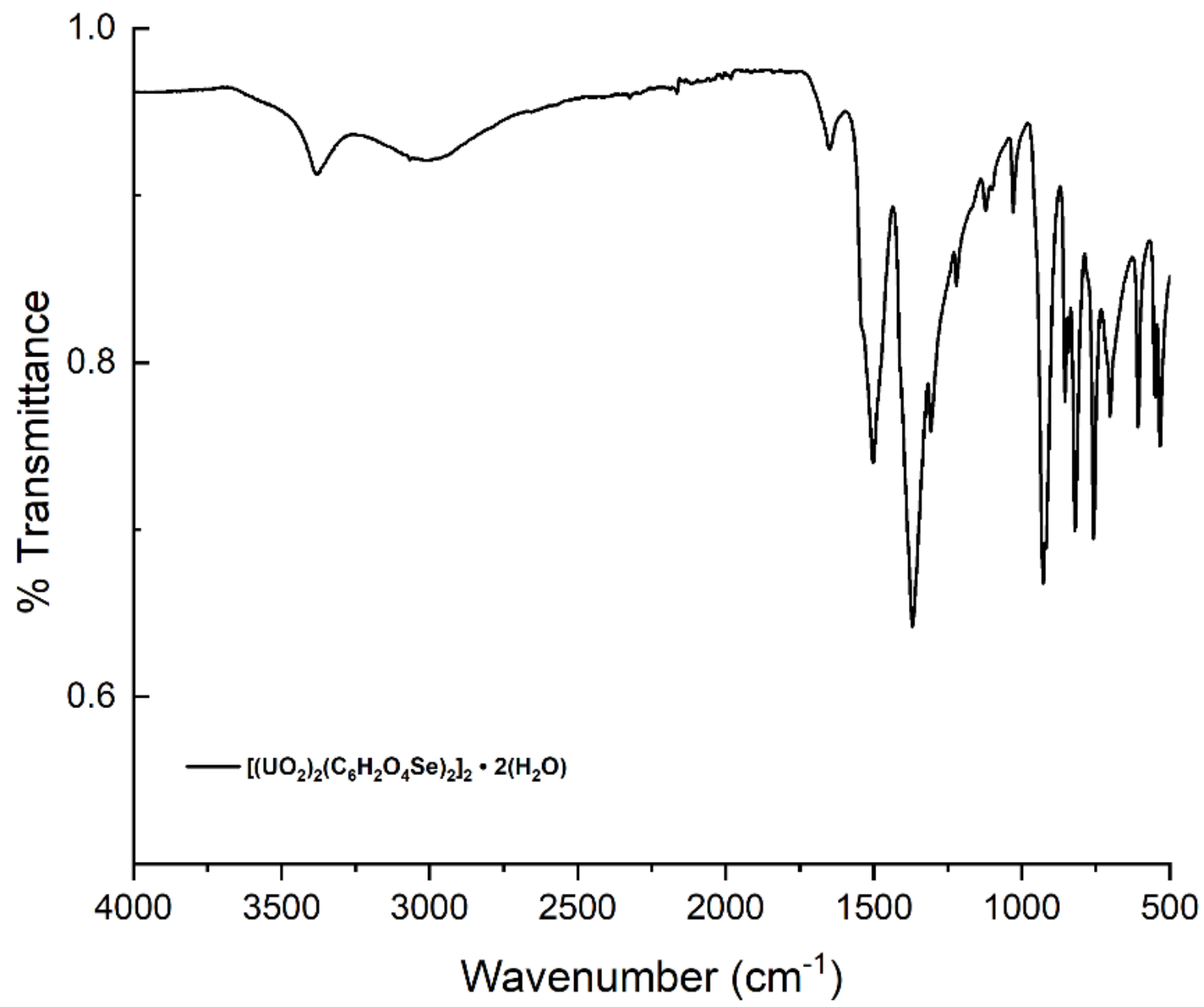

Figure S45. Mid-IR spectrum of compound **5**.

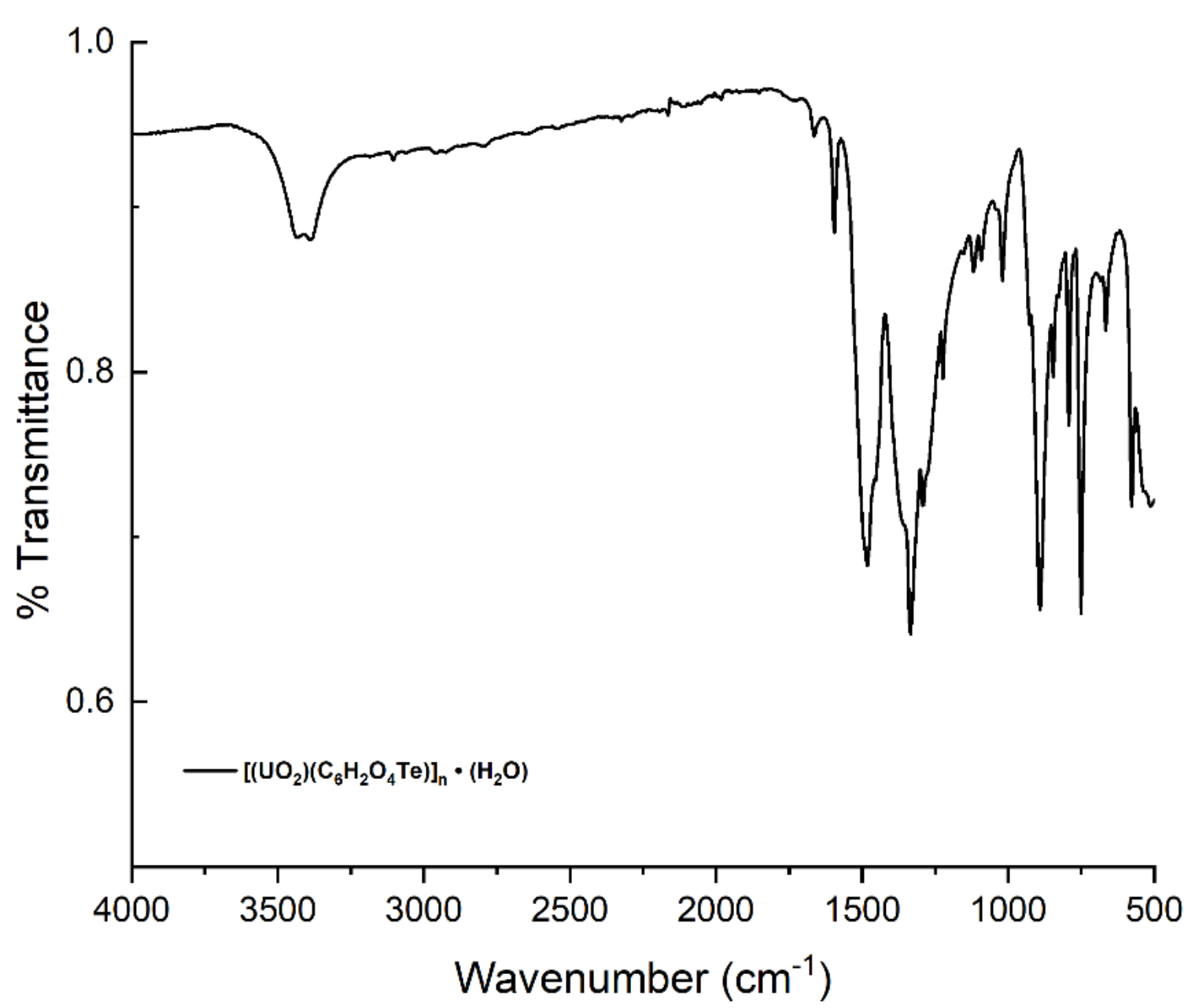

Figure S46. Mid-IR spectrum of compound **6**.

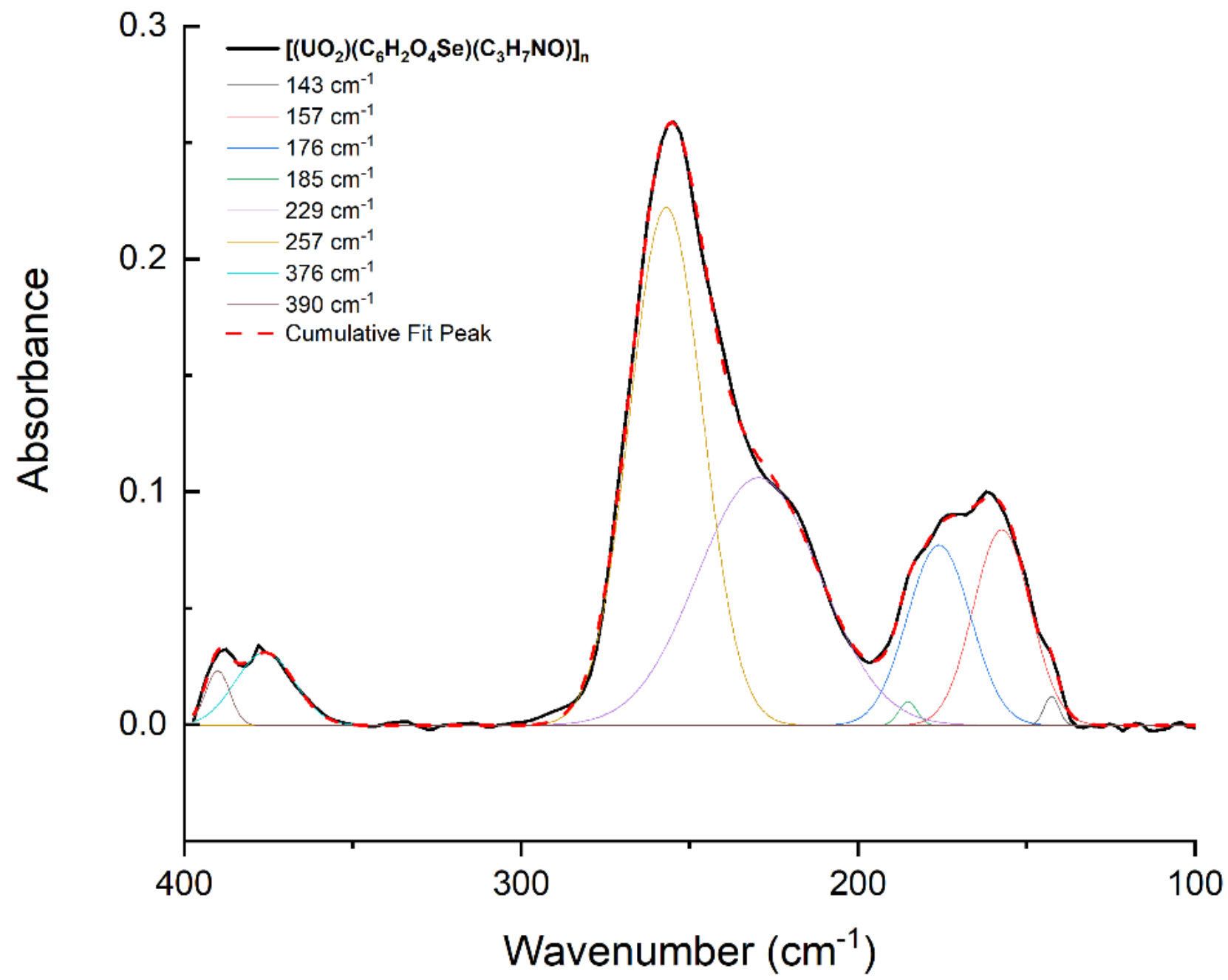

Model	Gauss							
Equation	y=y0 + (A/(w*sqrt(pi/2)))*exp(-2*((x-xc)/w)^2)							
Plot	Peak1	Peak2	Peak3	Peak4	Peak5	Peak6	Peak7	Peak8
xc	143 ± 0	157 ± 1	176 ± 2	185 ± 1	229 ± 1	257 ± 0	376 ± 1	390 ± 0
A	0.06995 ± 0.02527	1.78274 ± 0.28589	1.82095 ± 0.262	0.07066 ± 0.03785	4.86331 ± 0.27351	6.01706 ± 0.24681	0.68711 ± 0.05195	0.21381 ± 0.04215
Reduced Chi-Sqr	4.45E-06							
R-Square (COD)	0.99914							
Adj. R-Square	0.99899							

Figure S47. (**Top**) The fitted far-IR spectrum of compound **1** in the 400–100 cm^{-1} spectral window. (**Bottom**) Fitted spectral features result in a R^2 of 0.999 and a χ^2 of 4.45 x 10^{-6}.

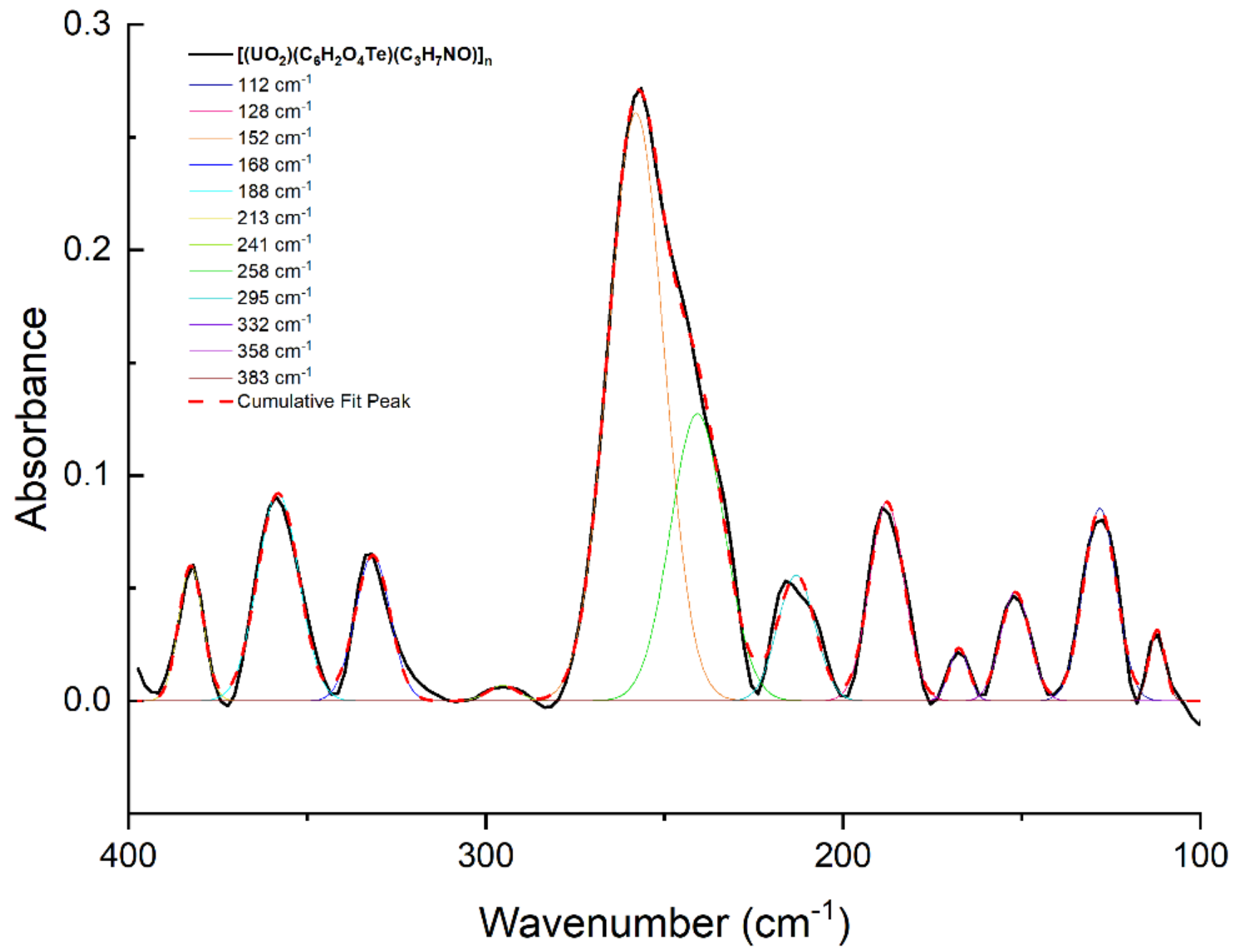

Model	Gauss											
Equation	y=y0 + (A/(w*sqrt(pi/2)))*exp(-2*((x-xc)/w)^2)											
Plot	Peak1	Peak2	Peak3	Peak4	Peak5	Peak6	Peak7	Peak8	Peak9	Peak10	Peak11	Peak12
xc	112 ± 0	128 ± 0	152 ± 0	168 ± 1	188 ± 0	213 ± 0	241 ± 1	258 ± 0	295 ± 2	332 ± 0	358 ± 0	383 ± 0
A	0.16838 ± 0.02345	0.99274 ± 0.0344	0.49589 ± 0.03236	0.15567 ± 0.02613	1.07171 ± 0.03499	0.73054 ± 0.03861	2.400975 ± 0.25181	5.10723 ± 0.24942	0.071341 ± 0.03239	0.7889 ± 0.0351	1.33497 ± 0.03827	0.51064 ± 0.02919
Reduced Chi-Sqr	2.44E-05											
R-Square (COD)	0.99493											
Adj. R-Square	0.99345											

Figure S48. (**Top**) The fitted far-IR spectrum of compound **2** in the 400–100 cm^{-1} spectral window. (**Bottom**) Fitted spectral features result in a R^2 of 0.994 and a χ^2 of 2.44 x 10^{-5}.

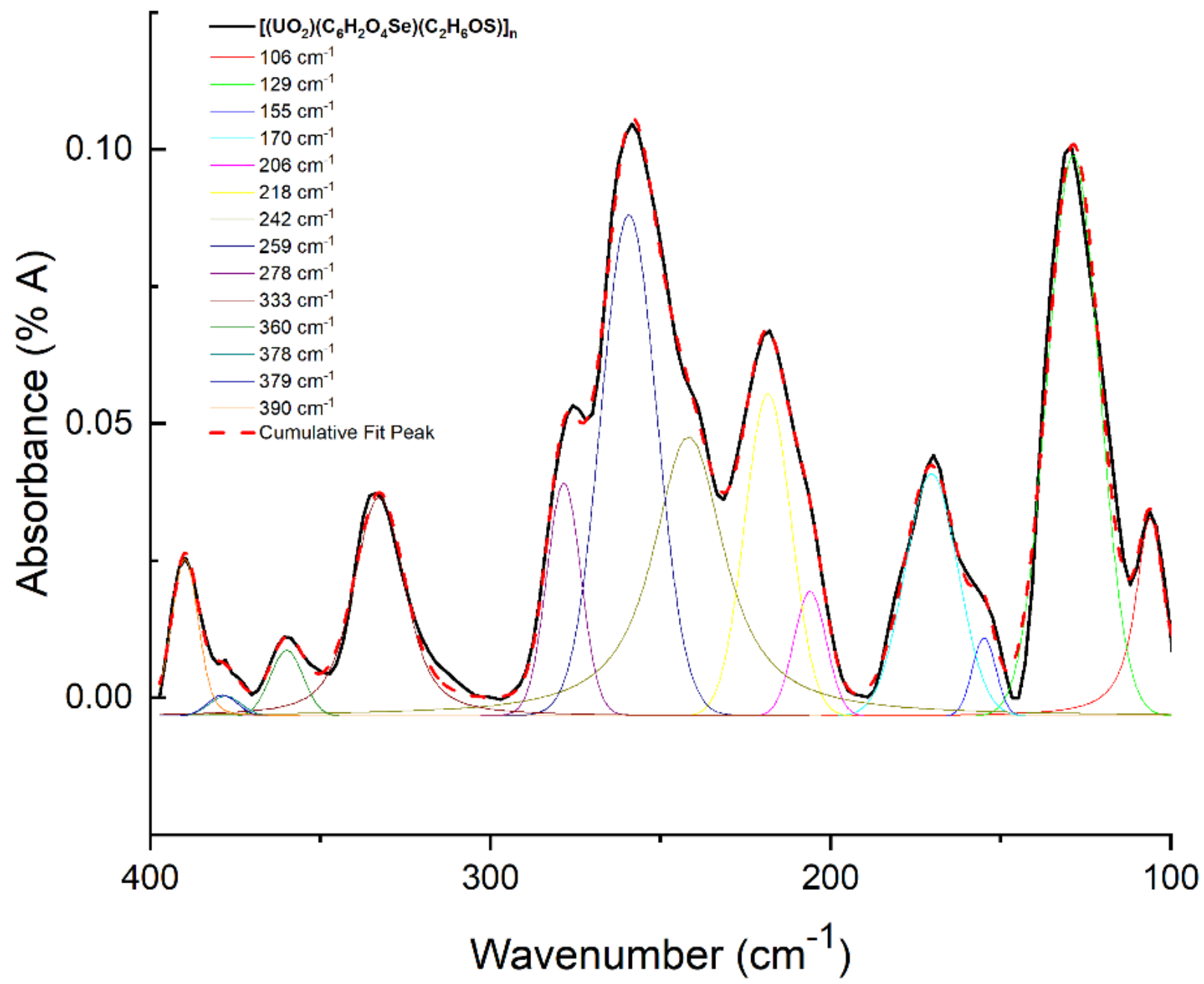

Model	Voigt													
Equation	y = nlf_voigt(x,y0,xc,A,wG,wL);													
Plot	Peak1	Peak2	Peak3	Peak4	Peak5	Peak6	Peak7	Peak8	Peak9	Peak10	Peak11	Peak12	Peak13	Peak14
xc	106 ± 0	129 ± 0	155 ± 1	170 ± 0	206 ± 3	218 ± 2	242 ± 6	259 ± 5	278 ± 2	333 ± 1	360 ± 18	378 ± 43113	379 ± 8420	390 ± 17
A	0.55647 ± 0.394	1.96409 ± 0.18057	0.1246 ± 0.05699	0.86691 ± 0.77352	0.2736 ± 2.01915	1.00263 ± 8.57591	1.92133 ± 19.66181	1.89849 ± 12.01033	0.54833 ± 1.7387	0.92811 ± 0.7311	0.14591 ± 1.82887	0.04396 ± 2633.97965	0.04296 ± 2646.53977	0.3092 ± 10.5845
Reduced Chi-Sqr	8.78E-06													
R-Square (COD)	0.99311													
Adj. R-Square	0.98921													

Figure S49. (**Top**) The fitted far-IR spectrum of compound **3** in the 400–100 cm^{-1} spectral window. (**Bottom**) Fitted spectral features result in a R^2 of 0.993 and a χ^2 of 8.78 x 10^{-6}.

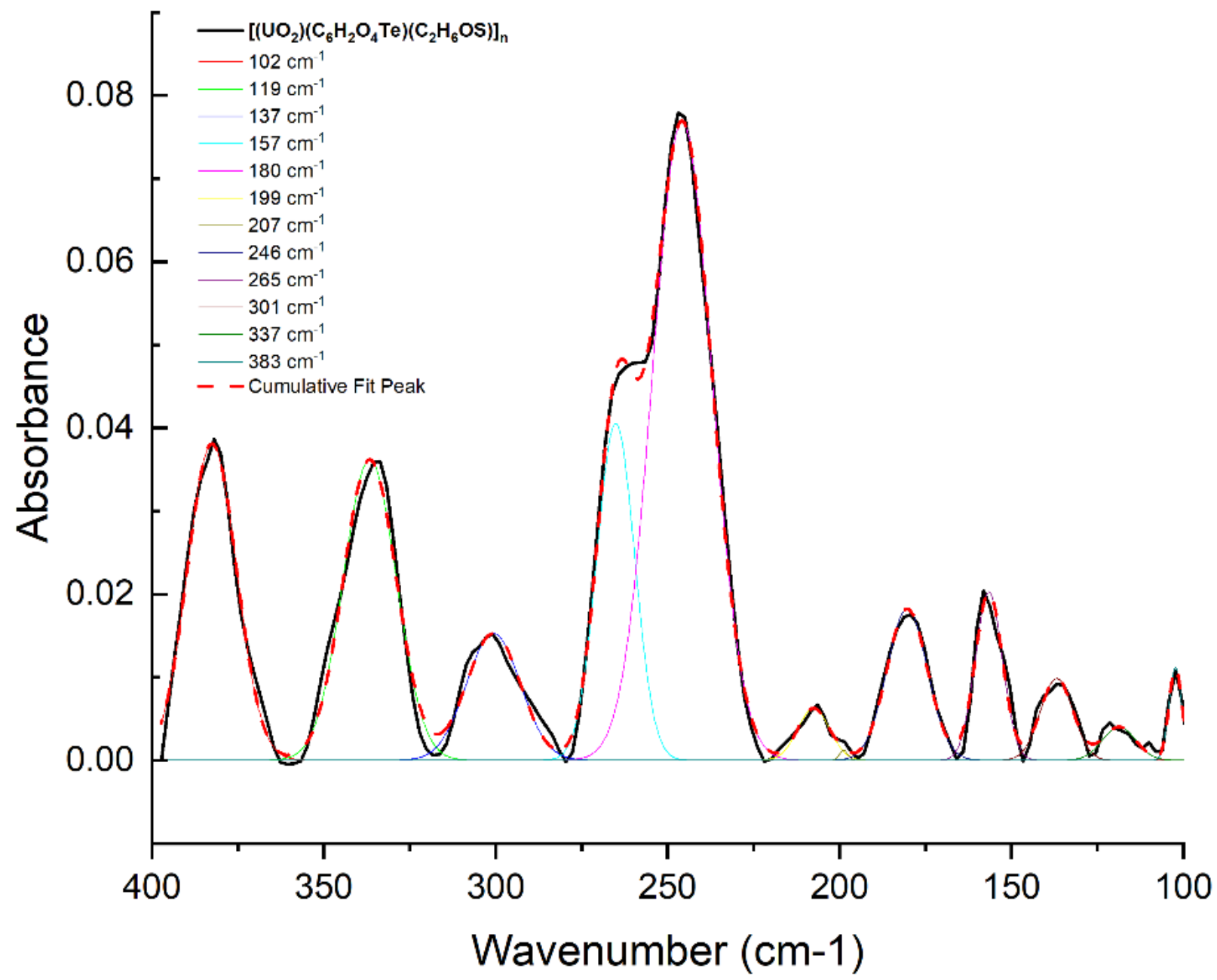

Model	Gaussian											
Equation	y = y0 + A/(w*sqrt(pi/(4*ln(2)))) * exp(-4*ln(2)*(x-xc)^2/w^2)											
Plot	Peak1	Peak2	Peak3	Peak4	Peak5	Peak6	Peak7	Peak8	Peak9	Peak10	Peak11	Peak12
xc	102 ± 0	119 ± 1	137 ± 1	157 ± 0	180 ± 0	199 ± 3	207 ± 1	246 ± 0	265 ± 0	301 ± 0	337 ± 0	383 ± 0
A	0.05195 ± 0.00702	0.04878 ± 0.01258	0.1164 ± 0.01257	0.20035 ± 0.01042	0.26861 ± 0.01253	0.00367 ± 0.00836	0.07258 ± 0.01376	1.66714 ± 0.02917	0.56111 ± 0.02663	0.29939 ± 0.0145	0.69984 ± 0.01438	0.68198 ± 0.01385
Reduced Chi-Sqr	2.56E-06											
R-Square (COD)	0.99383											
Adj. R-Square	0.99203											

Figure S50. (**Top**) The fitted far-IR spectrum of compound **4** in the 400–100 cm^{-1} spectral window. (**Bottom**) Fitted spectral features result in a R^2 of 0.993 and a χ^2 of 2.56 x 10^{-6}.

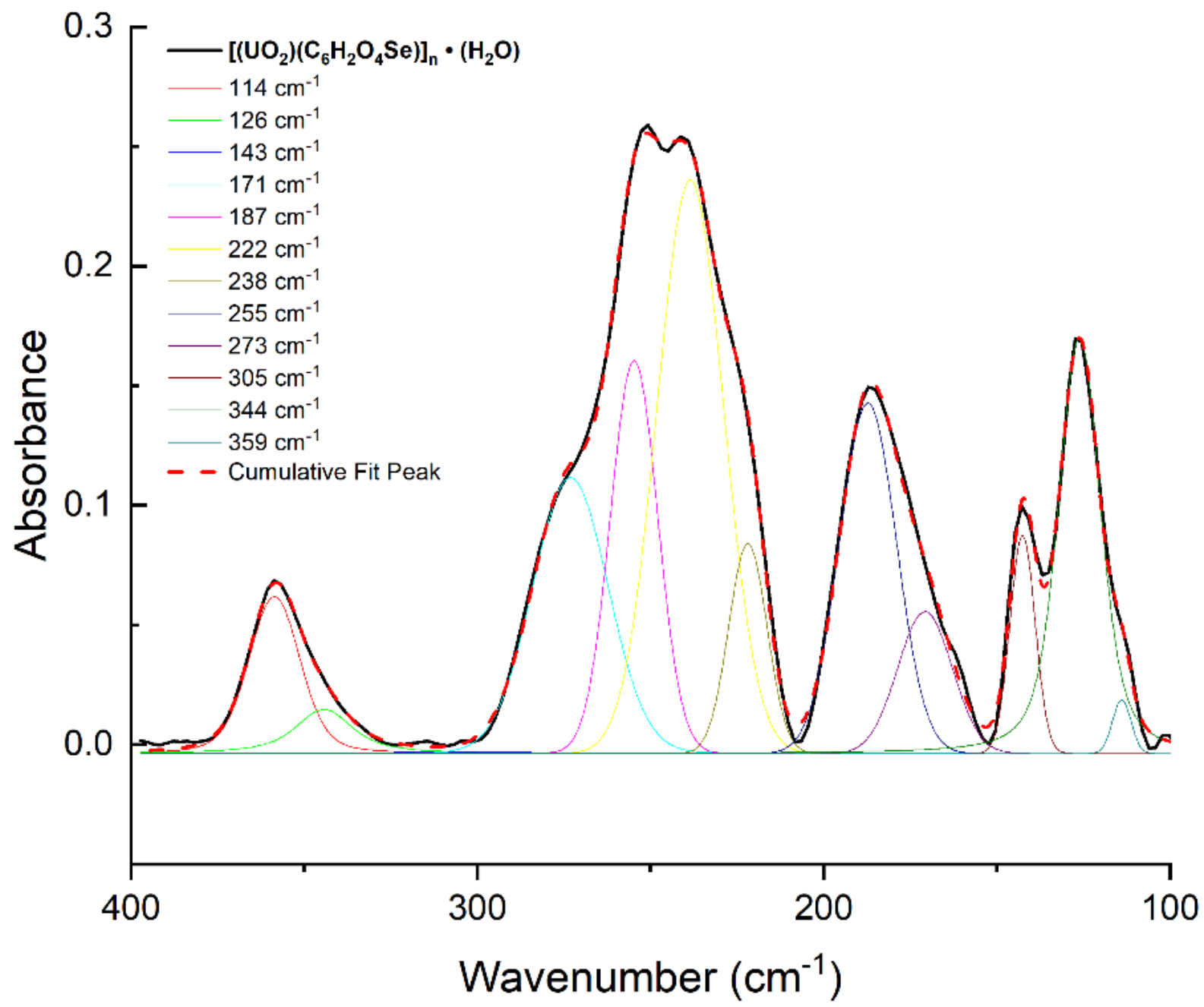

Model	Voigt											
Equation	y = nlf_voigt(x,y0,xc,A,wG,wL);											
Plot	Peak1	Peak2	Peak3	Peak4	Peak5	Peak6	Peak7	Peak8	Peak9	Peak10	Peak11	Peak12
xc	114 ± 0	126 ± 0	143 ± 0	171 ± 2	187 ± 1	222 ± 1	238 ± 2	255 ± 1	273 ± 5	305 ± 8552	344 ± 6	359 ± 1
A	0.15107 ± 0.06889	3.17439 ± 0.72602	0.83102 ± 0.08116	1.17252 ± 0.30431	3.02409 ± 0.33892	1.2172 ± 0.69596	5.76462 ± 2.53475	2.82352 ± 4.16188	3.18988 ± 22.40588	0.0469 ± 24.37027	0.52212 ± 4.64229	1.35009 ± 0.66261
Reduced Chi-Sqr	9.52E-06											
R-Square (COD)	0.99886											
Adj. R-Square	0.99834											

Figure S51. (**Top**) The fitted far-IR spectrum of compound **5** in the 400–100 cm^{-1} spectral window. (**Bottom**) Fitted spectral features result in a R^2 of 0.998 and a χ^2 of 9.52 x 10^{-6}.

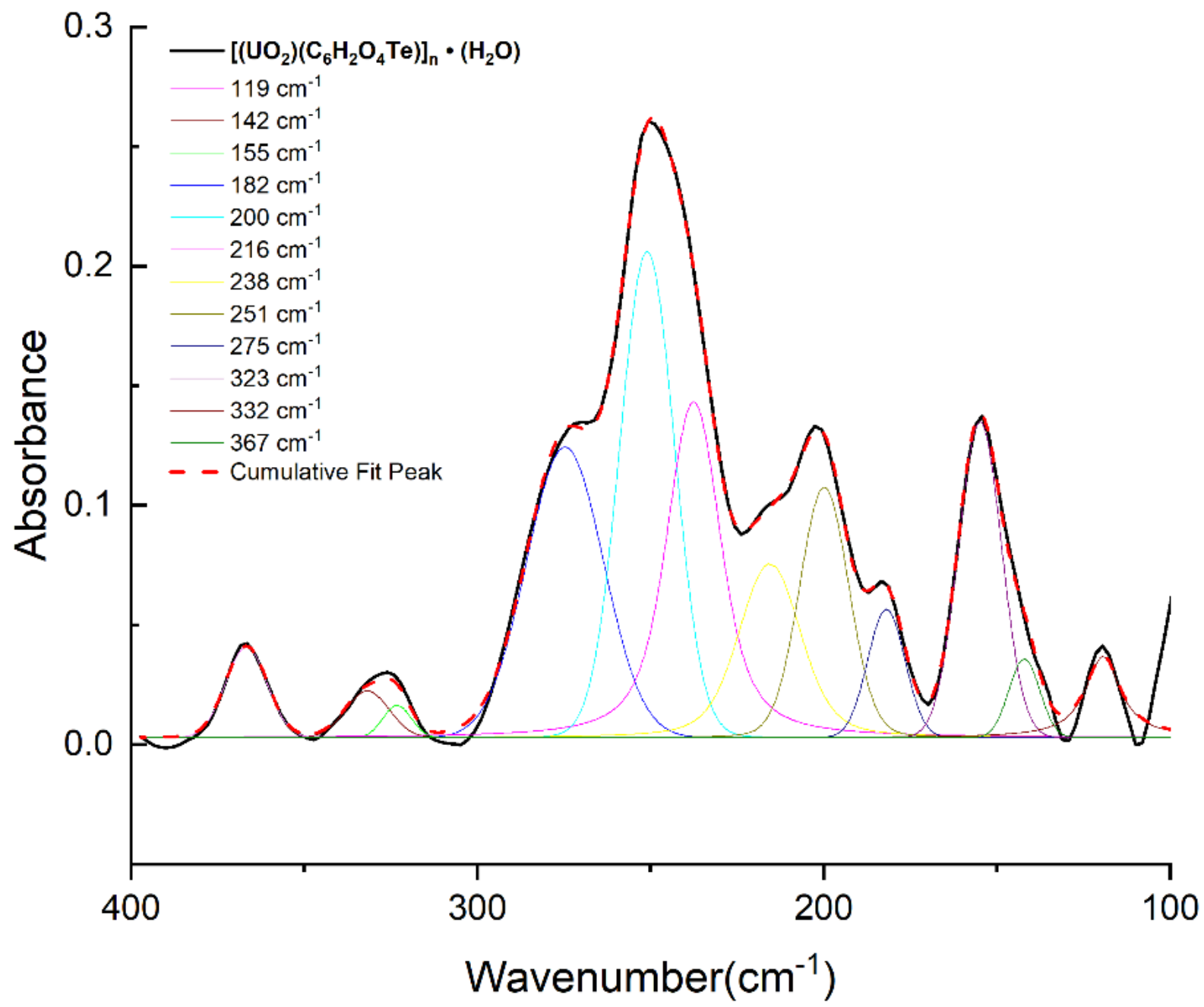

Model	Voigt											
Equation	y = nlf_voigt(x,y0,xc,A,wG,wL);											
Plot	Peak1	Peak2	Peak3	Peak4	Peak5	Peak6	Peak7	Peak8	Peak9	Peak10	Peak11	Peak12
xc	119 ± 1	142 ± 3	155 ± 1	182 ± 9	200 ± 10	216 ± 22	238 ± 6	251 ± 7	275 ± 12	323 ± 11	332 ± 25	367 ± 1
A	0.65002 ± 0.47771	0.3679 ± 0.37704	2.07132 ± 0.69776	0.7266 ± 6.60428	1.94007 ± 47.12802	1.87269 ± 80.77589	3.45891 ± 50.72599	4.05155 ± 23.66245	3.38848 ± 5.34134	0.13899 ± 1.11647	0.30961 ± 0.97583	0.56947 ± 0.4366
Reduced Chi-Sqr	8.96E-05											
R-Square (COD)	0.98628											
Adj. R-Square	0.98012											

Figure S52. (**Top**) The fitted far-IR spectrum of compound **6** in the 400–100 cm^{-1} spectral window. (**Bottom**) Fitted spectral features result in a R^2 of 0.986 and a χ^2 of 8.96 x 10^{-5}.

Thermogravimetric Analysis (TGA)

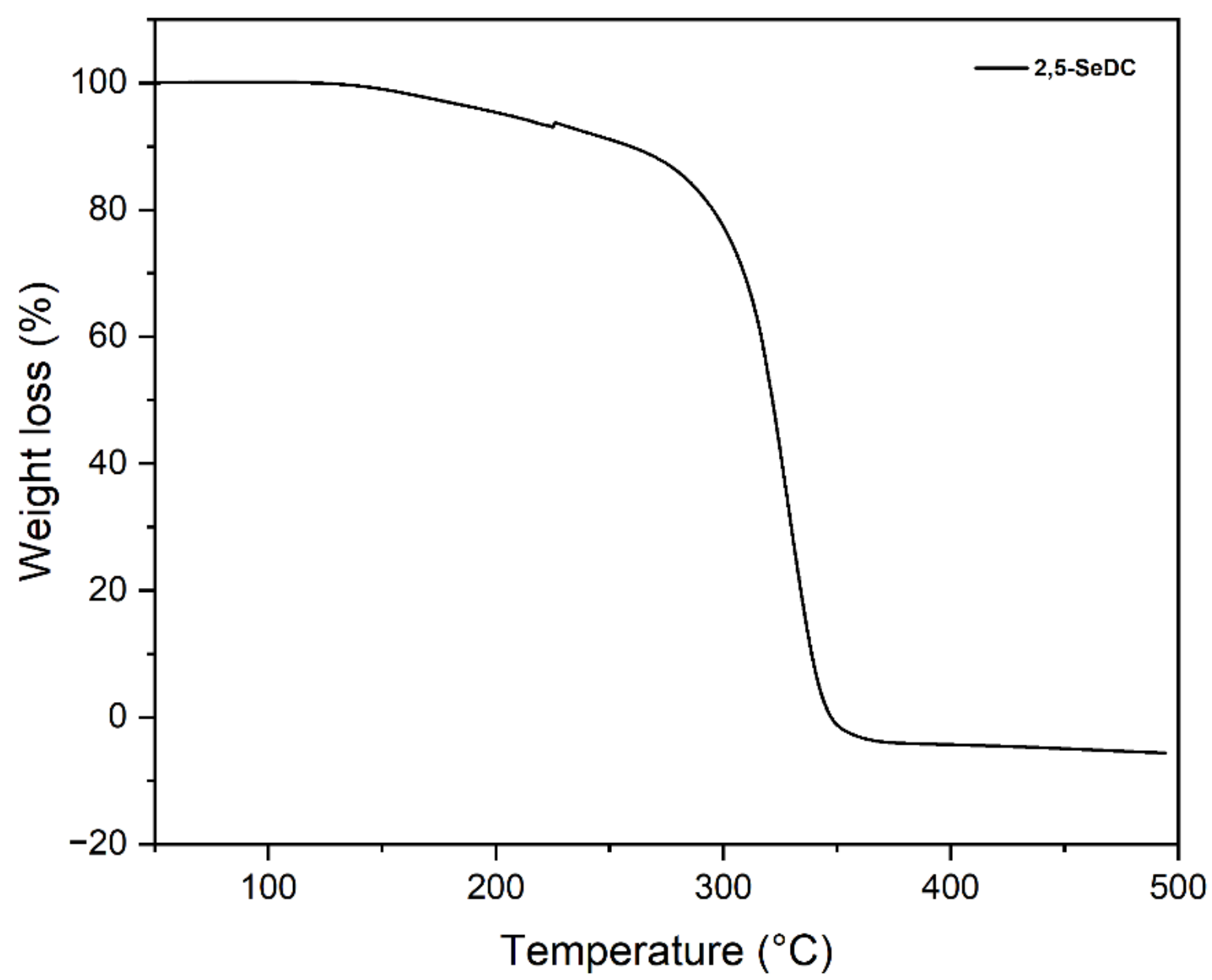

Figure S53. TGA curve for 2,5-SeDC.

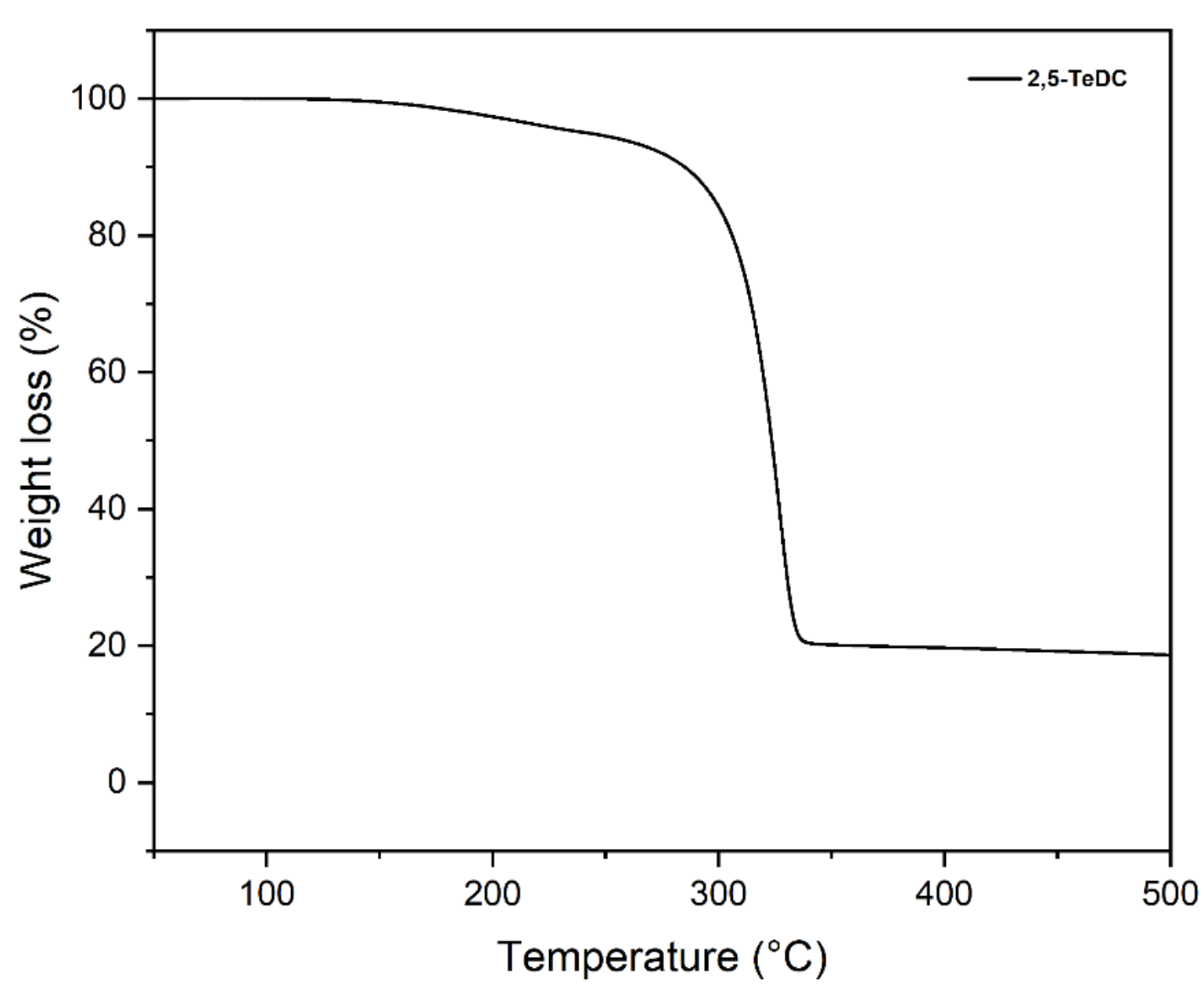

Figure S54. TGA curve for 2,5-TeDC.

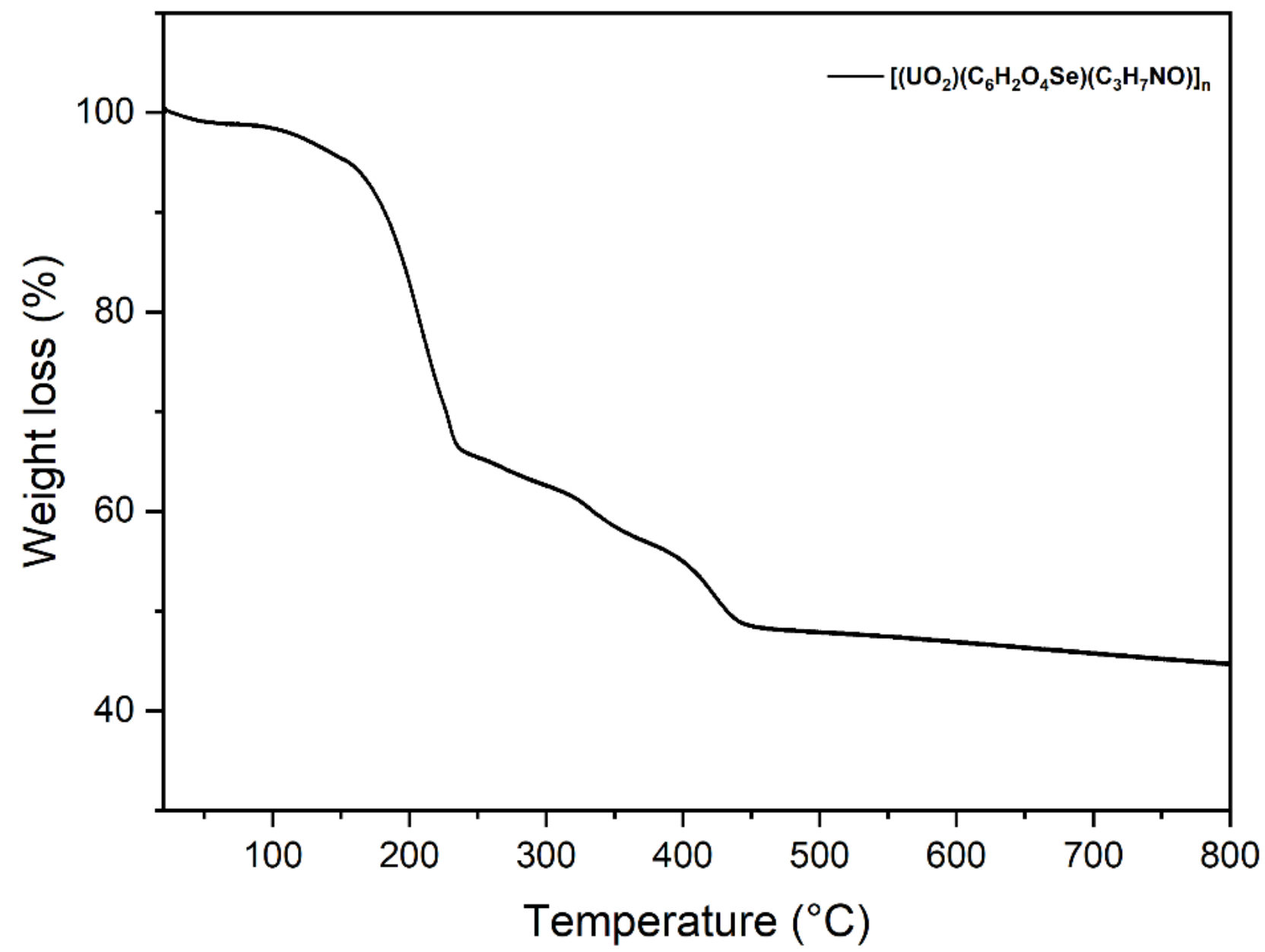

Figure S55. TGA curve for compound **1**, $[(UO_2)(C_6H_2O_4Se)(C_3H_7NO)]_n$.

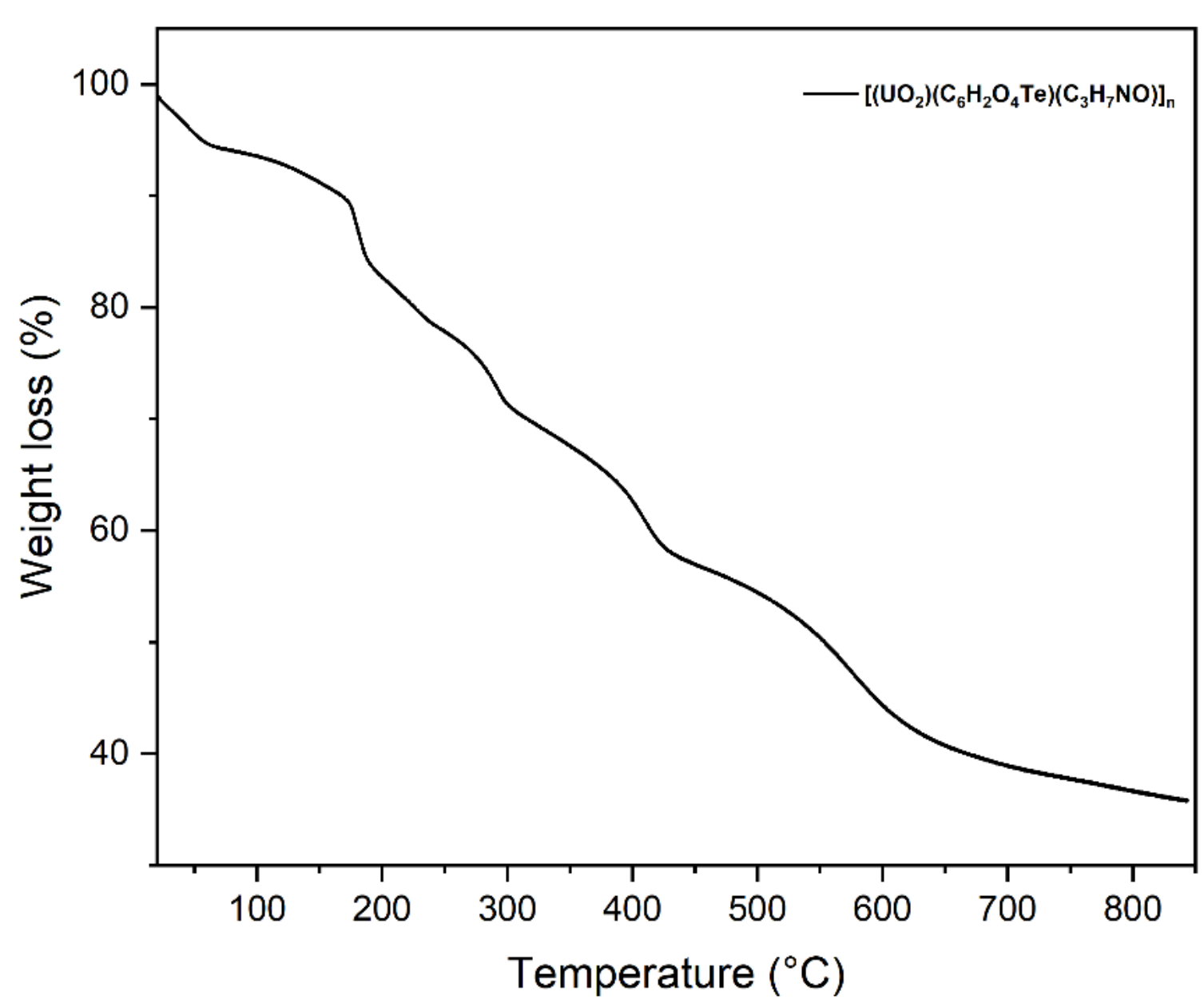

Figure S56. TGA curve for compound **2**, $[(UO_2)(C_6H_2O_4Te)(C_3H_7NO)]_n$.

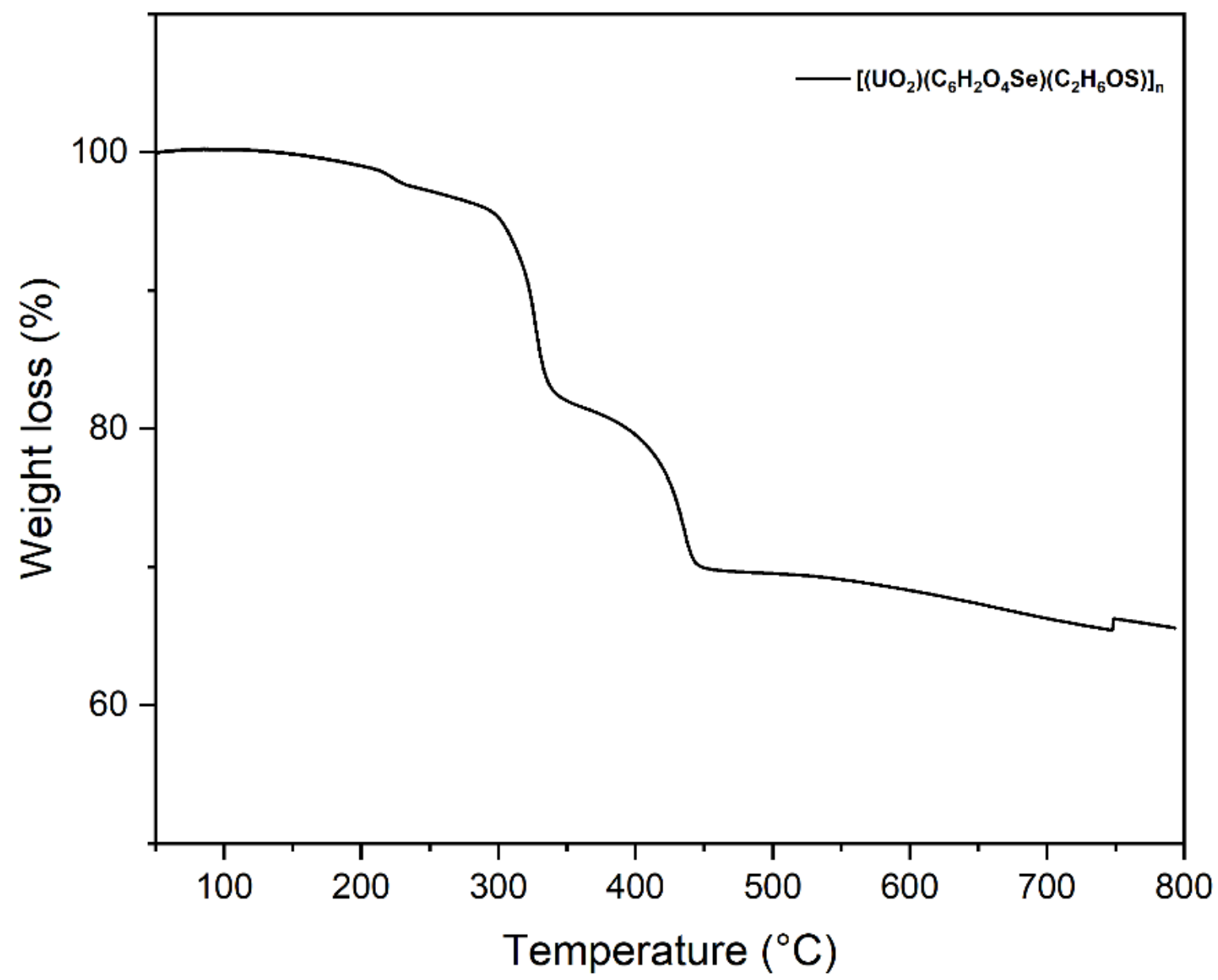

Figure S57. TGA curve for compound **3**, $[(UO_2)(C_6H_2O_4Se)(C_2H_6OS)]_n$.

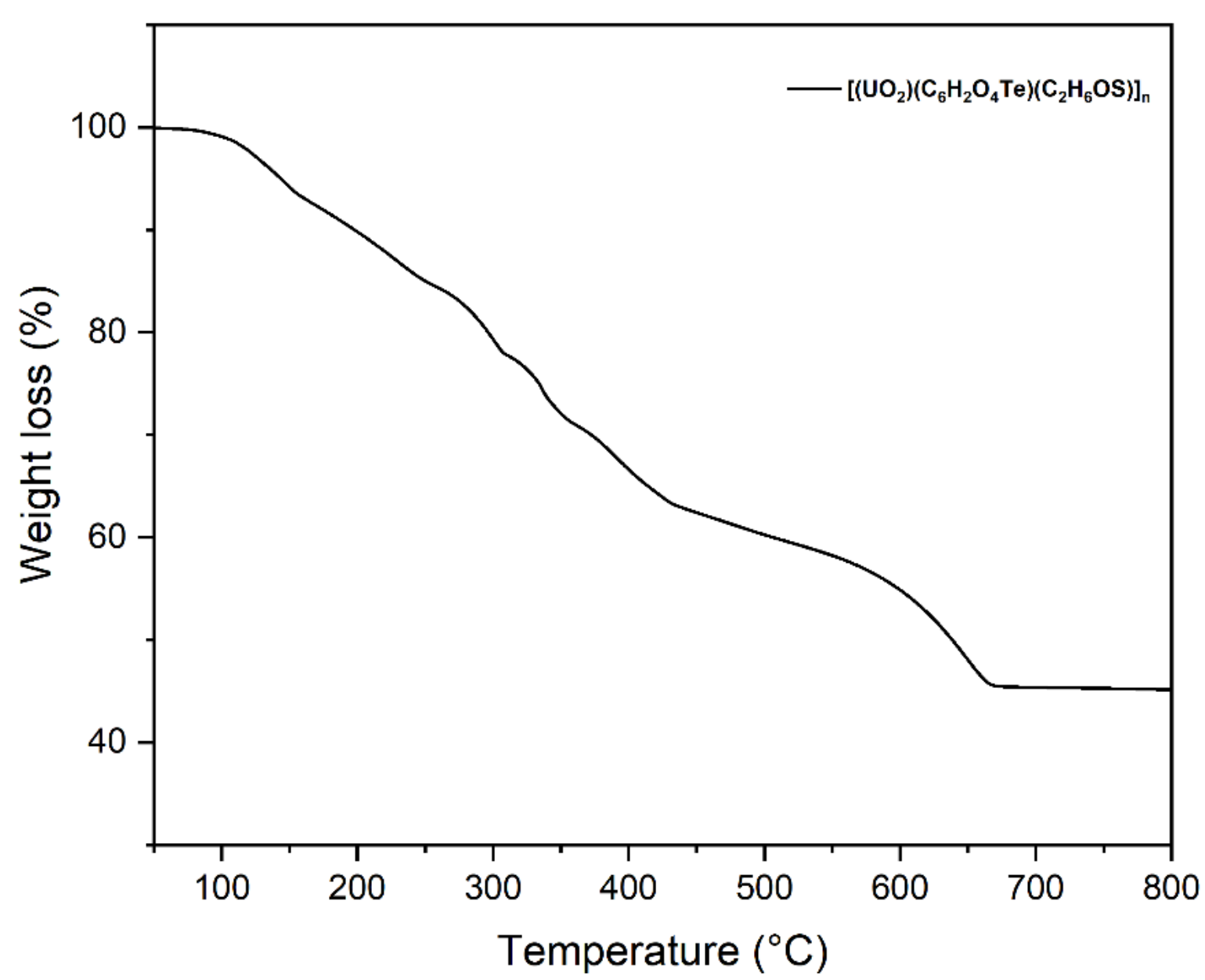

Figure S58. TGA curve for compound **4,** $[(UO_2)(C_6H_2O_4Te)(C_2H_6OS)]_n$.

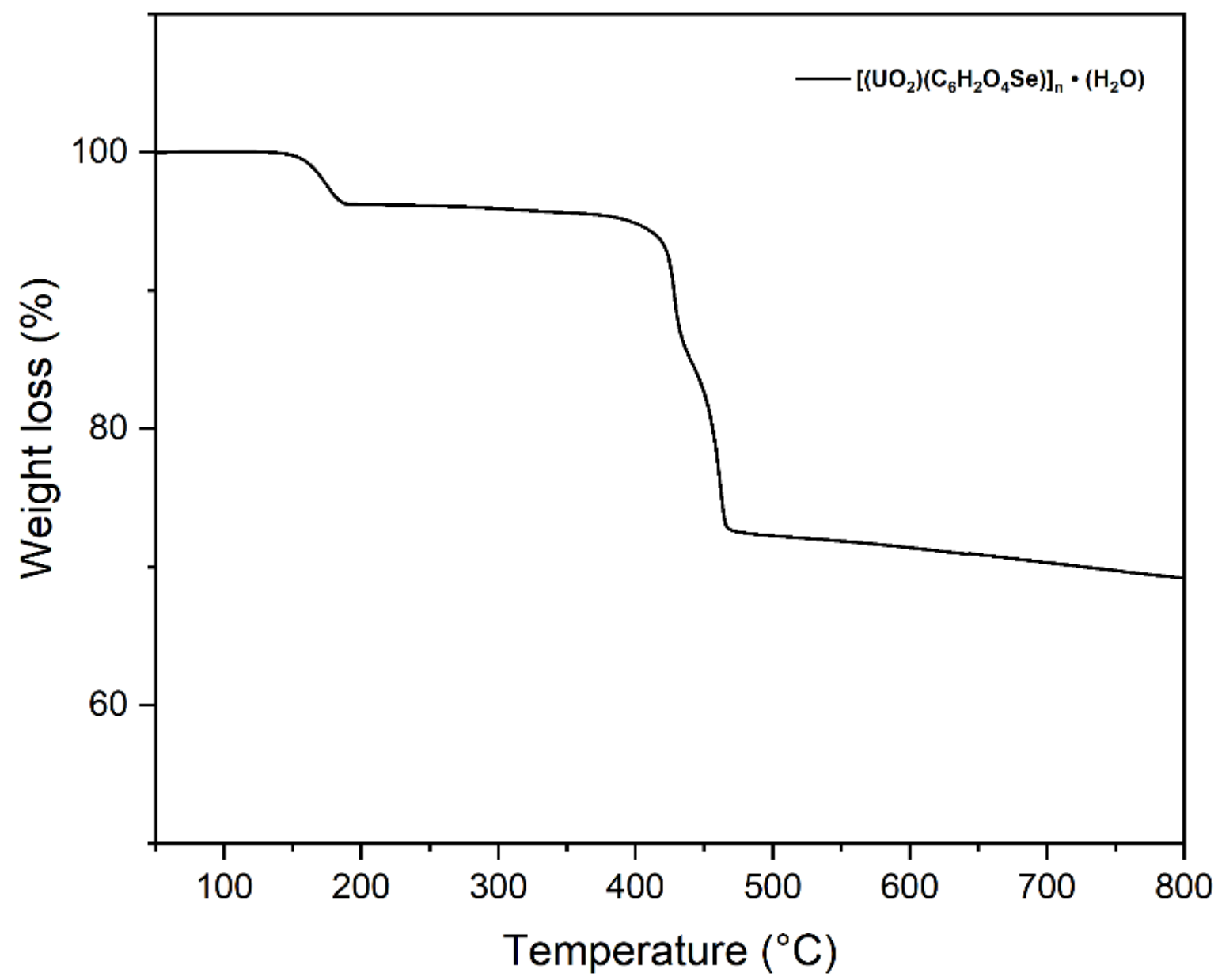

Figure S59. TGA curve for compound **5**, $[(UO_2)_2(C_6H_2O_4Se)_2]_n{\bullet}2H_2O$.

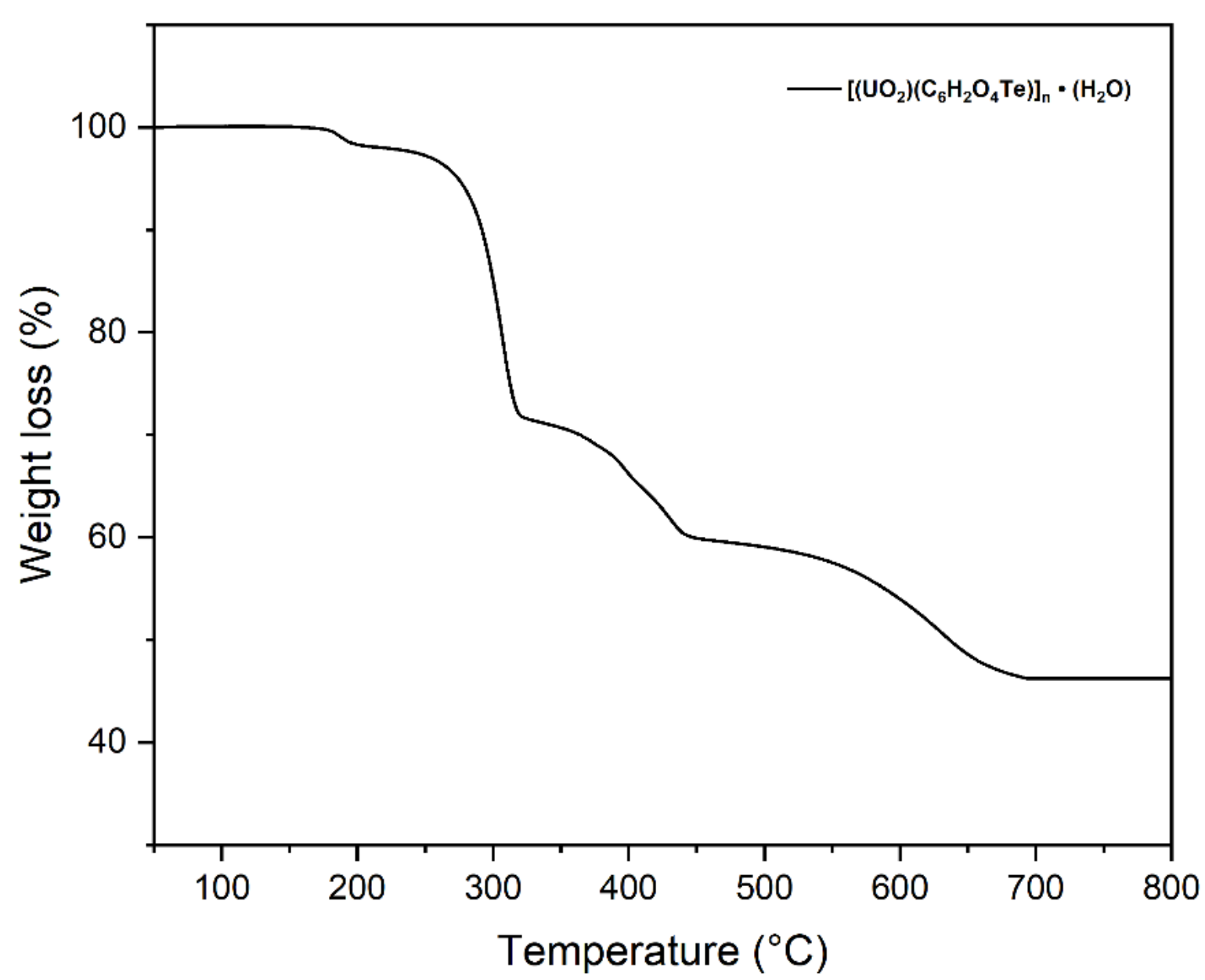

Figure S60. TGA curve for compound **6**, $[(UO_2)(C_6H_2O_4Te)]_n{\bullet}H_2O$.

Scanning Electron Microscopy (SEM) and Energy Dispersive Spectroscopy (EDS)

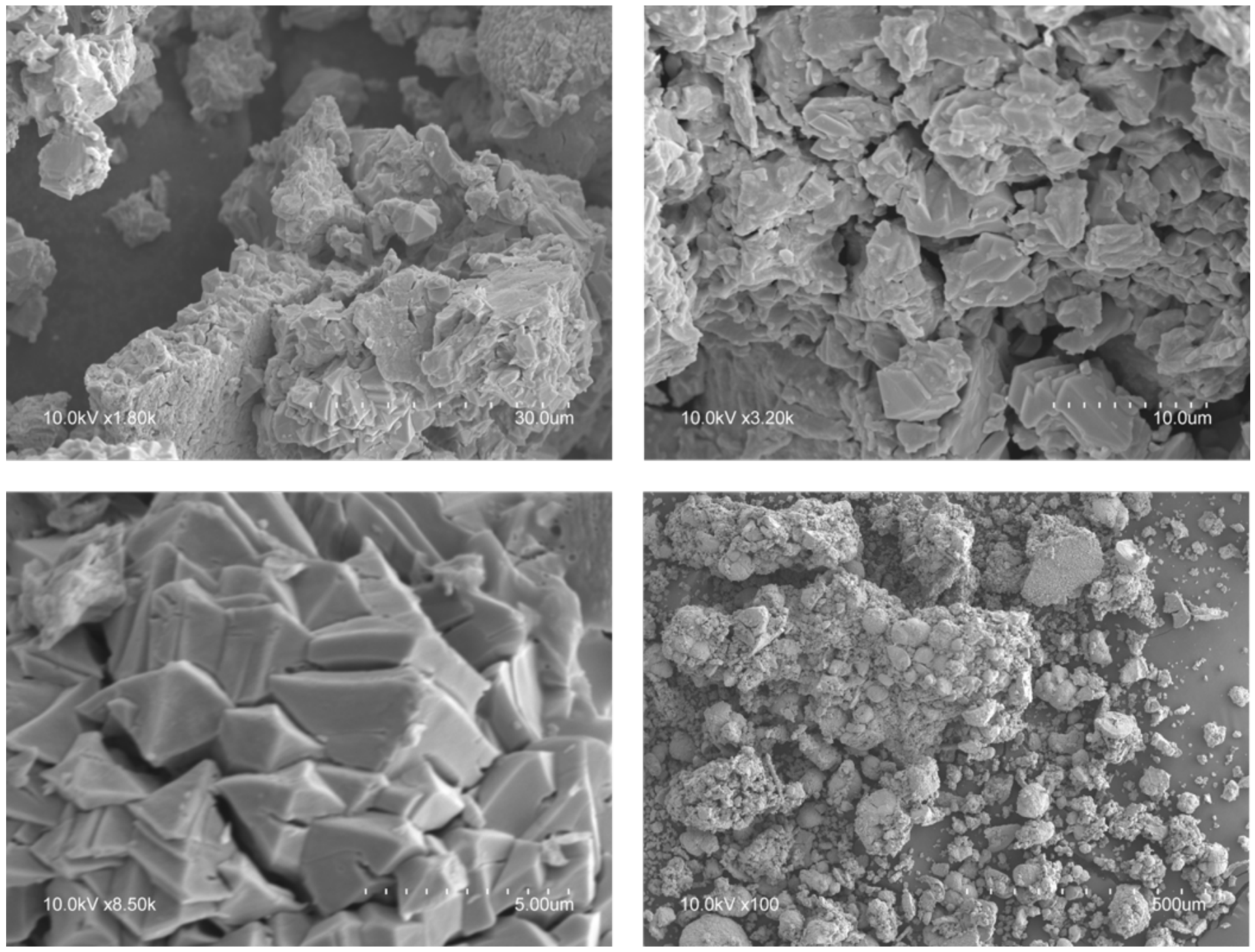

Figure S61. SEM images of compound **3** at different magnification **(Top Left)** 1.80k, **(Top Right)** 3.20k, **(Bottom Left)** 8.50k, and **(Bottom Right)** 100k.

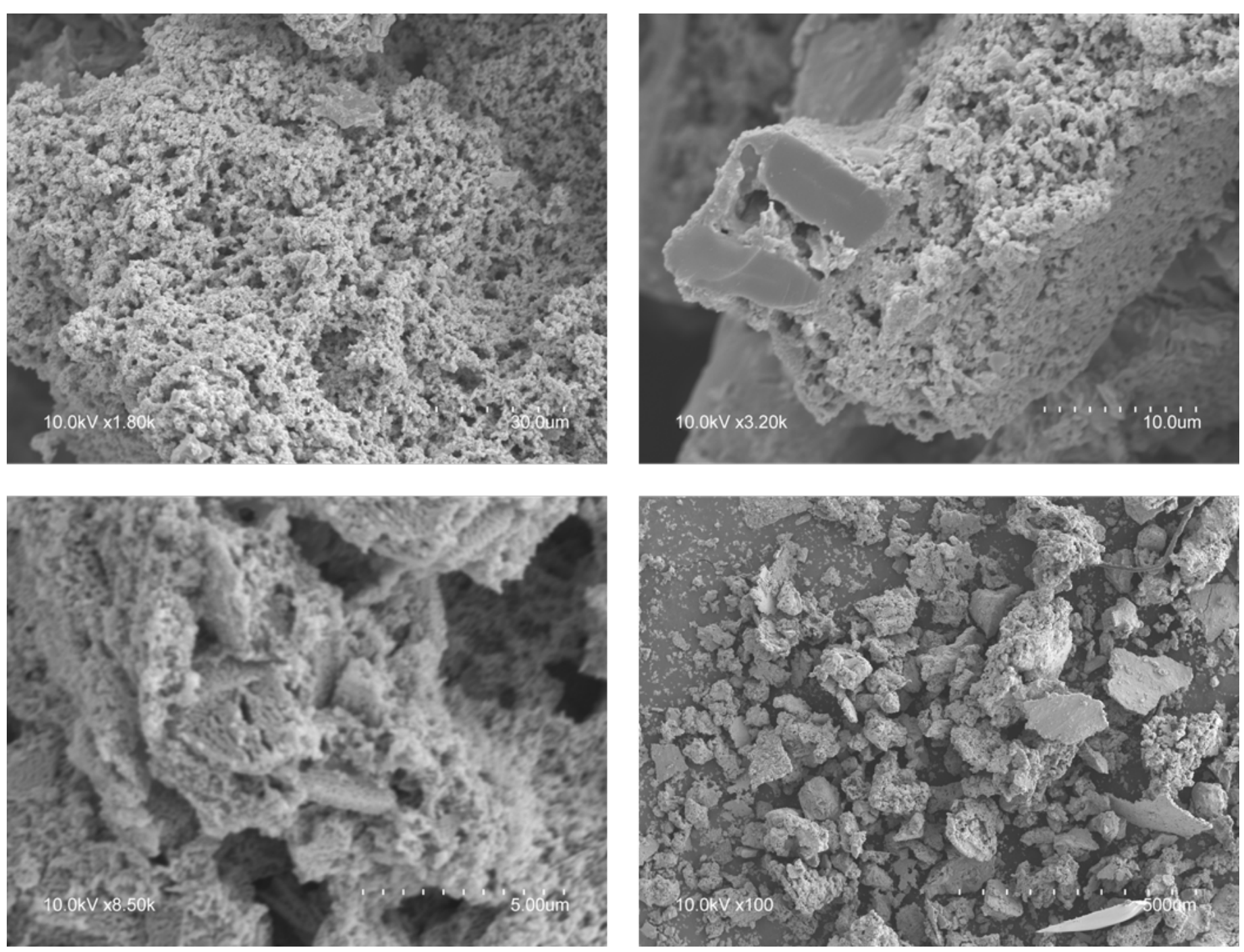

Figure S62. SEM images of compound **4** at different magnification **(Top Left)** 1.80k, **(Top Right)** 3.20k, **(Bottom Left)** 8.50k, and **(Bottom Right)** 100k.

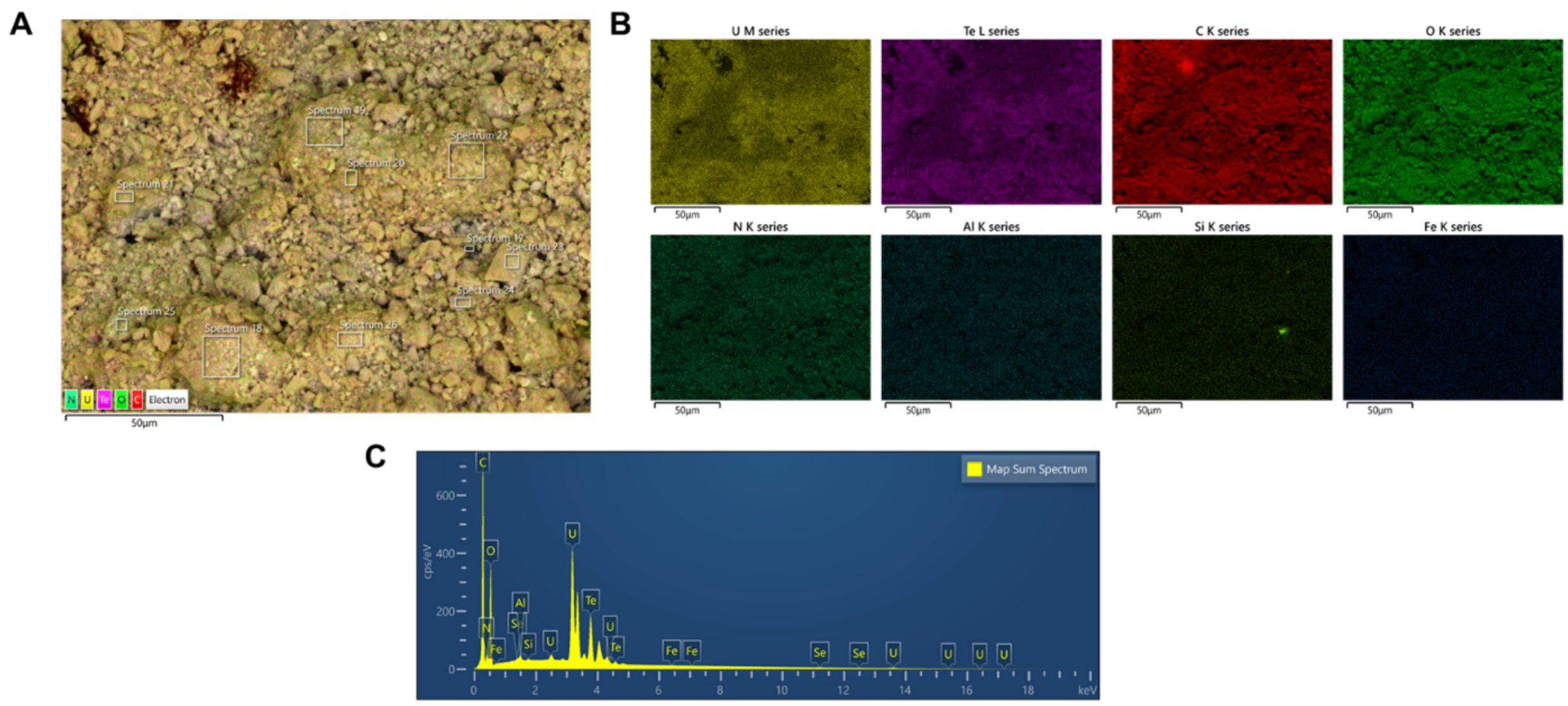

Map Sum Spectrum

Element	Line Type	Weight %	Weight % Sigma	Atomic %
C	K series	28.25	0.03	57.61
N	K series	3.86	0.06	6.74
O	K series	18.85	0.03	28.85
Al	K series	0.35	0.01	0.32
Si	K series	0.08	0.00	0.07
Fe	K series	0.05	0.01	0.02
Se	L series	0.34	0.01	0.10
Te	L series	14.91	0.02	2.86
U	M series	33.32	0.03	3.43
Total		100.00		100.00

Figure S63. Pre-TGA SEM/EDS analysis of compound **2**. **(a)** Overall elemental mapping via FE-SEM/EDS imaging, **(b)** the distribution of specific elements in the elemental maps, and **(c)** map sum spectrum. Material was placed on aluminum stub with double-sided carbon tape and not coated prior to the analysis.

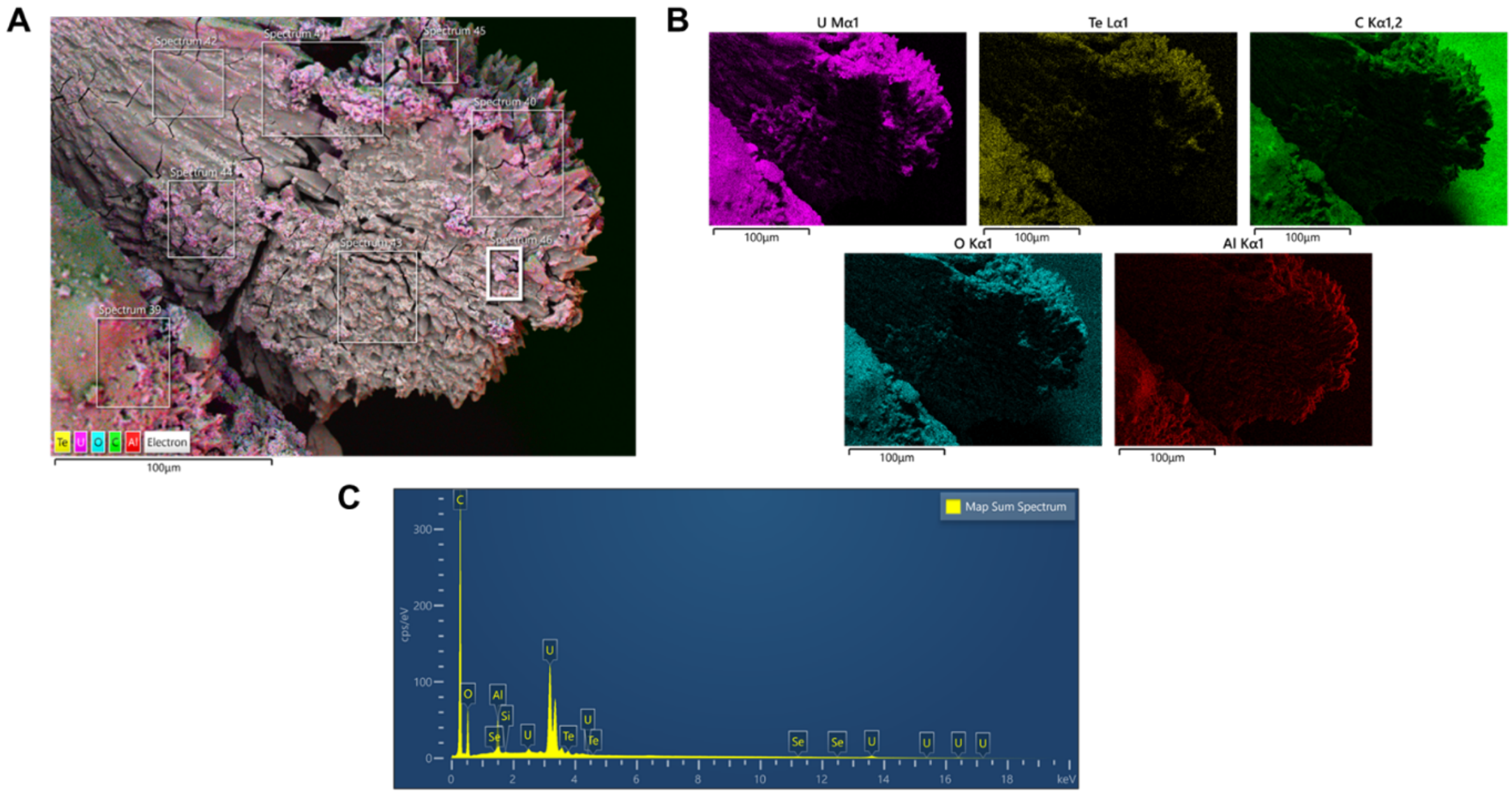

Map Sum Spectrum Element	Line Type	Weight %	Weight % Sigma	Atomic %
C	K series	45.77	0.05	76.52
O	K series	14.42	0.05	18.10
Al	K series	2.82	0.01	2.10
Si	K series	0.06	0.01	0.04
Se	L series	0.16	0.02	0.04
Te	L series	1.37	0.02	0.21
U	M series	35.41	0.05	2.99
Total		100.00		100.00

Figure S64. Post-TGA SEM/EDS analysis of compound **2**. **(a)** Overall elemental mapping via FE-SEM/EDS imaging, **(b)** the distribution of specific elements in the elemental mapping, and **(c)** map sum spectrum. Material was placed on aluminum stub with double-sided carbon tape and not coated prior to the analysis.

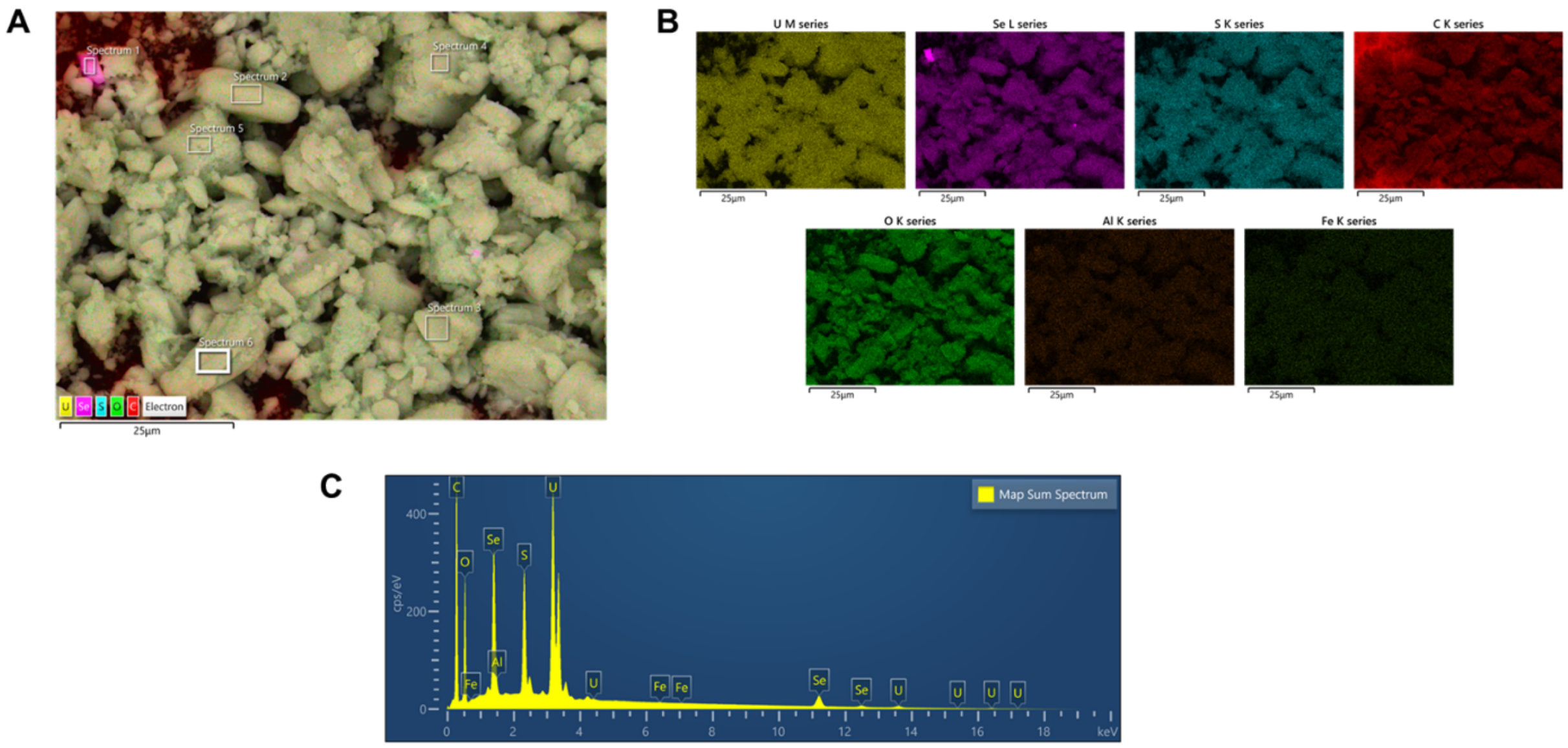

Map Sum Spectrum Element	Line Type	Weight %	Weight % Sigma	Atomic %
C	K series	32.57	0.03	65.70
O	K series	15.69	0.02	23.77
Al	K series	0.38	0.01	0.34
S	K series	4.35	0.01	3.29
Fe	K series	0.05	0.01	0.02
Se	L series	10.23	0.02	3.14
U	M series	36.73	0.03	3.74
Total		100.00		100.00

Figure S65. Pre-TGA SEM/EDS analysis of compound **3**. **(a)** Overall elemental mapping via FE-SEM/EDS imaging, **(b)** the distribution of specific elements in the elemental mapping, and **(c)** map sum spectrum. Material was placed on aluminum stub with double-sided carbon tape and not coated prior to the analysis.

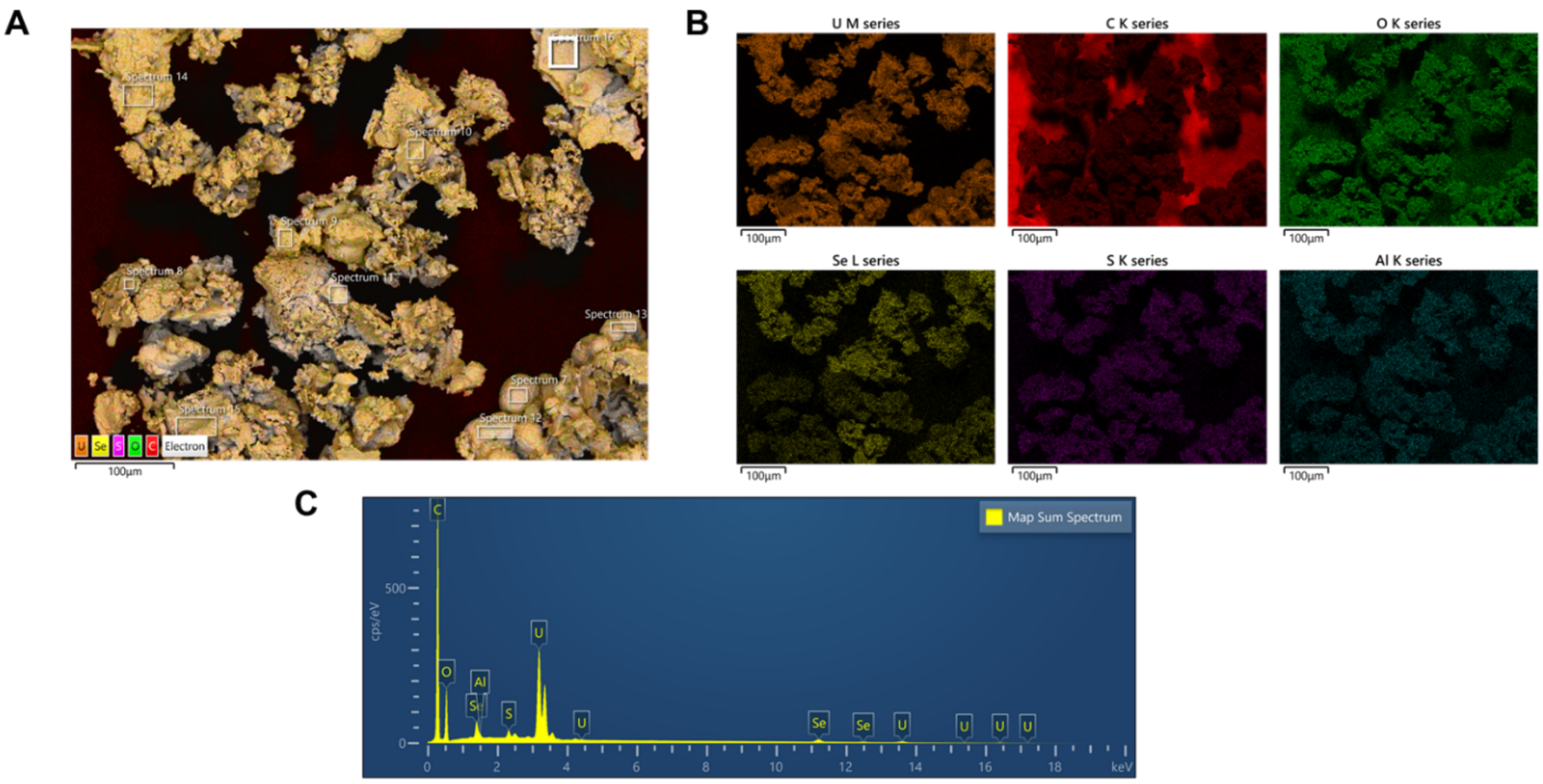

Map Sum Spectrum Element	Line Type	Weight %	Weight % Sigma	Atomic %
C	K series	44.70	0.03	75.38
O	K series	16.06	0.03	20.33
Al	K series	0.28	0.01	0.21
S	K series	0.57	0.00	0.36
Se	L series	2.65	0.01	0.68
U	M series	35.74	0.03	3.04
Total		100.00		100.00

Figure S66. Post-TGA SEM/EDS analysis of compound **3**. **(a)** Overall elemental mapping via FE-SEM/EDS imaging, **(b)** the distribution of specific elements in the elemental mapping, and **(c)** map sum spectrum. Material was placed on aluminum stub with double-sided carbon tape and not coated prior to the analysis.

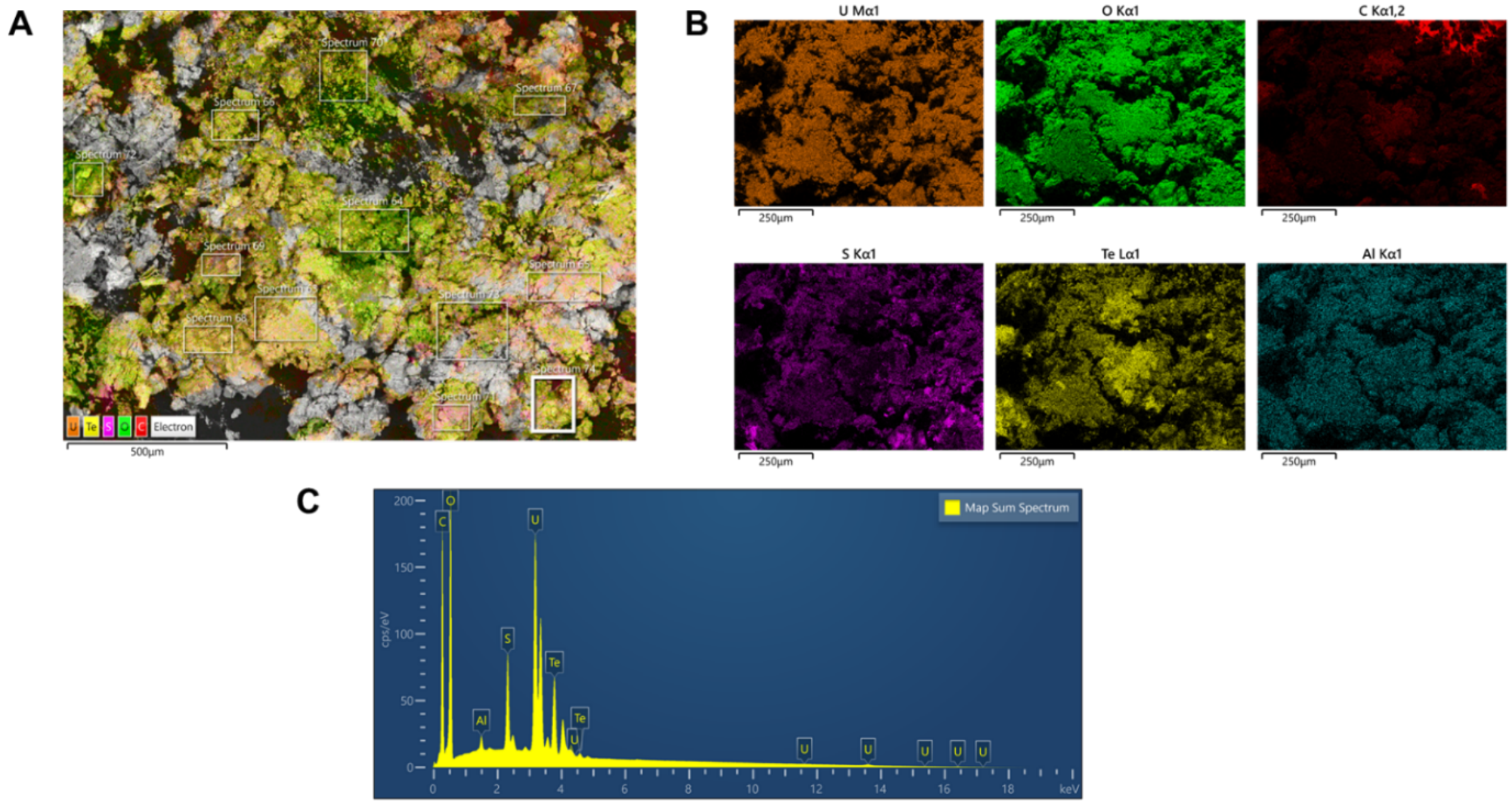

Map Sum Spectrum Element	Line Type	Weight %	Weight % Sigma	Atomic %
C	K series	22.68	0.04	49.64
O	K series	24.73	0.04	40.62
Al	K series	0.50	0.01	0.49
S	K series	3.11	0.01	2.55
Te	L series	13.53	0.03	2.79
U	M series	35.45	0.05	3.91
Total		100.00		100.00

Figure S67. Pre-TGA SEM/EDS analysis of compound **4**. **(a)** Overall elemental mapping via FE-SEM/EDS imaging, **(b)** the distribution of specific elements in the elemental mapping, and **(c)** map sum spectrum. Material was placed on aluminum stub with double-sided carbon tape and not coated prior to the analysis.

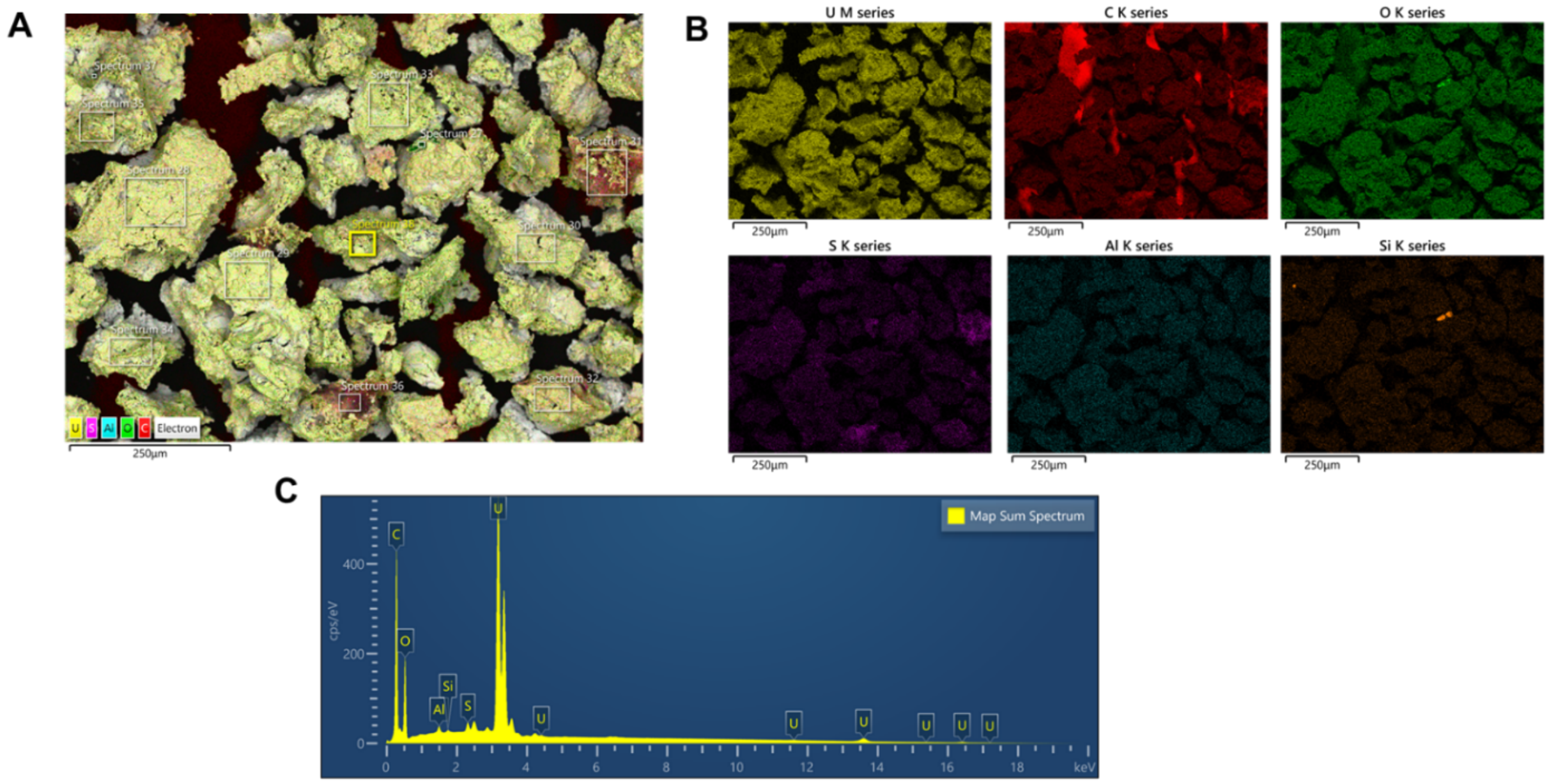

Map Sum Spectrum Element	Line Type	Weight %	Weight % Sigma	Atomic %
C	K series	21.87	0.03	59.72
O	K series	14.94	0.03	30.62
Al	K series	0.39	0.01	0.47
Si	K series	0.11	0.00	0.12
S	K series	0.47	0.00	0.49
U	M series	62.23	0.03	8.58
Total		100.00		100.00

Figure S68. Post-TGA SEM/EDS analysis of compound **4**. **(a)** Overall elemental mapping via FE-SEM/EDS imaging, **(b)** the distribution of specific elements in the elemental mapping, and **(c)** map sum spectrum. Material was placed on aluminum stub with double-sided carbon tape and not coated prior to the analysis.

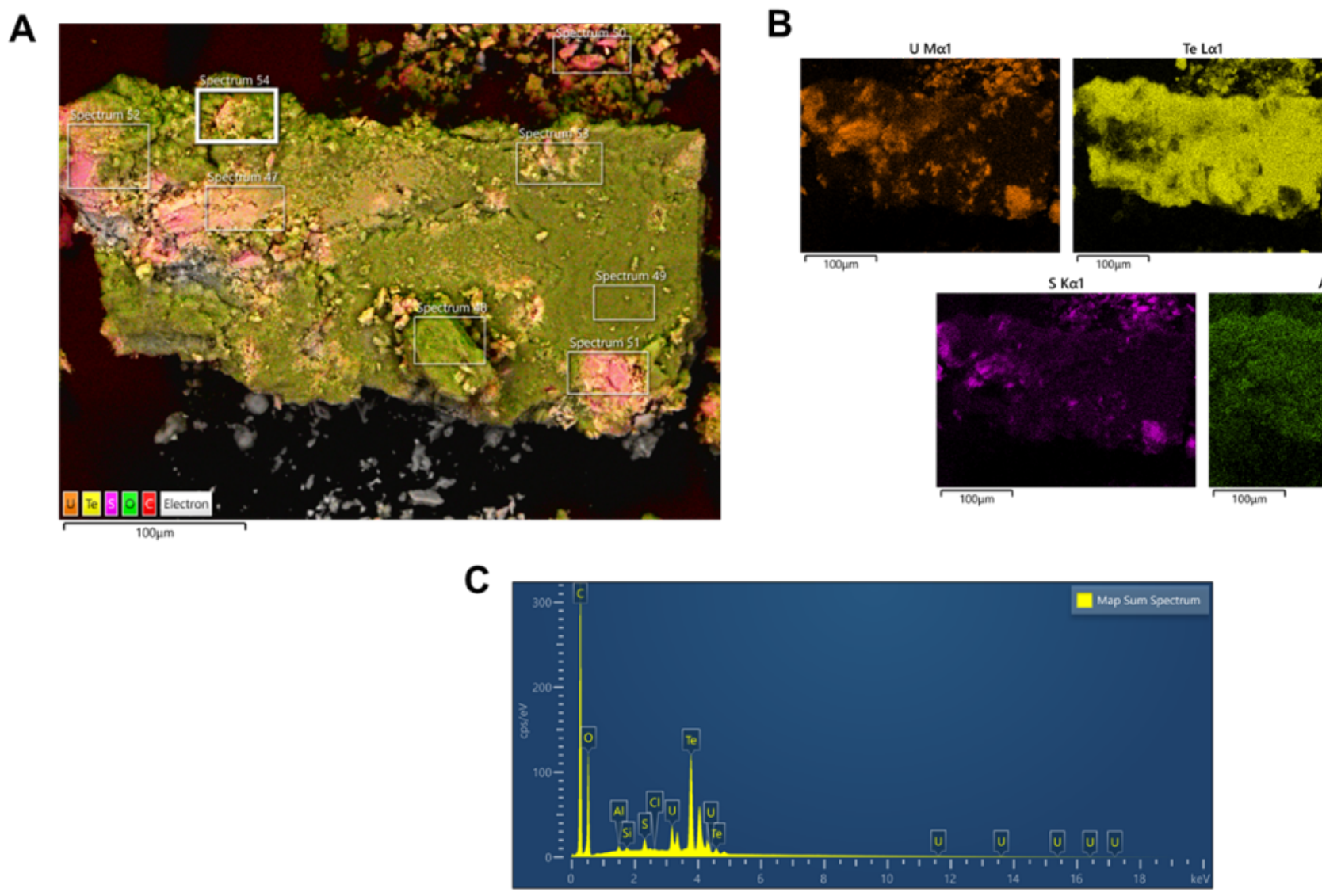

Map Sum Spectrum Element	Line Type	Weight %	Weight % Sigma	Atomic %
C	K series	42.55	0.05	70.40
O	K series	18.94	0.04	23.52
Al	K series	0.35	0.01	0.26
Si	K series	0.17	0.01	0.12
S	K series	0.71	0.01	0.44
Cl	K series	0.08	0.01	0.05
Te	L series	29.24	0.04	4.55
U	M series	7.96	0.03	0.66
Total		100.00		100.00

Figure S69. Pre-TGA SEM/EDS analysis of physical mixture of (1:1) U(VI)–2,5-TDC-DMSO with 2,5-TeDC. **(a)** Overall elemental mapping via FE-SEM/EDS imaging, **(b)** the distribution of specific elements in the elemental mapping, and **(c)** map sum spectrum. Material was placed on aluminum stub with double-sided carbon tape and not coated prior to the analysis.

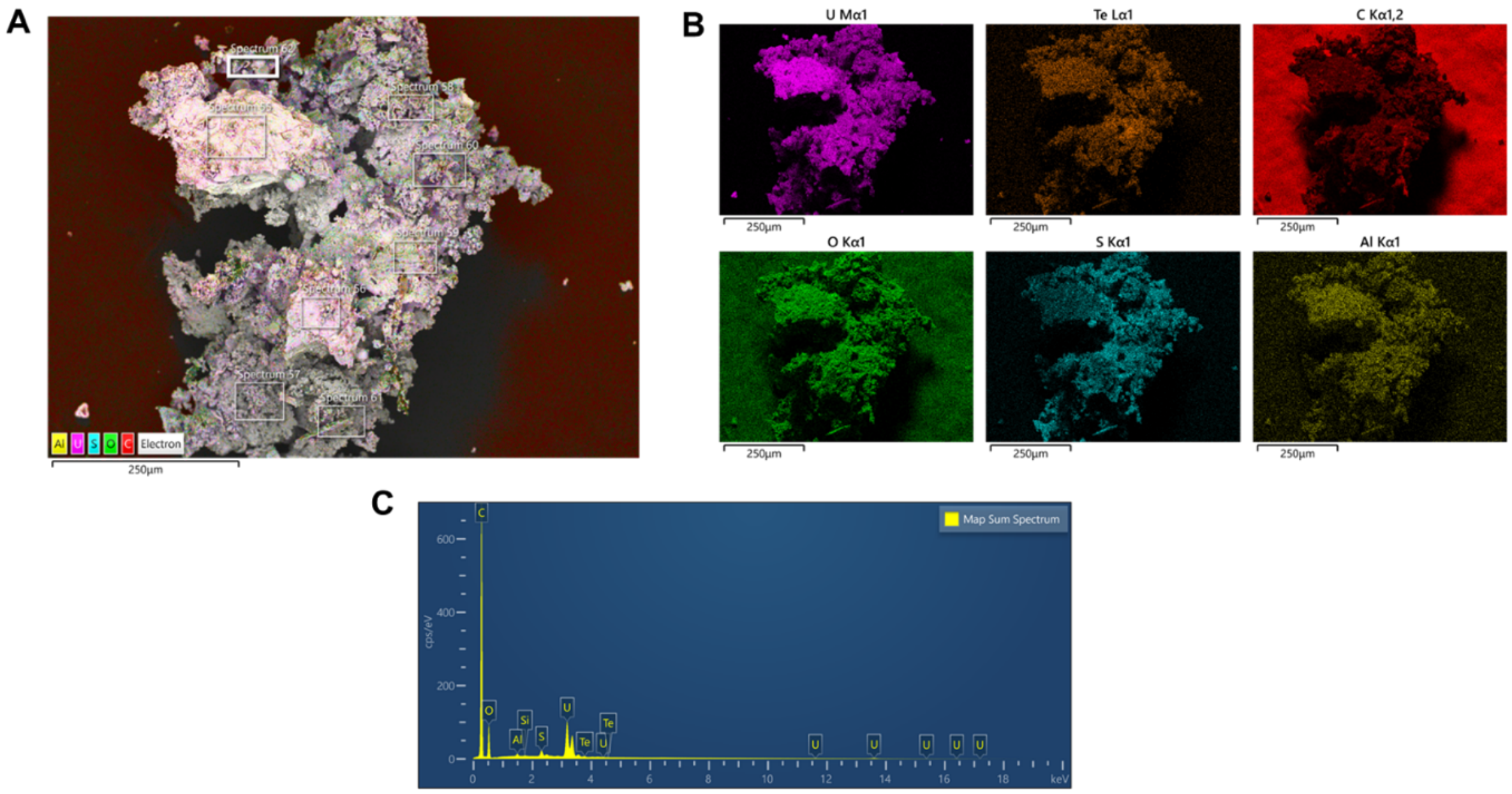

Map Sum Spectrum Element	Line Type	Weight %	Weight % Sigma	Atomic %
C	K series	59.89	0.05	80.67
O	K series	17.11	0.05	17.30
Al	K series	0.27	0.01	0.16
Si	K series	0.04	0.00	0.02
S	K series	0.63	0.01	0.32
Te	L series	0.53	0.01	0.07
U	M series	21.53	0.04	1.46
Total		100.00		100.00

Figure S70. Post-TGA SEM/EDS analysis of physical mixture of (1:1) U(VI)–2,5-TDC-DMSO with 2,5-TeDC. **(a)** Overall elemental mapping via FE-SEM/EDS imaging, **(b)** the distribution of specific elements in the elemental mapping, and **(c)** map sum spectrum. Material was placed on aluminum stub with double-sided carbon tape and not coated prior to the analysis.

Topological Analysis (ToposPro)

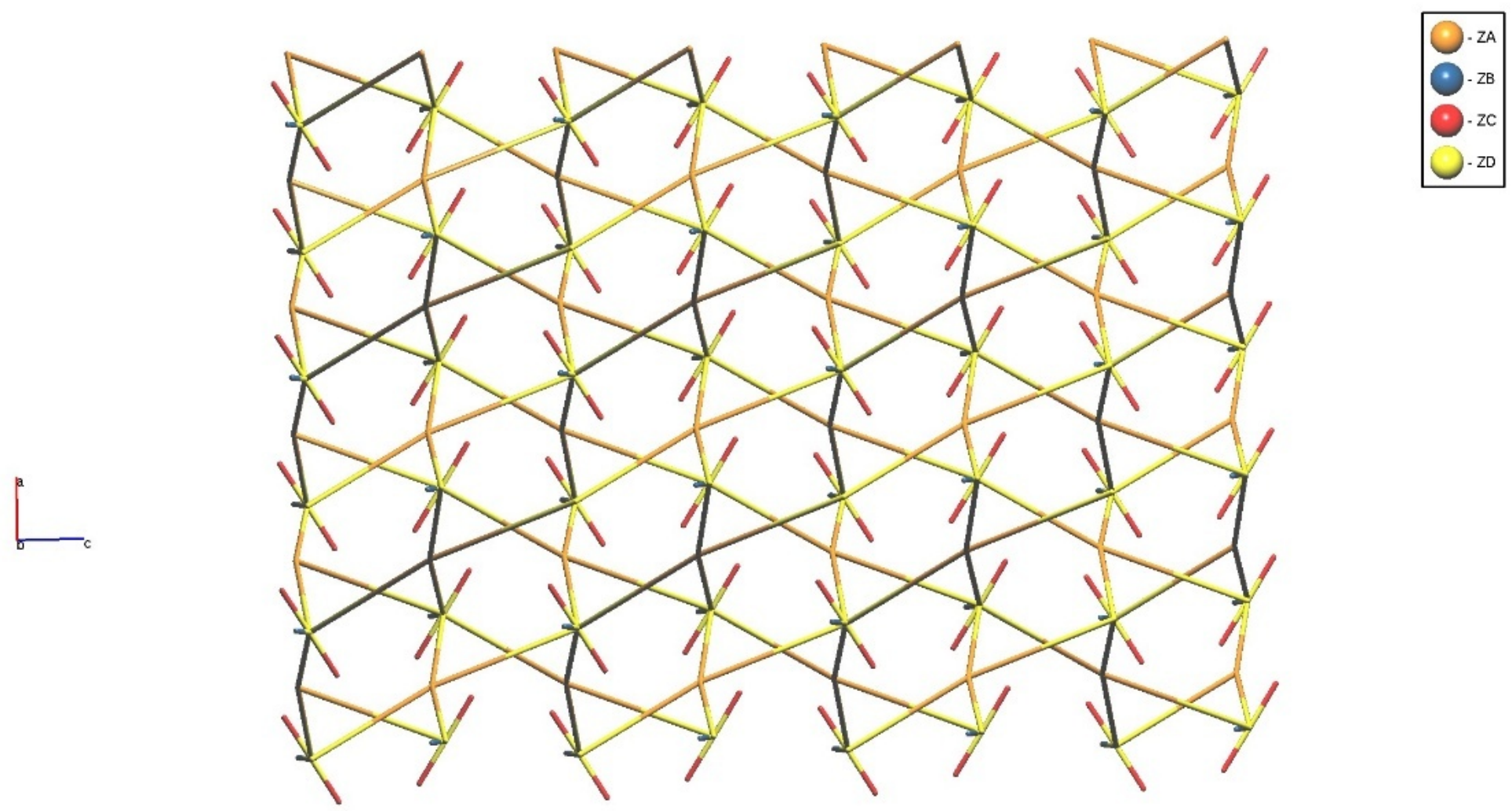

Figure S71. Topological representation of the framework of compound **1** where nodes are ZA: $C_6H_2O_4Se$, ZB: C_3H_7NO, ZC: O and ZD: U. The underlying net is classified as 4,4T150 and the projection is shown highlighting connectivity in the *ac*-plane.

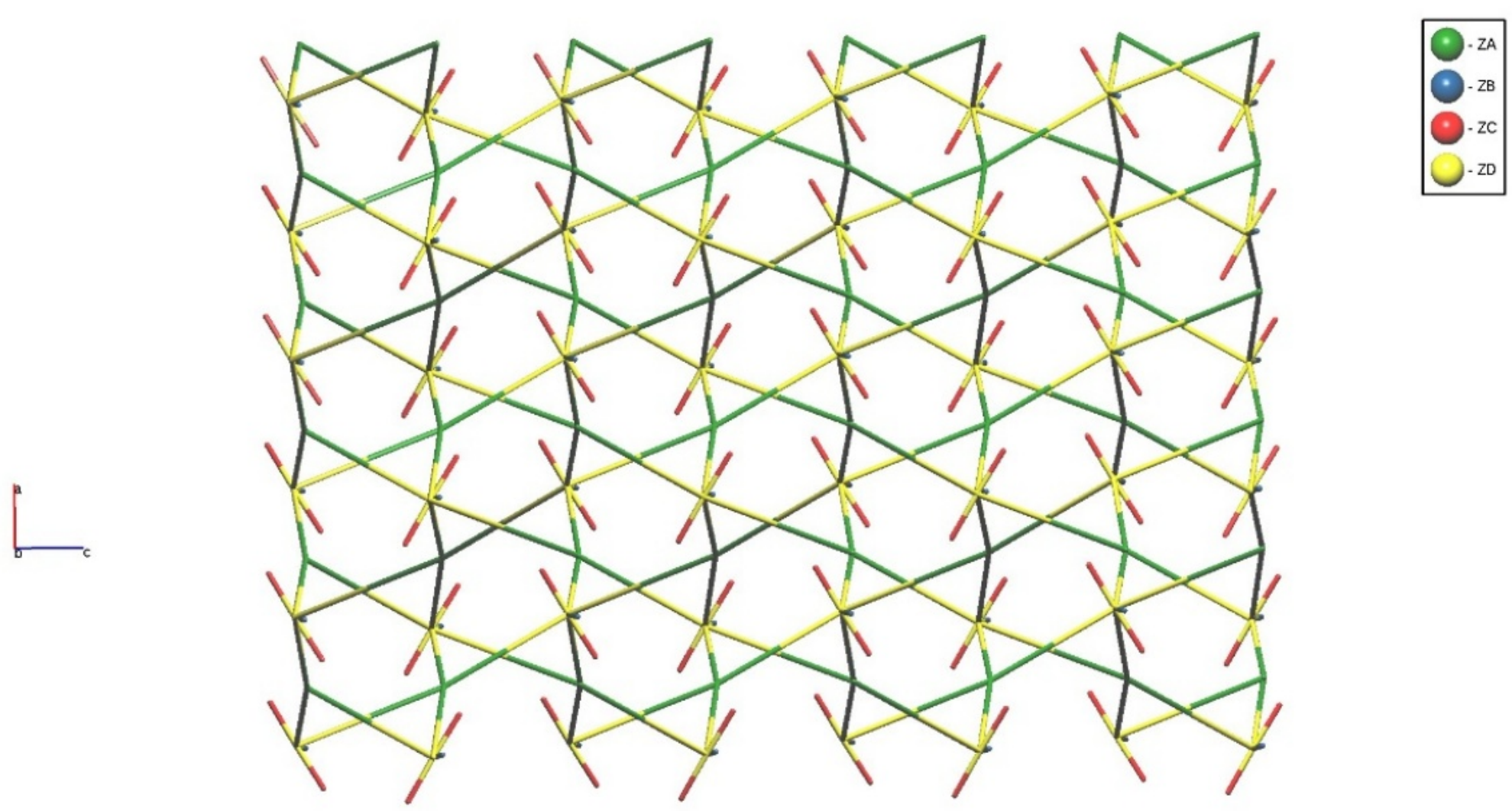

Figure S72. Topological representation of the framework of compound **2** where nodes are ZA: $C_6H_2O_4Te$, ZB: C_3H_7NO, ZC: O and ZD: U. The underlying net is classified as 4,4T150 and the projection is shown highlighting connectivity in the *ac*-plane.

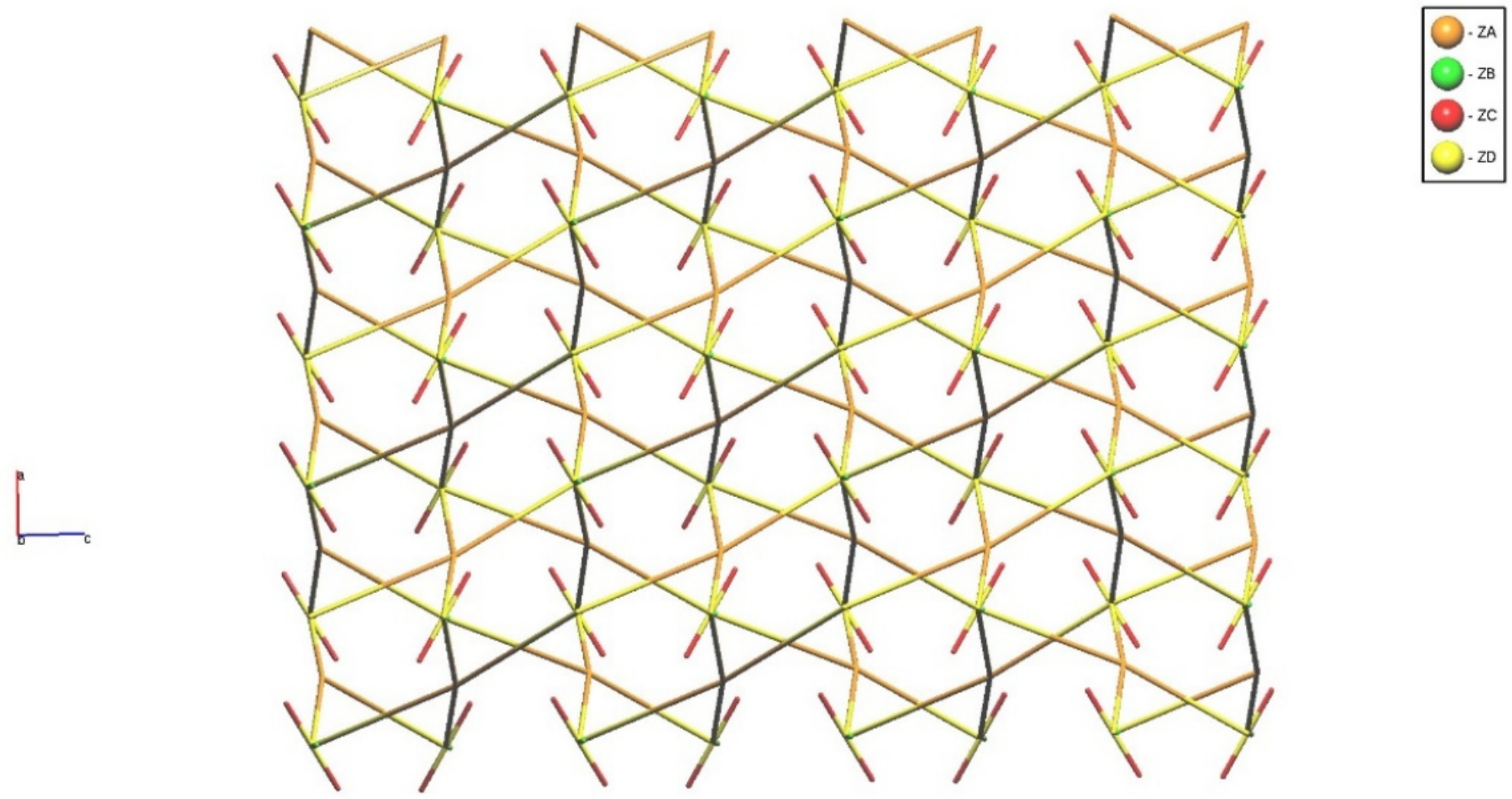

Figure S73. Topological representation of the framework of compound **3** where nodes are ZA: $C_6H_2O_4Se$, ZB: C_2H_6OS, ZC: O and ZD: U. The underlying net is classified as 4,4T150 and the projection is shown highlighting connectivity in the *ac*-plane.

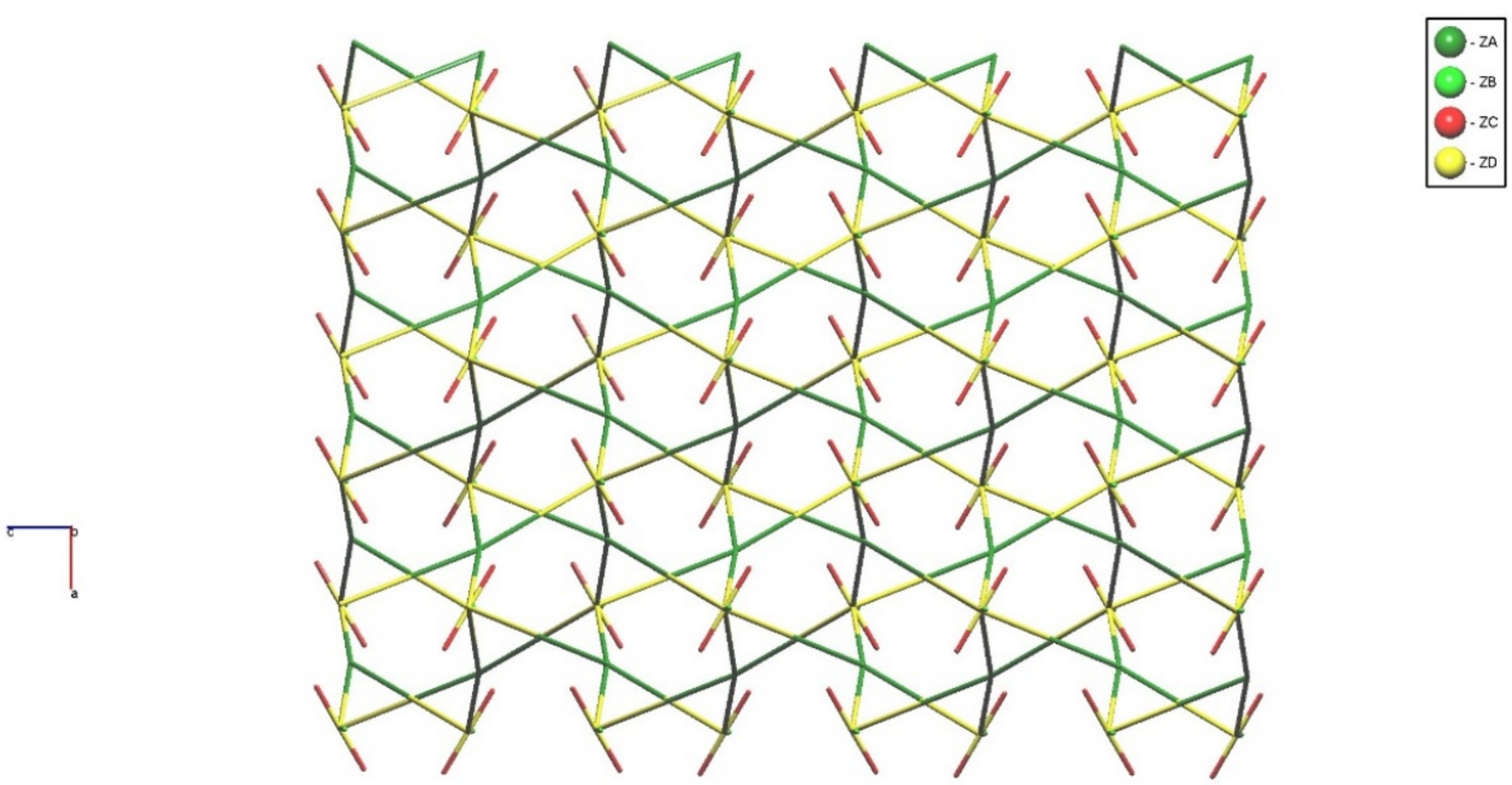

Figure S74. Topological representation of the framework of compound **4** where nodes are ZA: $C_6H_2O_4Te$, ZB: C_2H_6OS, ZC: O and ZD: U. The underlying net is classified as 4,4T150 and the projection is shown highlighting connectivity in the *ac*-plane.

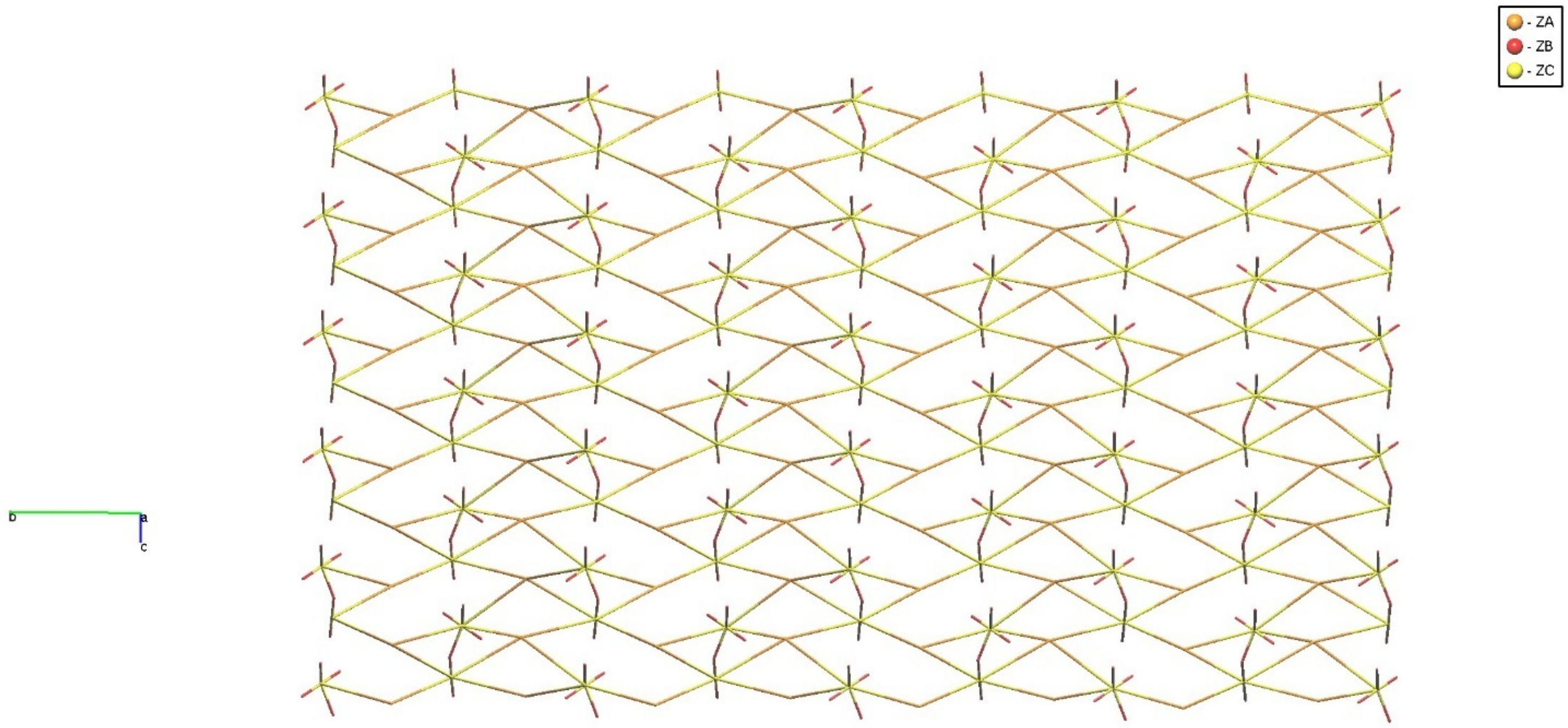

Figure S75. Topological representation of the framework of compound **5** where nodes are ZA: $C_6H_2O_4Se$, ZB: O, ZC: U. The underlying net is classified as 3,4^2,5-c and the projection is shown highlighting connectivity in the *bc*-plane.

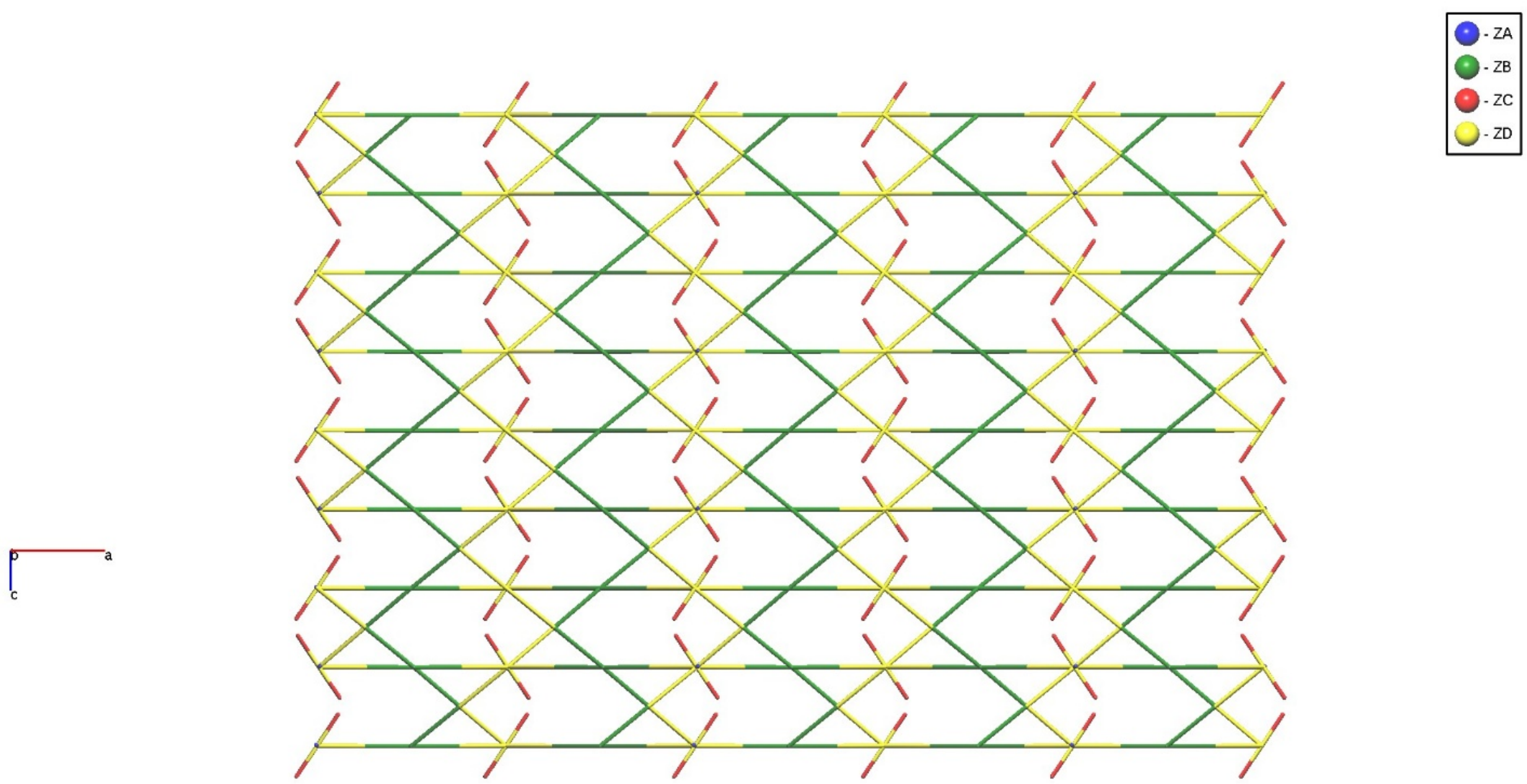

Figure S76. Topological representation of the framework of compound **6** where nodes are ZA: H_2O, ZB: $C_7H_4O_4Te$, ZC: O and ZD: U. The underlying net is classified as 4,4T3 and the projection is shown highlighting connectivity in the *ac*-plane.

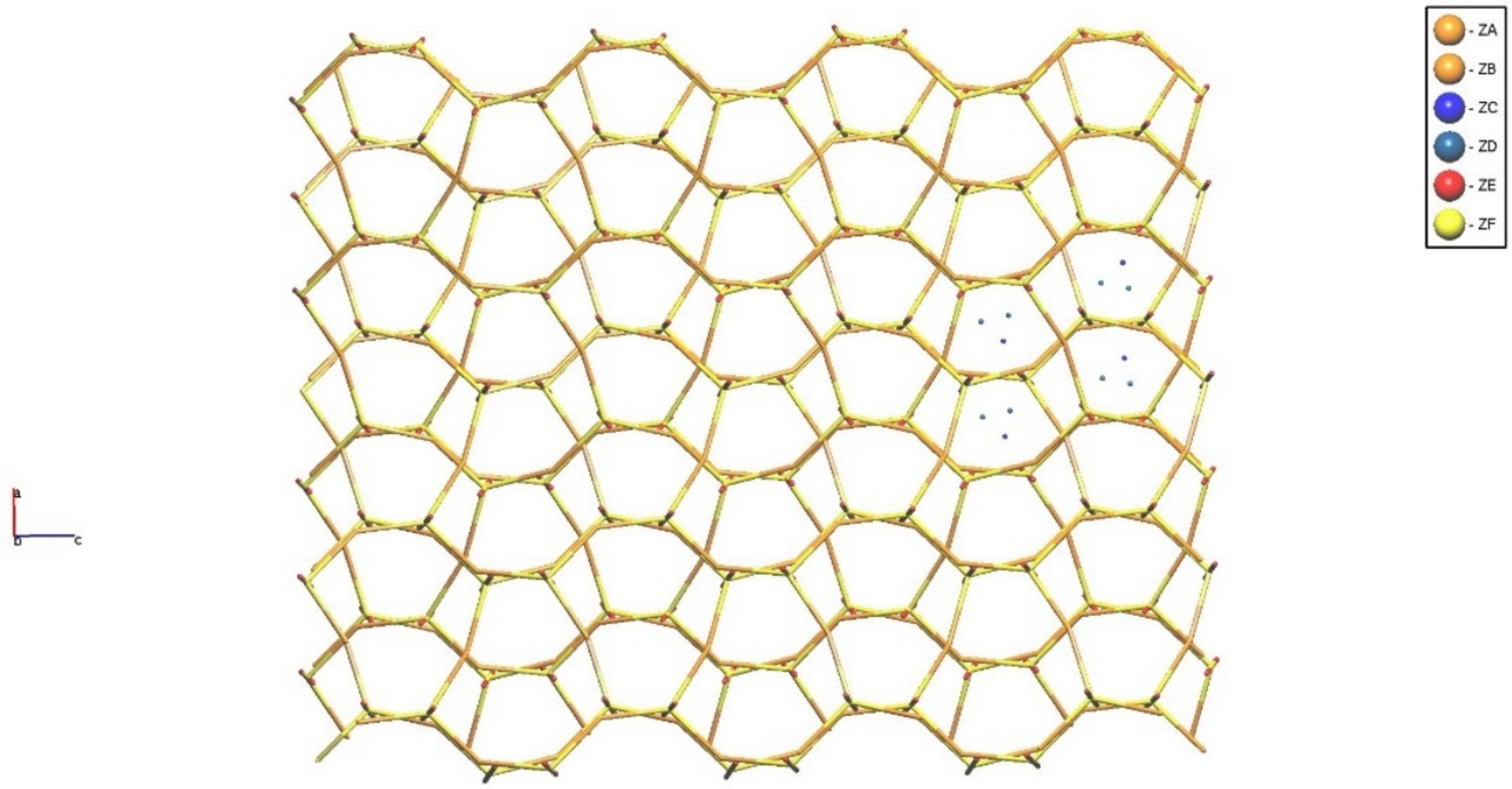

Figure S77. Topological representation of the framework of compound **7** where nodes are ZA: $C_6H_2O_4Se1$, ZB: $C_6H_2O_4Se2$, ZC: H_2O, ZD: C_2H_6N, ZE: O and ZF: U. The underlying net is classified as hcb, and the projection is shown highlighting connectivity in the *ac*-plane.

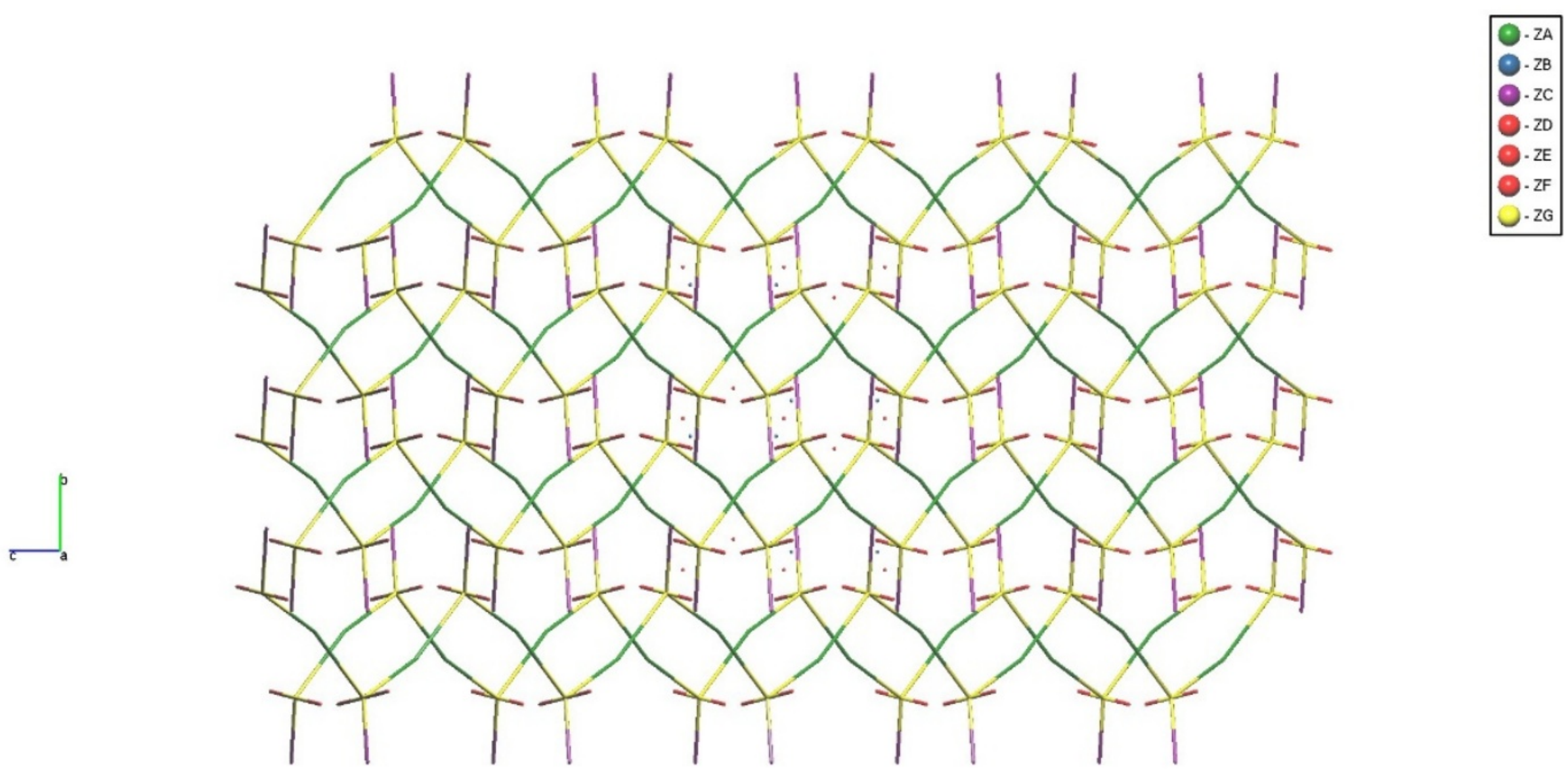

Figure S78. Topological representation of the framework of compound **8** where nodes are ZA: $C_6H_2O_4Te$, ZB: C_2H_6N, ZC: C_4O_2, ZD: O, ZE: O, ZF: O and ZG: U. The underlying net is classified as 2C1, and the projection is shown highlighting connectivity in the *bc*-plane.

References

(1) Mack, W. Synthesis of Tellurophene and its 2,5-Disubstituted Derivatives. *Angew. Chem. Int. Ed. Engl.* **1966**, *5* (10), 896-896.

(2) McCormick, T. M.; Jahnke, A. A.; Lough, A. J.; Seferos, D. S. Tellurophenes with Delocalized π-Systems and Their Extended Valence Adducts. *J. Am. Chem. Soc.* **2012**, *134* (7), 3542-3548.

(3) Carrera, E. I.; Seferos, D. S. Efficient halogen photoelimination from dibromo, dichloro and difluoro tellurophenes. *Dalton Trans.* **2015**, *44* (5), 2092-2096.
